# Enhanced Protection Against Toxicity of *Nemopilema nomurai* Venom Using a PEG-EGCG/Tetracycline Hydrochloride Micellar Nanocomplex

**DOI:** 10.3390/toxins18070278

**Published:** 2026-06-24

**Authors:** Jie Li, Yanan Hu, Yunfeng Qian, Sai Luo, Juxingsi Song, Shaoqian Zhu, Minglei Wang, Huiliang Gan, Qianqian Wang, Liming Zhang

**Affiliations:** 1 Naval Special Medical Center, Naval Medical University, Shanghai 200433, China; lijie1992@smmu.edu.cn (J.L.); yananhu20192022@163.com (Y.H.); m15056344020@163.com (S.L.); song9935@163.com (J.S.); shaoqianzhu2018@163.com (S.Z.); wml22134190@zuaa.zju.edu.cn (M.W.); 2 The Third Affiliated Hospital, Naval Medical University, Shanghai 200433, China; money0814@163.com

**Keywords:** *Nemopilema nomurai*, venom, toxicity, neutralization, nanocomplex, PEG-EGCG/tetracycline hydrochloride

## Abstract

Jellyfish stings are the most common type of marine life injuries. However, at present, the treatment measures against jellyfish stings are mostly empirical and supportive, with uncertain therapeutic outcomes, and there is a lack of specific antidotes based on the toxic mechanism of jellyfish venom in clinical practice. In our previous study, polyphenol epigallocatechin-3-gallate (EGCG) was found to neutralize the toxicity of jellyfish *Nemopilema nomurai* venom (NnV) in vivo and in vitro. Herein we further demonstrated that EGCG exerted its antagonistic effect against NnV through inhibiting the oxidative stress, pro-apoptotic proteins, and systemic inflammatory responses. Subsequently, we constructed a polyethylene glycol (PEG)-EGCG/tetracycline hydrochloride (HTC) co-loaded micellar nanocomplex in order to enhance the stability and bioavailability of EGCG in vivo, which successfully integrated the membrane-repair function of PEG, the enzyme inhibitory effect of HTC and the antioxidant properties of EGCG. Notably, this micellar nanocomplex demonstrated significant protective effects against both functional damage and pathological alterations in a non-lethal NnV-envenomed mouse model. When administered 1 h after NnV envenomation, EGCG (40 mg/kg), HTC and PEG-EGCG (containing 40 mg/kg EGCG) only partially improved abnormal blood biochemical indicators and moderately alleviated histopathologic damage, and PEG-EGCG/HTC containing merely 8 mg/kg EGCG completely mitigated the toxic reactions in envenomed mice. In the preventive regimen, the administration of EGCG, HTC or PEG-EGCG 30 min before exposure showed no significant improvement in abnormal blood biochemical indicators and histopathologic damage, while PEG-EGCG/HTC could still significantly improve the functional impairments and histopathologic damage of the heart and liver in NnV-envenomed mice. These findings suggest the clinical translational potential of PEG-EGCG/HTC against jellyfish envenomation.

## 1. Introduction

In recent years, driven by factors such as climate warming and marine eutrophication, jellyfish blooms have been occurring frequently in global waters [1,2], severely threatening marine ecosystems and coastal economic development [3]. Jellyfish stings can cause local pain and swelling, and in severe cases, may lead to respiratory failure, cardiac arrest, and even death [4]. About 100 people die from jellyfish stings every year [5]. Jellyfish venom has complex components and extensive biological activities, among which hemolytic toxicity, enzyme activity and myocardial cytotoxicity are the main factors leading to a series of pathophysiological processes and even death following jellyfish stings [4,6,7,8]. As one of the world’s largest jellyfish species, *Nemopilema nomurai* causes numerous jellyfish sting incidents every year [9]. Based on the clinical symptoms of the sting patients, it is generally acknowledged that *Nemopilema nomurai* is more venomous than *Cyanea nozakii*, another poisonous scyphozoan distributed in China [4]. In our early efforts to neutralize the toxic effects of jellyfish *N. nomurai* venom (NnV), we screened potential antagonists and ultimately identified the polyphenol epigallocatechin-3-gallate (EGCG), which completely antagonized the hemolytic toxicity and matrix metalloproteinase (MMP) activity of NnV in vitro [10].

EGCG is a natural phenolic substance present in foods and beverages, particularly in tea. It exhibits a broad spectrum of biological activities, including antioxidant, antimicrobial, anti-obesity, anti-inflammatory, and anti-cancer properties [11,12,13]. Moreover, EGCG has been shown to antagonize the toxic effects of snake venom (Chinese cobra phospholipase A2), botulinum toxin, tetanus toxin and Shiga toxin [14,15,16]. However, while our previous research demonstrated that EGCG effectively antagonized the NnV toxicity in vitro, its efficacy in vivo was limited to a narrow therapeutic time window. According to the literature review, EGCG has low bioavailability [17] and is prone to self-oxidation [18,19]. Moreover, EGCG readily aggregates and interacts with blood cells and plasma proteins, resulting in loss of activity, and is also susceptible to enzymatic degradation [20].

To enhance the bioavailability of EGCG and improve its therapeutic efficacy against jellyfish venom, various drug delivery strategies have been explored [21,22]. In recent years, the development of nanoscale systems using EGCG as a carrier has garnered significant attention in drug delivery and cancer therapy, owing to its unique biological activity and structural characteristics [23]. Importantly, both non-covalent and covalent interactions between EGCG and other substances contribute significantly to the improved performance of these nanoparticles [24]. For example, Wu et al. designed a fluorinated-coordinated EGCG delivery system, which can be self-assembled into nanoparticles with chemical drugs (e.g., sorafenib, gemcitabine, doxorubicin), nucleic acids (e.g., siRNA), peptides and proteins, thereby enhancing drug targeting and enabling sustained release [25]. Similarly, Yongvongsoontorn et al. developed a sunitinib-loaded nano-micelle complex using polyethylene glycol (PEG)-EGCG as a carrier, which specifically inhibited the tumor cell proliferation [26]. These innovative delivery systems not only serve as effective drug carriers, but also capitalize on the intrinsic bioactivity of EGCG as a carrier component.

Based on the excellent properties of EGCG, we selected PEG-EGCG, a conjugate of PEG and EGCG, as a carrier platform for the delivery of tetracycline hydrochloride (HTC) to determine the effect of PEG-EGCG/HTC as an antivenom against NnV. This approach was informed by several observations: high concentrations of PEG, a known membrane-repair agent, could completely inhibit the hemolytic toxicity of jellyfish venom [27]; moreover, HTC can inhibit the activities of MMP-2 and MMP-9, protect human keratinocytes and mouse fibroblasts from NnV-induced cytotoxicity, and alleviate the skin damage in a jellyfish envenomation model [28].

In this study, we further investigated the biosafety and therapeutic efficacy of PEG-EGCG/HTC in vivo. Our results demonstrate that PEG-EGCG/HTC exhibits significantly superior antivenom activity compared to EGCG, PEG-EGCG, or HTC alone, highlighting its clinical translational potential for the prevention and treatment of jellyfish envenomation.

## 2. Results and Discussion

### 2.1. EGCG Inhibited Oxidative Stress in NnV-Exposed Cells

**Figure 1:**
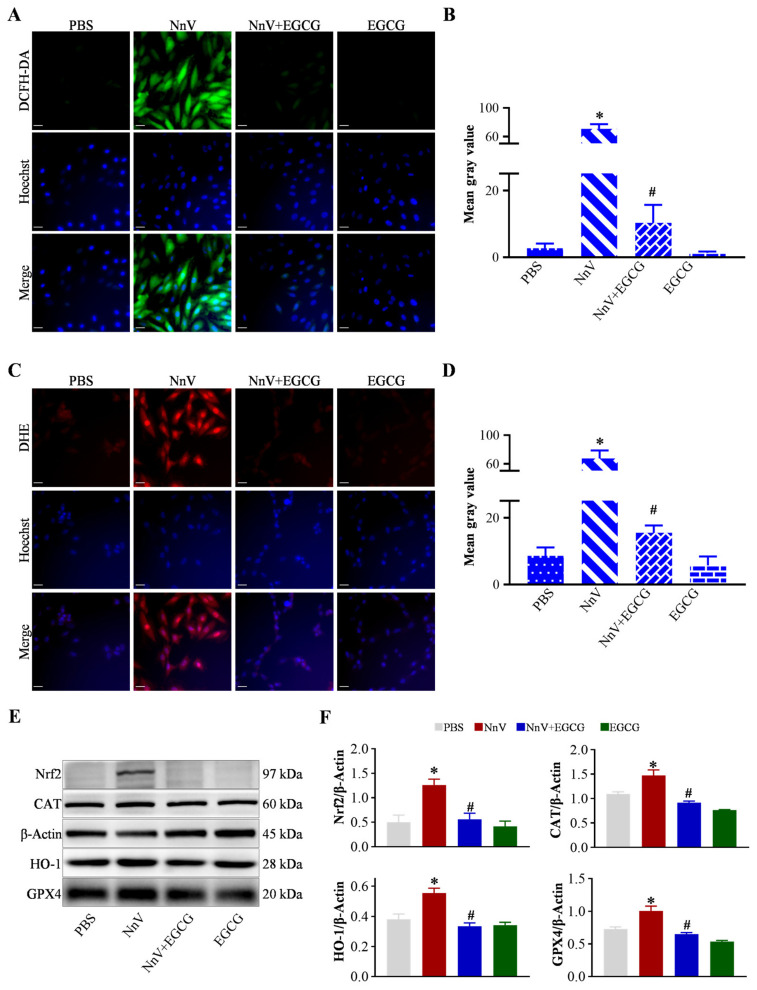
Effects of different interventions on intracellular oxidative stress in H9C2 cells. (**A**) Effects of different interventions on intracellular oxidative stress (detected by DCFH-DA) in H9C2 cells by fluorescence microscopy (Scale bar = 50 μm). (**B**) Quantitative analysis of fluorescence intensity in different intervention groups in Subfigure (**A**). (**C**) Effects of different interventions on intracellular oxidative stress (detected by DHE) in H9C2 cells by fluorescence microscopy (Scale bar = 50 μm). (**D**) Quantitative analysis of fluorescence intensity in different intervention groups in Subfigure (**C**). (**E**,**F**) Representative Western blot images and relative quantitative analysis of oxidative stress-related proteins in H9C2 cells under different intervention conditions. (*n* = 3, mean ± SD. * *p* < 0.05 versus PBS group, # *p* < 0.05 versus NnV group).

Given that jellyfish venom can induce apoptosis and cell cycle arrest, which are closely related to oxidative stress damage [29,30], and considering the known biological activities of EGCG, we reasonably hypothesize that EGCG may also antagonize the toxic effects of jellyfish venom by suppressing apoptosis, oxidative stress and inflammatory response. Since a previous study has confirmed that NnV suppresses H9C2 cell viability with an IC_50_ of 260 ng/mL [10], this section further investigated the cytotoxicity of NnV and the underlying mechanism by which EGCG inhibits NnV-induced cytotoxicity. After 4 h of exposure to NnV (260 ng/mL), cardiomyocytes loaded with the DCFH-DA fluorescent probe showed a significant increase in green fluorescence intensity (Figure 1A,B), indicating an increase in intracellular H_2_O_2_ levels. Similarly, after 4 h of NnV (260 ng/mL) treatment, intracellular loading of the DHE fluorescent probe resulted in a marked increase in red fluorescence intensity (Figure 1C,D), reflecting an accumulation of superoxide anion (•O^2−^). Both H_2_•O_2_− and •O^2−^ are major reactive oxygen species (ROS), and their excessive generation can cause severe damage to cells [31]. In contrast, treatment with EGCG significantly attenuated both green and red fluorescence signals (Figure 1A–D), indicating that EGCG effectively suppressed NnV-induced ROS production.

Subsequently, we analyzed the oxidative stress-related proteins. Nuclear factor erythroid 2-related factor 2 (Nrf2) is a key transcription factor in the endogenous antioxidant response, and interacts with heme oxygenase-1 (HO-1) to constitute the crucial Nrf2/HO-1 antioxidant pathway [32]. Under NnV exposure, expression levels of Nrf2, CAT, HO-1 and GPX4 were significantly elevated, whereas EGCG intervention reversed their upregulation (Figure 1E,F).

Under physiological conditions, Kelch-like ECH-associated protein 1 (Keap1) mediates rapid ubiquitination and degradation of Nrf2, thereby suppressing its transcriptional activity in non-stressful conditions [32,33]. However, under NnV exposure, the massive ROS production in cardiomyocytes inhibited Nrf2 ubiquitination and degradation, leading to cytoplasmic accumulation and subsequent nuclear translocation of Nrf2. In the nucleus, Nrf2 acts as a transcription factor to upregulate antioxidant enzymes, such as CAT, HO-1 and GPX4 in NnV-exposed cells. EGCG intervention markedly suppressed ROS production in NnV-exposed cells and restored the expression of oxidative stress-related proteins to near-normal levels. These findings suggest that EGCG protects cardiomyocytes by alleviating NnV-induced oxidative stress.

### 2.2. EGCG Inhibited Apoptosis in NnV-Exposed Cells

**Figure 2:**
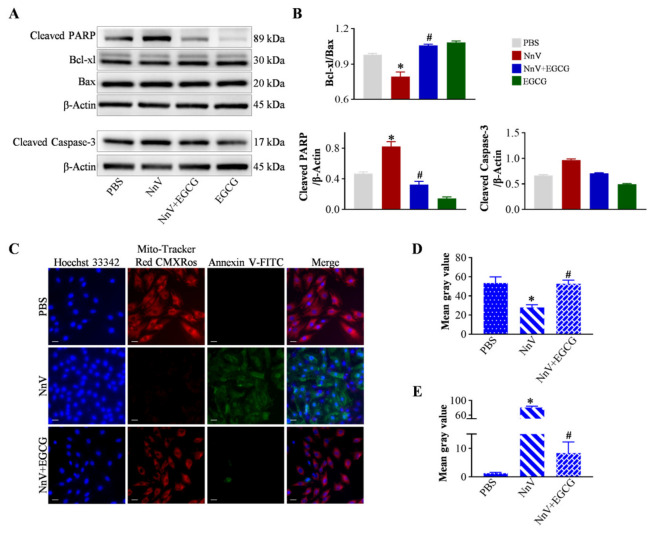
Effects of EGCG on apoptosis of H9C2 cells induced by NnV. (**A**,**B**) Representative Western blot images and relative quantitative analysis of apoptosis-related factors in H9C2 cells under different intervention conditions. (**C**) Mitochondrial membrane potential and apoptosis fluorescence in H9C2 cells under different interventions (Scale bar = 50 μm). (**D**) Quantitative analysis of mitochondrial membrane potential fluorescence intensity in different intervention groups. (**E**) Quantitative analysis of apoptosis fluorescence intensity across intervention groups. (*n* = 3, mean ± SD. * *p* < 0.05 versus PBS group, # *p* < 0.05 versus NnV alone group).

Previous laboratory experiments have demonstrated that NnV can induce the generation of large amounts of ROS in H9C2 cells, and ROS-mediated oxidative stress injury is a significant mechanism that promotes apoptosis [34]. Therefore, this section further investigated the effects of NnV on apoptosis in H9C2 cells. DNA and mitochondria are common downstream effectors of oxidative stress injury [35]. Western blot results showed that NnV treatment significantly enhanced the degradation of intracellular DNA damage repair enzymes and markedly increased the level of cleaved PARP, indicating that NnV can induce cellular DNA damage and lead to apoptosis. Concurrently, the levels of cleaved Caspase-3 (an executioner protein in apoptosis) and the pro-apoptotic protein Bax were elevated, while the level of the anti-apoptotic protein Bcl-xl decreased, leading to a reduced Bcl-xl/Bax ratio (Figure 2A,B). The balance between Bax and Bcl-xl is a critical determinant of apoptosis [33,36]. Disruption of the Bcl-xl/Bax balance compromises mitochondrial membrane integrity, promotes cytochrome c release, activates caspases, and triggers the caspase cascade [37]. Caspase-3, the most critical downstream apoptotic executor in the caspase cascade, was also abnormally activated by NnV, further promoting apoptosis. However, after EGCG intervention, the expression levels of cellular DNA damage repair factors and apoptosis-related factors were restored to normal levels.

To observe apoptosis in H9C2 cells more intuitively, specific fluorescence labeling was used under a fluorescence microscope. Mito-Tracker Red CMXRos is a red fluorescent mitochondrial probe that specifically labels bioactive mitochondria within cells. Annexin V-FITC is a green fluorescent probe for detecting phosphatidylserine. When cells undergo apoptosis, phosphatidylserine translocates to the cytoplasmic face of the cell membrane, where it is recognized and bound by Annexin V-FITC. As shown in Figure 2C–E, weakened red fluorescence suggested decreased mitochondrial membrane potential and severe mitochondrial damage. Enhanced green fluorescence indicated phosphoacyl serine externalization on the H9C2 cell membrane. At the same time, nuclei stained blue with Hoechst 33342 appeared fragmented and condensed. These results clearly demonstrate that NnV can induce oxidative stress, DNA damage, and mitochondrial dysfunction in cells, subsequently activating downstream apoptotic signaling factors to promote cell death. In contrast, after EGCG intervention, no significant activation of related apoptotic factors was observed within the cells, indicating that EGCG inhibits the pro-apoptotic effects of NnV.

### 2.3. EGCG Inhibited Oxidative Stress in NnV-Envenomed Mice

Cellular experiments have proved that NnV can stimulate cardiomyocytes to produce excessive ROS, leading to oxidative stress injury at the cellular level. To further investigate oxidative stress status in NnV-envenomed mice and the underlying mechanism of EGCG intervention at the animal level, we analyzed the expression profiles of Nrf2, CAT, HO-1, and GPX4 in the heart and liver of the mice under different intervention conditions using Western blotting (WB) and immunohistochemical staining (IHC).

**Figure 3:**
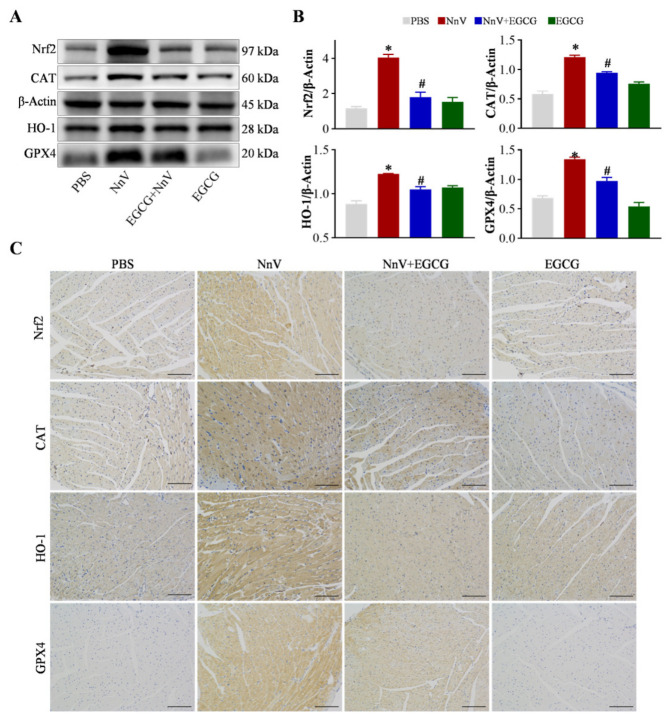
Effects of EGCG intervention on oxidative stress injury of the cardiac tissue in NnV-envenomed mice. (**A**,**B**) Representative Western blot images and relative quantitative analysis of oxidative stress-related proteins in cardiac tissues under different intervention conditions. (**C**) Distribution and expression of Nrf2, CAT, HO-1, and GPX4 in cardiac tissues under different intervention conditions (Scale bar = 100 μm). (*n* = 3, mean ± SD. * *p* < 0.05 versus PBS group, # *p* < 0.05 versus NnV alone group).

**Figure 4:**
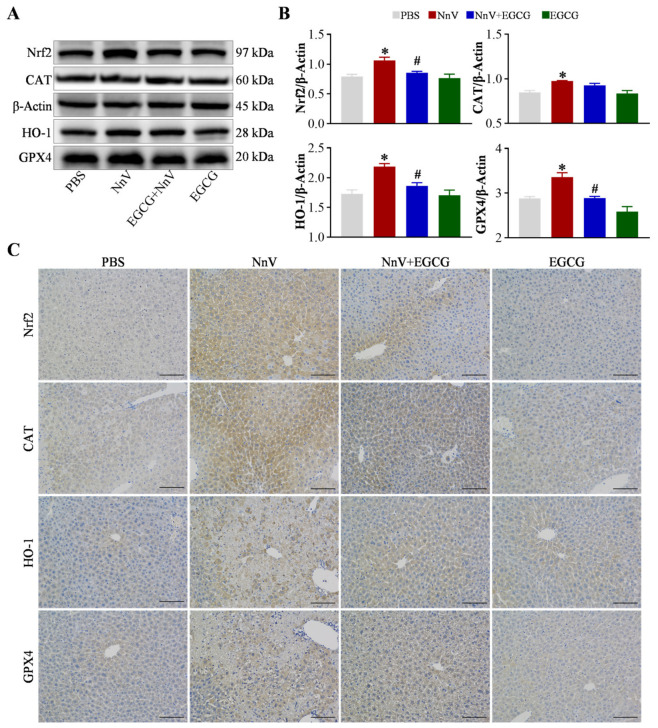
Effects of EGCG intervention on oxidative stress injury of the hepatic tissue in NnV-envenomed mice. (**A**,**B**) Representative Western blot images and relative quantitative analysis of oxidative stress-related proteins in hepatic tissues under different intervention conditions. (**C**) Distribution and expression of Nrf2, CAT, HO-1, and GPX4 in hepatic tissues under different intervention conditions (Scale bar = 100 μm). (*n* = 3, mean ± SD. * *p* < 0.05 versus PBS group, # *p* < 0.05 versus NnV alone group).

In NnV-envenomed mice, NnV significantly upregulated the expression levels of Nrf2, CAT, HO-1 and GPX4 in both heart (Figure 3) and liver tissues (Figure 4). The results indicated that NnV exposure induced oxidative stress, leading to substantial accumulation of Nrf2 and a compensatory increase in antioxidant enzymes. In contrast, EGCG intervention remarkedly reduced the levels of Nrf2, CAT, HO-1, and GPX4 compared with the NnV-alone group, with the most pronounced change observed in Nrf2, a key regulator of antioxidant signaling. These findings indicate that NnV induces severe ROS-mediated oxidative stress injury in cardiac and hepatic tissues, while EGCG effectively attenuates the damage induced by NnV.

### 2.4. EGCG Inhibited Inflammatory Response in NnV-Envenomed Mice

**Figure 5:**
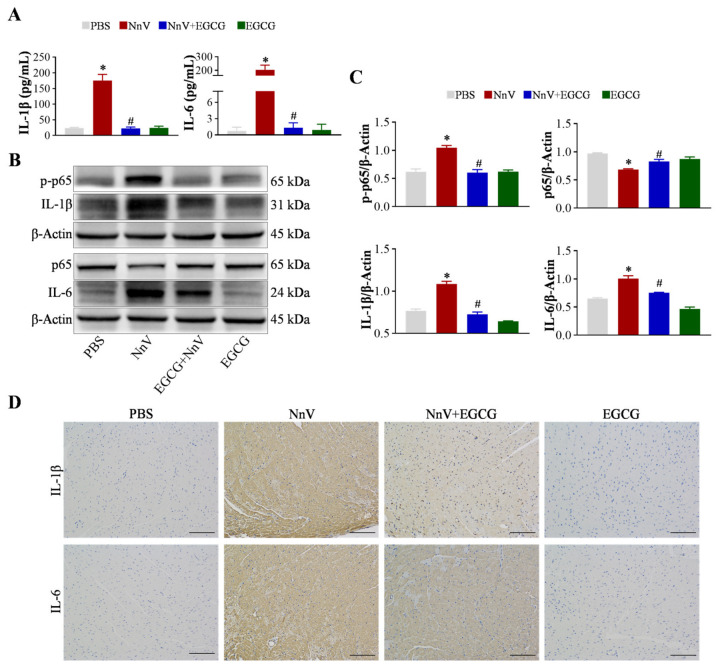
Effects of EGCG intervention on serum inflammatory factors and cardiac tissue inflammation in NnV-envenomed mice. (**A**) Serum levels of IL-1β and IL-6 (*n* = 6, mean ± SD). (**B**,**C**) Representative Western blot images and relative quantitative analysis of oxidative stress markers in cardiac tissues under different intervention conditions (*n* = 3, mean ± SD). (**D**) Distribution and expression of IL-1β and IL-6 in cardiac tissues (Scale bar = 100 μm). (* *p* < 0.05 versus PBS group, # *p* < 0.05 versus NnV group).

Given the oxidative stress and apoptosis induced by NnV, our attention was directed to the NF-κB (p65) signaling molecule that serves as a critical link between oxidative stress and apoptosis [32]. Furthermore, p65 is closely related to the body’s inflammatory response. Therefore, we first detected the inflammatory factors IL-1β and IL-6 in the serum by ELISA. As shown in Figure 5A, NnV exposure caused a marked elevation in serum levels of IL-1β and IL-6, indicating severe systemic inflammation in the envenomed mice. In contrast, the serum levels of IL-1β and IL-6 were significantly reduced after EGCG treatment compared with the NnV-alone group, suggesting that EGCG intervention suppressed the abnormal increase in inflammatory factors and alleviated the systemic inflammation in envenomed mice.

**Figure 6:**
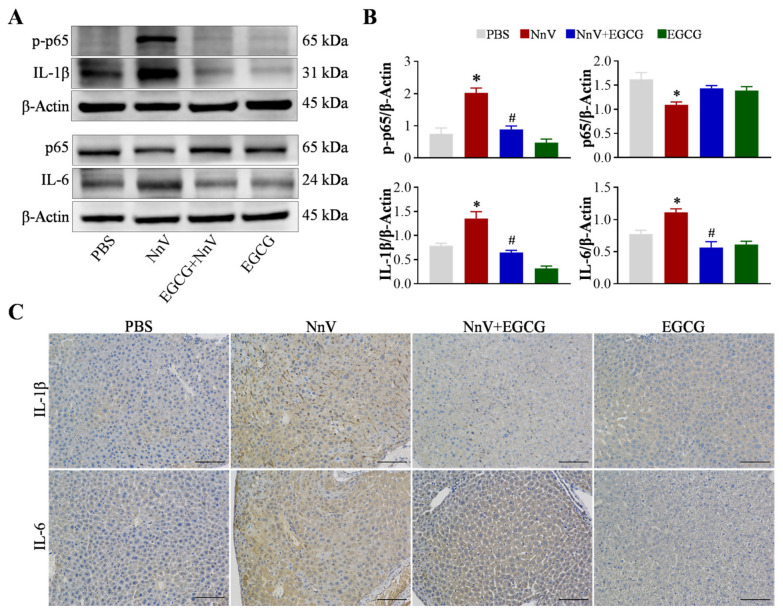
Effects of EGCG intervention on inflammatory response in hepatic tissue of NnV-envenomed mice. (**A**,**B**) Representative Western blot images and relative quantitative analysis of oxidative stress markers in hepatic tissues under different intervention conditions. (**C**) Distribution and expression of IL-1β and IL-6 in hepatic tissues (Scale bar = 100 μm). (*n* = 3, mean ± SD. * *p* < 0.05 versus PBS group, # *p* < 0.05 versus NnV group).

WB and IHC analyses further revealed that, compared with the PBS group, the expression level of phosphorylated p65 (p-p65) was significantly increased in both heart (Figure 5B,C) and liver (Figure 6A,B) tissues of NnV-envenomed mice. However, EGCG intervention significantly decreased p-p65 expression in the heart and liver. Concurrently, the expression levels of inflammatory mediators IL-1β and IL-6 in the heart (Figure 5B–D) and liver (Figure 6A–C) of the NnV group were higher than those in the PBS group, which was consistent with the up-regulation of inflammatory factors in serum, while EGCG intervention significantly reduced the expression of IL-1β and IL-6 in the heart and liver. These results indicate that an obvious inflammatory response is present in the heart, liver and even the whole body of NnV-envenomed mice, and that EGCG can alleviate the inflammatory injury by inhibiting NF-κB phosphorylation.

### 2.5. EGCG Inhibited Apoptosis in the Heart and Liver Tissues of NnV-Envenomed Mice

Preliminary experimental results have revealed that there was obvious parenchymal cell death in both the heart and liver of NnV-envenomed mice [10], accompanied by significantly elevated biochemical indicators of cardiac and hepatic function. Given the in vitro pro-apoptotic effect of NnV, we further explored the impact of NnV on the apoptosis process at the tissue level.

**Figure 7:**
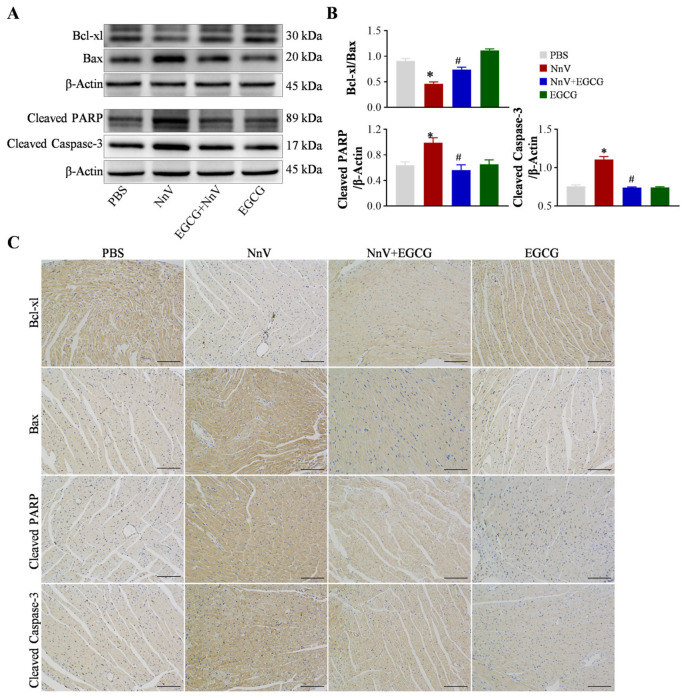
Effects of EGCG intervention on apoptosis in cardiac tissue of NnV-envenomed mice. (**A**,**B**) Representative Western blot images and relative quantitative analysis of apoptotic factors in cardiac tissues under different intervention conditions. (**C**) Distribution and expression of cleaved Caspase-3, cleaved PARP, Bax, and Bcl-xl in cardiac tissues (Scale bar = 100 μm). (*n* = 3, mean ± SD. * *p* < 0.05 versus PBS group, # *p* < 0.05 versus NnV group).

**Figure 8:**
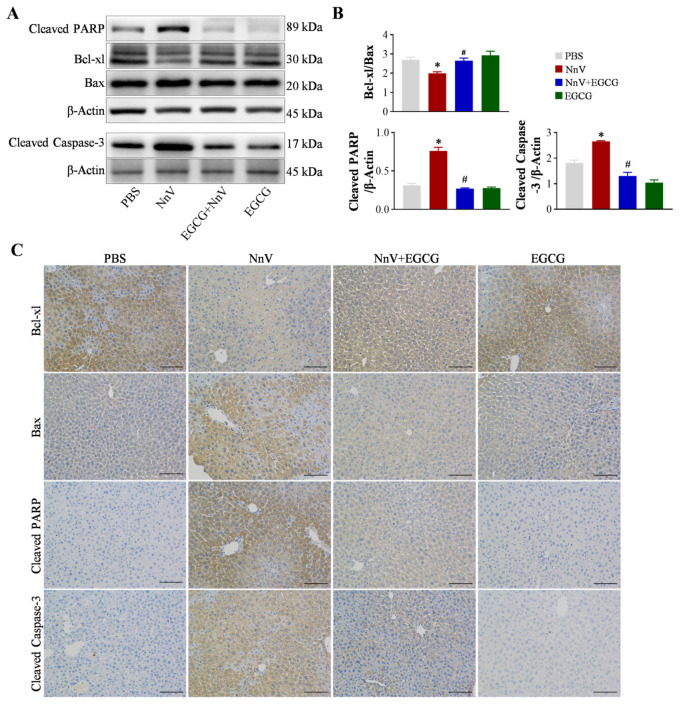
Effects of EGCG intervention on apoptosis in hepatic tissue in NnV-envenomed mice. (**A**,**B**) Representative Western blot images and relative quantitative analysis of apoptotic factors in hepatic tissues under different intervention conditions. (**C**) Distribution and expression of cleaved Caspase-3, cleaved PARP, Bax, and Bcl-xl in hepatic tissues (Scale bar = 100 μm). (*n* = 3, mean ± SD. * *p* < 0.05 versus PBS group, # *p* < 0.05 versus NnV group).

Western blot analysis revealed that, compared with the PBS group, the hearts (Figure 7A,B) and livers (Figure 8A,B) of NnV-envenomed mice exhibited increased expression of cleaved Caspase-3, cleaved PARP, and Bax, along with decreased expression of Bcl-xl. Following EGCG intervention, the expression levels of cleaved Caspase-3, cleaved PARP, and Bax decreased, while Bcl-xl expression level increased. The ratio of Bcl-xl to Bax in the EGCG intervention group showed no significant difference compared to the PBS group. In addition, we also used immunohistochemical analysis to evaluate the expression and distribution of these four proteins in heart and liver tissues. The results indicated that NnV stimulated the expression of pro-apoptotic factors such as cleaved Caspase-3, cleaved PARP, and Bax in heart (Figure 7C) and liver (Figure 8C) tissues, while suppressed the anti-apoptotic protein Bcl-xl. However, these abnormal expression patterns induced by NnV were markedly improved by EGCG treatment. It was seen that NnV exerted a pro-apoptotic effect on parenchymal cells in the heart and liver, contributing to biochemical abnormalities and histopathological damage in envenomed mice, but EGCG treatment significantly counteracted these changes, ameliorating the dysregulation of apoptosis-related proteins and protecting cardiac and hepatic tissues from NnV-induced apoptotic injury.

In summary, jellyfish venom can stimulate both cultured cells and animal tissues to generate excessive ROS. On one hand, ROS directly damages mitochondria and DNA, triggering intrinsic apoptosis; on the other hand, ROS activates the NF-κB signaling pathway, exerting pro-apoptotic effects and inducing systemic inflammatory responses in NnV-envenomed mice. EGCG intervention significantly suppresses NnV-induced oxidative stress, pro-apoptotic effects, and inflammatory injury, thereby effectively antagonizing the toxic effects of jellyfish venom.

EGCG possesses a wide spectrum of biological activities, including antibacterial, anti-inflammatory, antidiabetic, anti-radiation, anti-obesity, and antitumor effects, which are attributed to its unique polyphenolic structure containing eight phenolic groups [38,39,40]. However, the clinical application of EGCG has been limited by its low bioavailability and susceptibility to various biotransformation reactions in the body (e.g., glucuronidation, methylation, and sulfonylation), leading to rapid inactivation in vivo [41]. Inspired by the work of Yongvongsoontorn et al. [26] in which PEG-EGCG was used as a nanocarrier with an intrinsic therapeutic effect to synergistically augment the efficacy of loaded drugs, and taking into account that tetracycline may prevent the jellyfish venom-mediated tissue damage as a metalloproteinase inhibitor [28], PEG-EGCG was herein used as a carrier to load tetracycline hydrochloride for counteracting jellyfish envenoming.

### 2.6. Synthesis and Characteristics of PEG-EGCG/HTC

**Figure 9:**
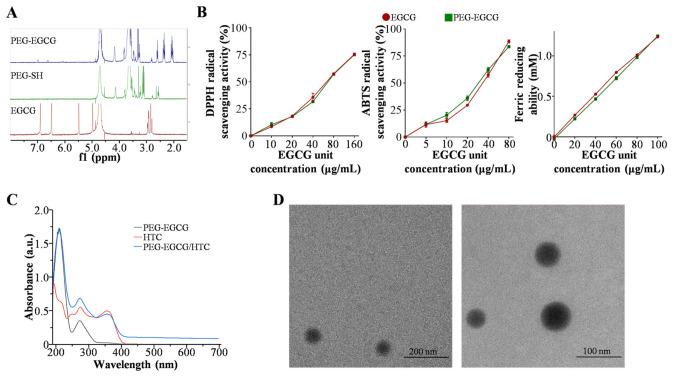
Characterization of PEG-EGCG/HTC nanomicelles. (**A**) ^1^H NMR spectrums of EGCG, PEG and PEG-EGCG. (**B**) Antioxidant activities of EGCG and PEG-EGCG. (**C**) UV absorption spectrums of PEG-EGCG, HTC and PEG-EGCG/HTC. (**D**) TEM images of PEG-EGCG/HTC nanomicelles. (*n* = 3, mean ± SD).

Combined with the inherent advantages of nanomaterials, such as high specific surface area, abundant active sites and good biocompatibility, such approaches offer promising new directions for the design of advanced drug delivery platforms. Accordingly, PEG-EGCG was synthesized via nucleophilic addition reaction of thiol-functionalized PEG to the pyrogallol moiety of EGCG [42]. The interaction between HTC and PEG-EGCG was confirmed by ^1^H NMR spectroscopy (Figure 9A). To evaluate whether the activity of EGCG changes after PEG conjugation, the antioxidant capacity of PEG-EGCG was measured by DPPH, ABTS and FRAP assays. The results showed no significant difference in antioxidant capacity between PEG-EGCG and free EGCG (Figure 9B), indicating that PEG conjugation preserves the inherent activity of EGCG. Ultraviolet absorption spectrum revealed that PEG-EGCG/HTC exhibited a characteristic absorption peak at 356 nm, identical to that of free HTC (Figure 9C). Concurrently, TEM imaging further confirmed the successful assembly of PEG-EGCG/HTC nanoparticles (Figure 9D).

DLS analysis showed that PEG-EGCG/HTC formed nanomicelles with an average hydrodynamic diameter of 147 ± 3.78 nm and a low polydispersity index (PDI), reflecting good colloidal dispersion, and the surface zeta potential of PEG-EGCG/HTC was −38.62 ± 1.86 mV, suggesting favorable in vitro stability (Table 1). Based on UV absorption measurements of PEG-EGCG, HTC, and PEG-EGCG/HTC, the encapsulation efficiency was calculated to be 84.26%, and the drug loading of HTC in PEG-EGCG/HTC was determined to be 6.73% (Table 1).

### 2.7. Distribution and Safety of PEG-EGCG/HTC

**Figure 10:**
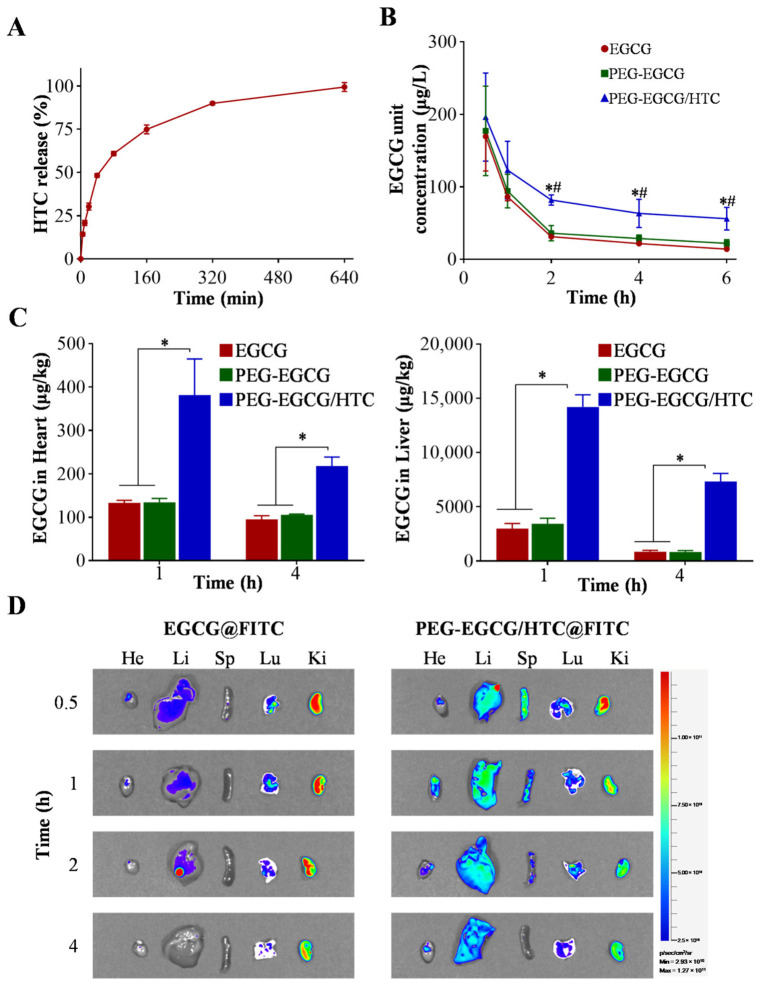
Metabolism and distribution of PEG-EGCG/HTC. (**A**) HTC release profile from PEG-EGCG/HTC in vitro. (*n* = 3, mean ± SD). (**B**) EGCG concentration in serum at different time points post-injection in mice (*n* = 6, mean ± SD. * *p* < 0.05 versus EGCG, # *p* < 0.05 versus PEG-EGCG). (**C**) EGCG levels in heart and liver tissues 1 h and 4 h post-injection in mice. (*n* = 6, mean ± SD. * *p* < 0.05). (**D**) Fluorescence imaging in the heart (He), liver (Li), spleen (Sp), lungs (Lu) and kidneys (Ki) was collected at 0.5 h, 1 h, 2 h, and 4 h ex vivo.

The in vitro release profile of HTC from PEG-EGCG/HTC was evaluated in PBS containing 10% mouse serum. The results showed that HTC was released continuously, reaching nearly 100% at approximately 10 h (Figure 10A). The content of EGCG in mouse serum, heart and liver was quantified by UPLC-MS/MS. It was shown that the circulation time of PEG-EGCG/HTC nanomicelles was significantly longer than that of free EGCG and PEG-EGCG in vivo. At 6 h post-injection, serum EGCG concentration in the PEG-EGCG/HTC group was approximately twofold higher than those in the free EGCG and PEG-EGCG groups (Figure 10B). Furthermore, at both 1 h and 4 h post-injection, EGCG level in heart and liver tissues of the PEG-EGCG/HTC group, particularly in the liver tissue, was significantly higher than those of the other two groups (Figure 10C). We also evaluated the biodistribution and degradation of PEG-EGCG/HTC@FITC compared with the EGCG@FITC by ex vivo imaging (Figure 10D). In the EGCG@FITC group, EGCG was rapidly metabolized through the kidneys after injection, with a limited distribution in the liver. By the end of 4th hour, there was almost no EGCG@FITC in the liver. However, in the nanomicelle group, PEG-EGCG/HTC@FITC presented a strong and rapid accumulation in the liver, and the drug content in the liver was much higher than that in the EGCG@FITC group. Moreover, the nanomicelle group also showed a sustained release effect, and PEG-EGCG/HTC@FITC in the liver remained at a high level after 4 h of administration, indicating an excellent liver-targeting property and sustained release pattern of PEG-EGCG/HTC.

**Figure 11:**
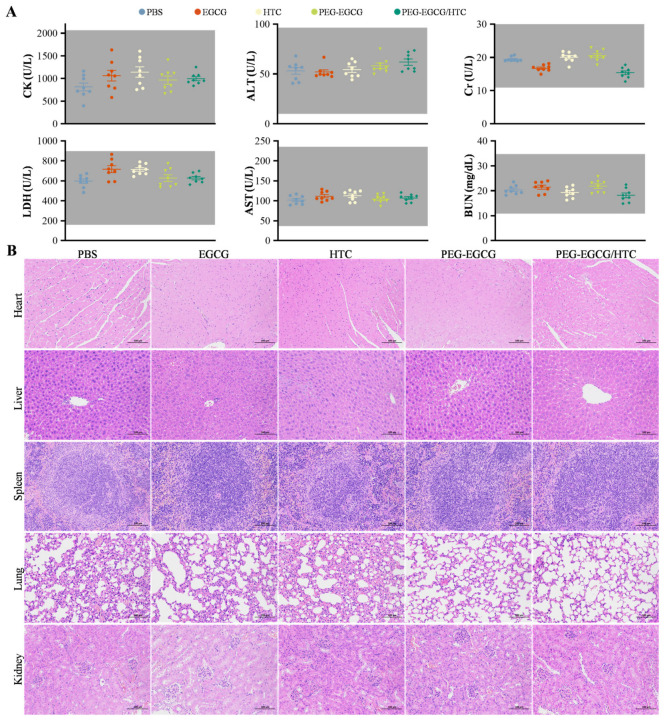
Safety profile of PEG-EGCG/HTC nanomicelles. (**A**) Blood biochemistry analysis assessing cardiac, hepatic, and renal function (gray shaded areas in the figures indicate the normal reference ranges for mice) (*n* = 8, mean ± SD). (**B**) Histopathological examination of the vital organs (heart, liver, spleen, lungs, kidneys) (Scale bar = 100 μm).

After one week of daily tail-vein injections of EGCG, HTC, PEG-EGCG, or PEG-EGCG/HTC, blood biochemical parameters (including CK, ALT, Cr, LDH, AST, and BUN) did not show any significant changes in intervention groups compared with the negative control group (PBS group), with all parameters falling within the normal ranges (Figure 11A). Histopathological examination (Figure 11B) revealed no apparent abnormalities in the vital organs (heart, liver, spleen, lungs, kidneys). These findings confirm that neither EGCG nor HTC exerts obvious toxic effects, and demonstrate that both PEG-EGCG and the final product PEG-EGCG/HTC exhibit favorable safety profiles for subsequent in vivo studies.

### 2.8. Therapeutic Efficacy of PEG-EGCG/HTC on Non-Lethal Jellyfish Envenomation

**Figure 12:**
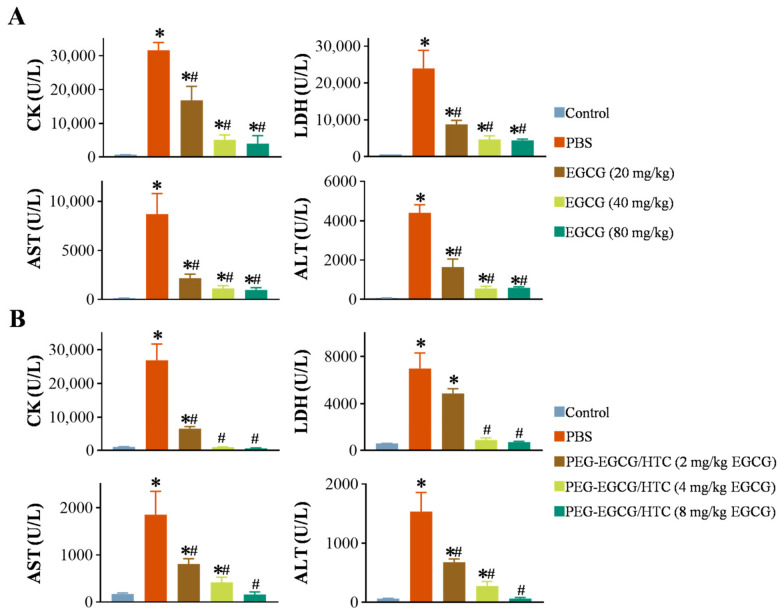
Dose–response evaluation of EGCG and PEG-EGCG/HTC on non-lethal jellyfish envenomation in mice. (**A**) Effects of different doses of EGCG on blood biochemical parameters in mice with non-lethal envenomation by NnV. (**B**) Effects of different doses of PEG-EGCG/HTC on blood biochemical parameters in mice with non-lethal envenomation by NnV. (*n* = 6, mean ± SD. * *p* < 0.05 versus Control group, # *p* < 0.05 versus PBS group).

Different doses of EGCG or PEG-EGCG/HTC were administered via tail vein injection as a therapeutic intervention 1 h after venom exposure. Mouse serum was collected 10 h post-envenomation for blood biochemical analysis. The results in Figure 12A indicated that EGCG exhibited therapeutic efficacy in a mouse model of non-lethal envenomation by NnV, with a dose–response relationship within the range of 20 to 40 mg/kg. However, increasing the EGCG dose from 40 mg/kg to 80 mg/kg did not significantly improve therapeutic outcomes. Notably, cardiac and hepatic functional damage persisted in the envenomed mice of all dose groups.

Instead, the results in Figure 12B demonstrated that PEG-EGCG/HTC was a more effective alternative to EGCG in the same model. When administered at a dose containing 8 mg/kg of EGCG, the PEG-EGCG/HTC nanomicelles restored all the blood biochemical parameters in envenomed mice to normal levels.

**Figure 13:**
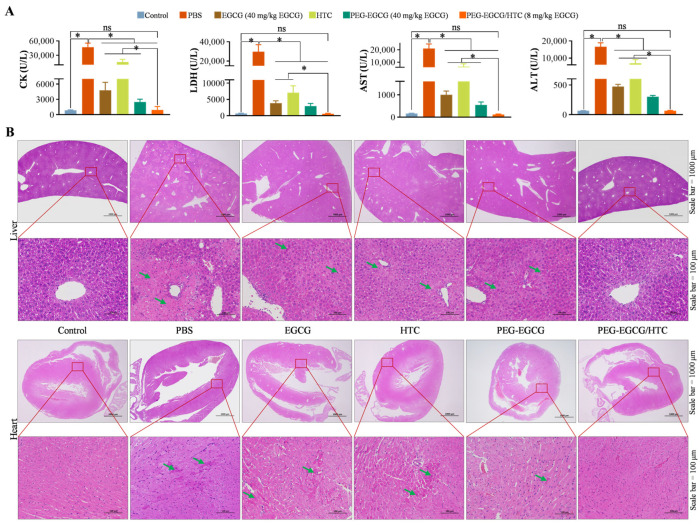
Therapeutic effects of EGCG, HTC, PEG-EGCG and PEG-EGCG/HTC on non-lethal jellyfish envenomation in mice. (**A**) Effects of different treatments on blood biochemical parameters in NnV-envenomed mice (*n* = 6, mean ± SD. * *p* < 0.05, ns, no significant difference). (**B**) Histopathological findings in NnV-envenomed mice following different treatments. Green arrows indicated pathological alterations.

Finally, a comparative analysis was conducted on the detoxification efficacy of EGCG (40 mg/kg), HTC (3.1 mg/kg), PEG-EGCG (containing 40 mg/kg EGCG), and PEG-EGCG/HTC (containing 8 mg/kg EGCG and 3.1 mg/kg HTC). The blood biochemical test results (Figure 13A) indicated that all four different treatments improved the indicators of cardiac and hepatic dysfunction in envenomed mice compared to the PBS control. Among them, the HTC group showed the weakest therapeutic effect, while the PEG-EGCG/HTC group exhibited the most potent therapeutic effect.

Histopathological findings were largely consistent with the blood biochemical analysis (Figure 13B). In the EGCG, HTC, and PEG-EGCG intervention groups, although there was a marked improvement compared to the PBS group, minor pathological changes were still observable, such as small hepatic hemorrhages, slight destruction of hepatic lobule structure, and focal cardiomyocyte eosinophilia with nuclear condensation. However, no obvious histopathological abnormalities were detected in the heart or liver of the mice treated with PEG-EGCG/HTC.

### 2.9. Preventive Effect of PEG-EGCG/HTC on Non-Lethal Jellyfish Envenomation

**Figure 14:**
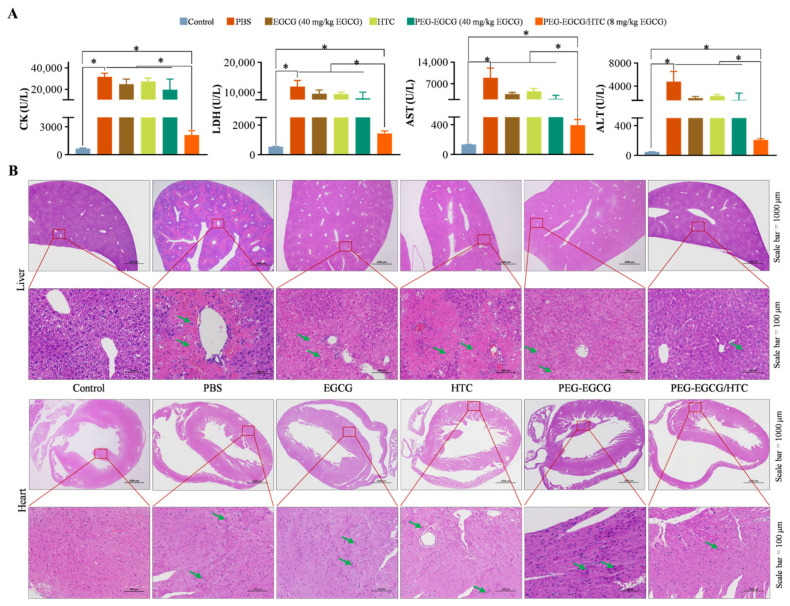
Preventive effects of EGCG, HTC, PEG-EGCG and PEG-EGCG/HTC on non-lethal jellyfish envenomation in mice. (**A**) Effects of different preventive interventions on blood biochemical parameters in mice with non-lethal jellyfish envenomation (*n* = 6, mean ± SD. * *p* < 0.05). (**B**) Histopathological analysis of the heart and liver tissues from NnV-envenomed mice following different preventive interventions. Green arrows indicated pathological alterations.

To investigate the preventive efficacy, mice were injected with EGCG, HTC, PEG-EGCG or PEG-EGCG/HTC via the tail vein 30 min prior to envenomation. The blood biochemical test results indicated that preventive administration of EGCG, HTC or PEG-EGCG failed to ameliorate NnV-induced cardiac dysfunction and pathological alterations in mice, except for providing only partial protection to the liver (Figure 14A). In contrast, preventive administration of PEG-EGCG/HTC significantly attenuated NnV-induced functional impairment and pathological changes in both cardiac and hepatic tissues (Figure 14B).

To sum up, PEG-EGCG/HTC nanomicelles were successfully developed. DLS analysis confirmed that the nanomicelles possessed an appropriate particle size, good dispersibility and favorable stability in vitro. The results of the in vivo toxicity study revealed that EGCG, HTC, PEG-EGCG and PEG-EGCG/HTC, administered separately via short-term injection, did not induce abnormalities in blood biochemical indexes (reflecting cardiac, hepatic and renal function) or pathological changes in the vital organs (heart, liver, spleen, lungs and kidneys). These findings indicate that PEG-EGCG/HTC has no detectable toxic effects and is suitable for in vivo application. Furthermore, the therapeutic and preventive potential of PEG-EGCG/HTC was comprehensively evaluated. In the therapeutic regimen, PEG-EGCG/HTC exhibited superior efficacy compared to EGCG, HTC or PEG-EGCG. EGCG (40 mg/kg) and PEG-EGCG (containing 40 mg/kg EGCG) only partially improved the abnormal blood biochemical indexes and pathological damage in NnV-envenomed mice, while PEG-EGCG/HTC containing only 8 mg/kg EGCG completely reversed the toxic effects of NnV. In addition, preventive administration of EGCG or PEG-EGCG did not show obvious antitoxic effects against NnV, whereas preventive administration of PEG-EGCG/HTC significantly improved the functional damage and pathological changes in the heart and liver in the mice envenomed by NnV.

## 3. Conclusions

In this study, we first demonstrated that therapeutic intervention with EGCG attenuated NnV-induced oxidative stress and inflammatory injury, and suppressed apoptosis at both the cellular and animal levels, elucidating the protective mechanism of EGCG. Subsequently, we designed and synthesized PEG-EGCG/HTC nanomicelles, which successfully integrated the enzyme inhibitory effect of HTC and antioxidant properties of EGCG. Notably, this nano-drug delivery system exhibited an optimal particle size, excellent dispersibility, good in vitro stability, and favorable safety profile; moreover, it demonstrated significant protective effects against functional damage and pathological alterations in non-lethal NnV-envenomed mice. However, the antitoxic effect of PEG-EGCG/HTC nanomicelles still needs further validation in other toxic jellyfish species.

## 4. Materials and Methods

### 4.1. Reagents and Jellyfish Collection

Methoxy-poly (ethylene glycol) with a thiol terminal (PEG-SH, molecular mass = 2000 Da) and HTC were purchased from Ruixi Biological Technology Co., Ltd. (Xi’an, China). EGCG (>95% purity) was purchased from Shanghai Macklin Biochemical Co., Ltd. (Shanghai, China). Mouse IL-1β ELISA kit and mouse IL-6 ELISA kit were obtained from Shanghai Beyotime Biotechnology Co., Ltd. (Shanghai, China). Dihydroethidine (DHE) and 2,7-dichlorofluorescein diacetate (DCFH-DA) were purchased from Shanghai Aladdin Biochemical Technology Co., Ltd. (Shanghai, China).

Specimens of the jellyfish *Nemopilema nomurai* were collected from the Laoshan Bay in Qingdao, China, in August 2023. The tentacles were excised and quickly transported to a laboratory in Shanghai with dry ice, then stored in an ultra-low temperature freezer at −80 °C for subsequent experiments.

### 4.2. Nematocyst Isolation

Nematocysts were isolated from the tentacles of *N. nomurai* [43] using a slightly modified version of the method described by Wang et al. Frozen tentacles were immersed in artificial seawater at 4 °C and incubated for 4 days. The mixture was filtered through a 200-mesh sieve to remove tissue debris, and the filtrate was centrifuged at 500× *g* for 3 min at 4 °C. The resulting precipitate was collected and washed three times with artificial seawater, then further purified by Percoll density gradient centrifugation. The bottom precipitate was collected and rinsed to obtain venom-containing nematocysts. The prepared nematocysts were either used immediately for venom extraction or stored at −80 °C for subsequent experiments.

### 4.3. Venom Extraction

With slight modifications to the method established by Li et al., nematocyst venom was extracted via ultrasonication [44]. The nematocysts were resuspended in deionized water and fully ruptured using a Misonix S-4000-010 ultrasonic cell disruptor (Misonix, Inc., Farmingdale, NY, USA) at an output power of 400 W. The mixture was centrifuged at 12,000× *g* for 15 min at 4 °C, and the supernatant was collected as *Nemopilema nomurai* venom. The venom was dialyzed against 0.01 mol/L phosphate buffer solution (pH 7.4) using a 3000 Da dialysis bag for 8 h before use. This venom was designated as NnV, and its protein concentration was determined by the Bradford assay.

### 4.4. Animals and Cell Lines

Male ICR mice (20 ± 2 g) were provided by the Laboratory Animal Center of Naval Medical University. All mice were kept at constant temperature with regular circadian rhythm and acclimatized for one week prior to experiments. This study was carried out in strict accordance with the guidelines of the Animal Ethics Committee of Naval Medical University. Rat cardiomyocyte (H9C2) cells, obtained from the American Type Culture Collection (ATCC) (Manassas, VA, USA), were cultured in DMEM supplemented with 10% FBS and 1% penicillin-streptomycin at 37 °C in a 5% CO_2_ humidified incubator.

### 4.5. In Vitro Experiments

#### 4.5.1. Cell Treatments

The H9C2 cells were incubated at a density of 7 × 10^4^ cells/well in 6-well plates for 24 h in 2.5 mL of complete culture medium and divided into the following four groups: (1) PBS group (negative control group); (2) NnV group: 260 ng/mL NnV; (3) NnV + EGCG group: 20 μM EGCG was added after 260 ng/mL NnV treatment for 30 min; (4) EGCG group: 20 μM EGCG. The different reagents were added to the 6-well plate at the corresponding time points, and then incubated for 4 h.

#### 4.5.2. Detection of Intracellular ROS

After H9C2 cells were treated according to Section 4.5.1, the fluorescent probes of DCFH-DA and DHE (Beyotime, Shanghai, China) were dissolved in serum-free medium with a stock solution of 5 μM and 10 μM, respectively. Then, the diluted DCFH-DA or DHE was added to each well. The intracellular ROS was observed by a fluorescence microscope. All assays were independently repeated at least three times.

#### 4.5.3. Protein Expression Analysis by Western Blot In Vitro

Cells were lysed using RIPA lysis buffer (containing 2% protease inhibitor mixture and 2% phosphatase inhibitor mixture; Beyotime, Shanghai, China) to extract the total proteins. Next, the proteins were fractionated and electrotransferred onto polyvinylidene difluoride (PVDF) membranes. The separated proteins were subsequently probed with primary antibodies followed by incubation with their corresponding secondary antibodies. Protein bands were visualized and quantified using ImageJ software 1.53c (National Institutes of Health, Bethesda, MD, USA). Primary antibodies against Nrf2, CAT, β-Actin, HO-1, GPX4, cleaved PARP, Bcl-xl, Bax, cleaved Caspase-3 were purchased from Cell Signaling Technology Inc. (Danvers, MA, USA). All assays were independently repeated at least three times. (Detailed procedures are provided in the Supplementary Materials.)

#### 4.5.4. Apoptosis Detection by Fluorescence

After their respective treatments, the cells in 6-well plates were stained with 5 μL of Annexin V-FITC, 2 μL of Mito-Tracker Red CMXROs and 5 μL of Hoechst 33342 (Beyotime, Shanghai, China), followed by incubation at room temperature in the dark for 30 min. Fluorescence images were acquired using a fluorescence microscope. All assays were independently repeated at least three times.

### 4.6. In Vivo Experiments

#### 4.6.1. Experimental Protocols

Male ICR mice were randomly divided into four groups as follows: (1) PBS (negative control), (2) NnV, (3) EGCG intervention group (NnV + EGCG), and (4) EGCG.

Therapeutic protocol: Mice in groups 2 and 3 were injected via the tail vein with 0.8 × LD_50_ NnV, groups 1 and 4 were injected with PBS. One hour later, groups 3 and 4 were given 20 mg/kg·EGCG intravenously, and groups 1 and 2 were injected with PBS (detailed in Section 4.11.1).

Preventive protocol: A separate cohort of mice adopted the same grouping scheme. Groups 3 and 4 were pretreated with 20 mg/kg EGCG i.v. 30 min before NnV; groups 1 and 2 were injected with PBS. All except group 4 were then injected with 0.8 × LD_50_ NnV (detailed in Section 4.12).

For both protocols, all mice were anesthetized before cervical dislocation, and the heart and liver tissues were harvested 10 h after the first injection for further study.

#### 4.6.2. Protein Expression Analysis by Western Blot In Vivo

The heart and liver tissues were crushed by a homogenizer and lysed using RIPA lysis buffer (containing 2% protease inhibitor mixture and 2% phosphatase inhibitor mixture). Protein expression analysis in the heart and liver tissues was also conducted by Western blot. The protocols have been described previously in Section 4.5.3.

#### 4.6.3. Protein Distribution and Expression Analysis by Immunohistochemistry

To further clarify the changes in expression and distribution of proteins in the heart and liver tissues, we performed immunohistochemical staining. Using the heart and liver samples embedded in paraffin, we evaluated the following proteins: Nrf2, CAT, HO-1, GPX4, cleaved PARP, Bcl-xl, Bax, cleaved Caspase-3, IL-1β, and IL-6. Their distribution and expression were detected under a light microscope. Detailed procedures are provided in the Supplementary Materials.

#### 4.6.4. Detection of Serum Inflammatory Factors by Enzyme-Linked Immunosorbent Assay (ELISA)

Blood samples were collected from the retro-orbital venous plexus of the mice. The collected blood samples were stored for 2 h at 4 °C and then were centrifuged at 2000× *g* for 15 min. The clear supernatants were collected and used for ELISA analysis, as per the kit protocol (Beyotime, Shanghai, China). *n* = 6.

### 4.7. Synthesis and Characterization of PEG-EGCG/HTC

#### 4.7.1. Synthesis of PEG-EGCG

PEG-EGCG was synthesized by nucleophilic addition reaction: EGCG (40 mg) was dissolved in 40 mL of a 1:1 (*v*/*v*) mixture of PBS and dimethyl sulfoxide (DMSO). PEG-SH (80 mg) was dissolved in 40 mL of PBS. The two solutions were mixed and stirred at 25 °C (pH 8.4) for 24 h. The reaction was terminated by adding 10% acetic acid (pH 4.0), and the obtained solution was dialyzed (MWCO = 2000 Da) against PBS and freeze-dried to obtain PEG-EGCG [26].

#### 4.7.2. ^1^H NMR Analysis of the Interactions Between PEG-SH and EGCG

Three milligrams of PEG-SH, EGCG and PEG-EGCG were dissolved in deuterated water, respectively. ^1^H NMR spectra were analyzed using a Bruker Ultrashield Plus NMR spectrometer (Bruker Biospin Corporation, Billerica, MA, USA) operating at 400 MHz.

#### 4.7.3. Evaluation of Antioxidant Properties of PEG-EGCG

The total antioxidant capacity of EGCG and PEG-EGCG (with equivalent EGCG) was evaluated using the DPPH, ABTS and FRAP assays, according to established protocols (Beyotime, Shanghai, China). *n* = 3. Detailed procedures are provided in the Supplementary Materials.

#### 4.7.4. Fabrication of PEG-EGCG/HTC

To fabricate PEG-EGCG/HTC, HTC and PEG-EGCG were dissolved in chloroform-methanol mixed solution (0.5:1 *v*/*v*) at a weight ratio of 1/10, and then chloroform and methanol were evaporated under reduced pressure. The resulting film of PEG-EGCG and HTC mixture was hydrated by adding deionized water and incubating under a nitrogen atmosphere at 37 °C for 24 h. Finally, the resulting PEG-EGCG/HTC drug-loaded micelles were obtained after 2 min ultrasound.

#### 4.7.5. Characterization of PEG-EGCG/HTC

The particle size, polydispersity index (PDI) and surface charge of PEG-EGCG/HTC were measured by dynamic light scattering (DLS) technology at 25 °C. The morphology and structure of PEG-EGCG/HTC were observed by a transmission electron microscope.

#### 4.7.6. Drug Loading and Encapsulation Efficiency of PEG-EGCG/HTC

PEG-EGCG, HTC and PEG-EGCG/HTC were dissolved in deionized water, respectively. The absorbance of each solution was measured across the wavelength range of 200~700 nm, using deionized water as the blank control. *n* = 3.

### 4.8. In Vitro Release Study

A 2 mL aliquot of PEG-EGCG/HTC (0.5 mg/mL, diluted in PBS containing 10% mouse serum) was placed in a dialysis bag (MWCO = 1000 Da). The bag was then immersed in 20 mL of PBS containing 10% mouse serum, and the release of HTC was monitored in an incubator shaker at 37 °C. At designated time-points (5, 10, 20, 40, 80, 160, 320 and 640 min), the absorbance of the outside dialysate was measured at 356 nm. The concentrations of released HTC in the samples were calculated based on an HTC standard curve generated from known concentrations of HTC in PBS containing 10% mouse serum at 37 °C.

### 4.9. Metabolism and Distribution of PEG-EGCG/HTC In Vivo

ICR mice were injected via tail vein with EGCG, PEG-EGCG or PEG-EGCG/HTC (all groups received an equivalent dose of EGCG). Blood samples were collected at 0.5, 1, 2, 4 and 6 h after administration, and centrifuged at 2000*× g* for 15 min at 4 °C to obtain serum. Heart and liver tissues were collected at 1 h and 4 h after administration. Detailed protocols for sample pretreatment are available in the Supplementary Materials. Serum and tissue samples were subsequently analyzed by ultra-performance liquid chromatography-tandem mass spectrometry (UPLC-MS/MS). For in vitro fluorescence imaging, EGCG and PEG-EGCG/HTC were labeled with FITC via covalent interaction. EGCG (5 mg/mL in DMSO) and FITC (10 mM in DMSO) were mixed at a molar ratio of 1:10. The pH was maintained at 8.5–9.0 using 0.1 M carbonate buffer, and the mixture reacted at 4 °C for 4 h with stirring in the dark. After dialysis and lyophilization, EGCG@FITC was obtained. PEG-EGCG/HTC@FITC was then synthesized from EGCG@FITC following the above protocol for subsequent assays. For in vivo fluorescence imaging, mice received tail vein injections of EGCG@FITC or PEG-EGCG/HTC@FITC (with an equivalent dose of EGCG). Major organs were collected at 0.5, 1, 2, and 4 h, respectively after different treatments, and fluorescent images were captured using an in vivo imaging system (IVIS Lumina III, PerkinElmer, Waltham, MA, USA). *n* = 6.

### 4.10. Safety Evaluation of PEG-EGCG/HTC In Vivo

ICR mice were randomly divided into five groups, and received daily tail-vein injections of PBS, EGCG, HTC or PEG-EGCG/HTC for one week. Blood samples were collected 10 h after the final injection (*n* = 8). At the same time, the vital organs such as heart, liver, spleen, lungs and kidneys were quickly dissected and immediately fixed in 4% paraformaldehyde for further histopathological analysis.

### 4.11. Therapeutic Effect of PEG-EGCG/HTC on Non-Lethal Jellyfish Envenomation

#### 4.11.1. Exploration of Therapeutic Dosage

Male ICR mice were randomly grouped and injected via tail vein with 0.8 × LD_50_ dose of NnV to establish a non-lethal jellyfish envenoming model. After 1 h, different doses of EGCG or PEG-EGCG/HTC were administered intravenously. All mice were observed for 10 h, after which blood samples were collected for biochemical analysis of cardiac and hepatic function markers. *n* = 6.

#### 4.11.2. Therapeutic Detoxification Effect of PEG-EGCG/HTC

After 1 h of NnV exposure, the envenomed mice were injected with PBS, 40 mg/kg EGCG, 3.1 mg/kg HTC, PEG-EGCG (containing 40 mg/kg EGCG) and PEG-EGCG/HTC (containing 8 mg/kg EGCG and 3.1 mg/kg HTC), respectively. After a total exposure period of 10 h, blood samples were collected to examine blood biochemical indexes, and the heart and liver were dissected for further histopathological evaluation by hematoxylin and eosin (H&E) staining. *n* = 6.

### 4.12. Experimental Methods for Evaluating the Preventive Effect of PEG-EGCG/HTC on Non-Lethal Jellyfish Envenomation

Male ICR mice were randomly grouped and pretreated with PBS, 40 mg/kg EGCG, 3.1 mg/kg HTC, PEG-EGCG (containing 40 mg/kg EGCG) or PEG-EGCG/HTC (containing 8 mg/kg EGCG and 3.1 mg/kg HTC), respectively. After 30 min, all mice were injected with 0.8 × LD_50_ dose of NnV. After 10 h of exposure, blood samples and heart and liver tissues were collected for further analysis. *n* = 6.

### 4.13. Statistical Analysis

In the experiments, all values in the figures and text are expressed as the mean ± SD. The Shapiro–Wilk test was used to assess data normality, and Levene’s test for variance homogeneity. One-way ANOVA was adopted for multiple-group comparisons with normal distribution and equal variance, followed by Tukey’s post hoc test for pairwise comparisons. Otherwise, the Kruskal-Wallis nonparametric test was performed, with Dunn’s test used for post hoc analysis. A *p* value < 0.05 was considered statistically significant.

## Figures and Tables

**Table 1:** Structural characteristics of PEG-EGCG/HTC nanomicelles.

Characterization	Parameter Value
hydrodynamic size (nm)	147.0 ± 3.78
polydispersity index (PDI)	0.23 ± 0.01
zeta potential (mV)	−38.62 ± 1.86
loading content (wt%)	6.73
loading efficiency (%)	84.26

(*n* = 3, mean ± SD).

## Data Availability

The original contributions presented in this study are included in the article/Supplementary Material. Further inquiries can be directed to the corresponding authors.

## Supplementary Materials

The following supporting information can be downloaded at: https://www.mdpi.com/article/10.3390/toxins18070278/s1, Figure S1: Uncropped original full-size cardiac tissue immunostaining images corresponding to Figure 3C; Figure S2: Uncropped original full-size hepatic tissue immunostaining images corresponding to Figure 4C; Figure S3: Uncropped original full-size cardiac tissue immunostaining images corresponding to Figure 5D; Figure S4: Uncropped original full-size cardiac tissue immunostaining images corresponding to Figure 6C; Figure S5: Uncropped original full-size cardiac tissue immunostaining images corresponding to Figure 7C; Figure S6: Uncropped full-size hepatic tissue immunostaining images corresponding to Figure 8C; Figure S7: Original full-size histopathological images of vital organs (heart, liver, spleen, lung, kidney) corresponding to Figure 11B; Figure S8: Uncropped full-size histopathological micrographs of heart tissues corresponding to Figure 13B; Figure S9: Uncropped full-size histopathological micrographs of liver tissues corresponding to Figure 13B; Figure S10: Uncropped full-size histopathological micrographs of heart tissues corresponding to Figure 14B; Figure S11: Uncropped full-size histopathological micrographs of liver tissues corresponding to Figure 14B; File S1: full protocols of experiments.
