# Supplementary material for: Enhanced Protection Against Toxicity of Nemopilema nomurai Venom Using a PEG-EGCG/Tetracycline Hydrochloride Micellar Nanocomplex
Source: Toxins (Basel). 2026 Jun 24;18(7):278. doi: 10.3390/toxins18070278 (PMC13417419; doi:10.3390/toxins18070278)
Supplement: Supplementary file 1 [file toxins-18-00278-s001.zip › File S1. full protocols of experiments.pdf]

Supplementary Materials: Enhanced Protection Against Toxicity of *Nemopilema nomurai* Venom Using a PEG-EGCG/Tetracycline Hydrochloride Micellar Nanocomplex

Jie Li [1,†], Yanan Hu [1,†], Yunfeng Qian [2,†], Sai Luo [1], Juxingsi Song [1], Shaoqian Zhu [1], Minglei Wang [1], Huiliang Gan [1,*], Qianqian Wang [1,*] and Liming Zhang [1,*]

[1] Naval Special Medical Center, Naval Medical University, Shanghai 200433, China; lijie1992@smmu.edu.cn (J.L.); yananhu20192022@163.com (Y.H.); m15056344020@163.com (S.L.); song9935@163.com (J.S.); shaoqianzhu2018@163.com (S.Z.); wml22134190@zuaa.zju.edu.cn (M.W.)

[2] The Third Affiliated Hospital, Naval Medical University, Shanghai 200433, China; money0814@163.com (Y.Q.)

* Correspondence: hjyx1823@163.com (H.G.); abc_w@smmu.edu.cn (Q.W.); lmzhang@smmu.edu.cn (L.Z.)

† These authors contributed equally to this work.

1. Protein expression analysis by Western blot

Protein samples were mixed with 5× protein loading buffer and denatured at 100 °C for 5 min. Electrophoresis was performed on precast 15% gels: 80 V for 15 min (stacking), then 120 V until the dye front reached the gel bottom. For transfer, 0.2 μm PVDF membranes were activated with methanol; electrotransfer was carried out at 200 mA for 90 min under low temperature. After blocking, membranes were washed three times with TBST (10 min each). Membranes were incubated with primary antibodies (1:1000 dilution) at 4 °C for 6-18 h, then rewashed with TBST. Secondary antibodies (1:1500 dilution) were applied for 2 h at room temperature with shaking. Finally, signals were detected using ECL substrate after TBST washing.

2. Immunohistochemistry

Paraffin-embedded sections were dewaxed and rehydrated, followed by microwave antigen retrieval in citrate buffer (pH 6.0). Endogenous peroxidase activity was blocked with 3% H_2O_2 for 25 min at room temperature in the dark. Non-specific binding sites were blocked with 3% BSA for 30 min at room temperature. The sections were incubated with primary antibody overnight at 4 °C, and subsequently with secondary antibody for 50 min at room temperature. DAB staining was monitored microscopically until brown positive signals were visible. Following hematoxylin counterstaining, sections were dehydrated, cleared and mounted for microscopic observation.

3. Evaluation of antioxidant properties of PEG-EGCG

Antioxidant activities were determined using Beyotime kits for DPPH, ABTS and FRAP assays. EGCG and PEG-EGCG were prepared at serial concentrations with equivalent EGCG content, and all procedures followed kit protocols. For DPPH assay, reactions proceeded at room temperature in the dark; absorbance was read at 515 nm to calculate radical scavenging rate. For ABTS assay, absorbance was measured at 734 nm after incubation to assess radical scavenging activity. For FRAP assay, a ferrous sulfate standard curve was generated. Samples were incubated at 37 °C, and absorbance at 593 nm was detected to determine total antioxidant capacity.

4. Metabolism and distribution of PEG-ECGC/HTC *in vivo*

Sample pretreatment

For solid tissues (approximately 0.1 g of heart and liver tissues), specimens were homogenized with 5 mL of 70% methanol, vortexed, and ultrasonically extracted for 15 min. The extraction was repeated once. The combined supernatants were centrifuged at 6000 × *g* for 10 min and filtered through 0.22 μm membranes. For serum (0.2 mL), it was

mixed with 0.8 mL methanol, vortexed, centrifuged at 6000 × *g* for 10 min, and the supernatant was filtered.

UPLC-MS/MS analysis

The analysis was performed on an Agilent 1290 II UPLC system coupled to an Agilent 6470 triple quadrupole tandem mass spectrometer. Chromatographic separation was achieved on an Agilent C18 column (2.1 mm× 100 mm, 1.8 μm) at a flow rate of 0.2 mL/min. The mobile phase consisted of solvent A (0.1% formic acid in water, v/v) and solvent B (acetonitrile). The column was equilibrated with 95% A and 5% B. The proportion of A was gradually reduced to 5% within 2 min, maintained for 2 min, and then returned to the initial ratio. EGCG was quantified in positive ion mode using multiple reaction monitoring (MRM). The mass spectrometric parameters were set as follows: gas temperature 280 °C, nebulizer gas flow 7 L/min, nebulizer pressure 40 psi, desolvation temperature 350 °C, drying gas flow 11 L/min, and capillary voltage 4000 V. The MRM transitions of *m/z* 459.1 → 138.9 and *m/z* 459.1 → 150.9 were used for quantification and qualification, respectively, at a collision energy of 90 eV.
