# Supplementary figures and images for: Enhanced Protection Against Toxicity of Nemopilema nomurai Venom Using a PEG-EGCG/Tetracycline Hydrochloride Micellar Nanocomplex (part 2 of 2)
Source: Toxins (Basel). 2026 Jun 24;18(7):278. doi: 10.3390/toxins18070278 (PMC13417419; doi:10.3390/toxins18070278)

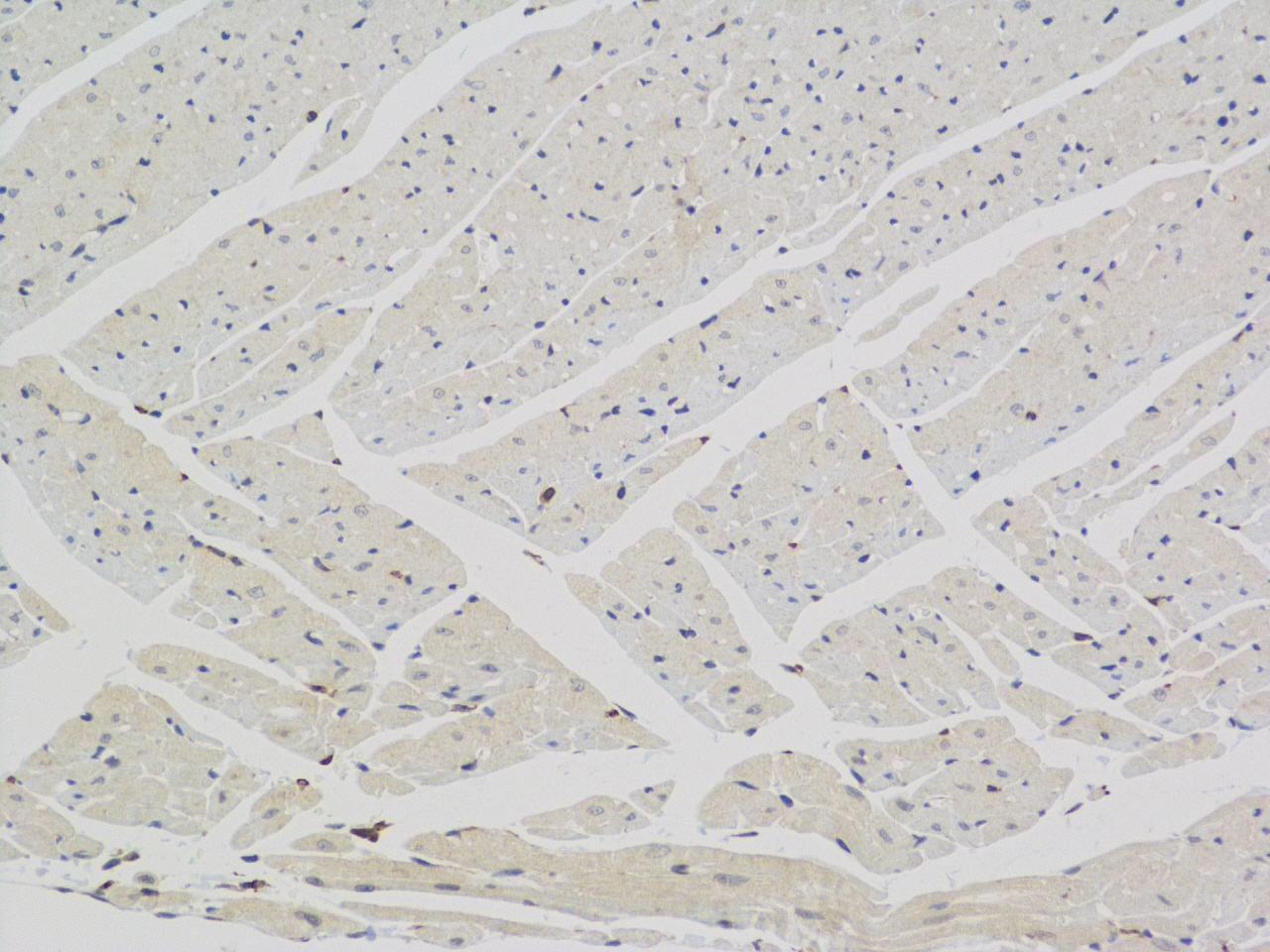

Supplement: Supplementary file 1 [file toxins-18-00278-s001.zip › Figure S1. Uncropped original full-size cardiac tissue immunostaining images corresponding to Figure 3C/PBS-Nrf2.jpg]

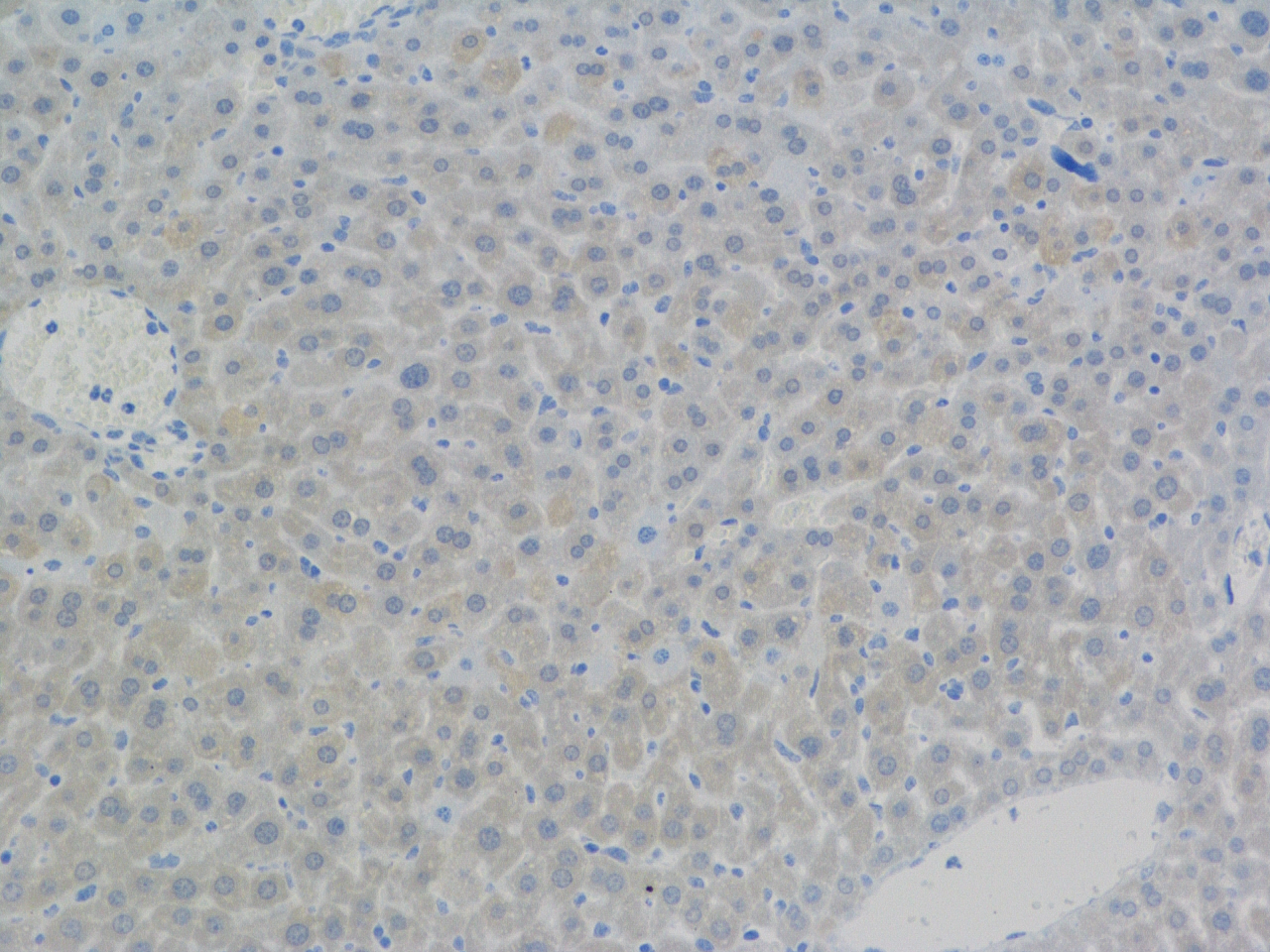

Supplement: Supplementary file 1 [file toxins-18-00278-s001.zip › Figure S2. Uncropped original full-size hepatic tissue immunostaining images corresponding to Figure 4C/EGCG-CAT.jpg]

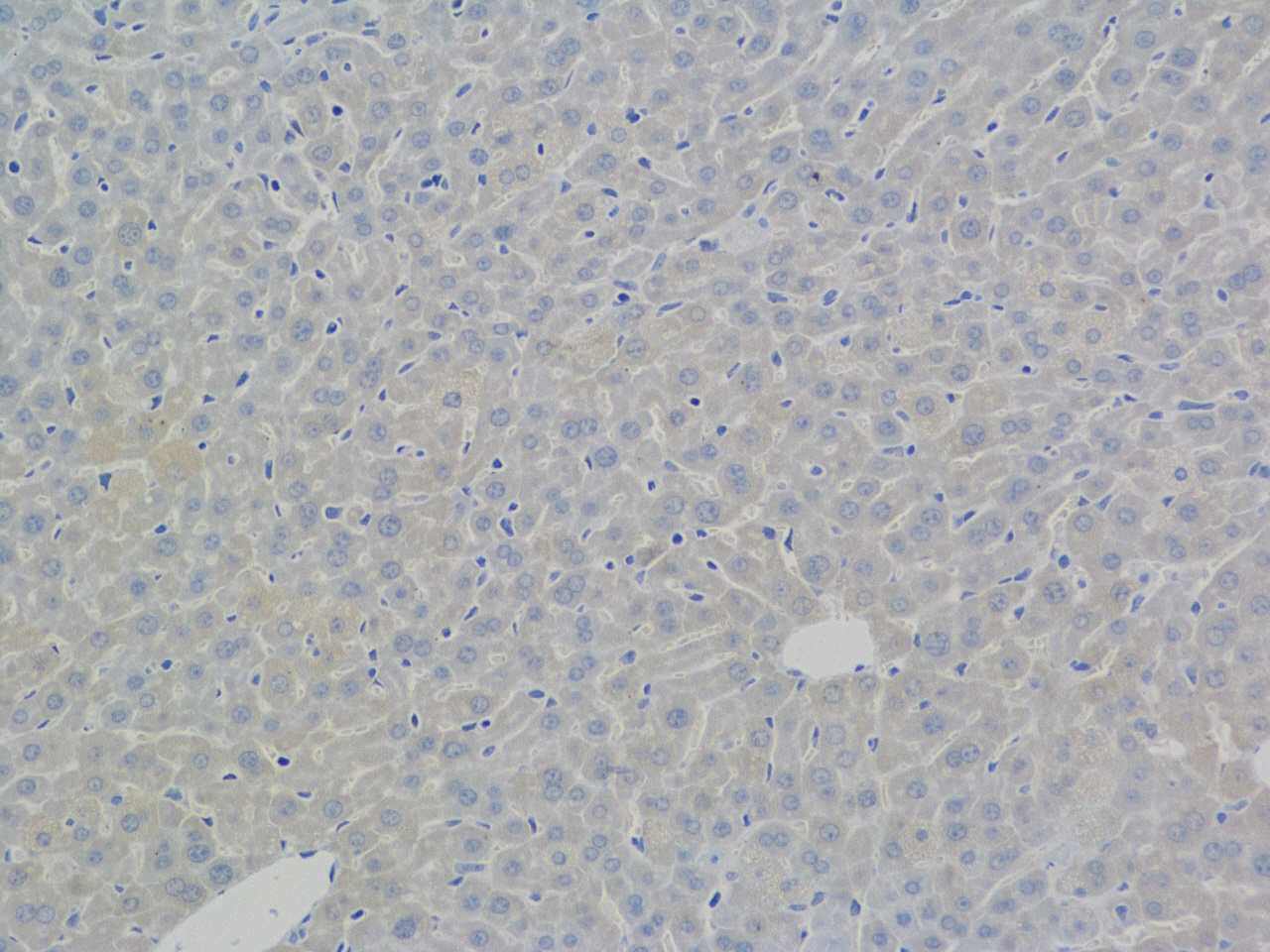

Supplement: Supplementary file 1 [file toxins-18-00278-s001.zip › Figure S2. Uncropped original full-size hepatic tissue immunostaining images corresponding to Figure 4C/EGCG-GPX4.jpg]

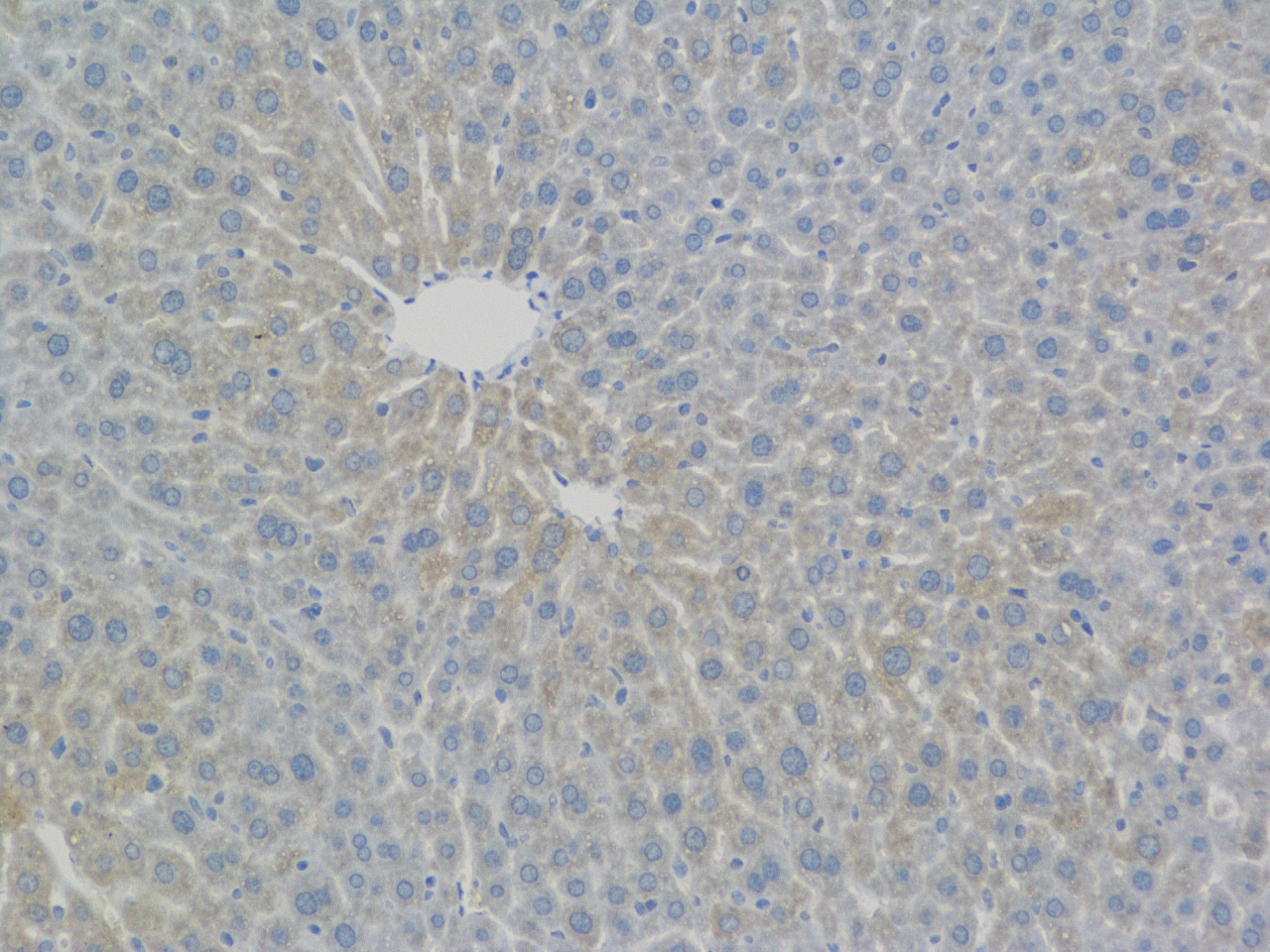

Supplement: Supplementary file 1 [file toxins-18-00278-s001.zip › Figure S2. Uncropped original full-size hepatic tissue immunostaining images corresponding to Figure 4C/EGCG-HO-1.jpg]

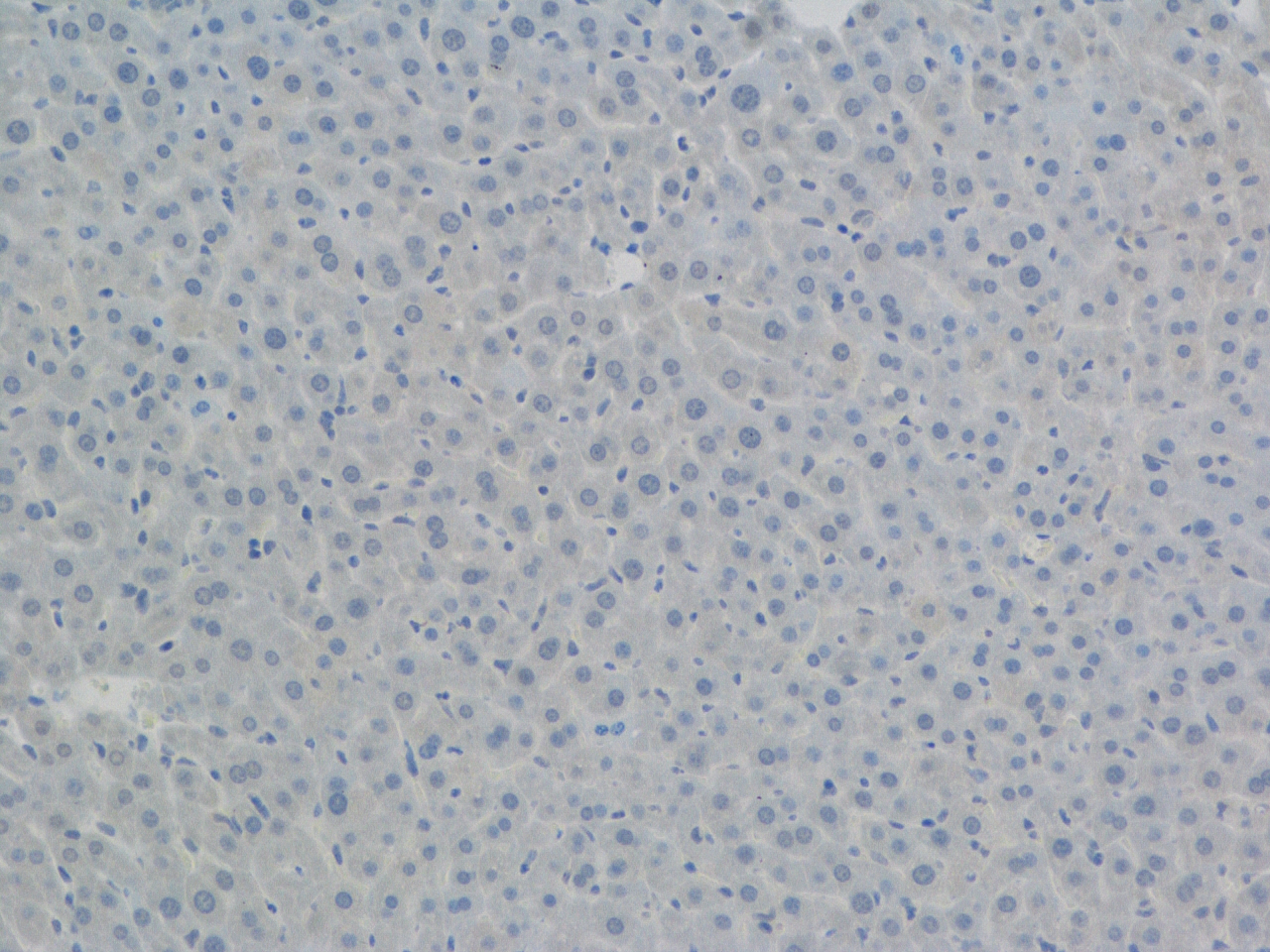

Supplement: Supplementary file 1 [file toxins-18-00278-s001.zip › Figure S2. Uncropped original full-size hepatic tissue immunostaining images corresponding to Figure 4C/EGCG-Nrf-2.jpg]

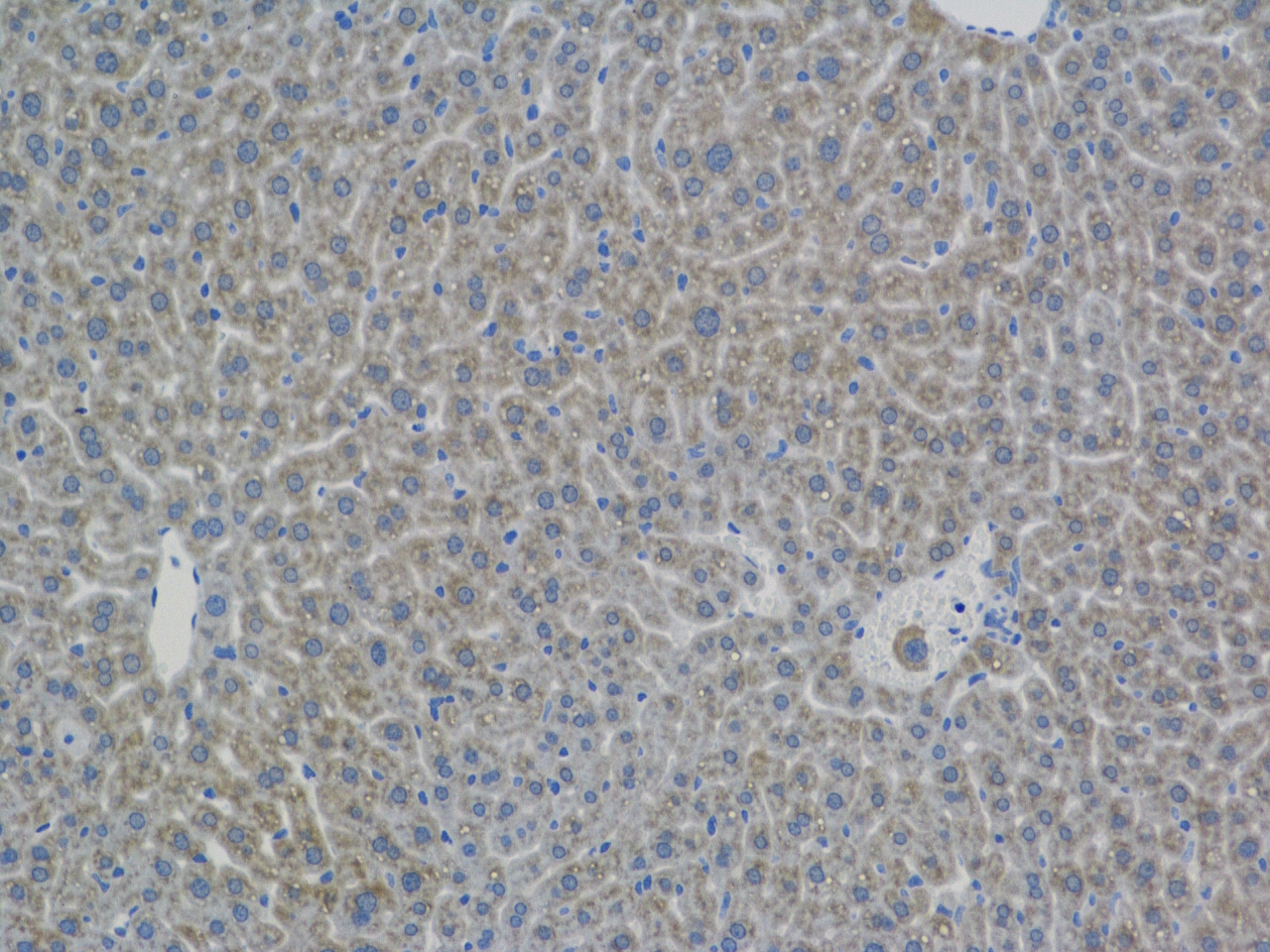

Supplement: Supplementary file 1 [file toxins-18-00278-s001.zip › Figure S2. Uncropped original full-size hepatic tissue immunostaining images corresponding to Figure 4C/NnV+EGCG-CAT.jpg]

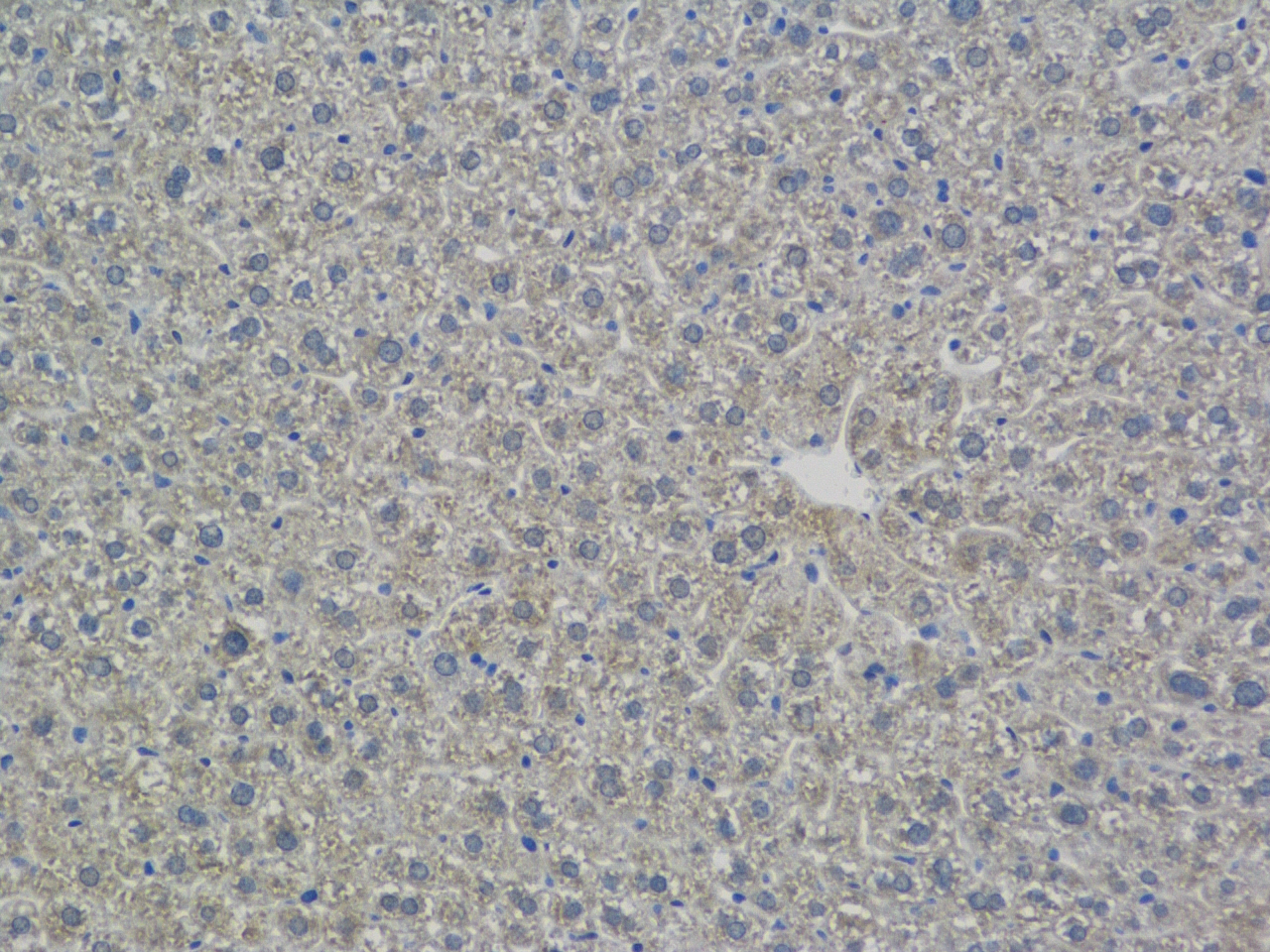

Supplement: Supplementary file 1 [file toxins-18-00278-s001.zip › Figure S2. Uncropped original full-size hepatic tissue immunostaining images corresponding to Figure 4C/NnV+EGCG-GPX4.jpg]

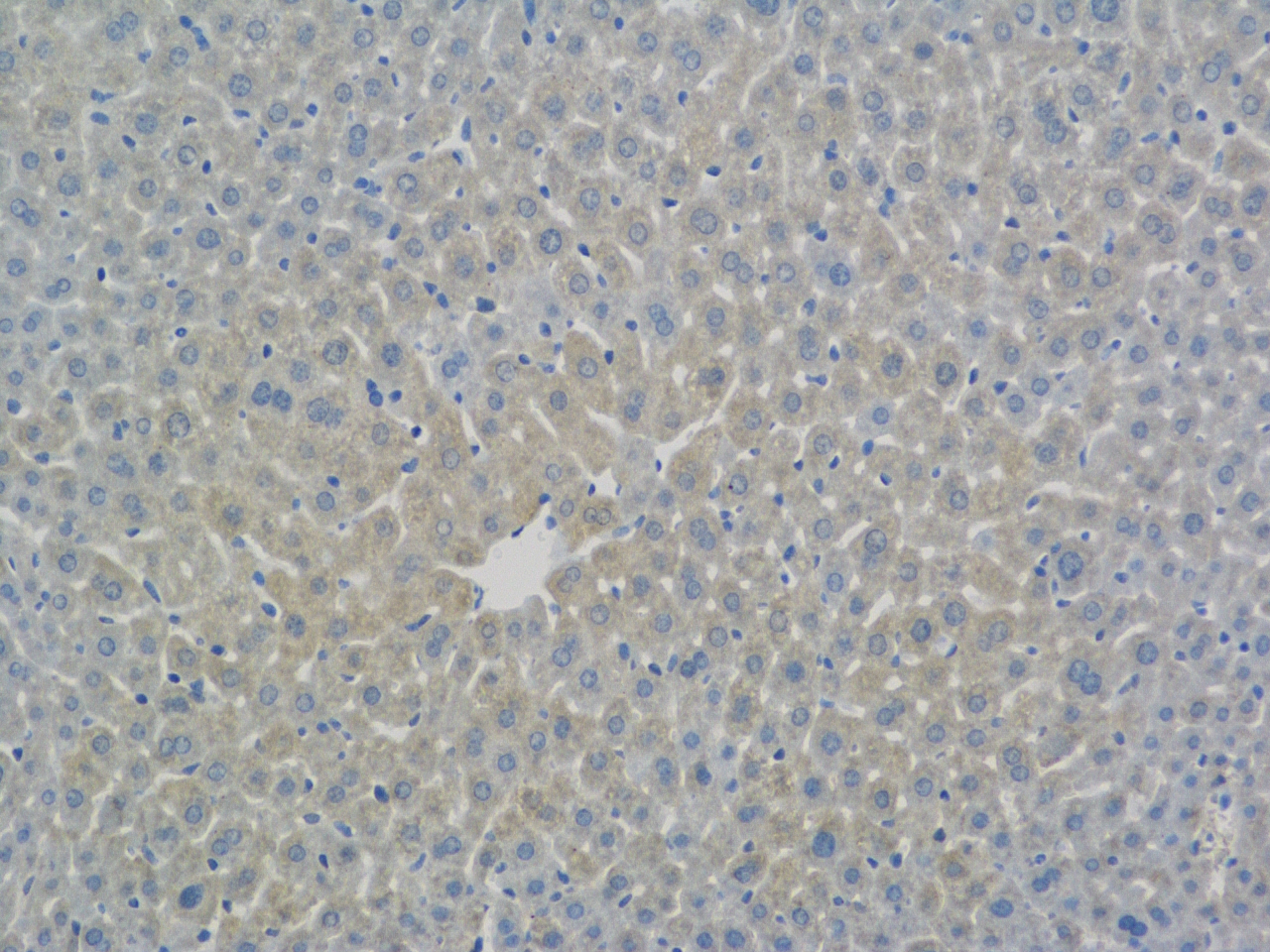

Supplement: Supplementary file 1 [file toxins-18-00278-s001.zip › Figure S2. Uncropped original full-size hepatic tissue immunostaining images corresponding to Figure 4C/NnV+EGCG-HO-1.jpg]

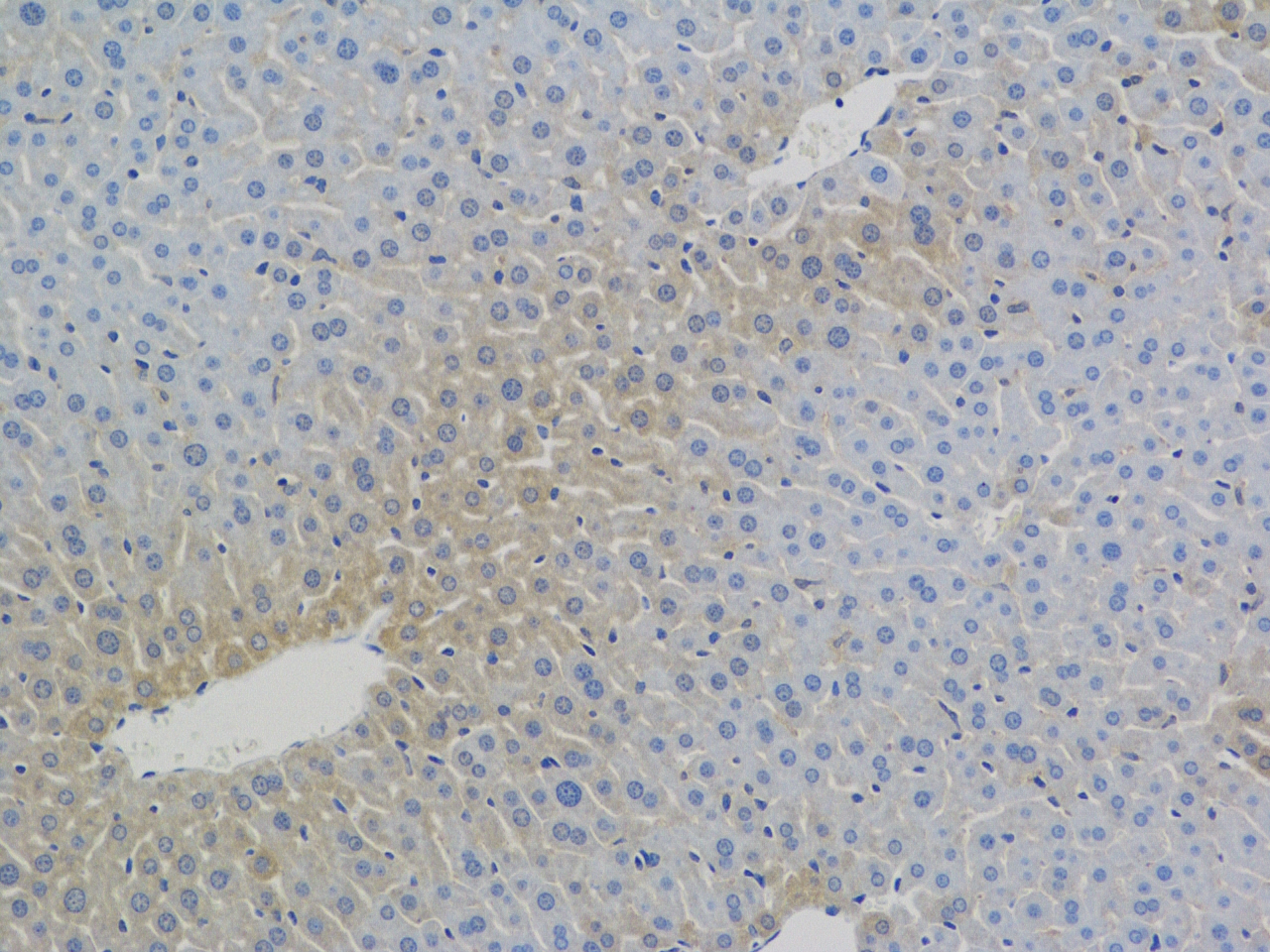

Supplement: Supplementary file 1 [file toxins-18-00278-s001.zip › Figure S2. Uncropped original full-size hepatic tissue immunostaining images corresponding to Figure 4C/NnV+EGCG-Nrf-2.jpg]

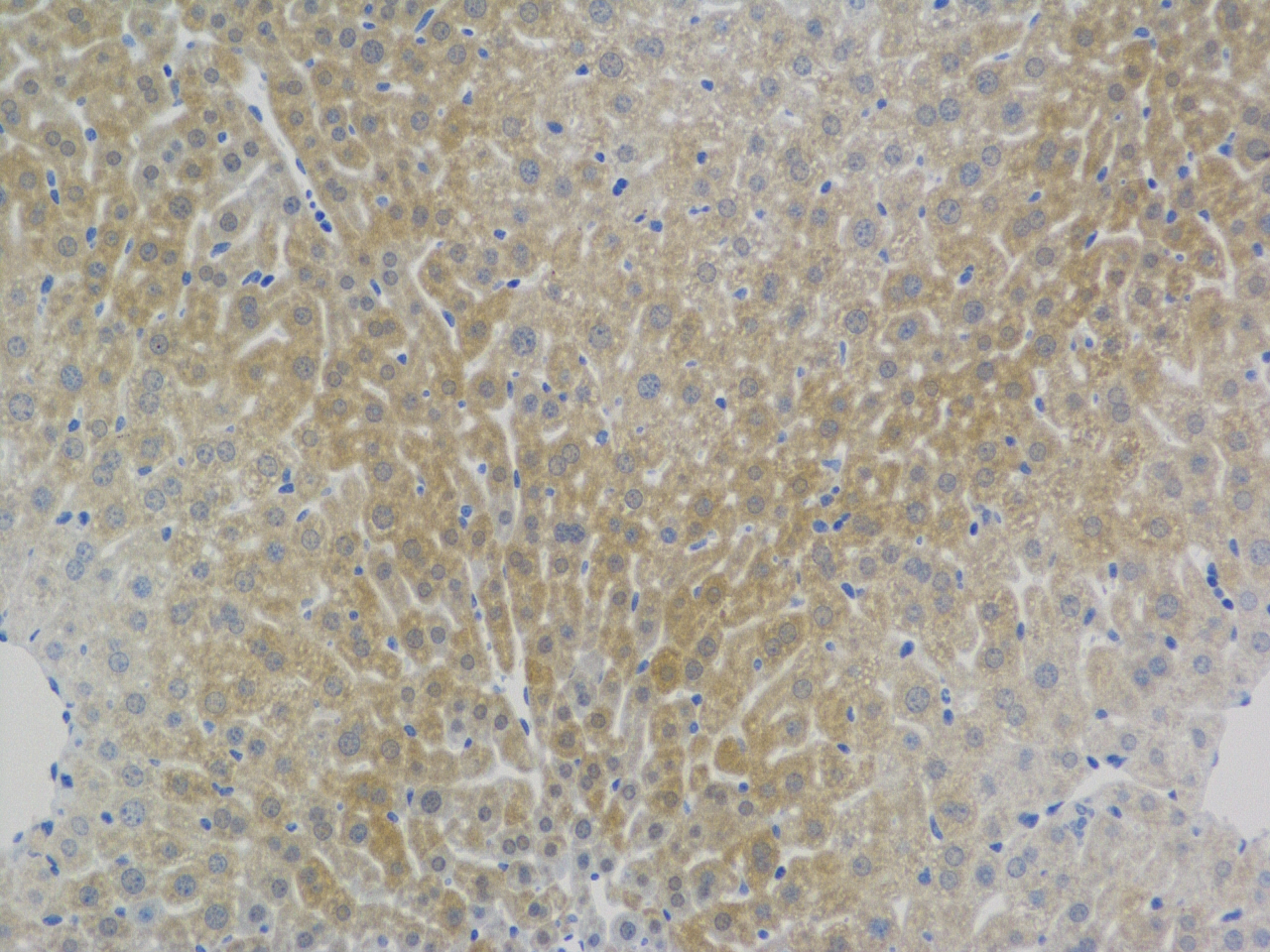

Supplement: Supplementary file 1 [file toxins-18-00278-s001.zip › Figure S2. Uncropped original full-size hepatic tissue immunostaining images corresponding to Figure 4C/NnV-CAT.jpg]

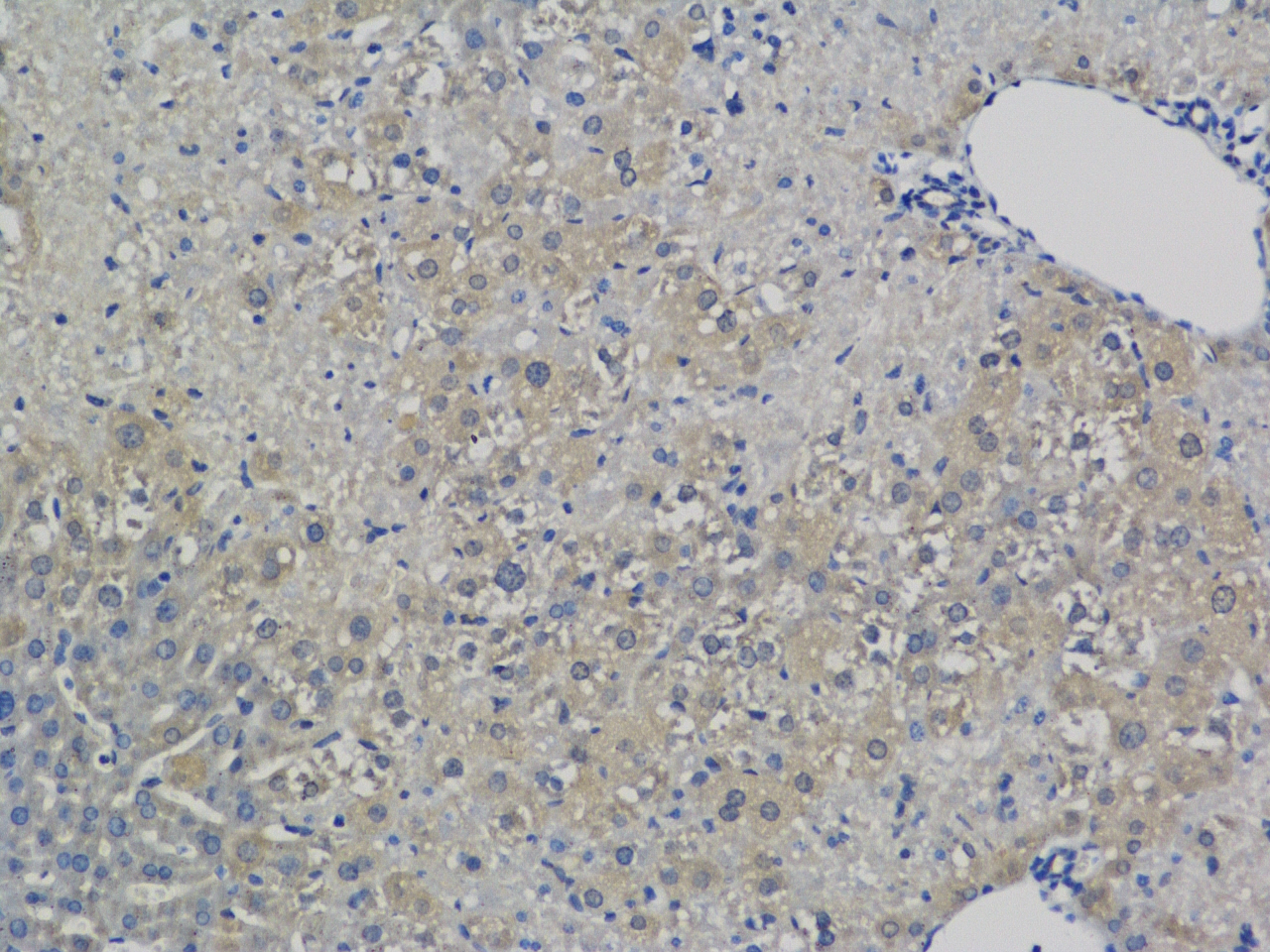

Supplement: Supplementary file 1 [file toxins-18-00278-s001.zip › Figure S2. Uncropped original full-size hepatic tissue immunostaining images corresponding to Figure 4C/NnV-GPX4.jpg]

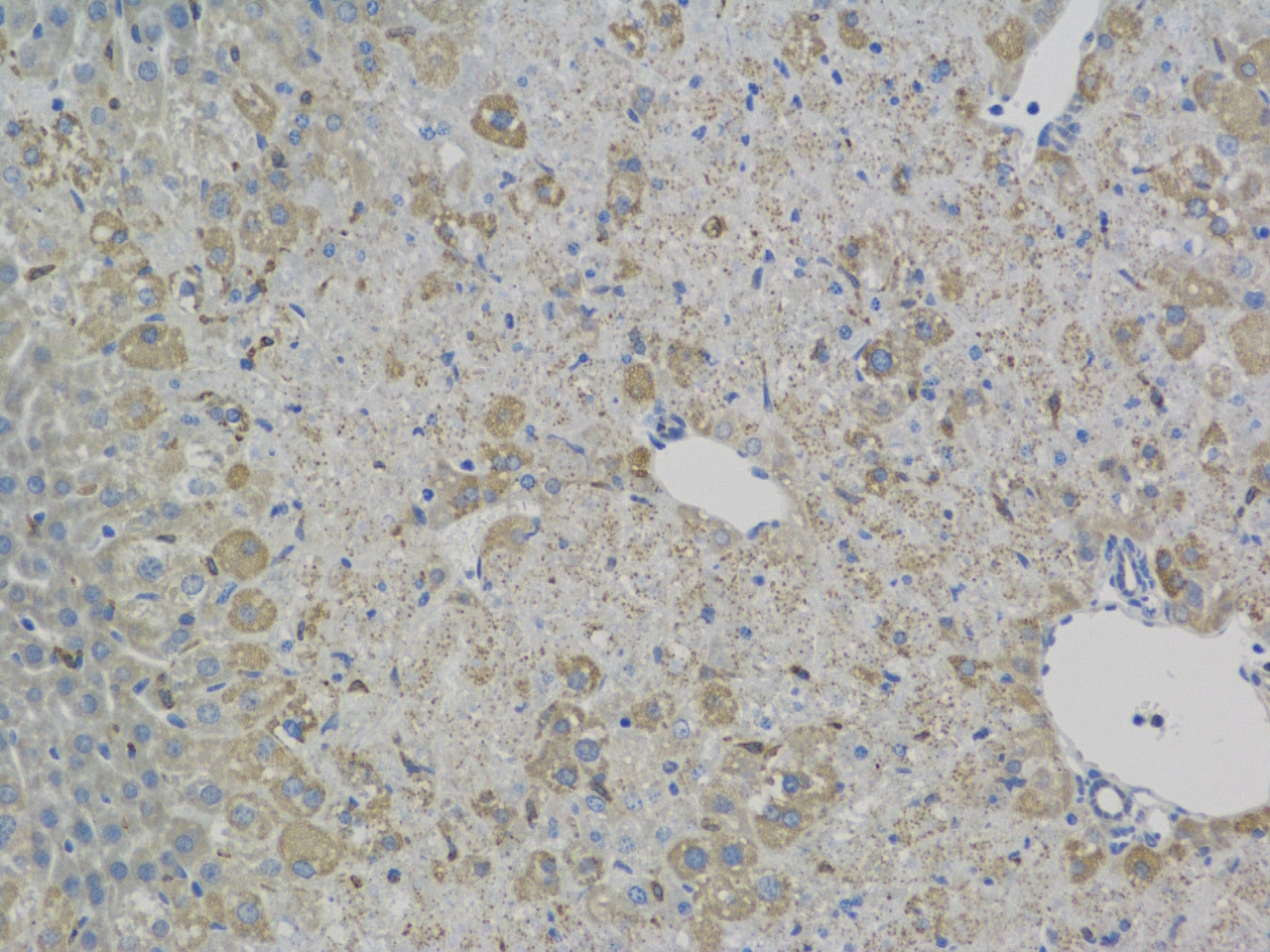

Supplement: Supplementary file 1 [file toxins-18-00278-s001.zip › Figure S2. Uncropped original full-size hepatic tissue immunostaining images corresponding to Figure 4C/NnV-HO-1.jpg]

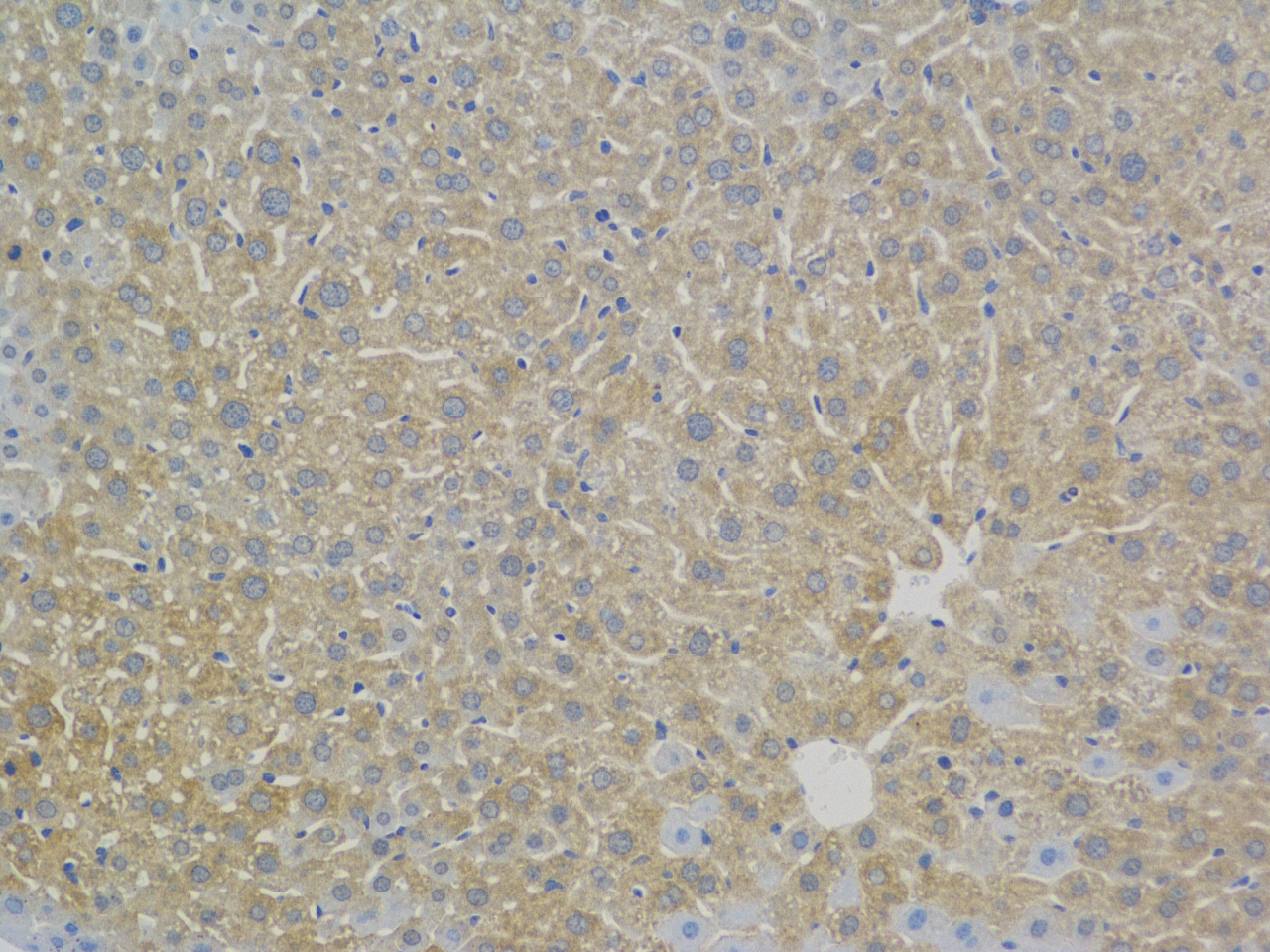

Supplement: Supplementary file 1 [file toxins-18-00278-s001.zip › Figure S2. Uncropped original full-size hepatic tissue immunostaining images corresponding to Figure 4C/NnV-Nrf-2.jpg]

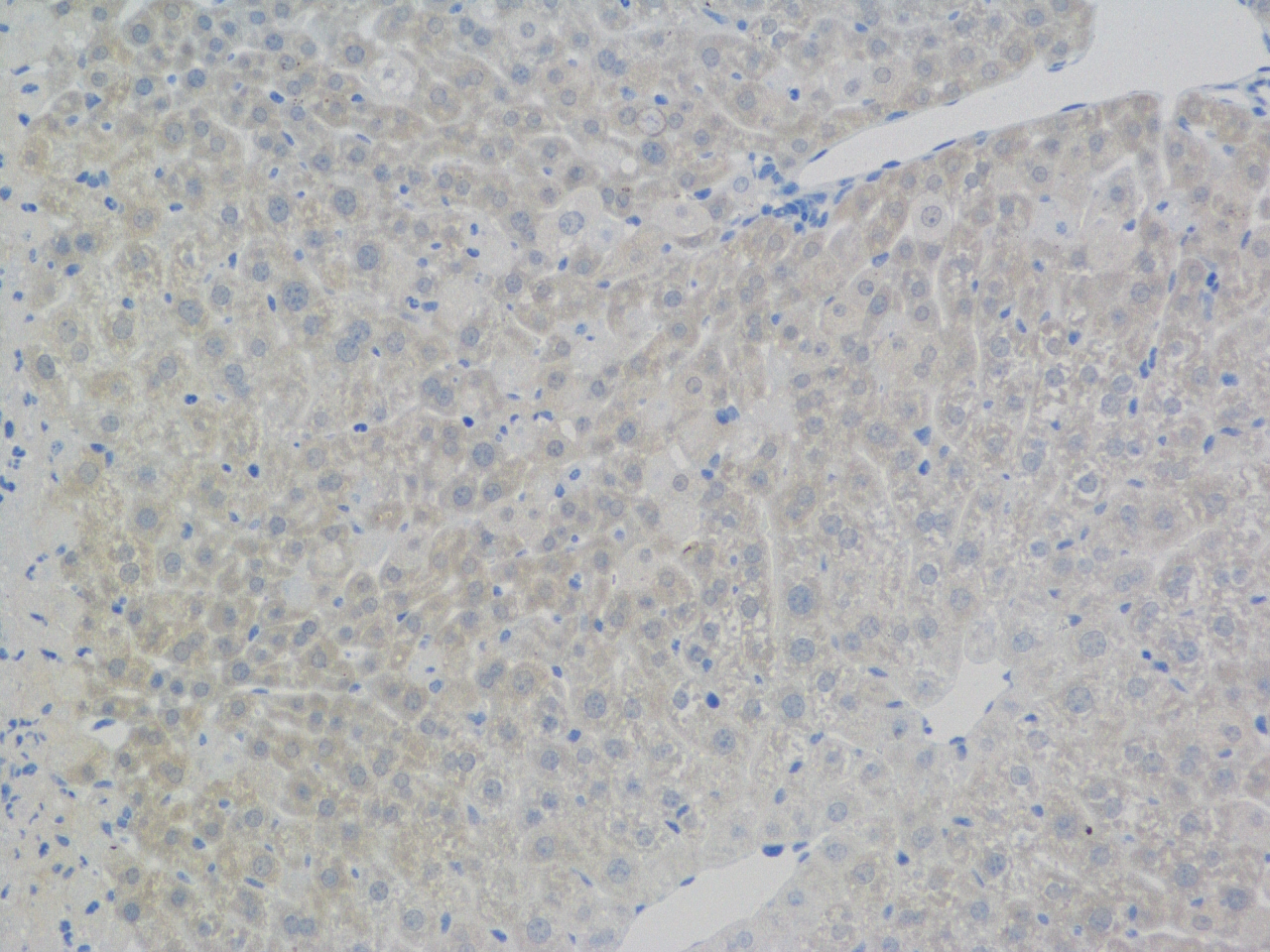

Supplement: Supplementary file 1 [file toxins-18-00278-s001.zip › Figure S2. Uncropped original full-size hepatic tissue immunostaining images corresponding to Figure 4C/PBS-CAT.jpg]

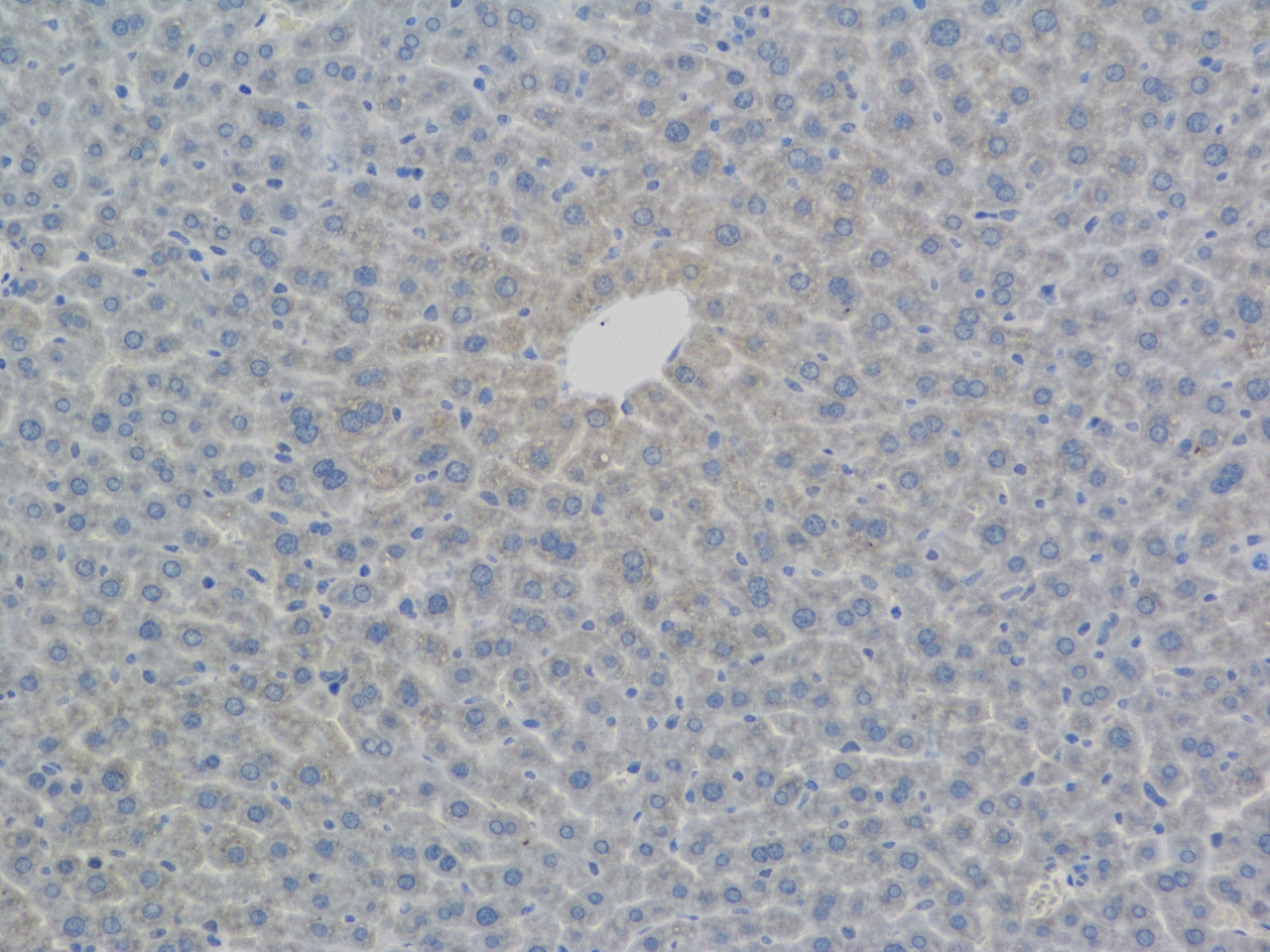

Supplement: Supplementary file 1 [file toxins-18-00278-s001.zip › Figure S2. Uncropped original full-size hepatic tissue immunostaining images corresponding to Figure 4C/PBS-GPX4.jpg]

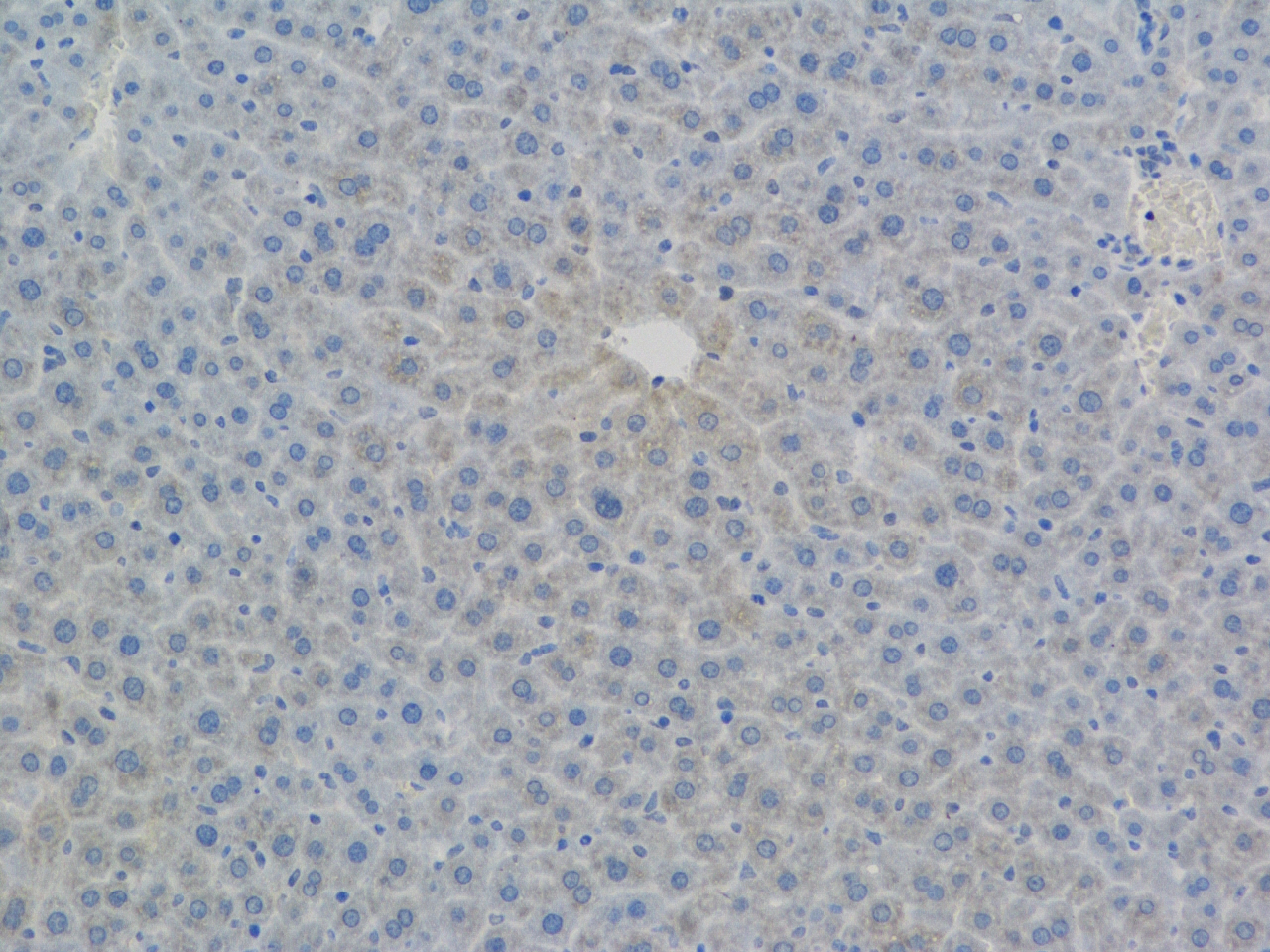

Supplement: Supplementary file 1 [file toxins-18-00278-s001.zip › Figure S2. Uncropped original full-size hepatic tissue immunostaining images corresponding to Figure 4C/PBS-HO-1.jpg]

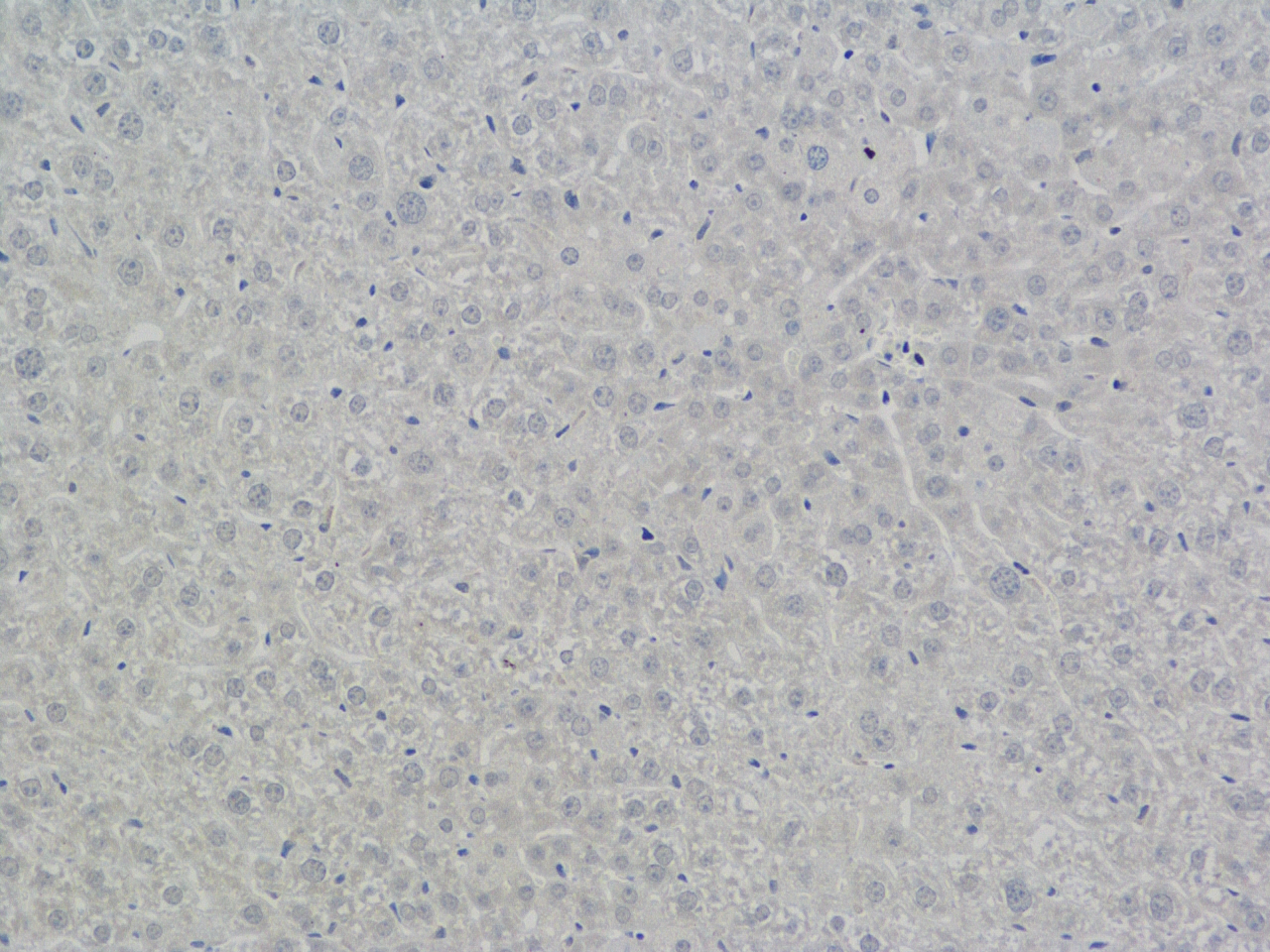

Supplement: Supplementary file 1 [file toxins-18-00278-s001.zip › Figure S2. Uncropped original full-size hepatic tissue immunostaining images corresponding to Figure 4C/PBS-Nrf-2.jpg]

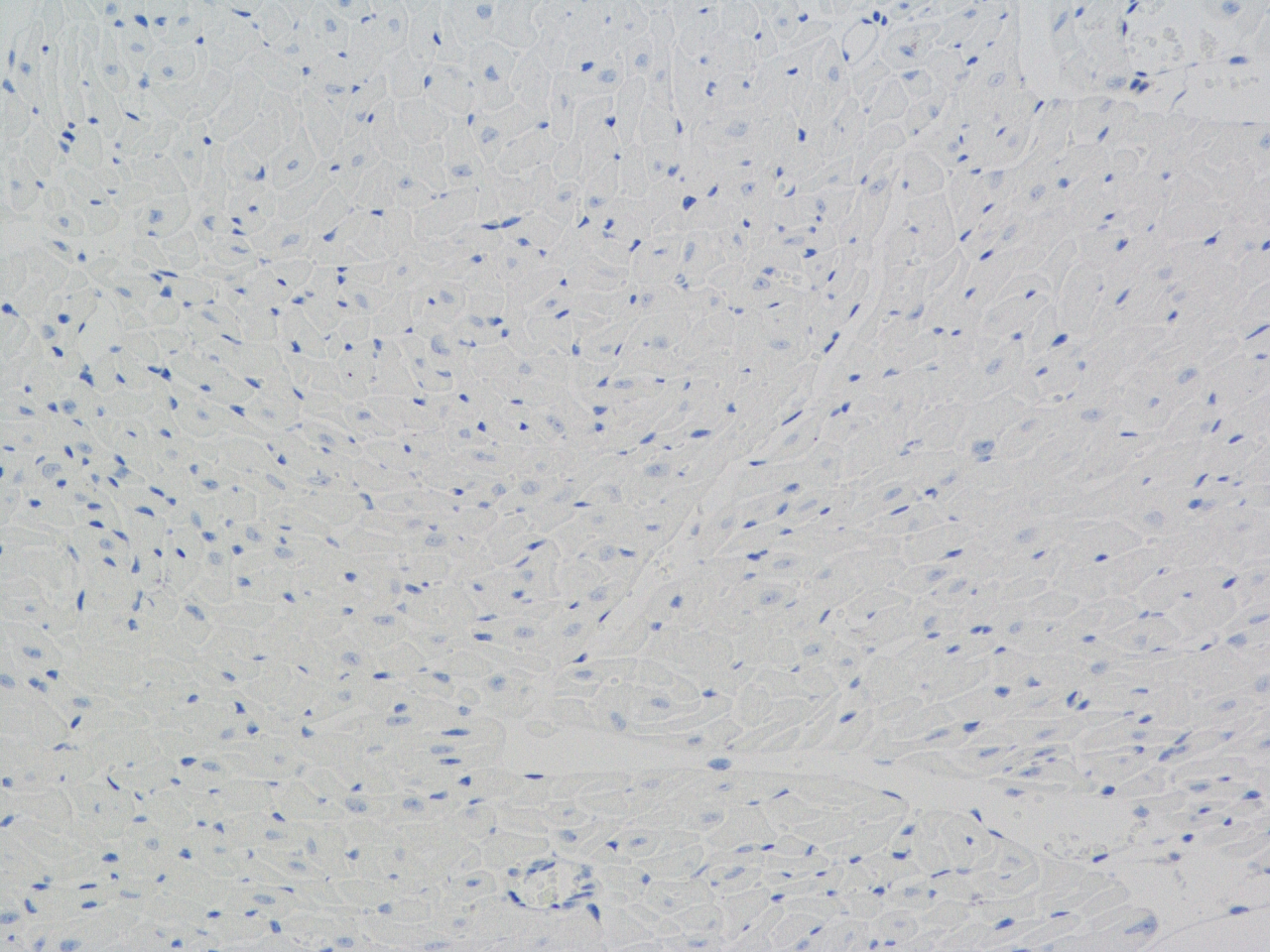

Supplement: Supplementary file 1 [file toxins-18-00278-s001.zip › Figure S3. Uncropped original full-size cardiac tissue immunostaining images corresponding to Figure 5D/EGCG-4IL-6.jpg]

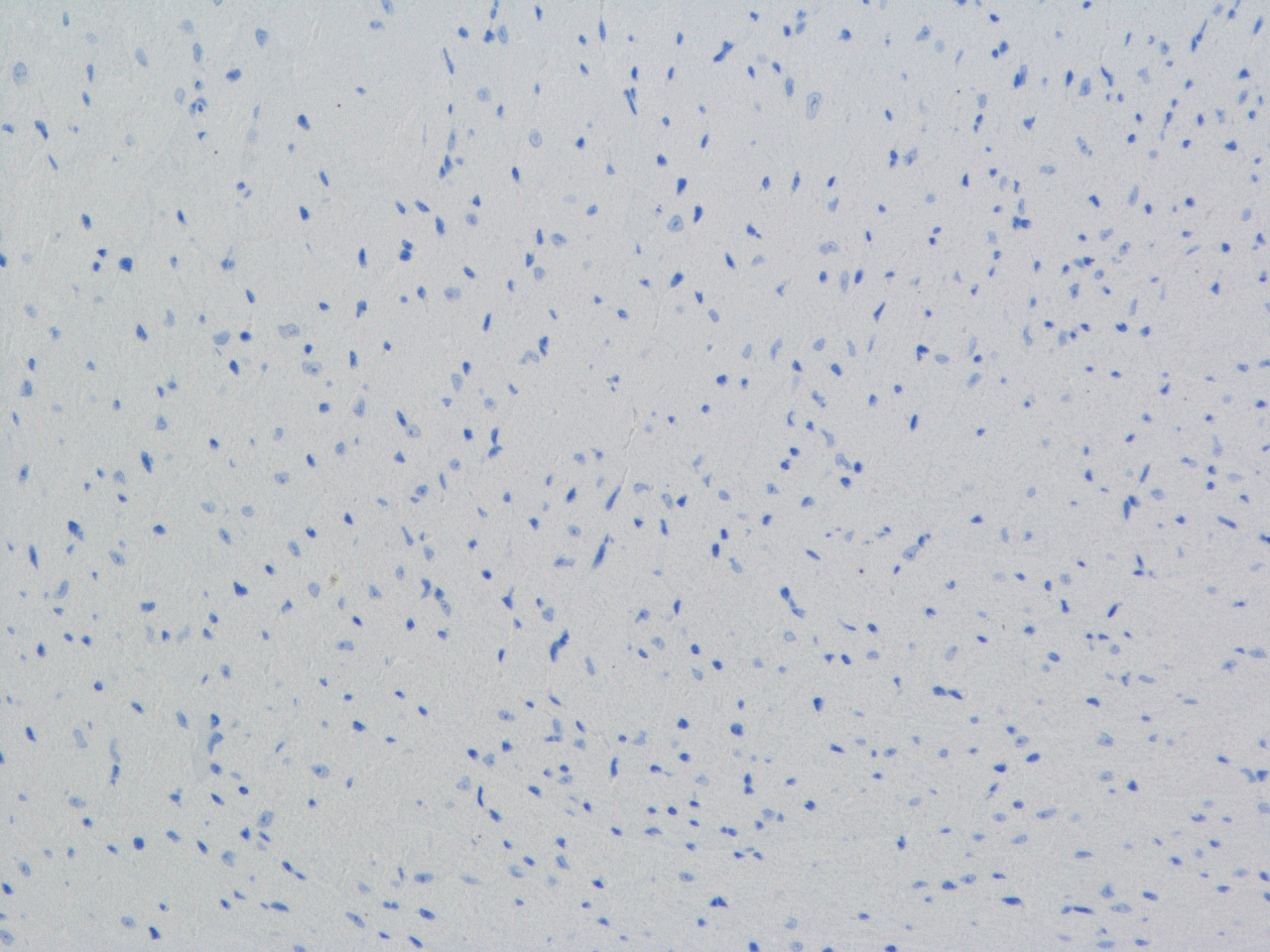

Supplement: Supplementary file 1 [file toxins-18-00278-s001.zip › Figure S3. Uncropped original full-size cardiac tissue immunostaining images corresponding to Figure 5D/EGCG-IL -1β.jpg]

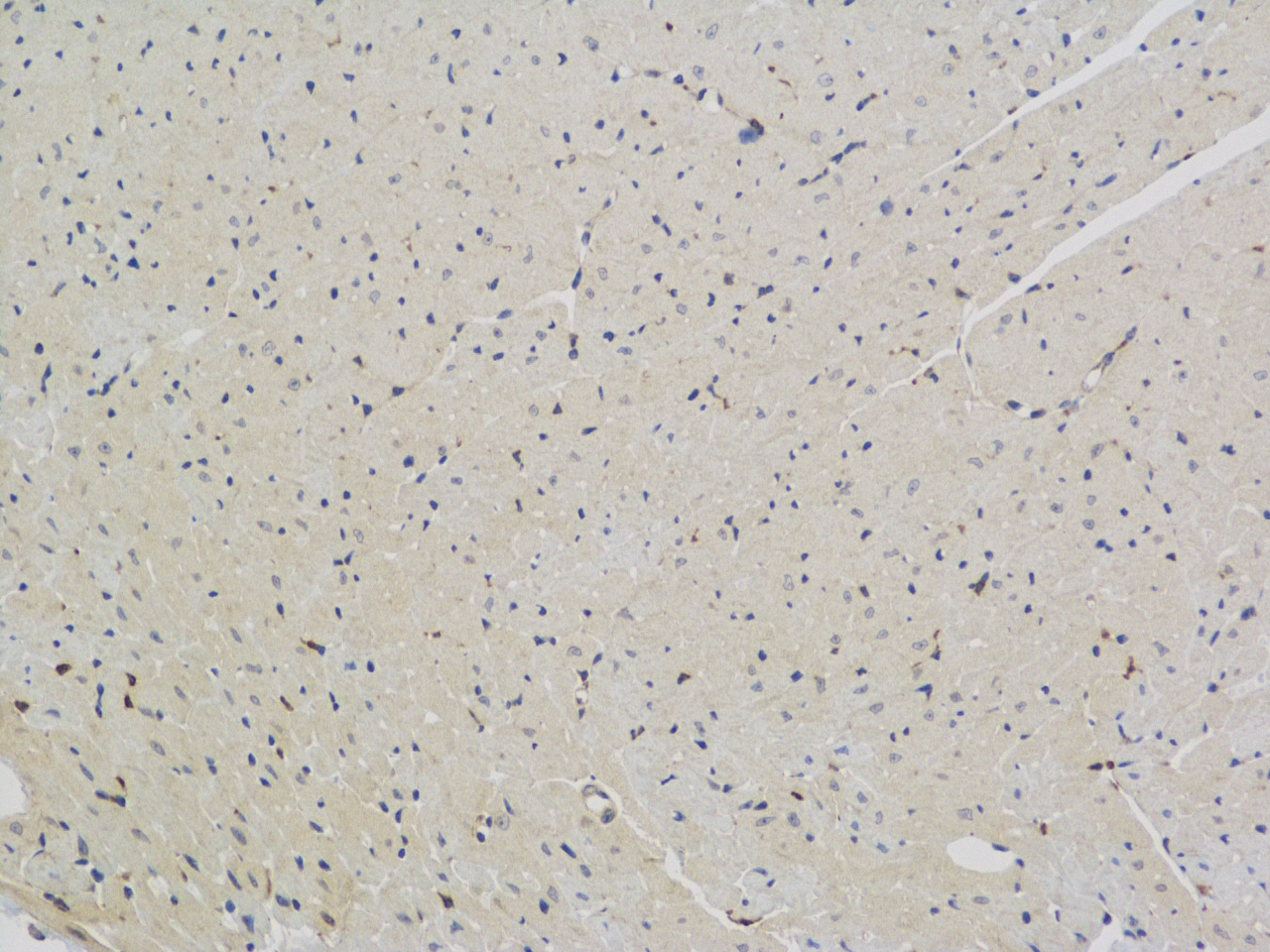

Supplement: Supplementary file 1 [file toxins-18-00278-s001.zip › Figure S3. Uncropped original full-size cardiac tissue immunostaining images corresponding to Figure 5D/NnV+EGCG-IL-1β.jpg]

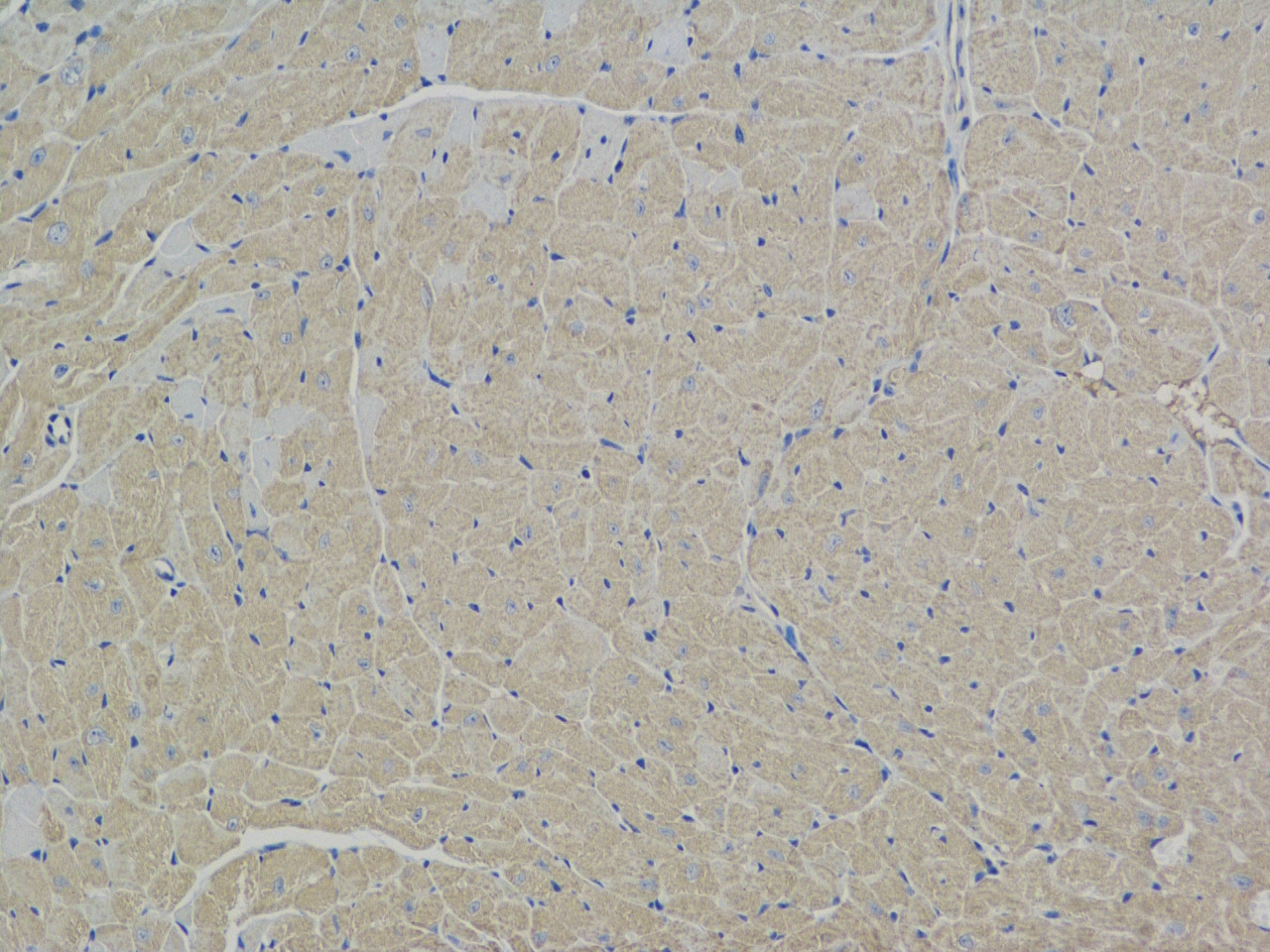

Supplement: Supplementary file 1 [file toxins-18-00278-s001.zip › Figure S3. Uncropped original full-size cardiac tissue immunostaining images corresponding to Figure 5D/NnV+EGCG-IL-6.jpg]

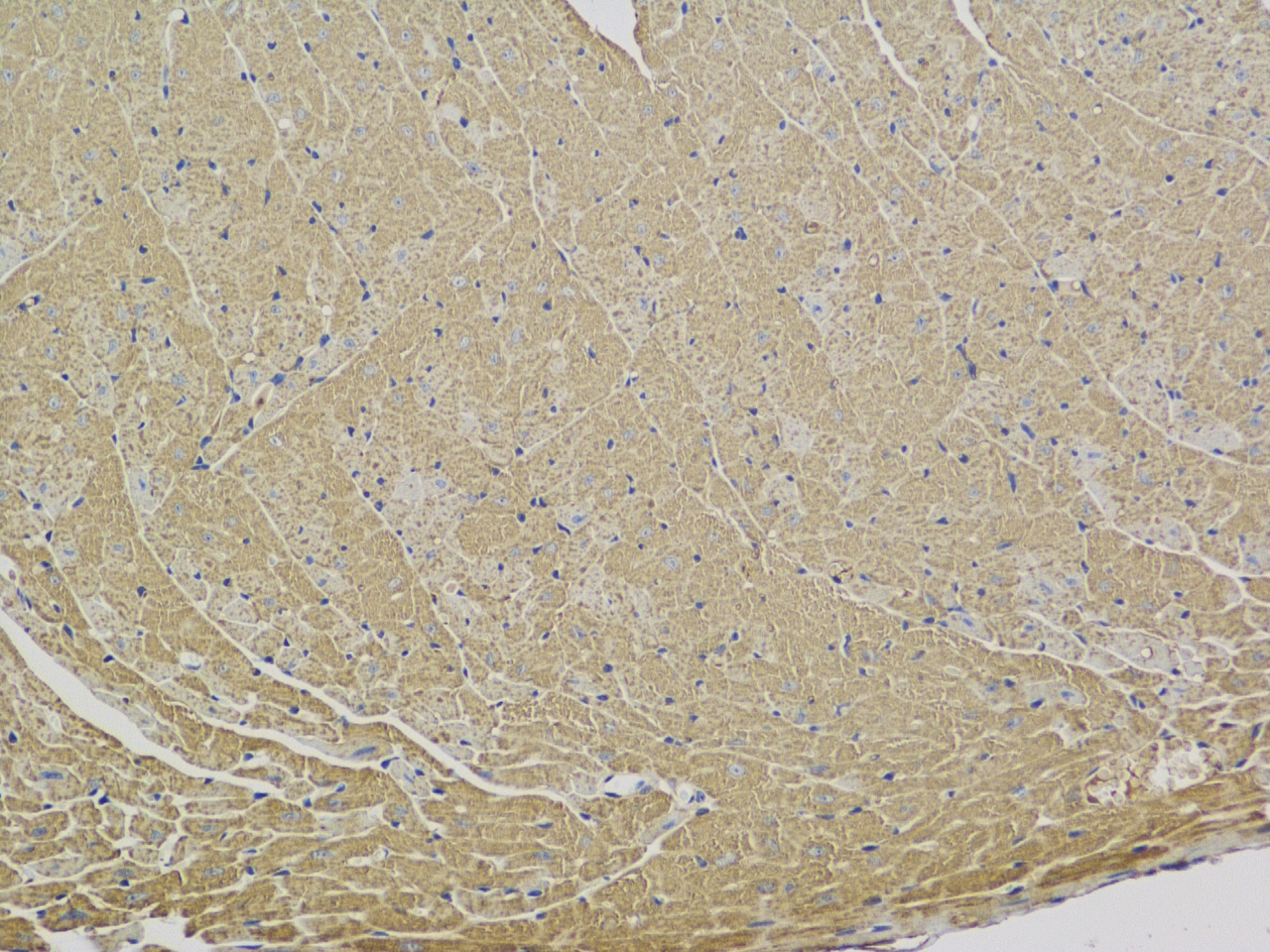

Supplement: Supplementary file 1 [file toxins-18-00278-s001.zip › Figure S3. Uncropped original full-size cardiac tissue immunostaining images corresponding to Figure 5D/NnV-IL-1β.jpg]

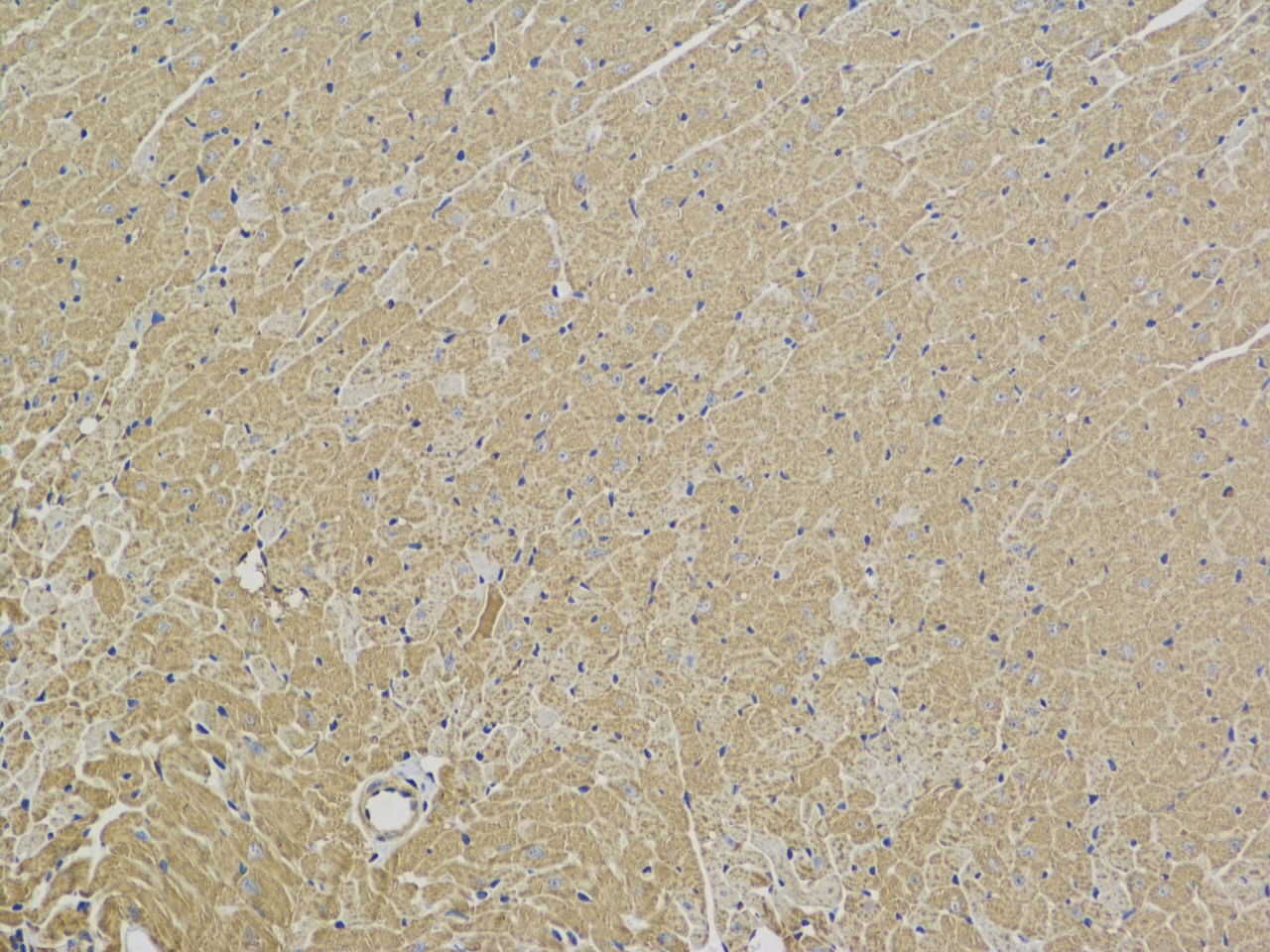

Supplement: Supplementary file 1 [file toxins-18-00278-s001.zip › Figure S3. Uncropped original full-size cardiac tissue immunostaining images corresponding to Figure 5D/NnV-IL-6.jpg]

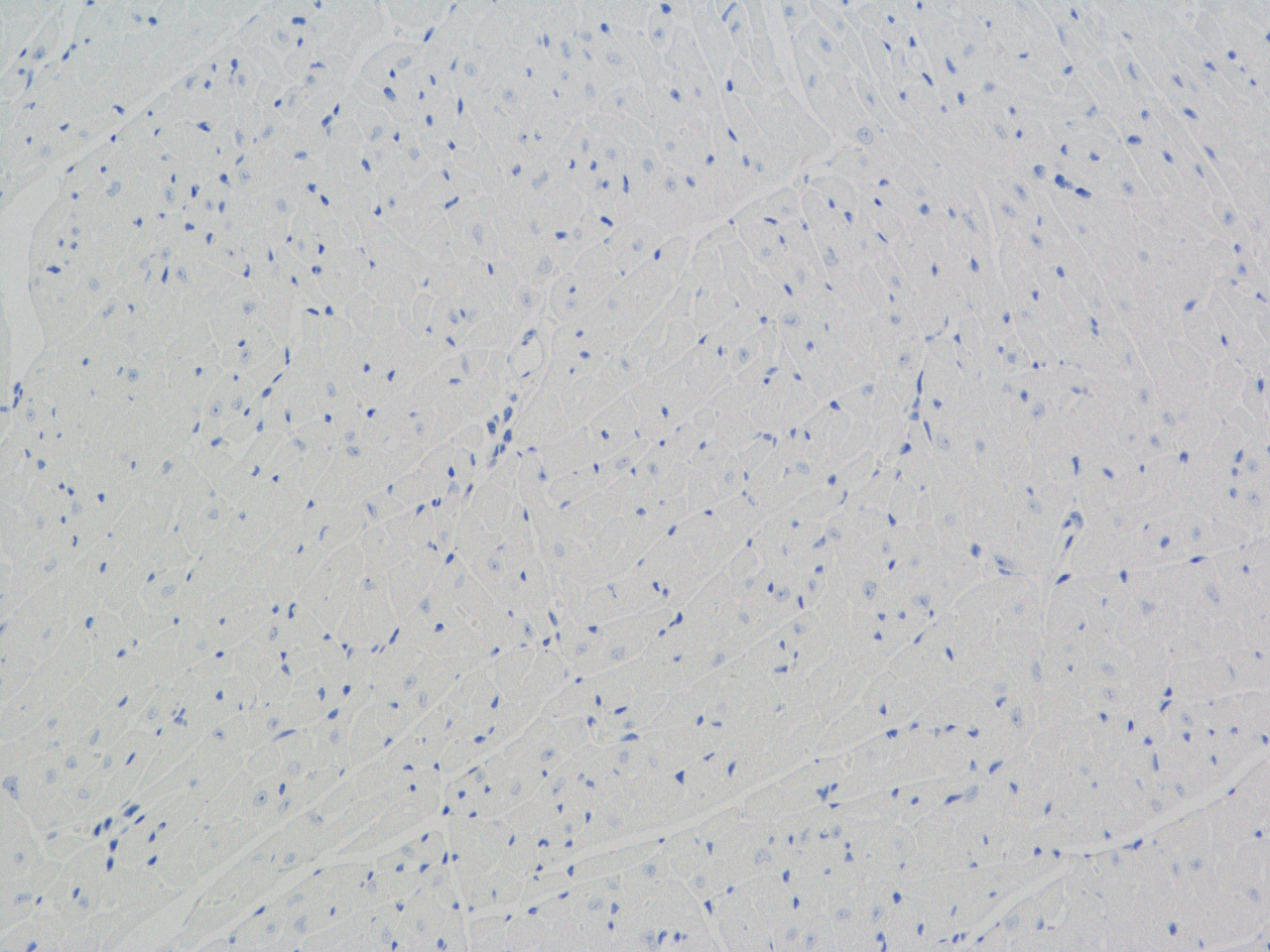

Supplement: Supplementary file 1 [file toxins-18-00278-s001.zip › Figure S3. Uncropped original full-size cardiac tissue immunostaining images corresponding to Figure 5D/PBS-IL-1β.jpg]

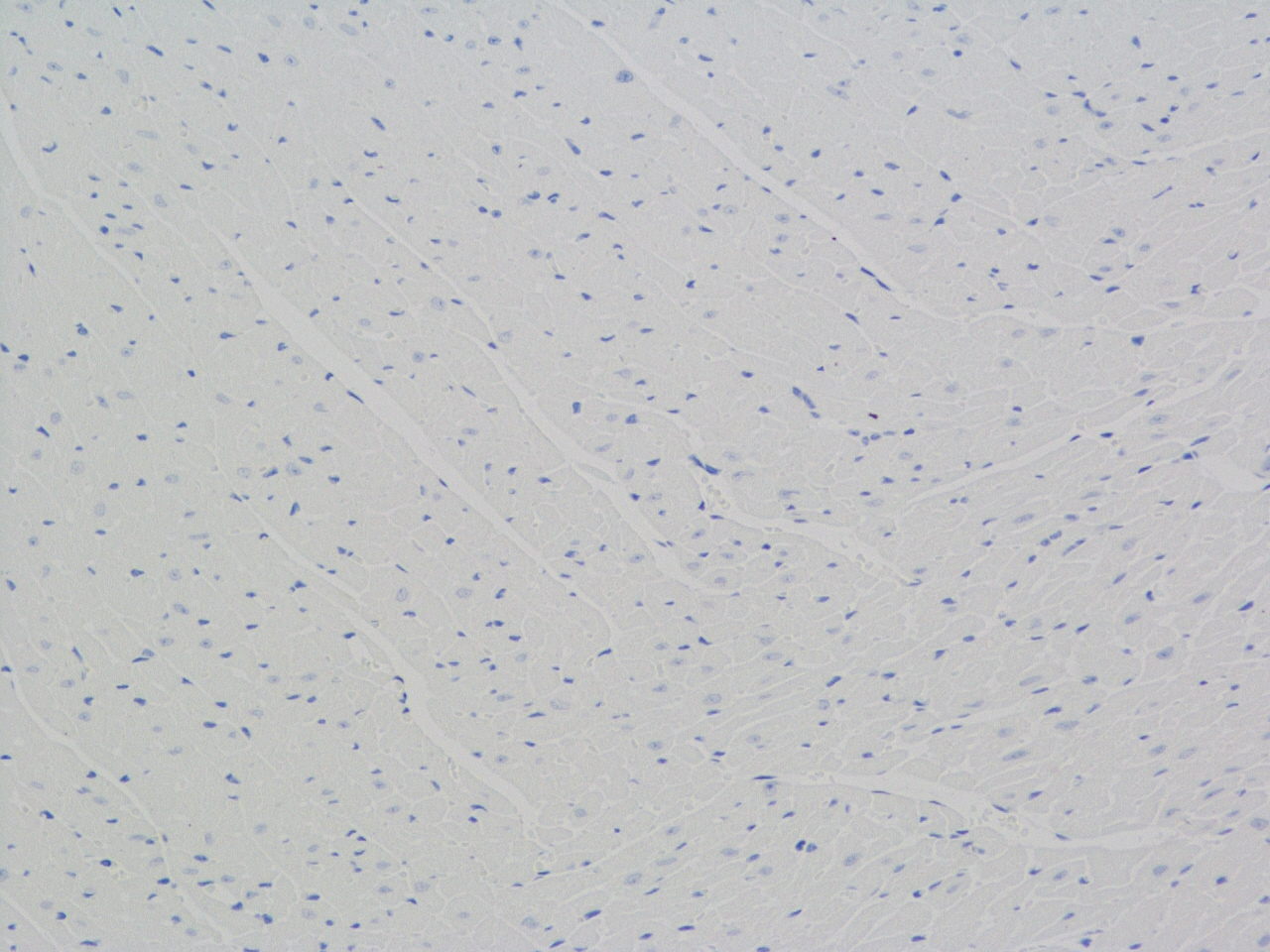

Supplement: Supplementary file 1 [file toxins-18-00278-s001.zip › Figure S3. Uncropped original full-size cardiac tissue immunostaining images corresponding to Figure 5D/PBS-IL-6.jpg]

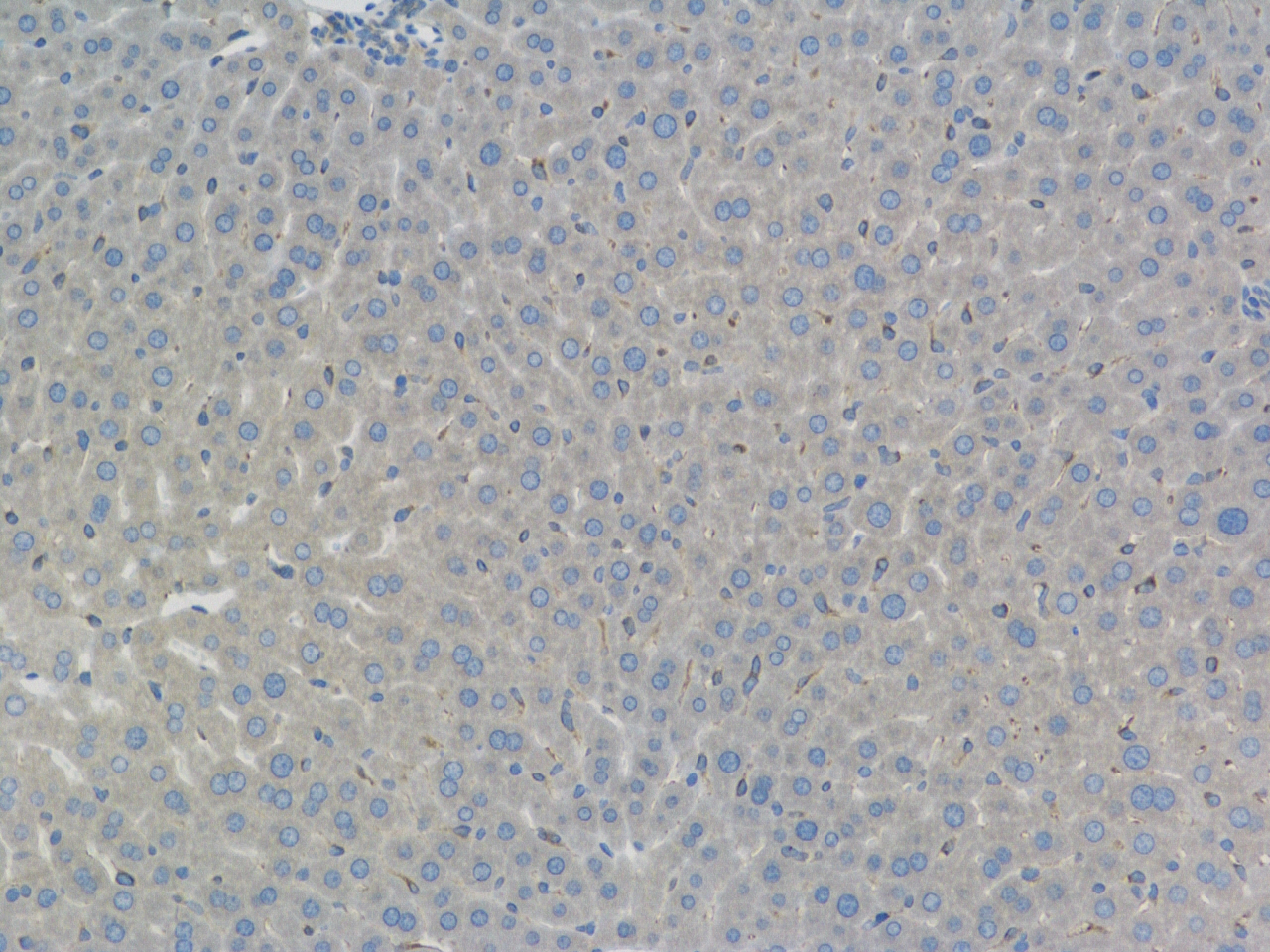

Supplement: Supplementary file 1 [file toxins-18-00278-s001.zip › Figure S4. Uncropped original full-size cardiac tissue immunostaining images corresponding to Figure 6C/EGCG-IL-1β.jpg]

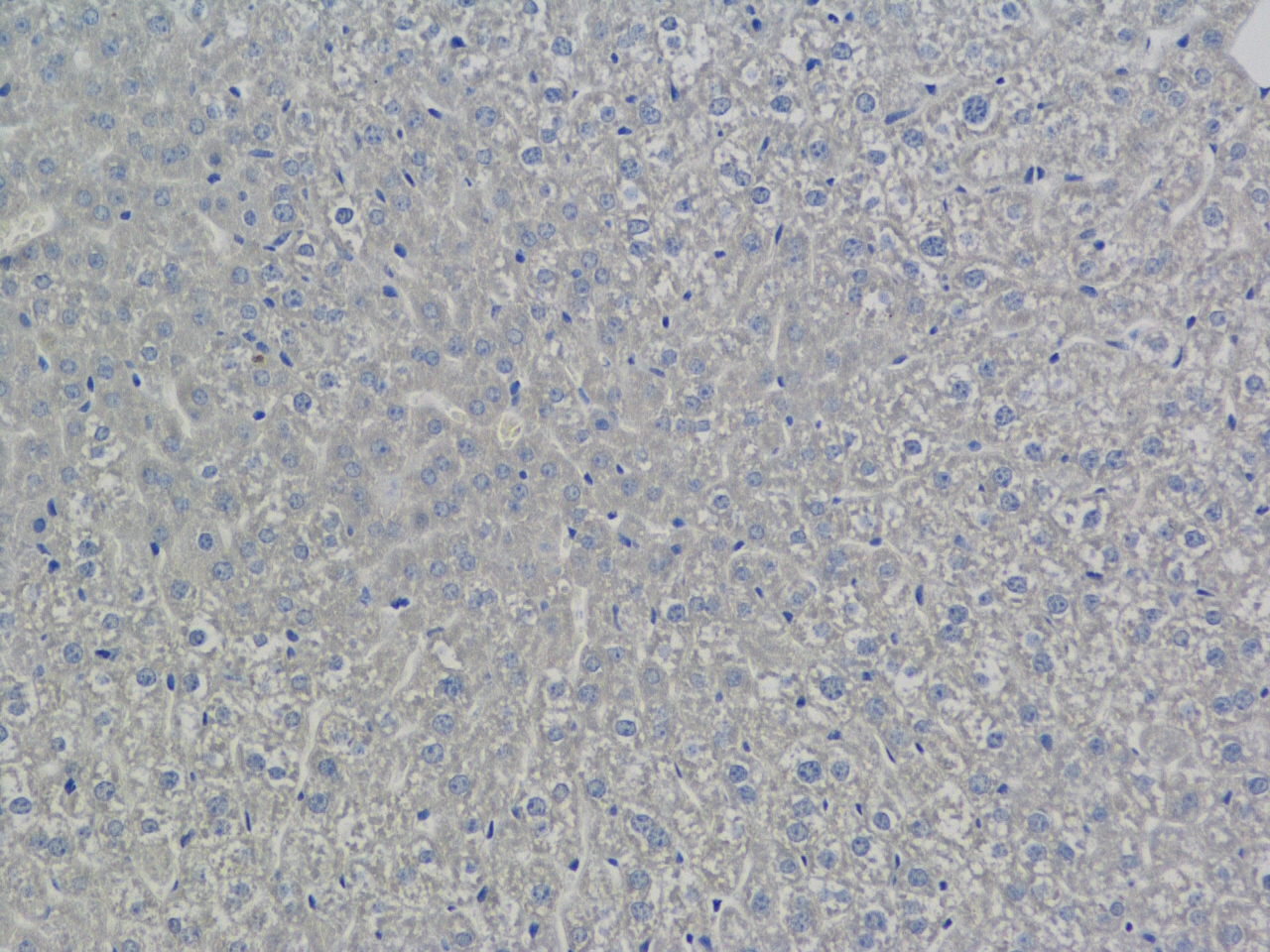

Supplement: Supplementary file 1 [file toxins-18-00278-s001.zip › Figure S4. Uncropped original full-size cardiac tissue immunostaining images corresponding to Figure 6C/EGCG-IL-6.jpg]

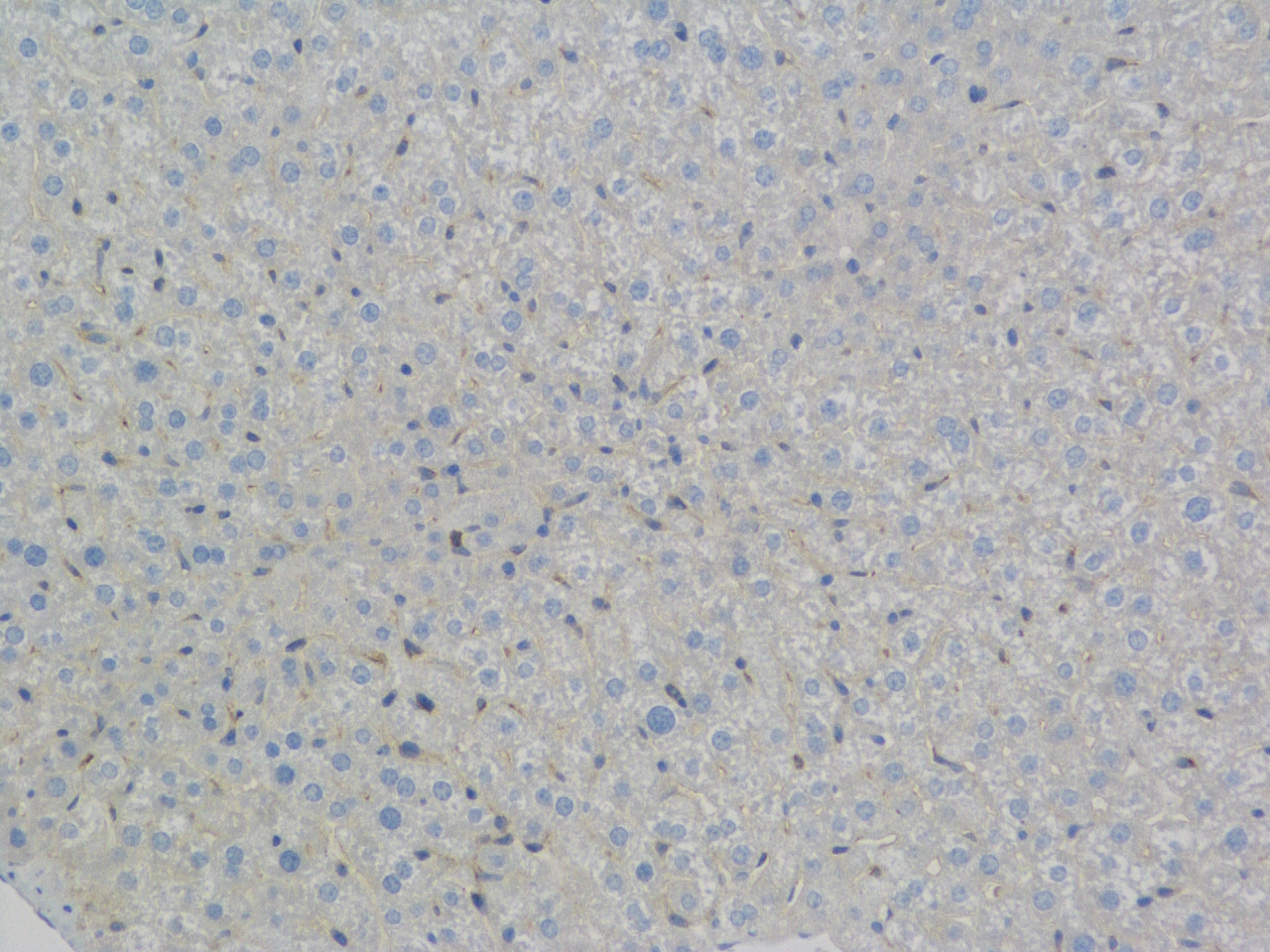

Supplement: Supplementary file 1 [file toxins-18-00278-s001.zip › Figure S4. Uncropped original full-size cardiac tissue immunostaining images corresponding to Figure 6C/NnV+EGCG-IL-1.jpg]

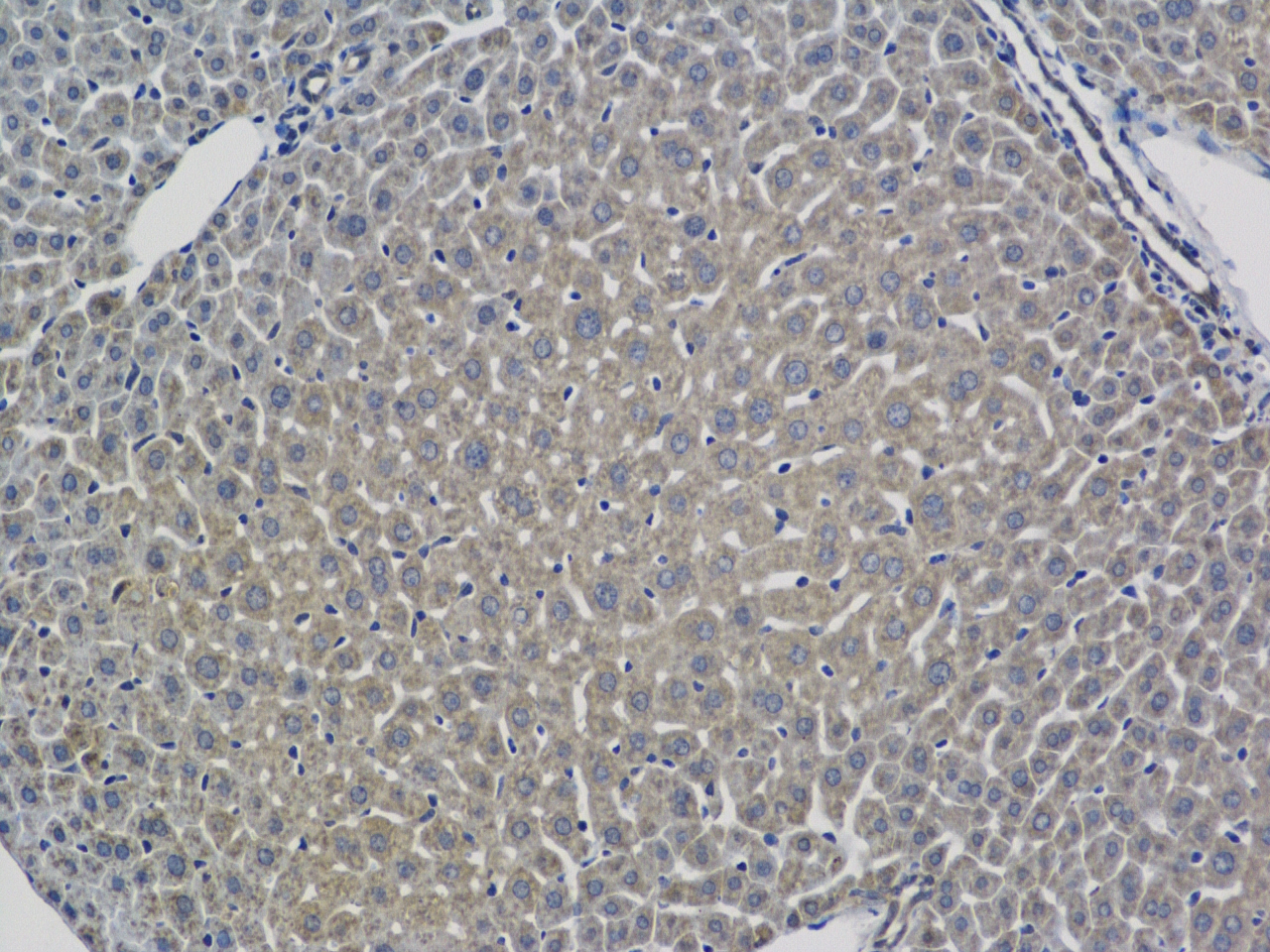

Supplement: Supplementary file 1 [file toxins-18-00278-s001.zip › Figure S4. Uncropped original full-size cardiac tissue immunostaining images corresponding to Figure 6C/NnV+EGCG-IL-6.jpg]

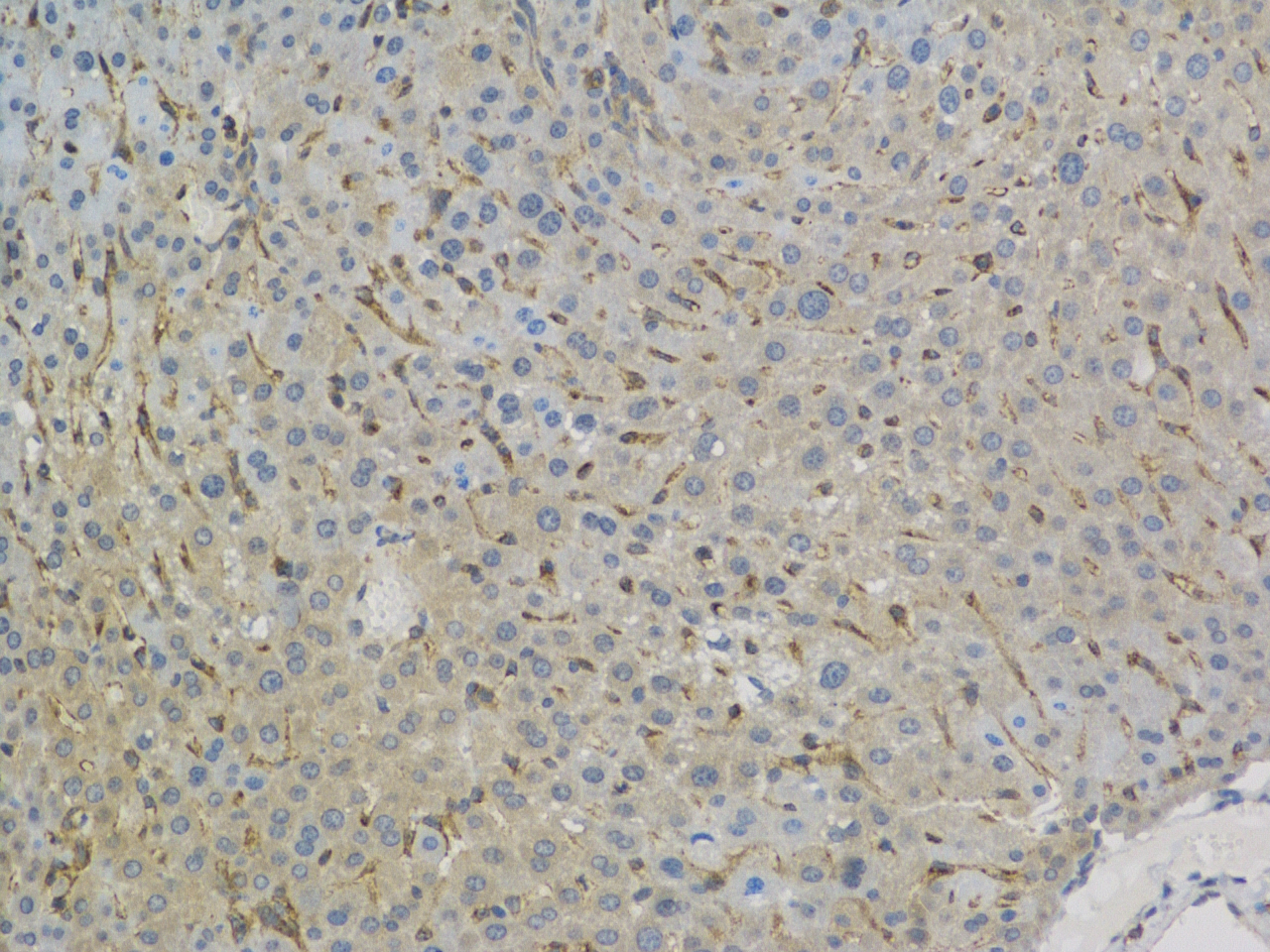

Supplement: Supplementary file 1 [file toxins-18-00278-s001.zip › Figure S4. Uncropped original full-size cardiac tissue immunostaining images corresponding to Figure 6C/NnV-IL-1β.jpg]

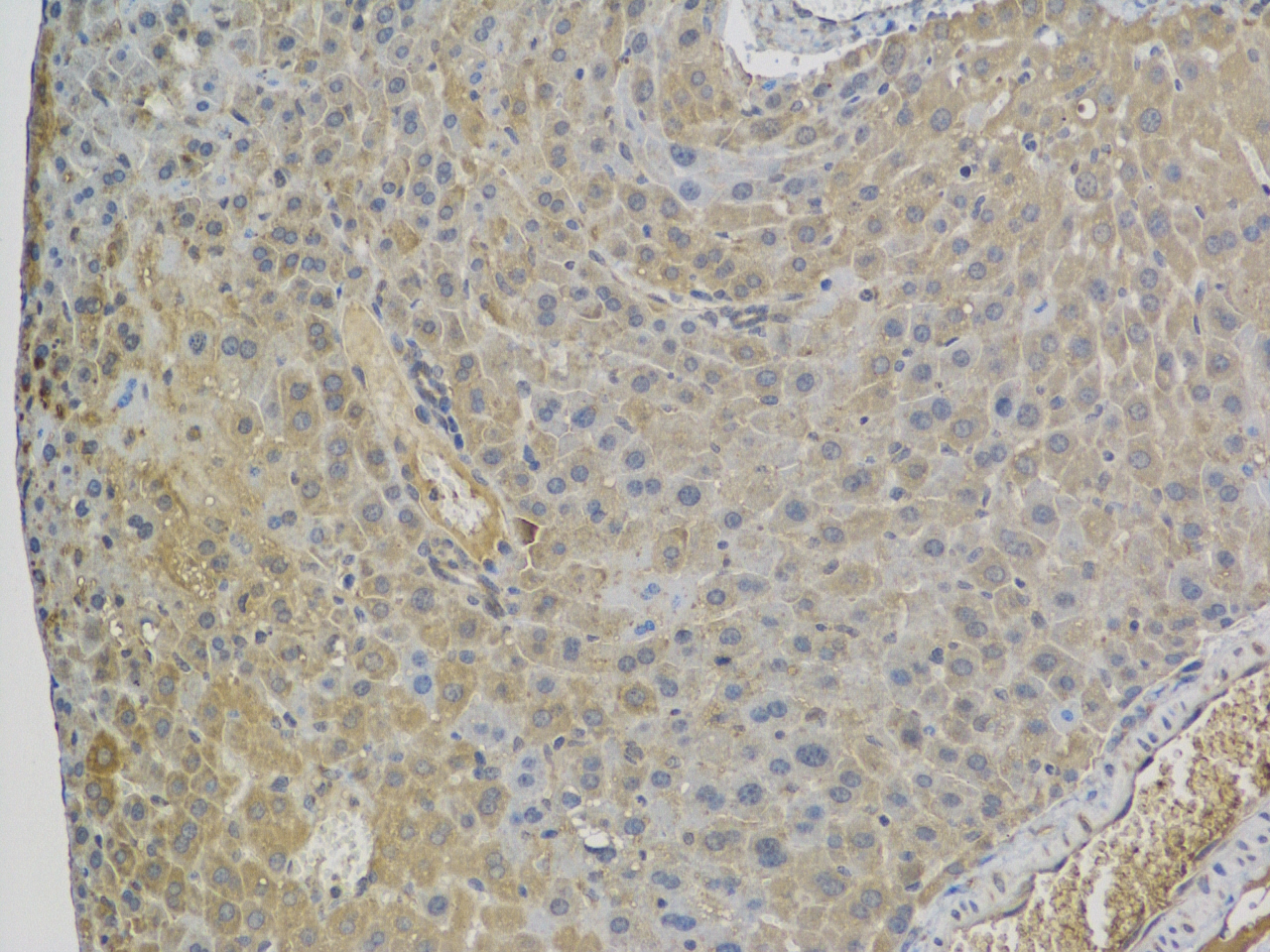

Supplement: Supplementary file 1 [file toxins-18-00278-s001.zip › Figure S4. Uncropped original full-size cardiac tissue immunostaining images corresponding to Figure 6C/NnV-IL-6.jpg]

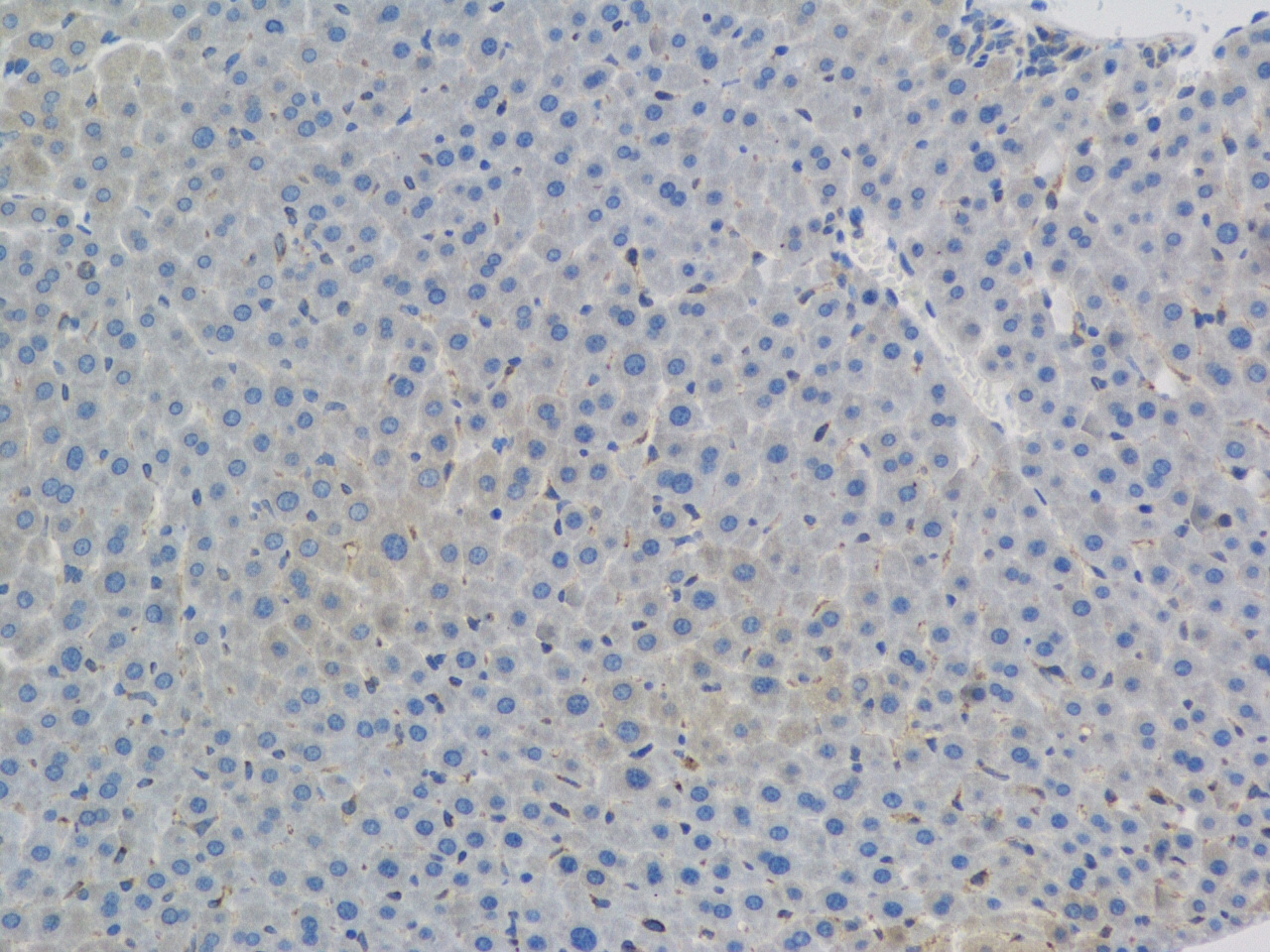

Supplement: Supplementary file 1 [file toxins-18-00278-s001.zip › Figure S4. Uncropped original full-size cardiac tissue immunostaining images corresponding to Figure 6C/PBS-IL-1β.jpg]

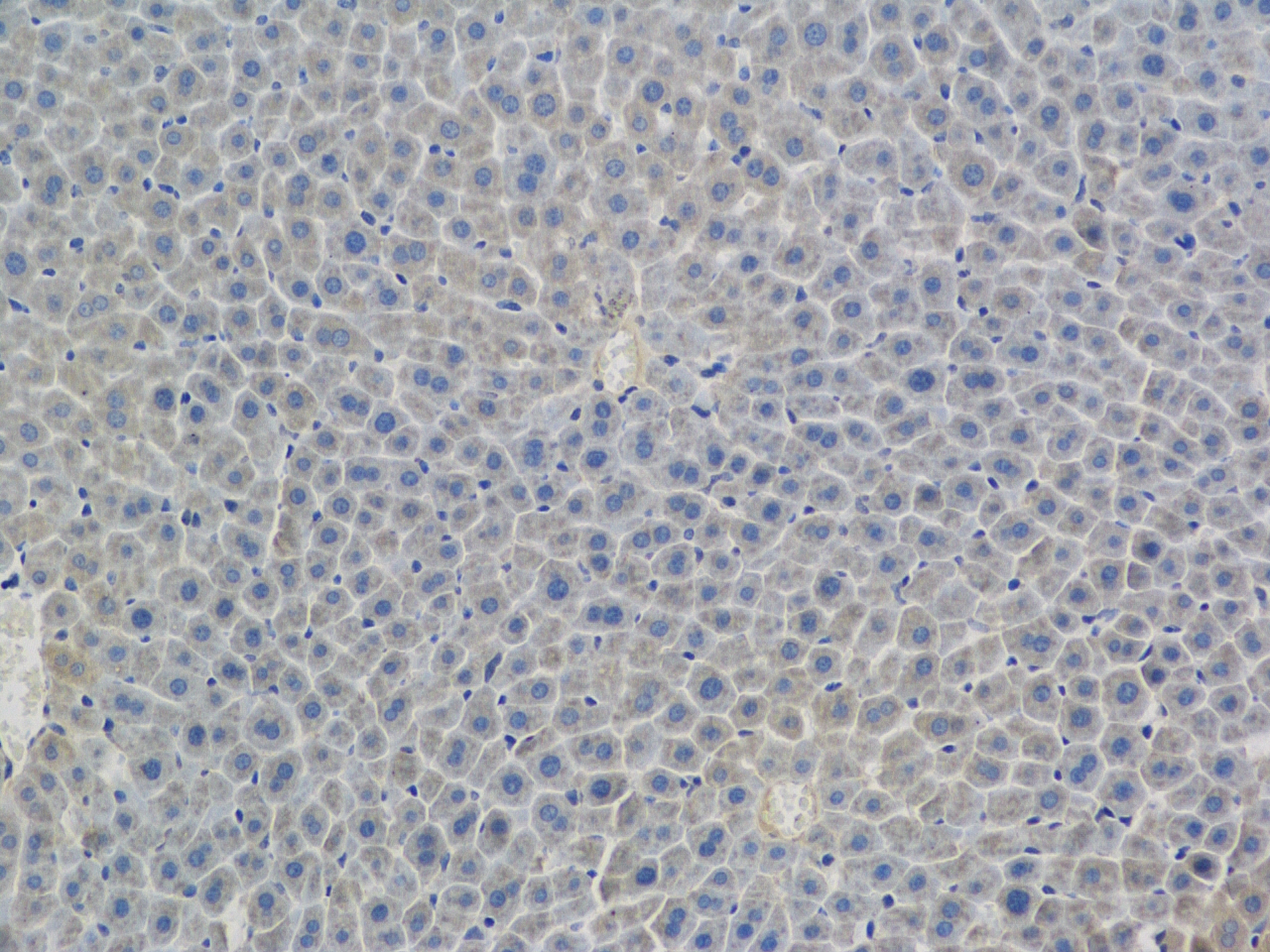

Supplement: Supplementary file 1 [file toxins-18-00278-s001.zip › Figure S4. Uncropped original full-size cardiac tissue immunostaining images corresponding to Figure 6C/PBS-IL-6.jpg]
