# Supplementary figures and images for: Enhanced Protection Against Toxicity of Nemopilema nomurai Venom Using a PEG-EGCG/Tetracycline Hydrochloride Micellar Nanocomplex (part 1 of 2)
Source: Toxins (Basel). 2026 Jun 24;18(7):278. doi: 10.3390/toxins18070278 (PMC13417419; doi:10.3390/toxins18070278)

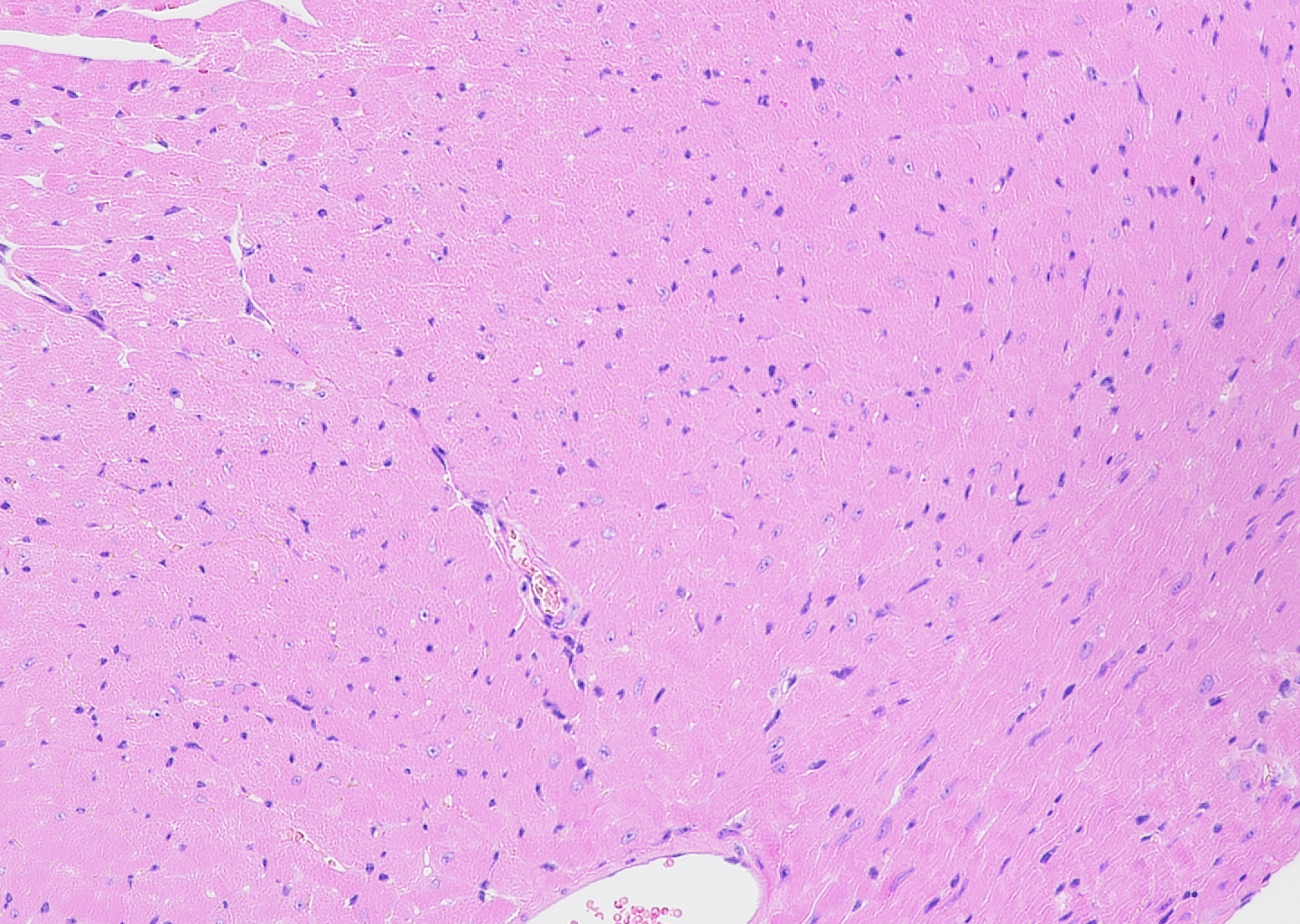

Supplement: Supplementary file 1 [file toxins-18-00278-s001.zip › Figure S7. Original full-size histopathological images of vital organs (heart, liver, spleen, lung, kidney) corresponding to Figure 11B/EGCG-Heart.jpg]

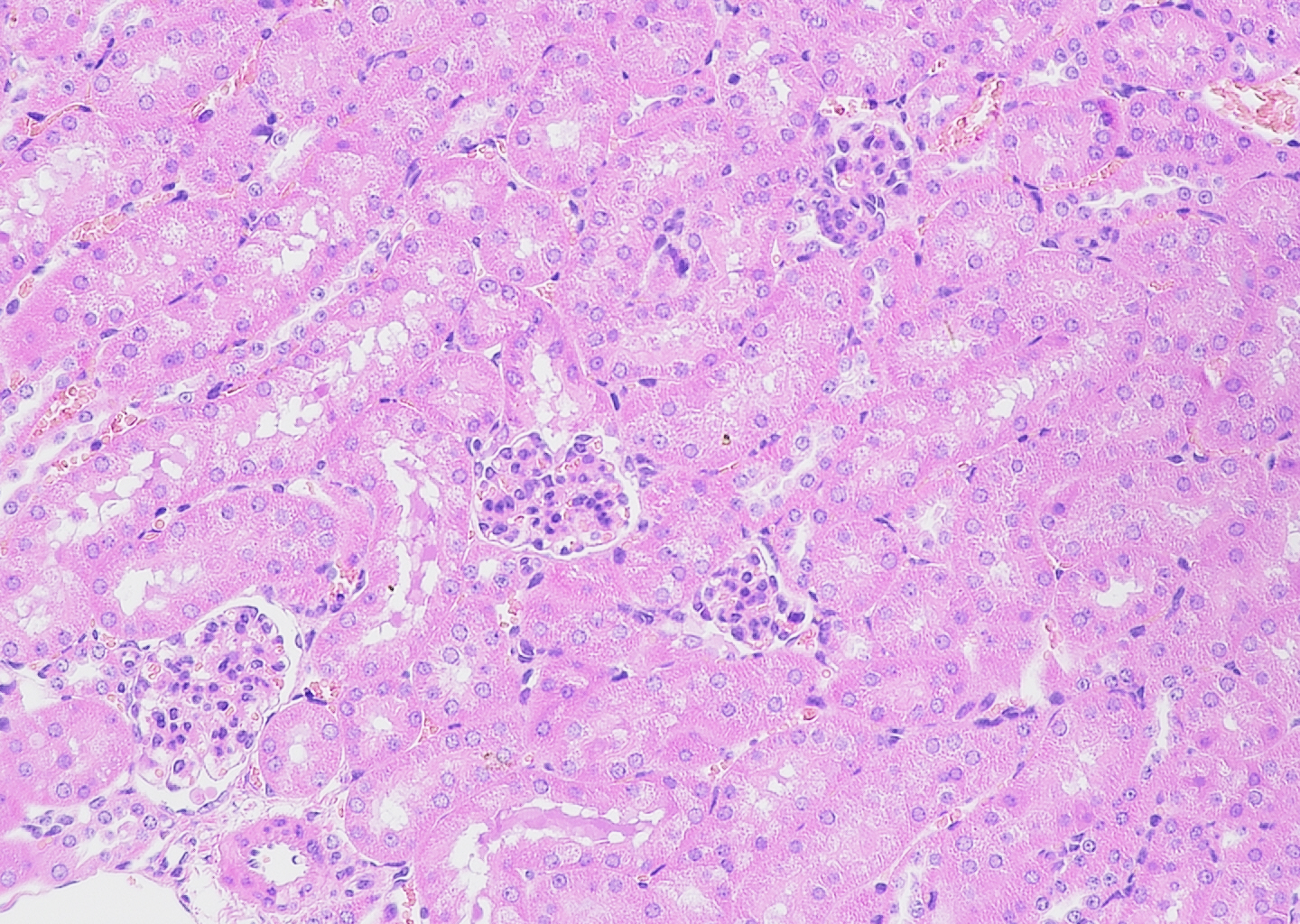

Supplement: Supplementary file 1 [file toxins-18-00278-s001.zip › Figure S7. Original full-size histopathological images of vital organs (heart, liver, spleen, lung, kidney) corresponding to Figure 11B/EGCG-Kidney.jpg]

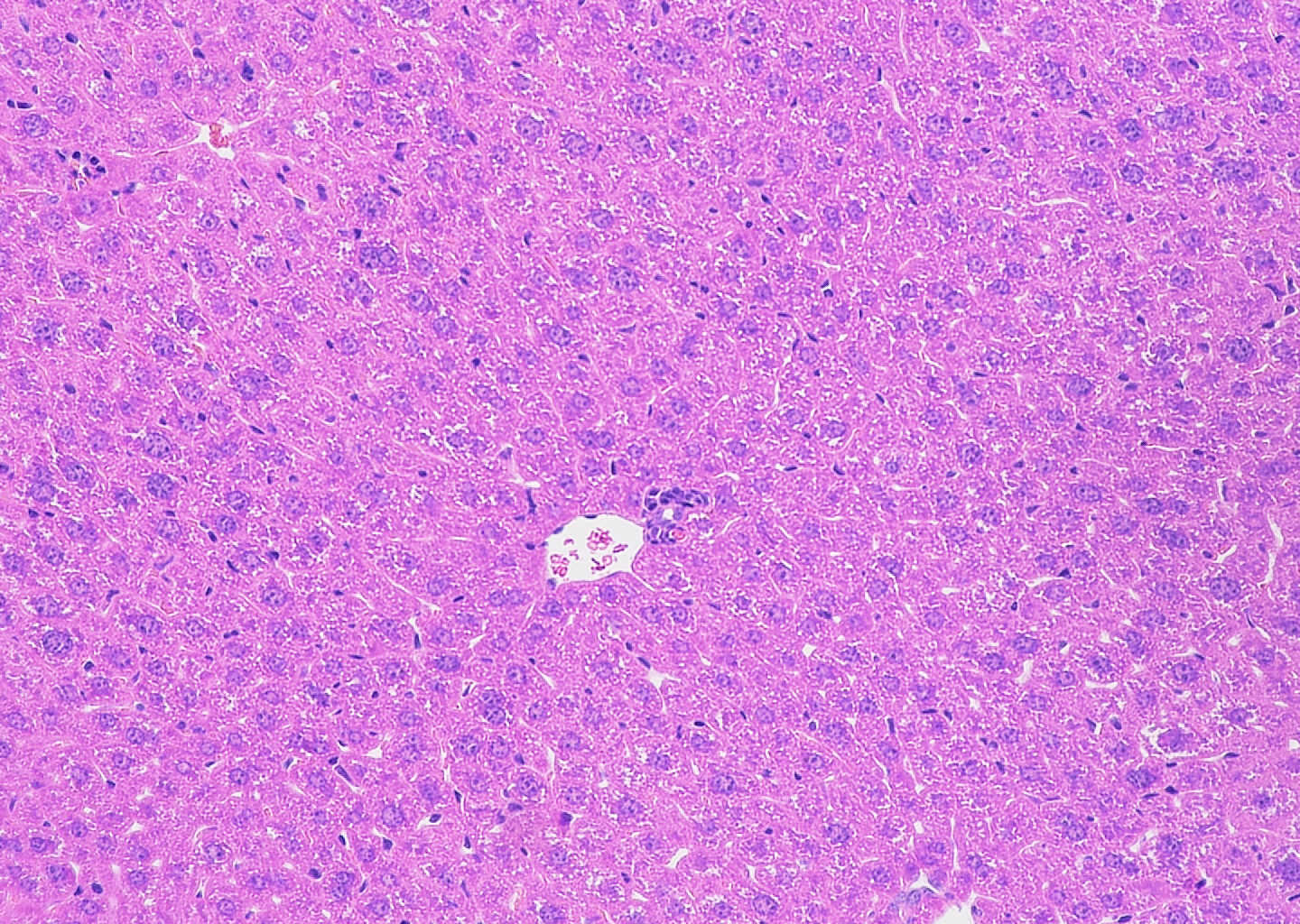

Supplement: Supplementary file 1 [file toxins-18-00278-s001.zip › Figure S7. Original full-size histopathological images of vital organs (heart, liver, spleen, lung, kidney) corresponding to Figure 11B/EGCG-Liver.jpg]

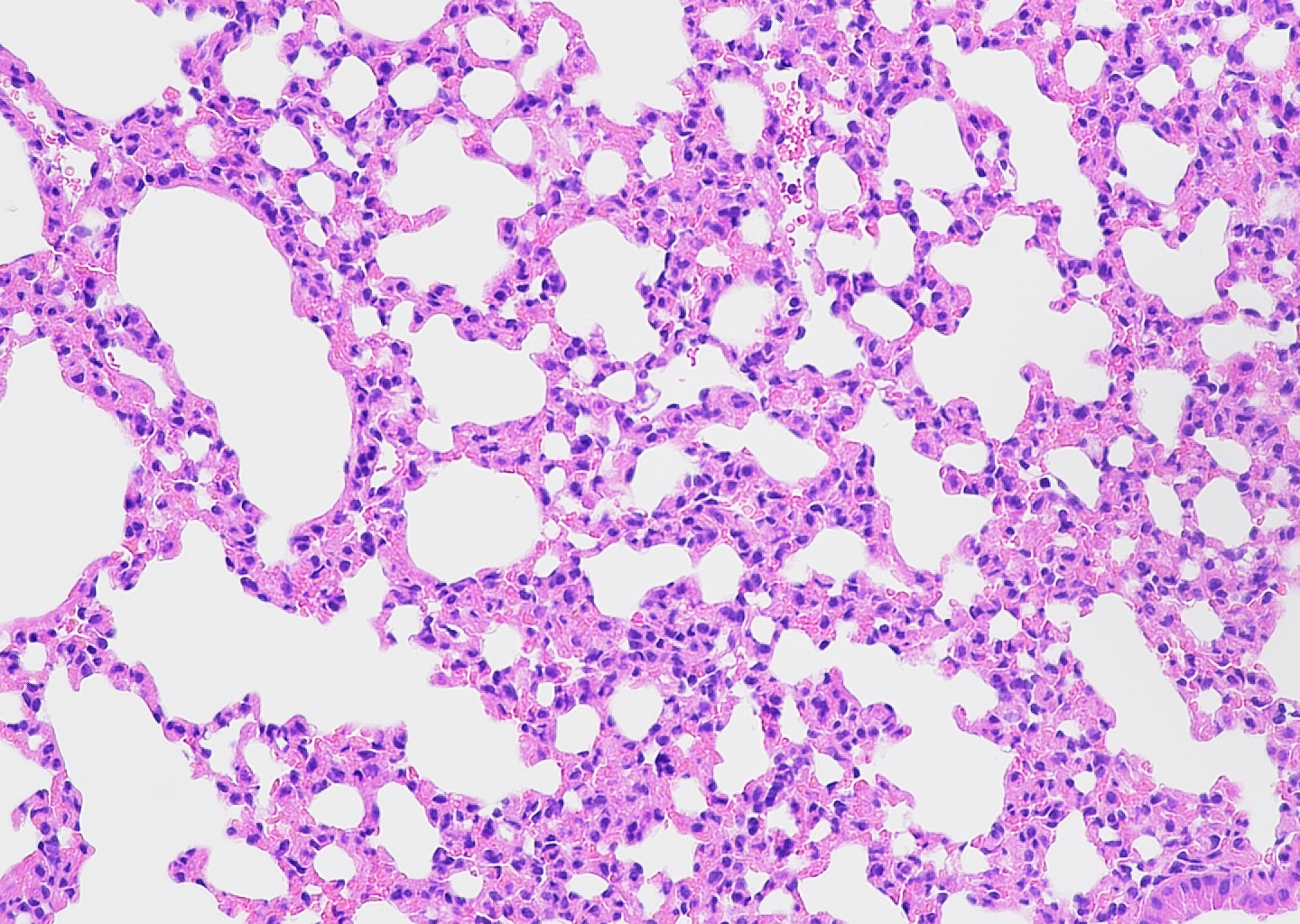

Supplement: Supplementary file 1 [file toxins-18-00278-s001.zip › Figure S7. Original full-size histopathological images of vital organs (heart, liver, spleen, lung, kidney) corresponding to Figure 11B/EGCG-Lung.jpg]

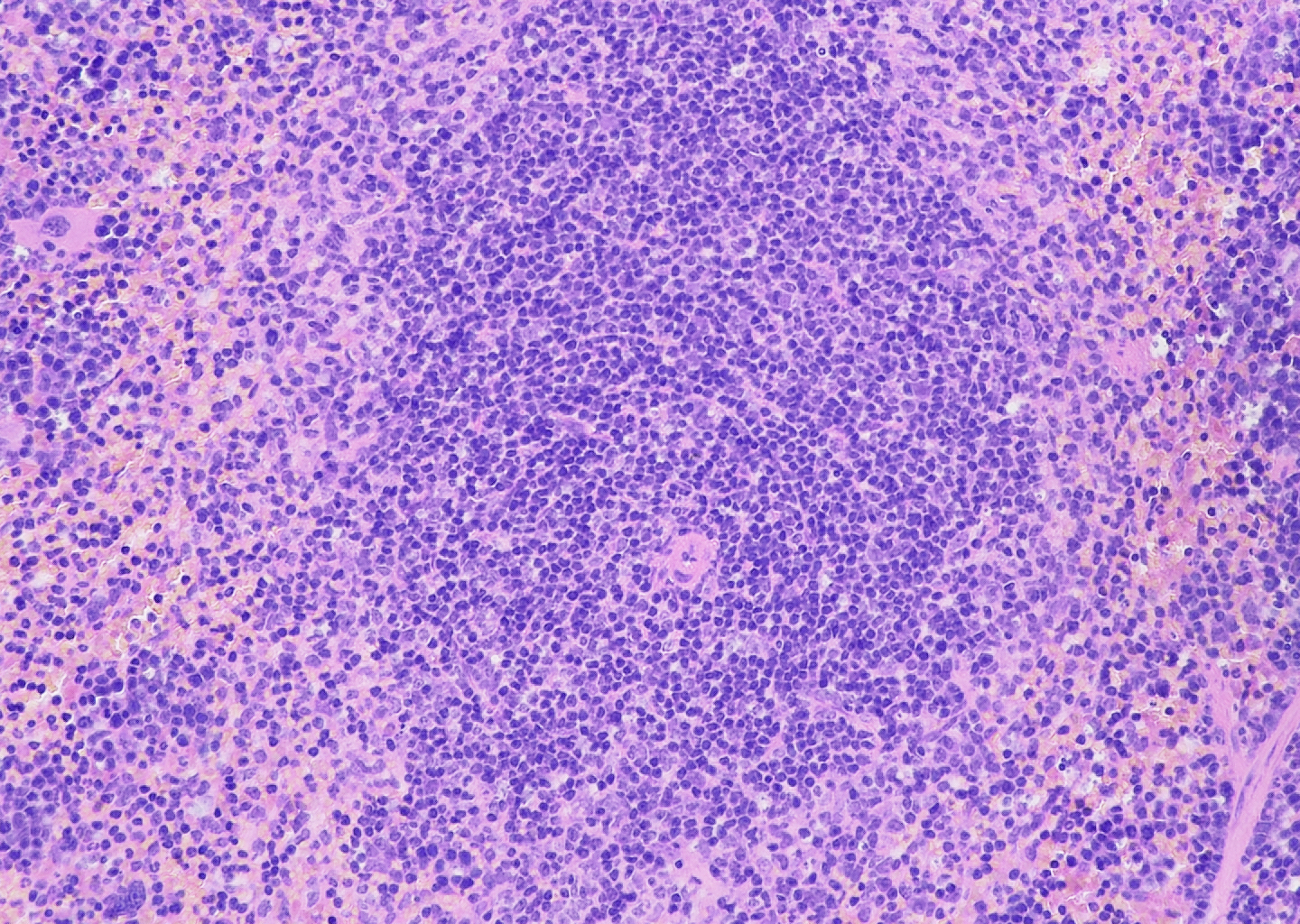

Supplement: Supplementary file 1 [file toxins-18-00278-s001.zip › Figure S7. Original full-size histopathological images of vital organs (heart, liver, spleen, lung, kidney) corresponding to Figure 11B/EGCG-Spleen.jpg]

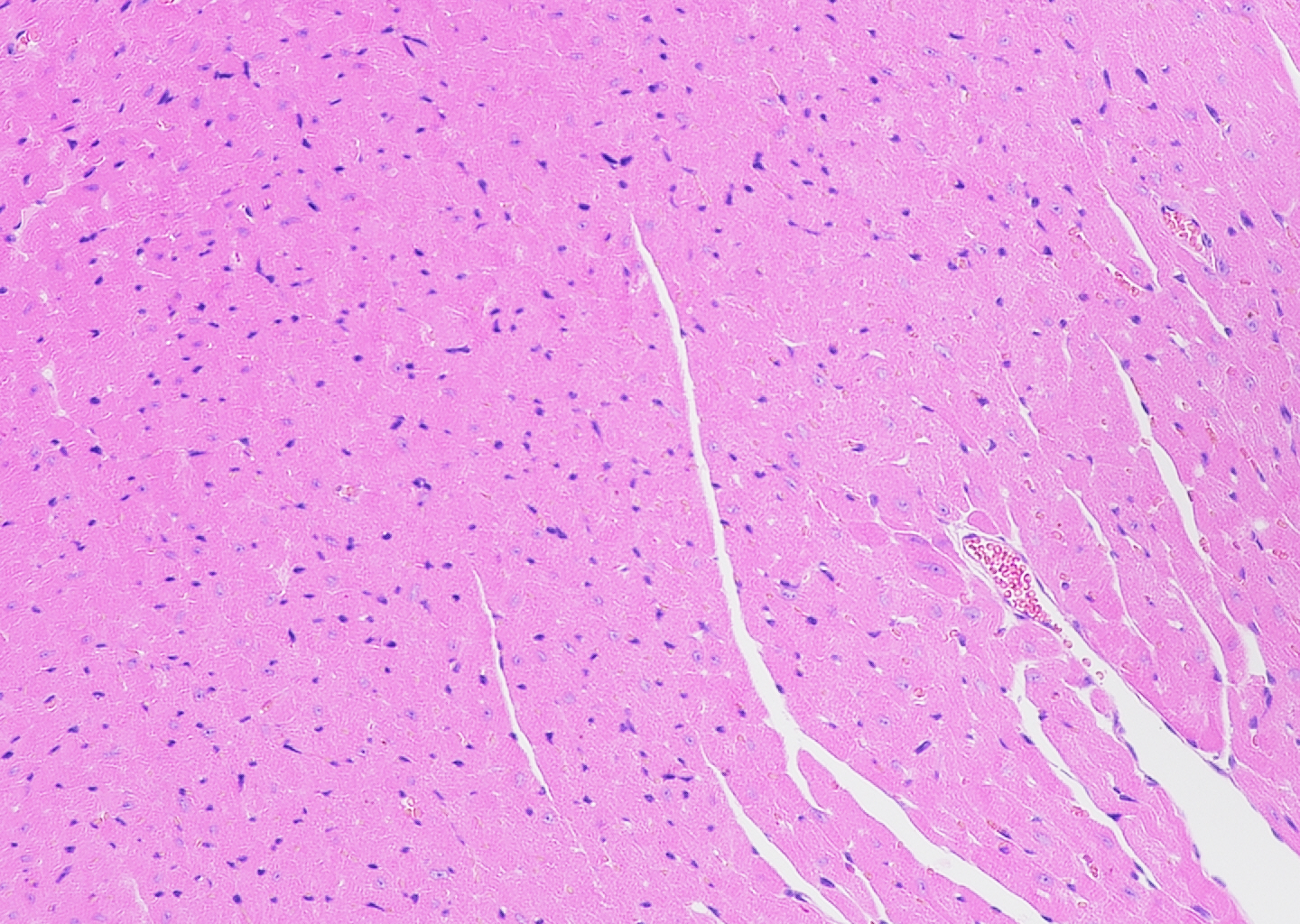

Supplement: Supplementary file 1 [file toxins-18-00278-s001.zip › Figure S7. Original full-size histopathological images of vital organs (heart, liver, spleen, lung, kidney) corresponding to Figure 11B/HTC-Heart.jpg]

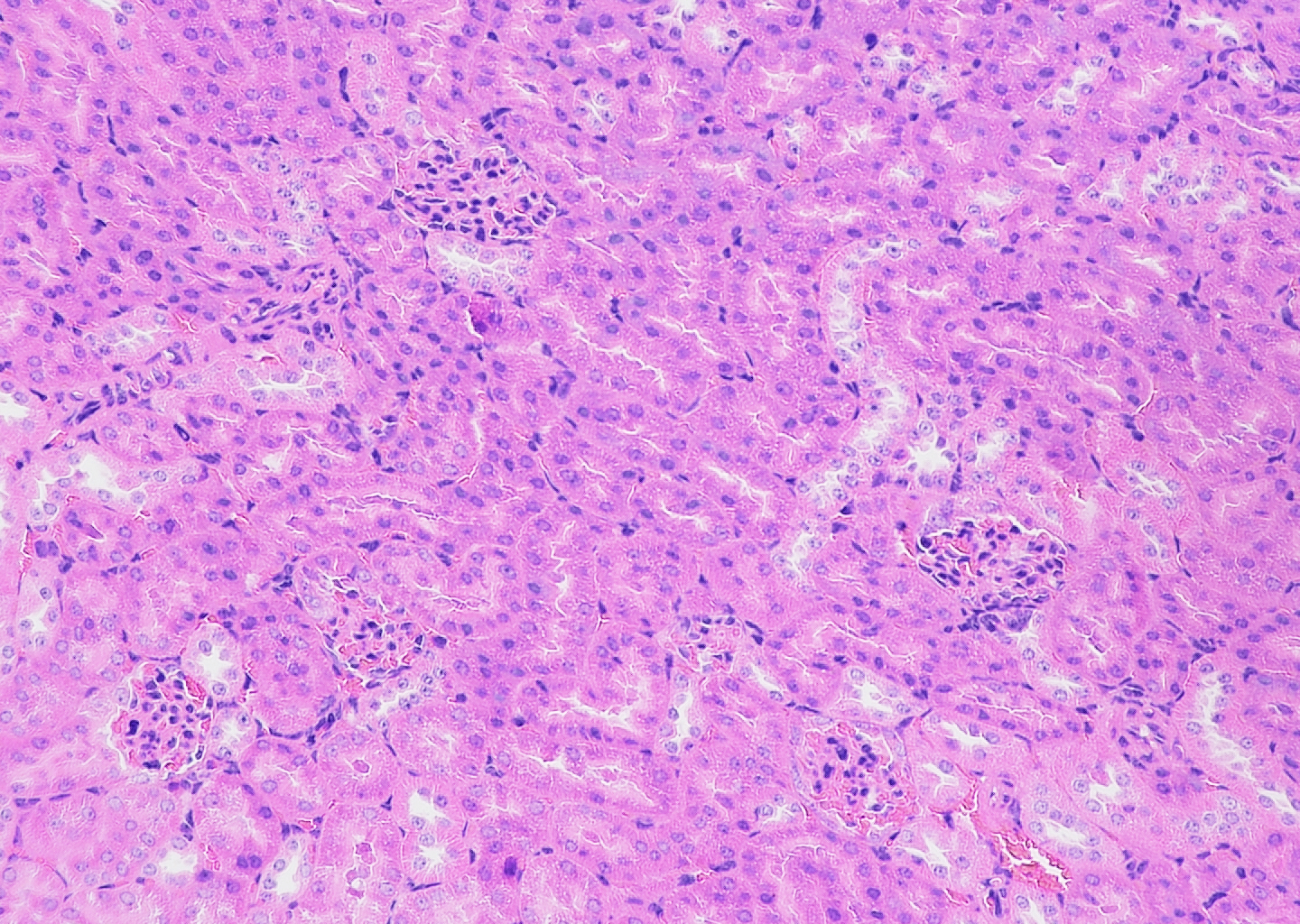

Supplement: Supplementary file 1 [file toxins-18-00278-s001.zip › Figure S7. Original full-size histopathological images of vital organs (heart, liver, spleen, lung, kidney) corresponding to Figure 11B/HTC-Kidney.jpg]

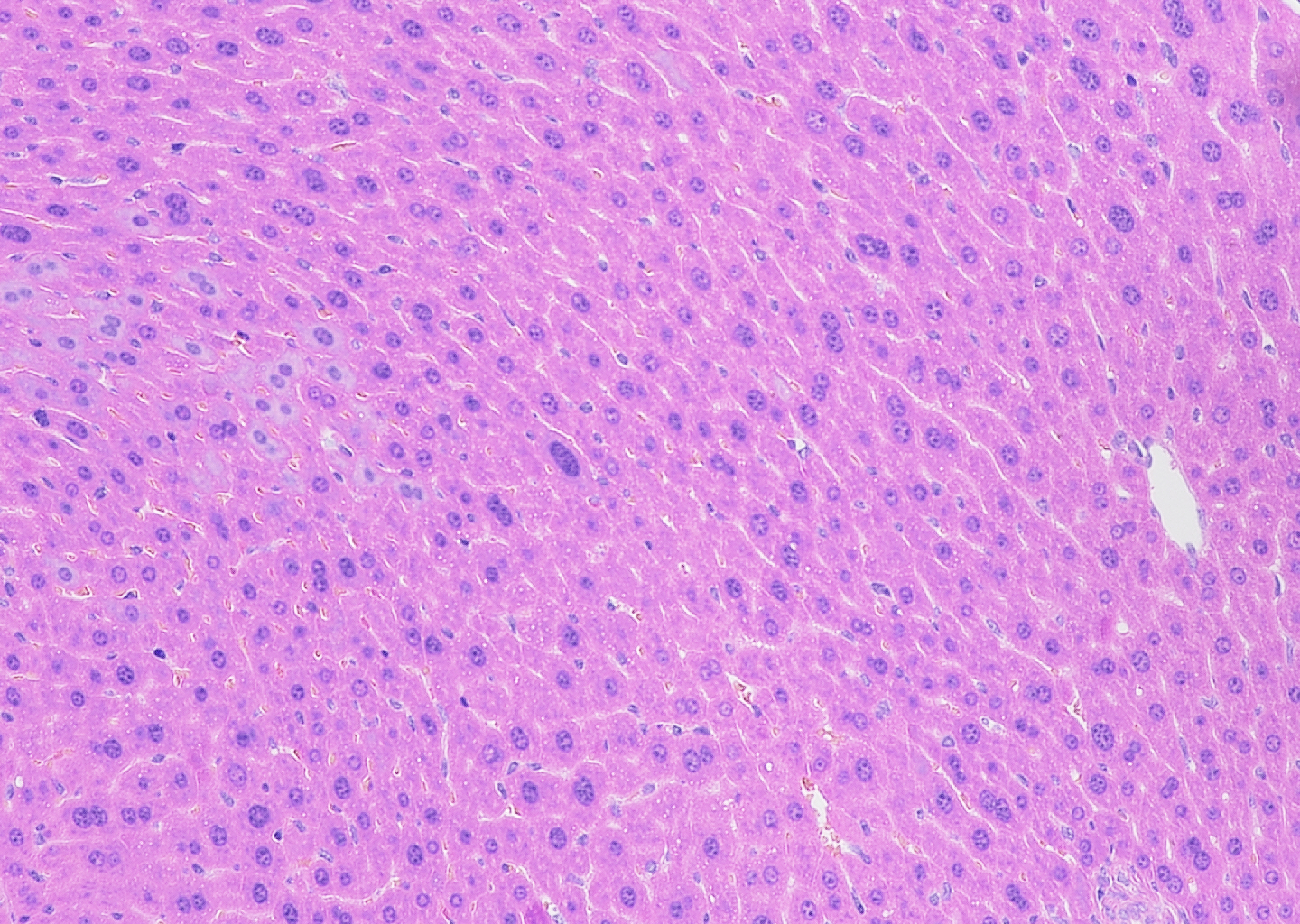

Supplement: Supplementary file 1 [file toxins-18-00278-s001.zip › Figure S7. Original full-size histopathological images of vital organs (heart, liver, spleen, lung, kidney) corresponding to Figure 11B/HTC-liver.jpg]

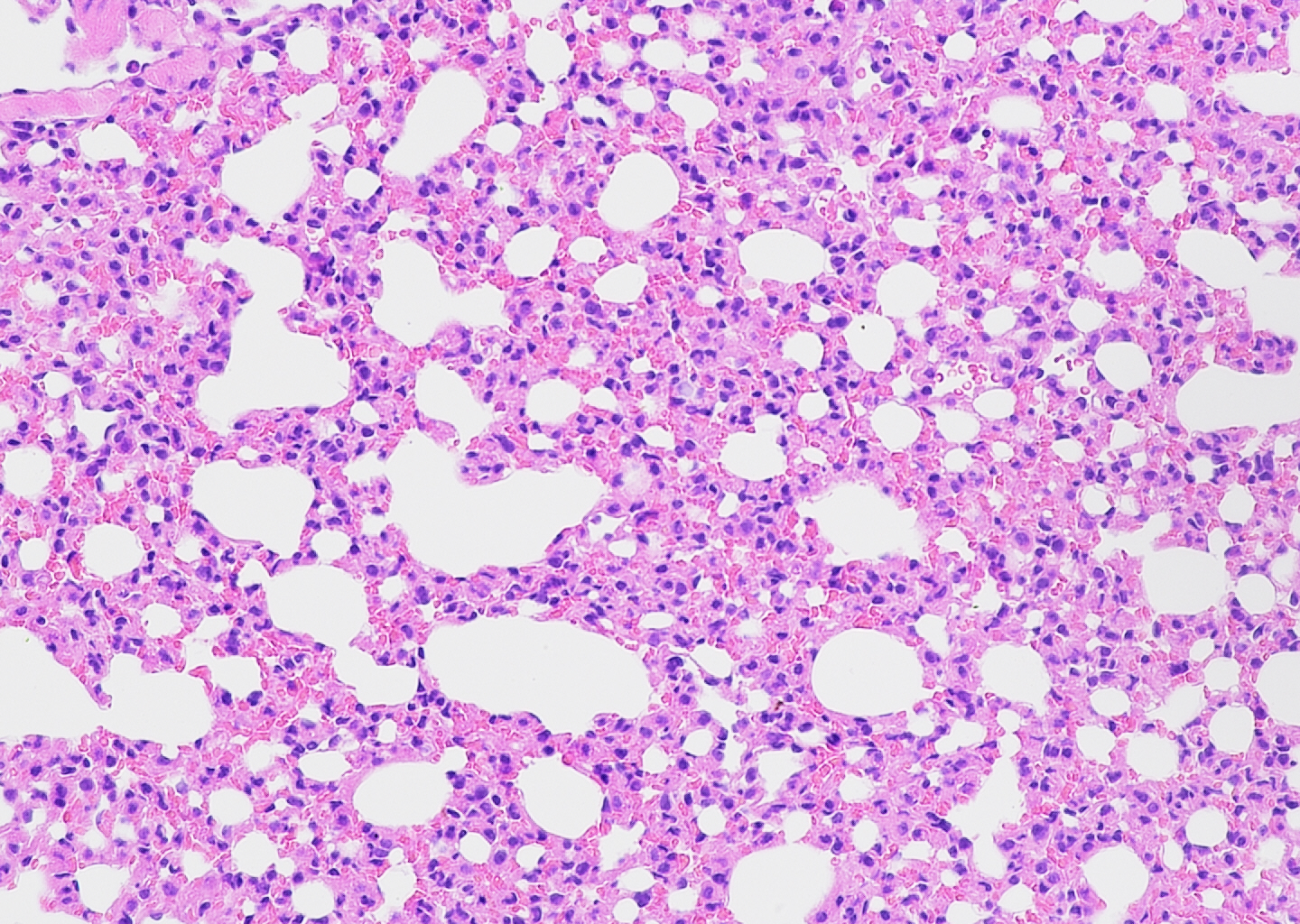

Supplement: Supplementary file 1 [file toxins-18-00278-s001.zip › Figure S7. Original full-size histopathological images of vital organs (heart, liver, spleen, lung, kidney) corresponding to Figure 11B/HTC-Lung.jpg]

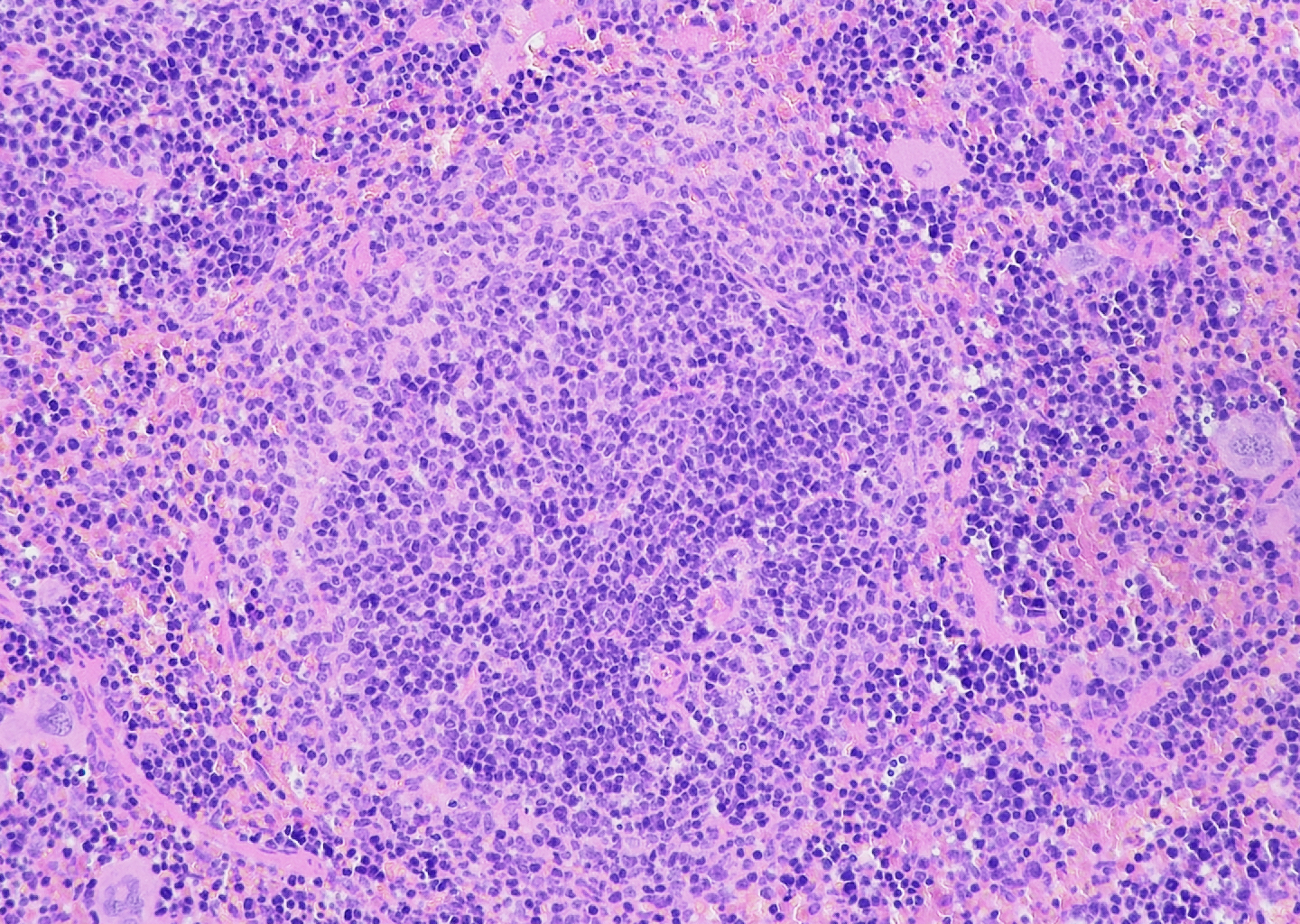

Supplement: Supplementary file 1 [file toxins-18-00278-s001.zip › Figure S7. Original full-size histopathological images of vital organs (heart, liver, spleen, lung, kidney) corresponding to Figure 11B/HTC-Spleen.jpg]

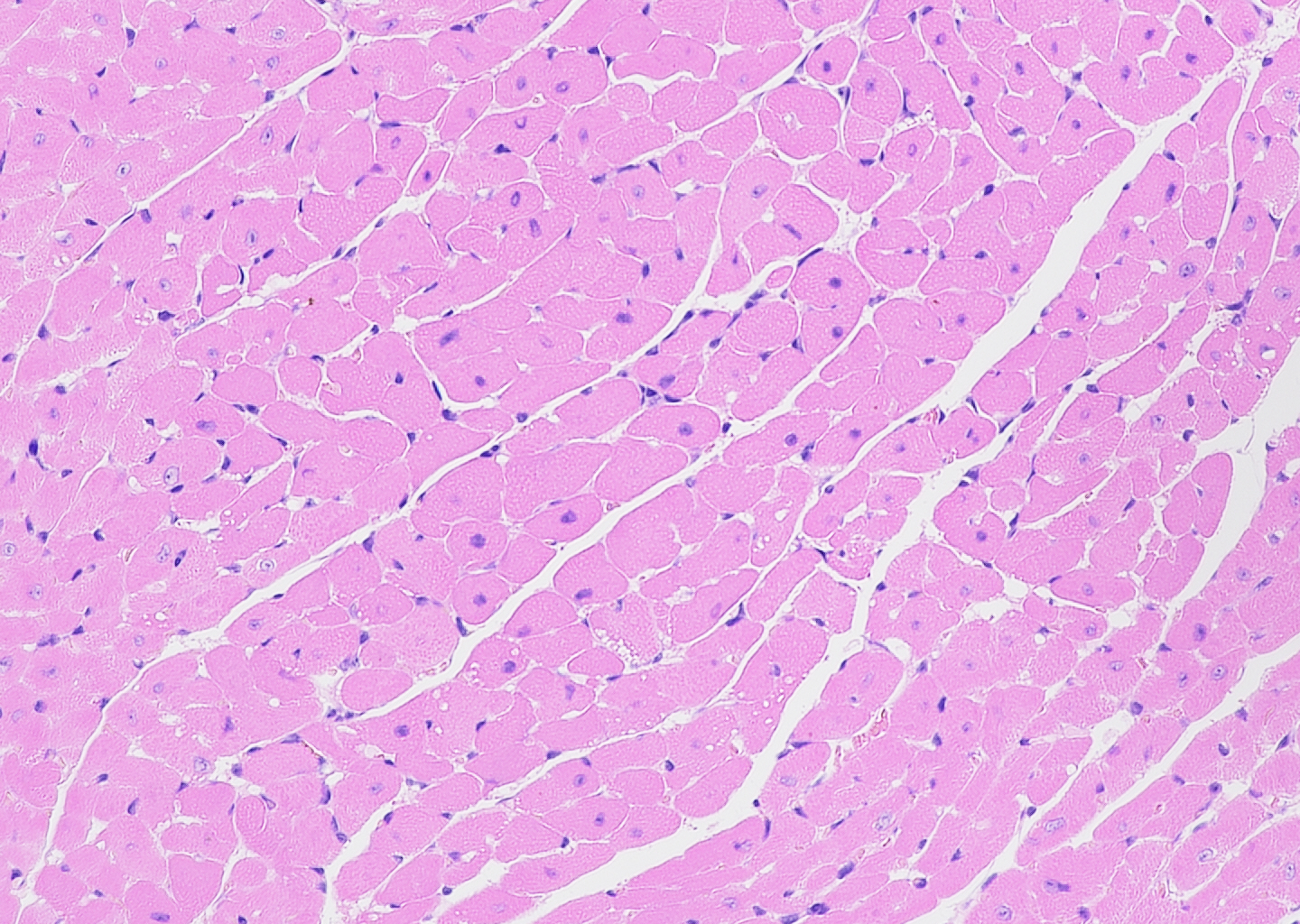

Supplement: Supplementary file 1 [file toxins-18-00278-s001.zip › Figure S7. Original full-size histopathological images of vital organs (heart, liver, spleen, lung, kidney) corresponding to Figure 11B/PBS-Heart.jpg]

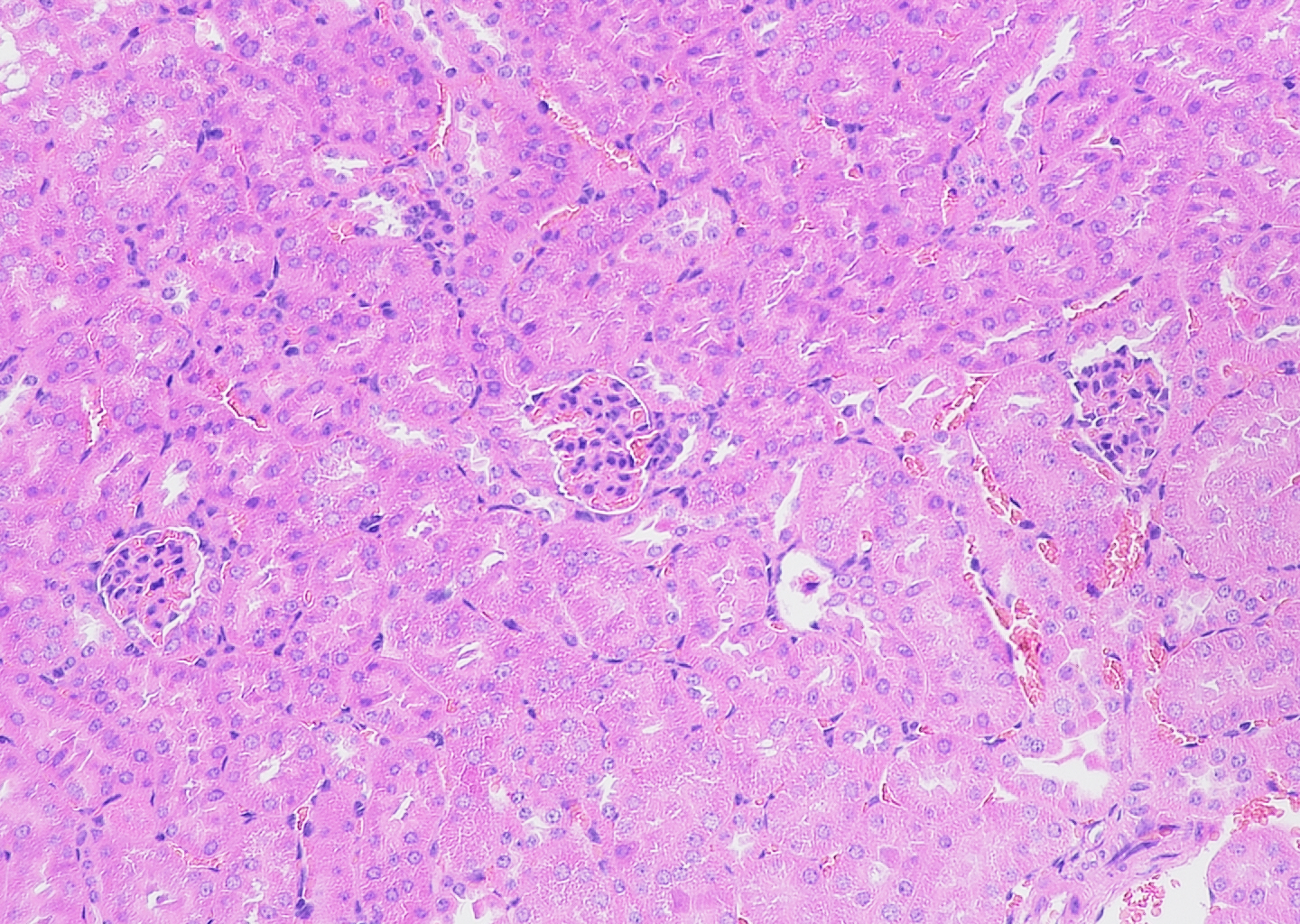

Supplement: Supplementary file 1 [file toxins-18-00278-s001.zip › Figure S7. Original full-size histopathological images of vital organs (heart, liver, spleen, lung, kidney) corresponding to Figure 11B/PBS-Kidney.jpg]

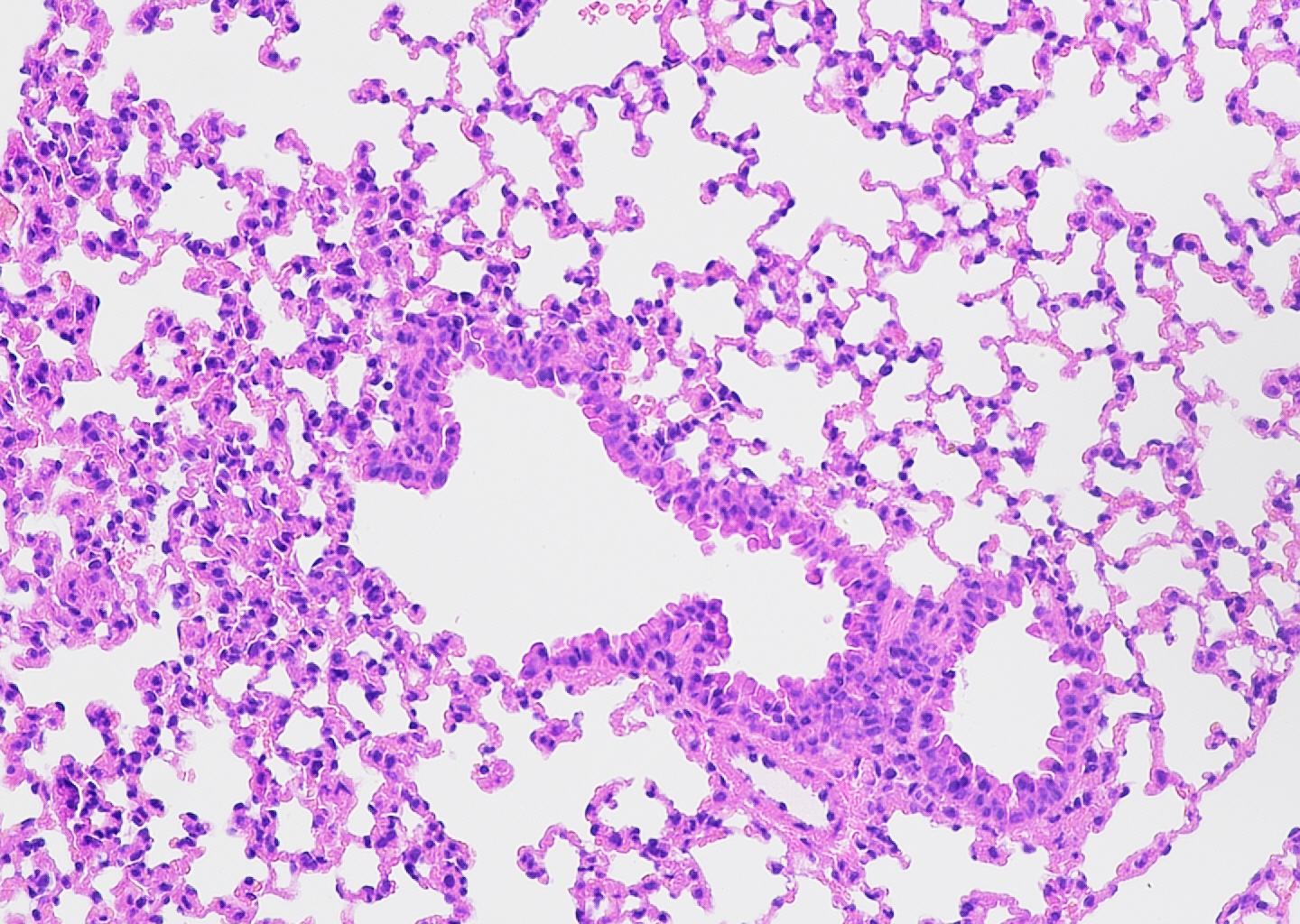

Supplement: Supplementary file 1 [file toxins-18-00278-s001.zip › Figure S7. Original full-size histopathological images of vital organs (heart, liver, spleen, lung, kidney) corresponding to Figure 11B/PBS-Liver.jpg]

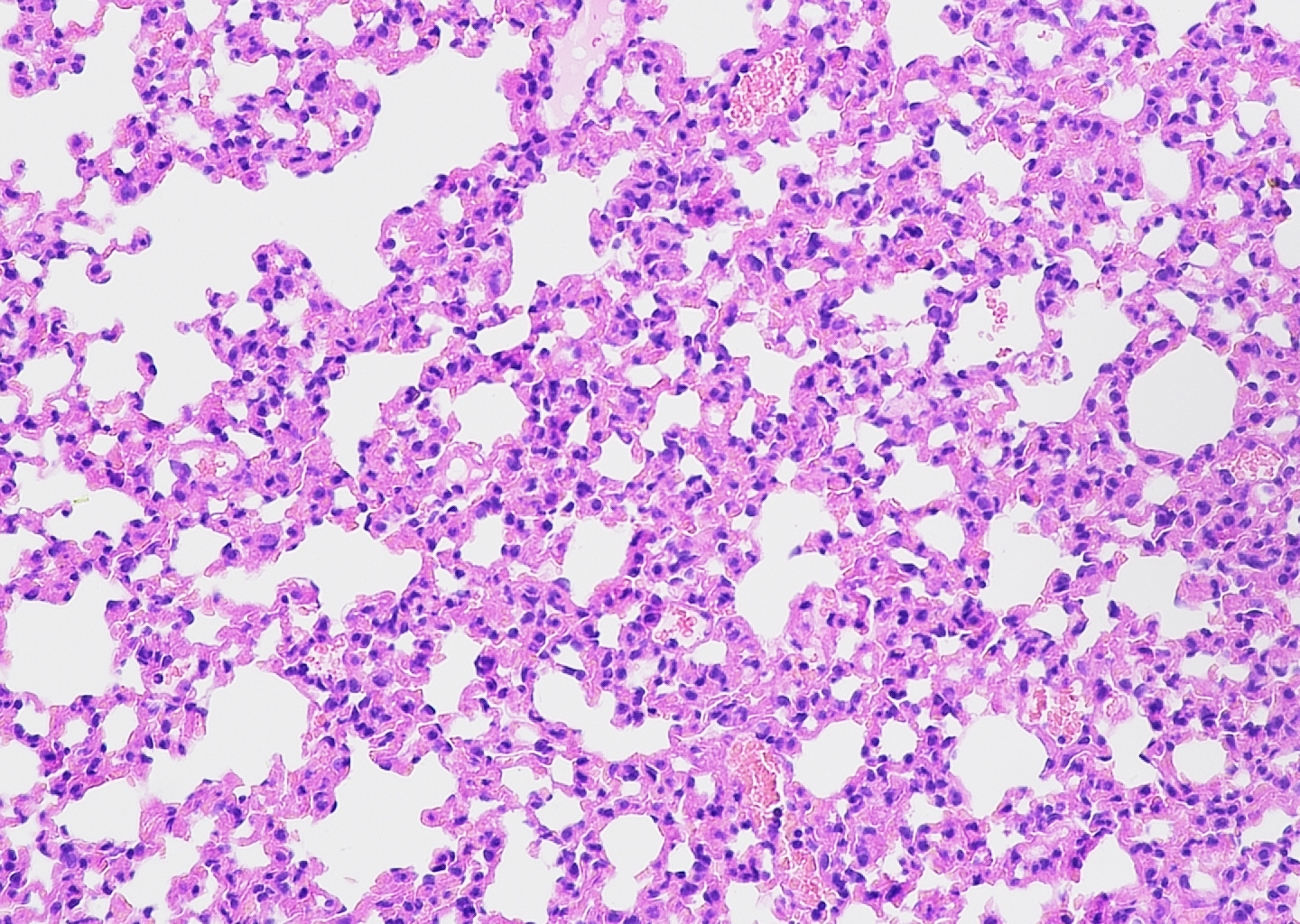

Supplement: Supplementary file 1 [file toxins-18-00278-s001.zip › Figure S7. Original full-size histopathological images of vital organs (heart, liver, spleen, lung, kidney) corresponding to Figure 11B/PBS-Lung.jpg]

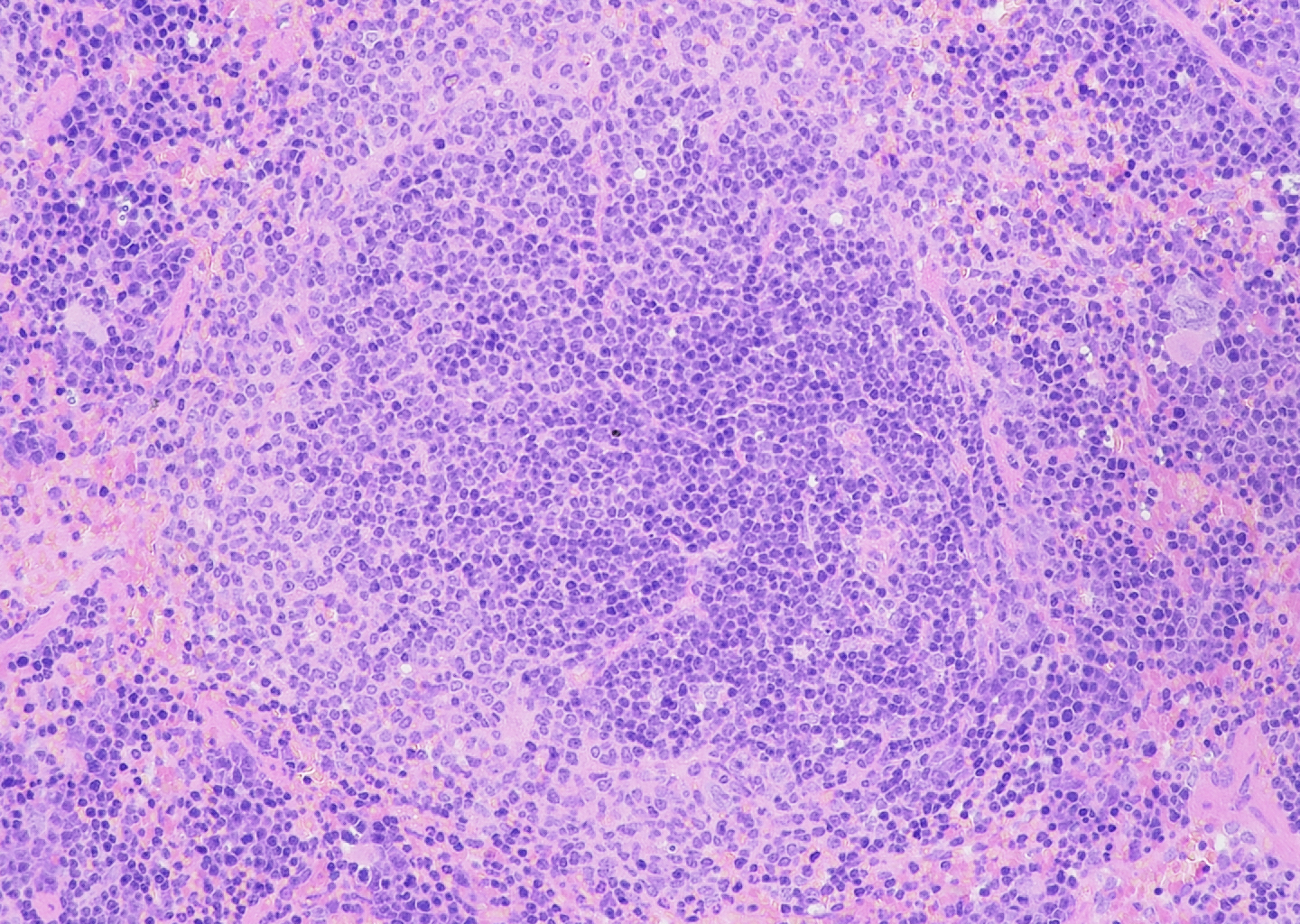

Supplement: Supplementary file 1 [file toxins-18-00278-s001.zip › Figure S7. Original full-size histopathological images of vital organs (heart, liver, spleen, lung, kidney) corresponding to Figure 11B/PBS-Spleen.jpg]

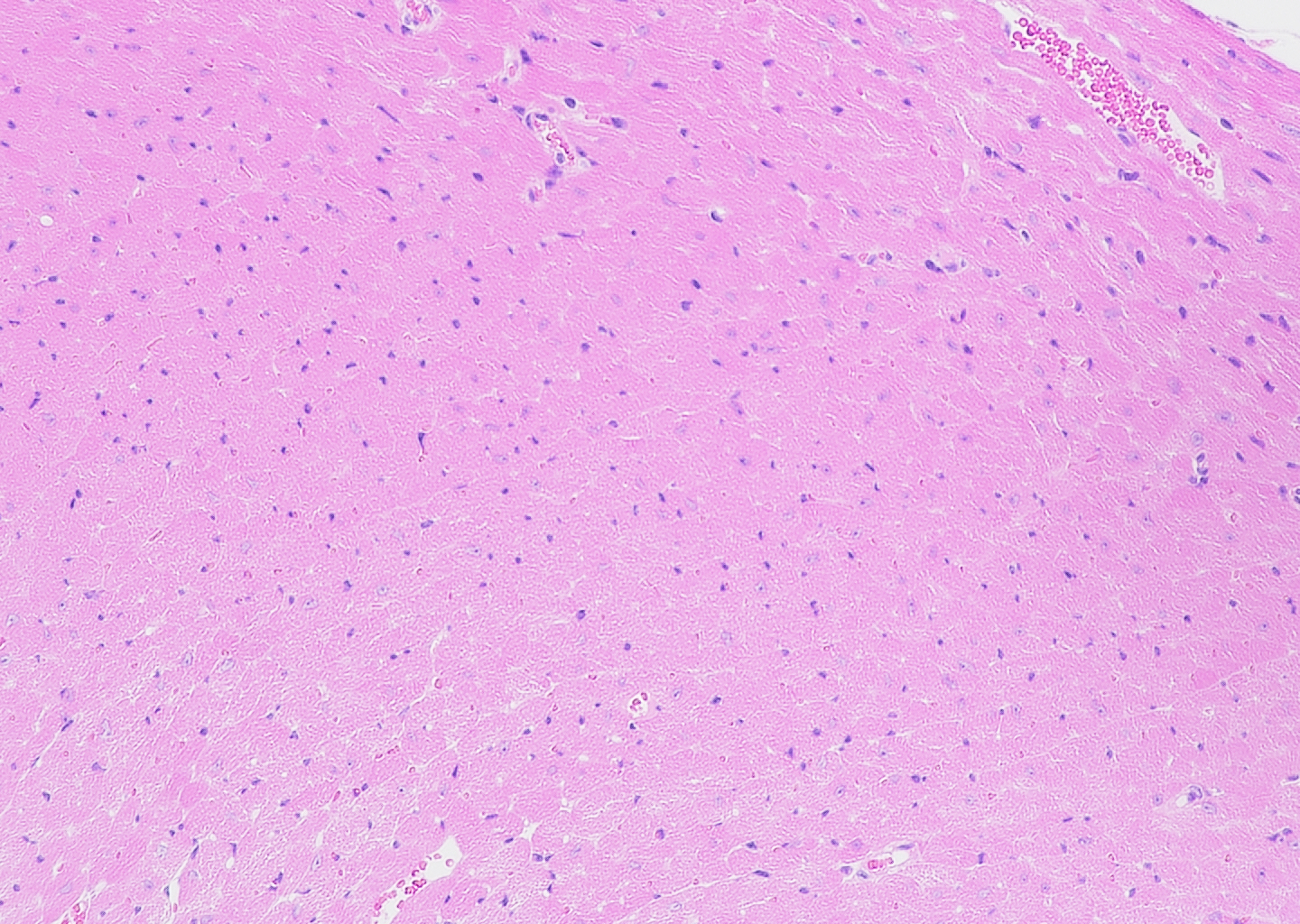

Supplement: Supplementary file 1 [file toxins-18-00278-s001.zip › Figure S7. Original full-size histopathological images of vital organs (heart, liver, spleen, lung, kidney) corresponding to Figure 11B/PEG-EGCG-Heart.jpg]

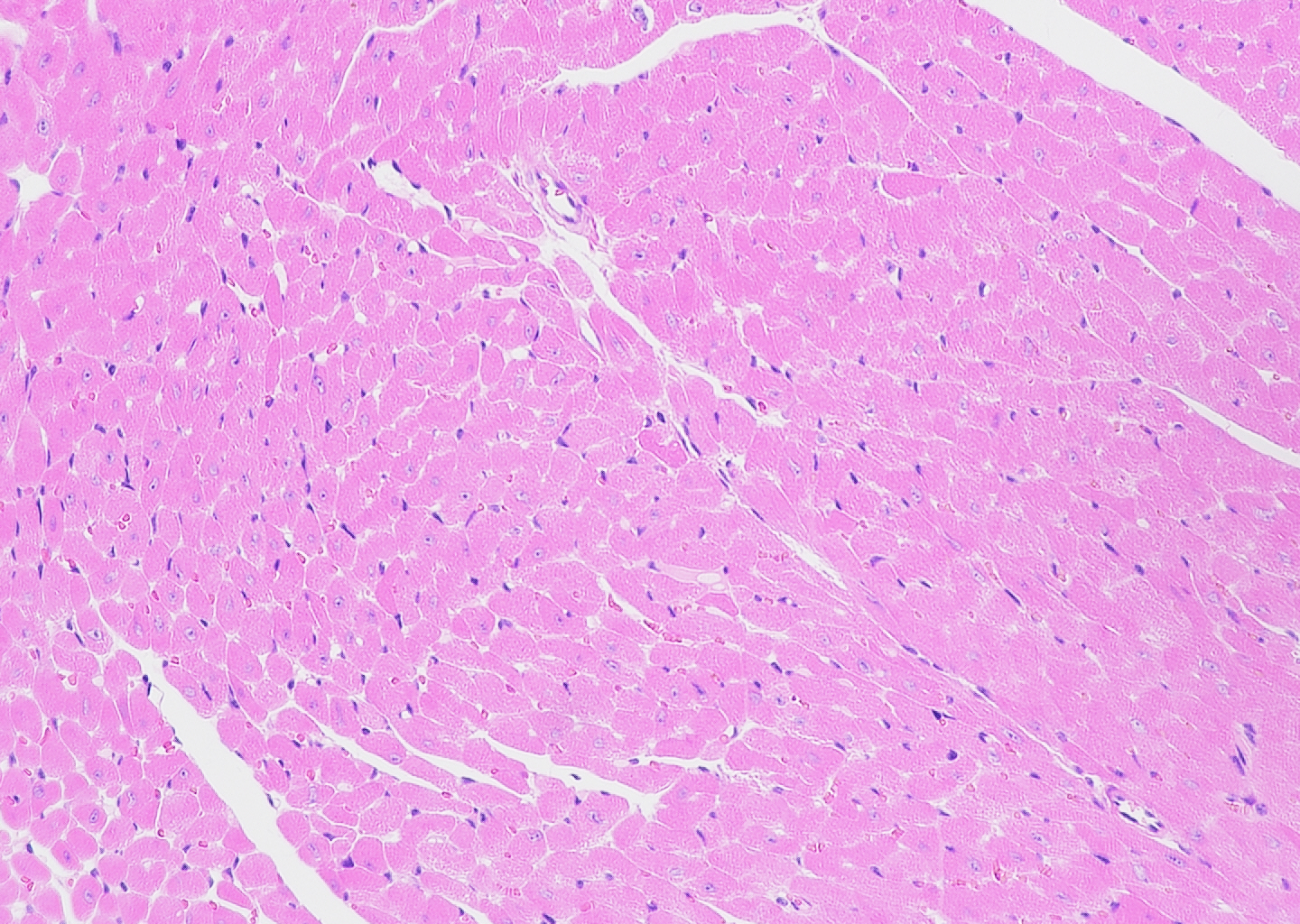

Supplement: Supplementary file 1 [file toxins-18-00278-s001.zip › Figure S7. Original full-size histopathological images of vital organs (heart, liver, spleen, lung, kidney) corresponding to Figure 11B/PEG-EGCG-HTC-Heart.jpg]

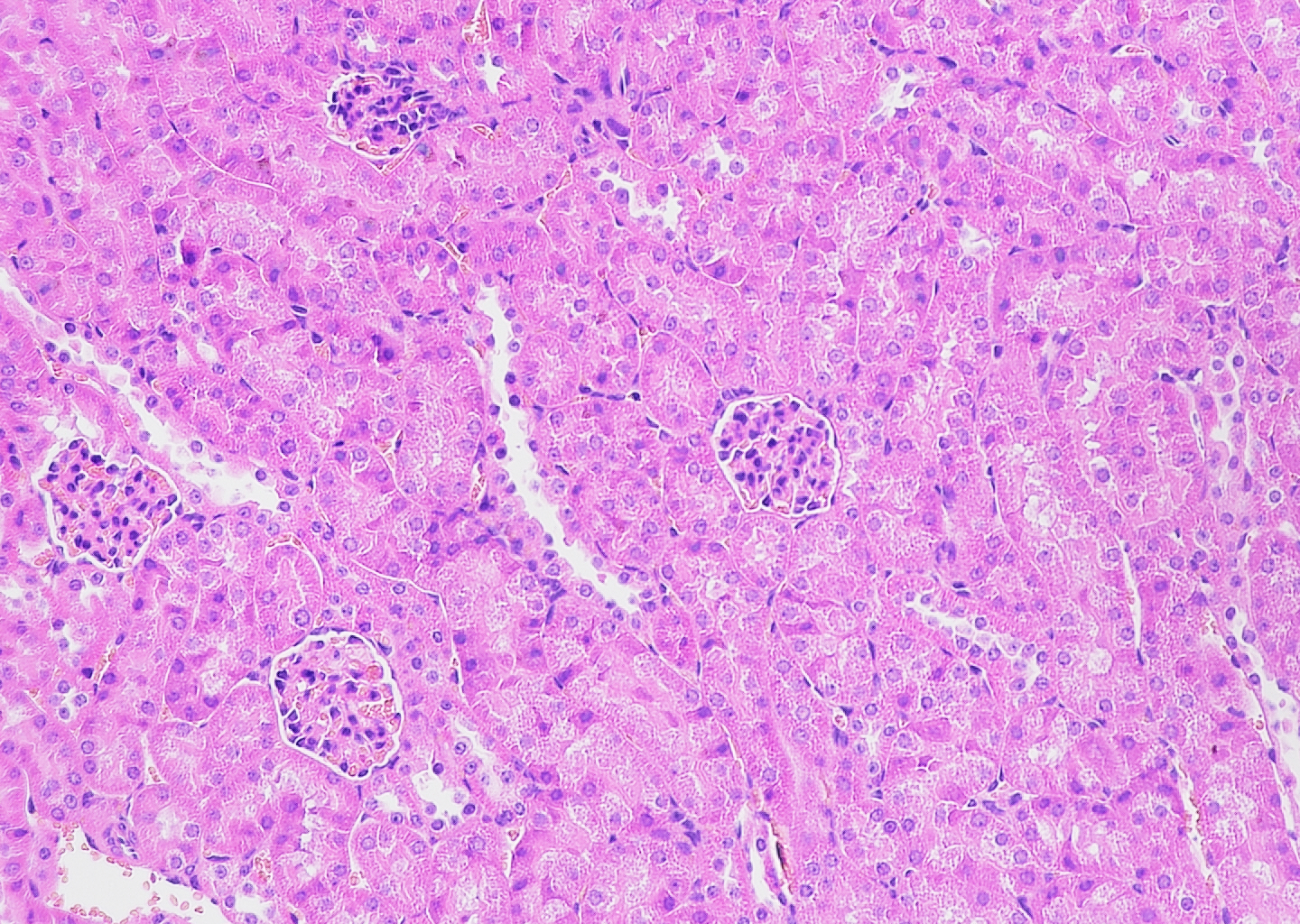

Supplement: Supplementary file 1 [file toxins-18-00278-s001.zip › Figure S7. Original full-size histopathological images of vital organs (heart, liver, spleen, lung, kidney) corresponding to Figure 11B/PEG-EGCG-HTC-Kidney.jpg]

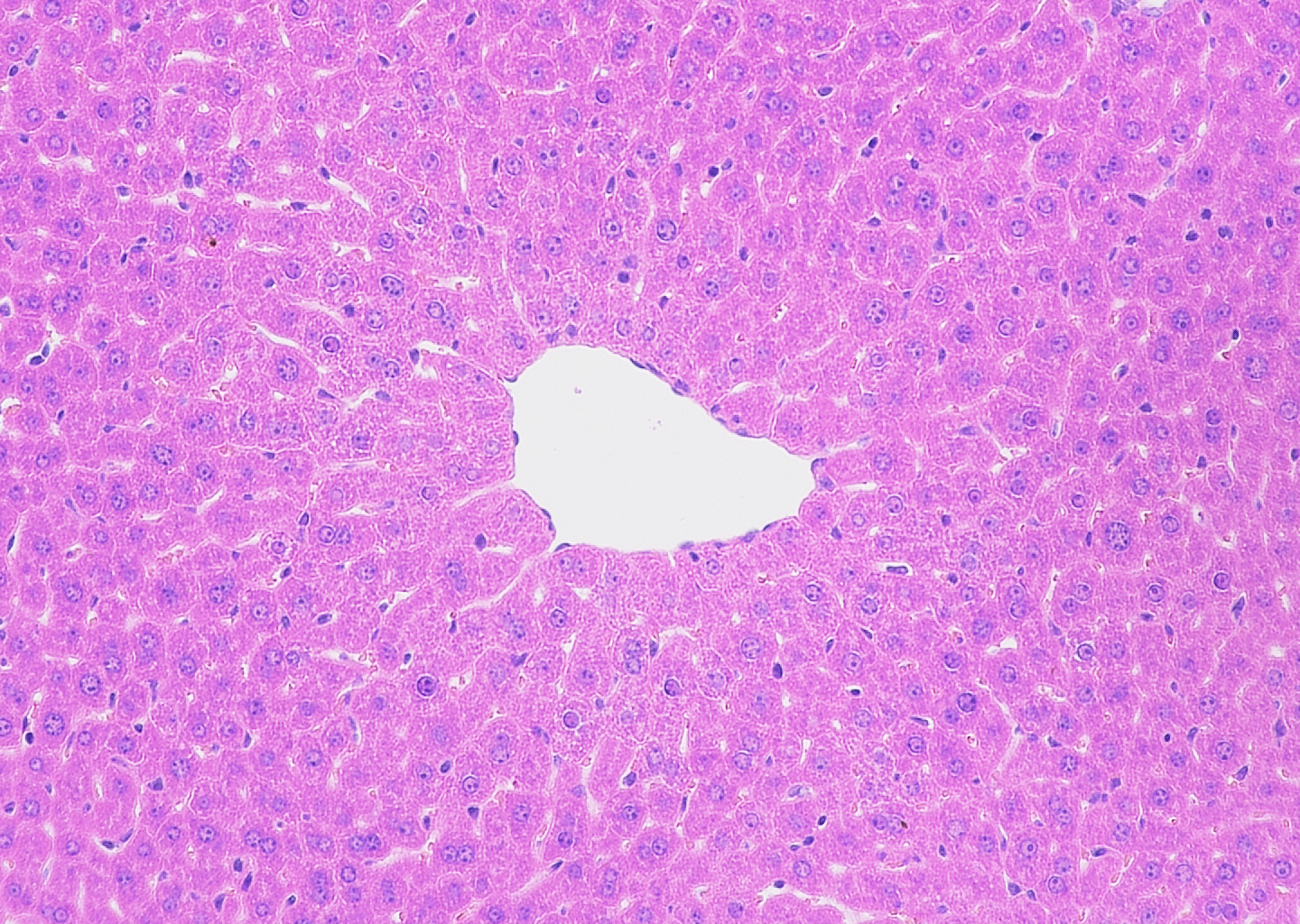

Supplement: Supplementary file 1 [file toxins-18-00278-s001.zip › Figure S7. Original full-size histopathological images of vital organs (heart, liver, spleen, lung, kidney) corresponding to Figure 11B/PEG-EGCG-HTC-Liver.jpg]

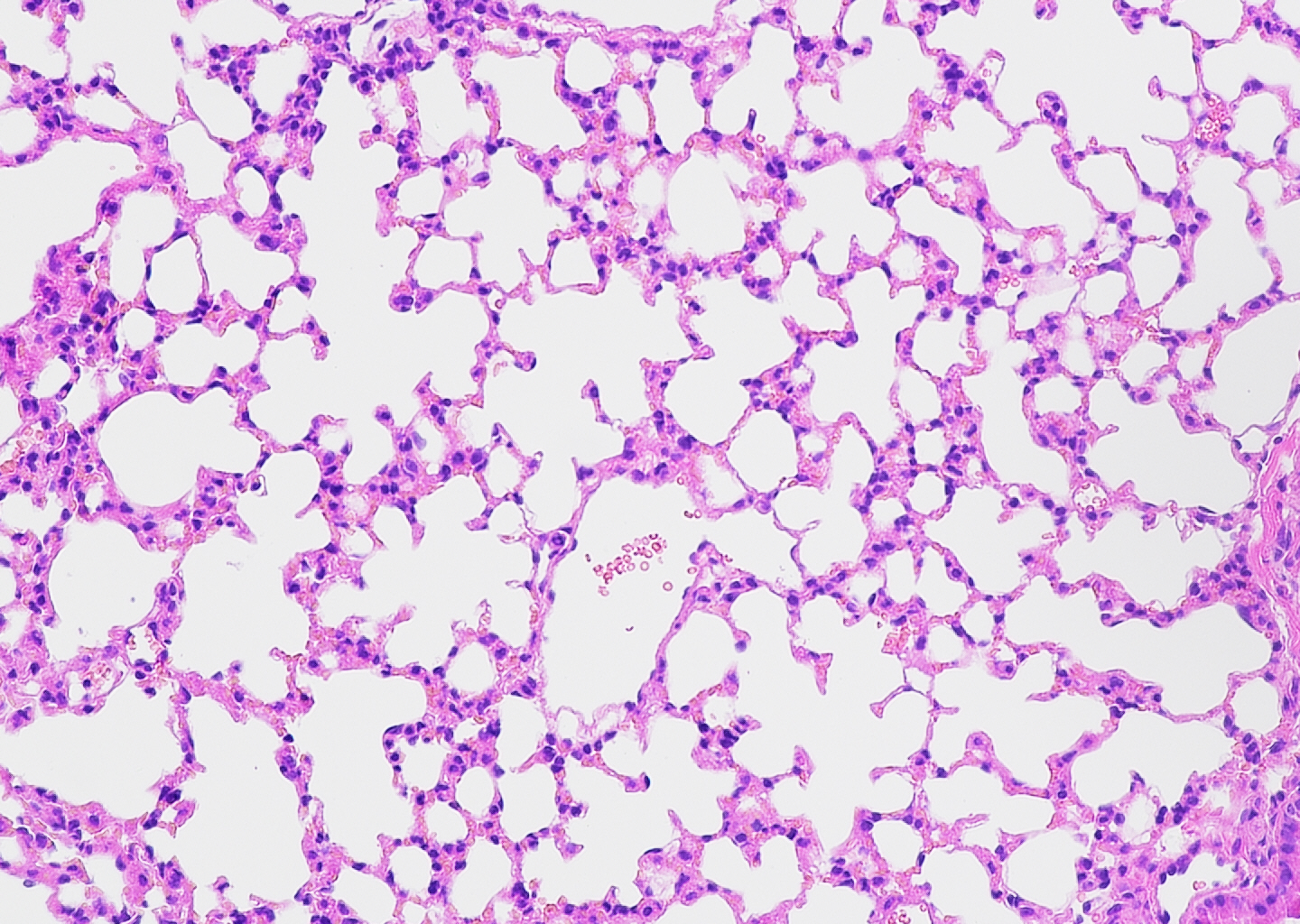

Supplement: Supplementary file 1 [file toxins-18-00278-s001.zip › Figure S7. Original full-size histopathological images of vital organs (heart, liver, spleen, lung, kidney) corresponding to Figure 11B/PEG-EGCG-HTC-Lung.jpg]

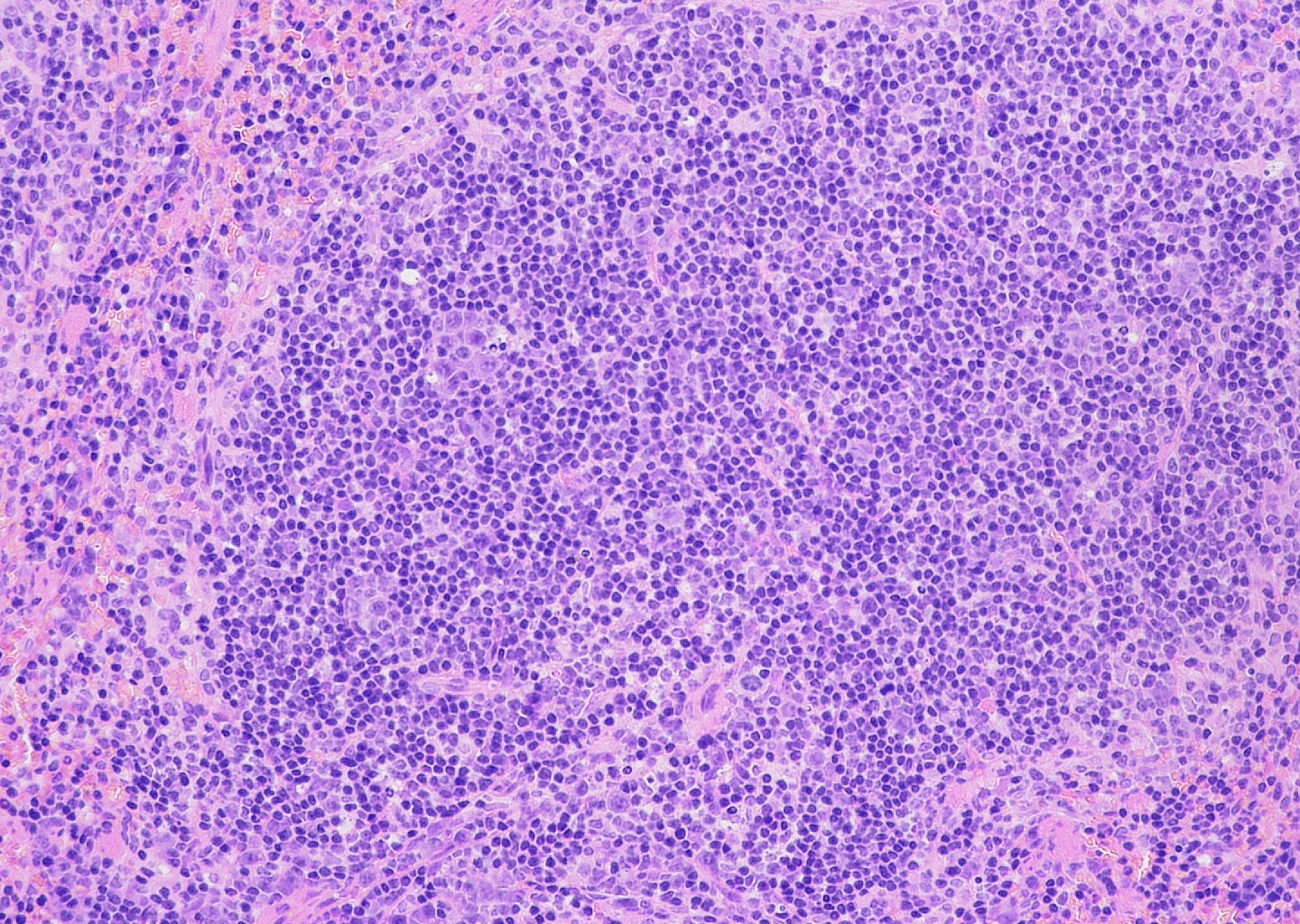

Supplement: Supplementary file 1 [file toxins-18-00278-s001.zip › Figure S7. Original full-size histopathological images of vital organs (heart, liver, spleen, lung, kidney) corresponding to Figure 11B/PEG-EGCG-HTC-Sleen.jpg]

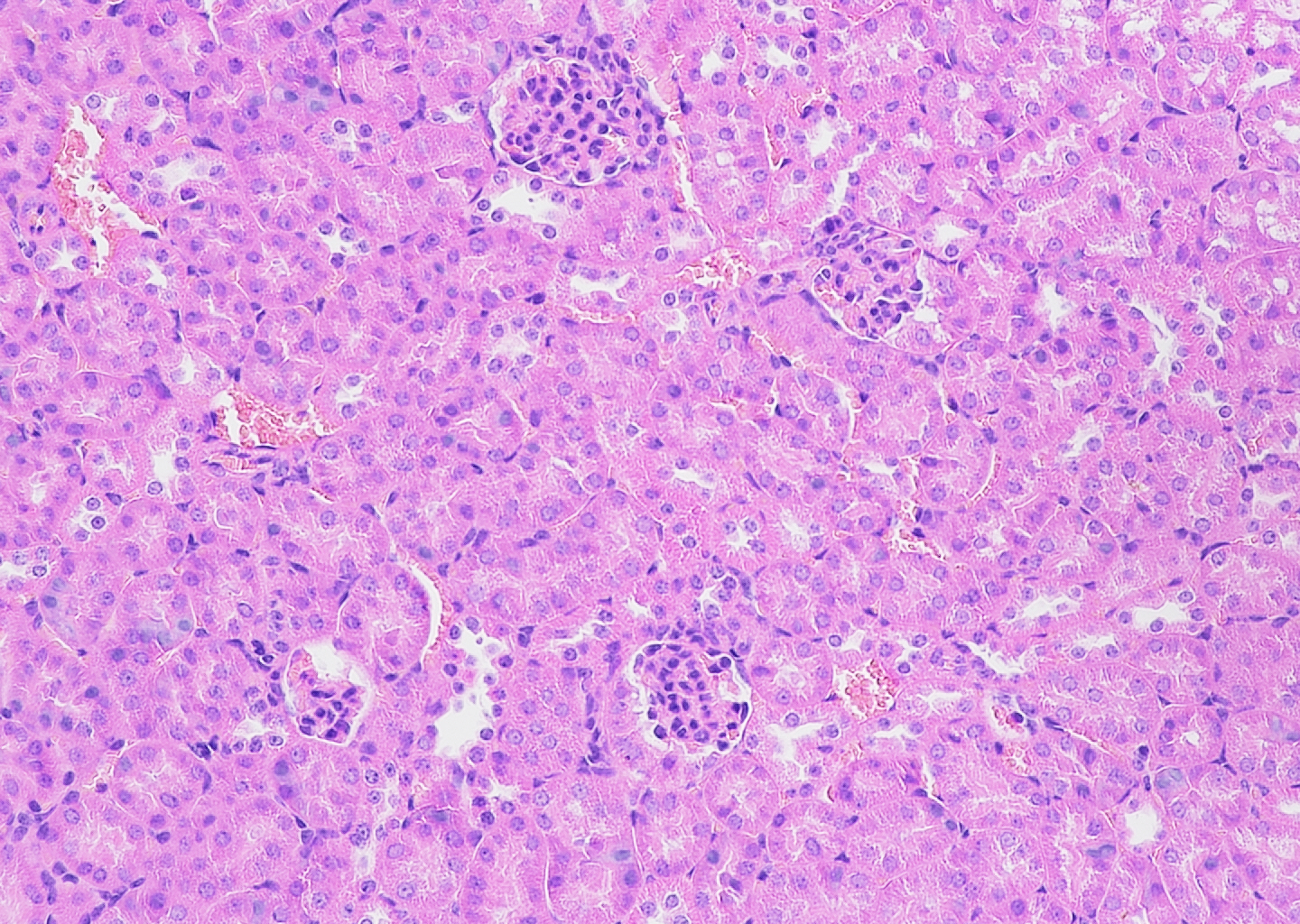

Supplement: Supplementary file 1 [file toxins-18-00278-s001.zip › Figure S7. Original full-size histopathological images of vital organs (heart, liver, spleen, lung, kidney) corresponding to Figure 11B/PEG-EGCG-Kidney.jpg]

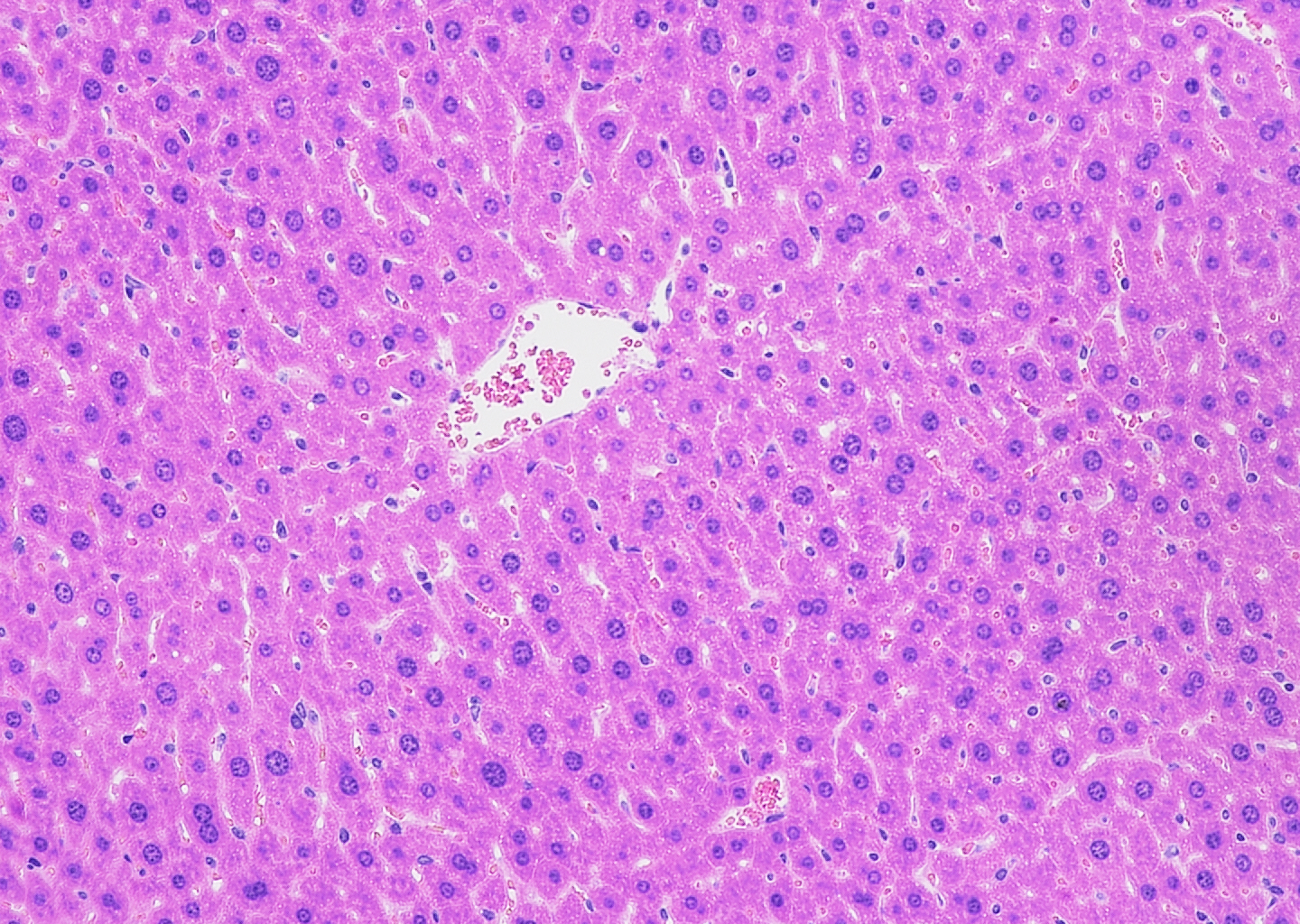

Supplement: Supplementary file 1 [file toxins-18-00278-s001.zip › Figure S7. Original full-size histopathological images of vital organs (heart, liver, spleen, lung, kidney) corresponding to Figure 11B/PEG-EGCG-liver.jpg]

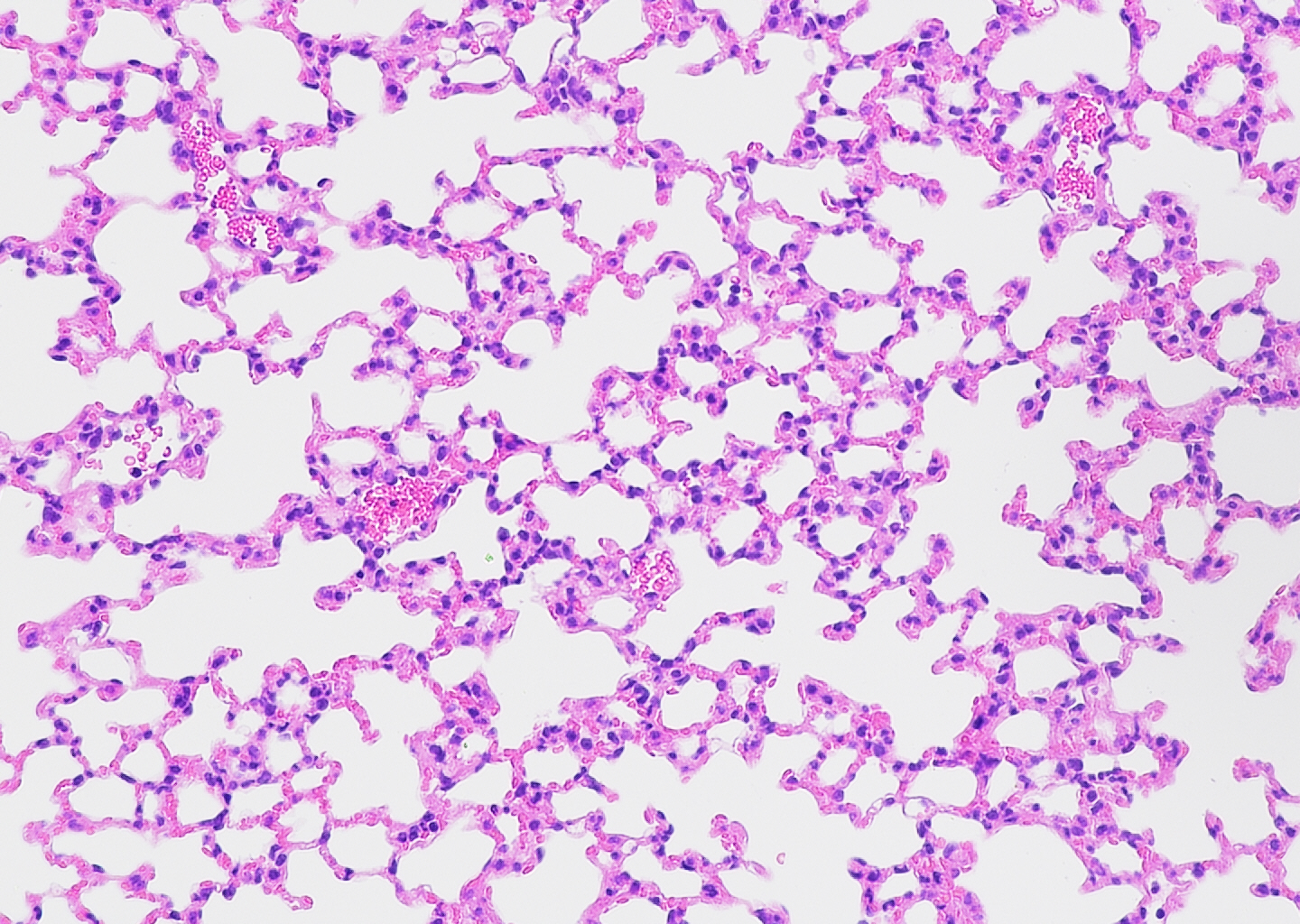

Supplement: Supplementary file 1 [file toxins-18-00278-s001.zip › Figure S7. Original full-size histopathological images of vital organs (heart, liver, spleen, lung, kidney) corresponding to Figure 11B/PEG-EGCG-Lung.jpg]

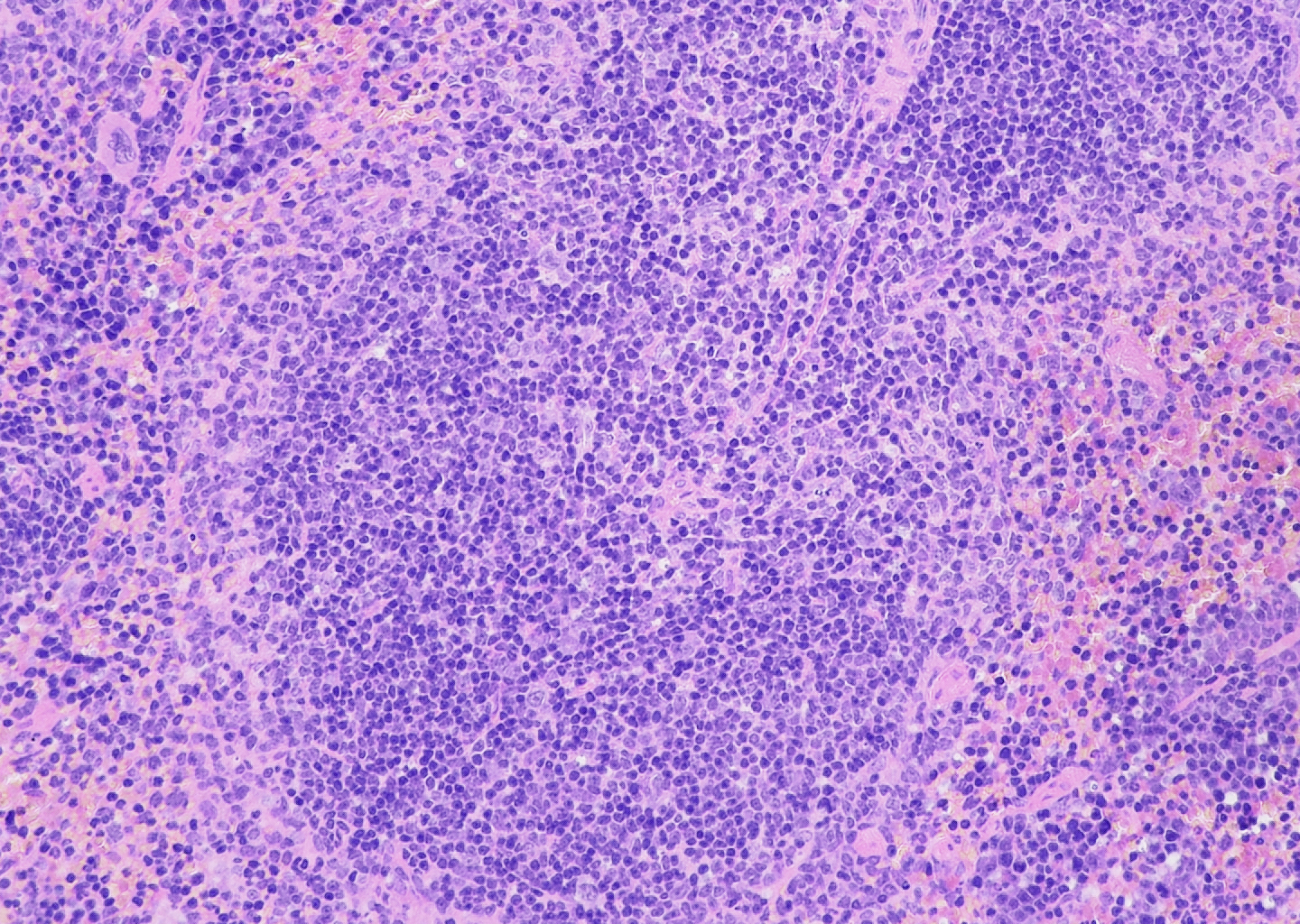

Supplement: Supplementary file 1 [file toxins-18-00278-s001.zip › Figure S7. Original full-size histopathological images of vital organs (heart, liver, spleen, lung, kidney) corresponding to Figure 11B/PEG-EGCG-Spleen.jpg]

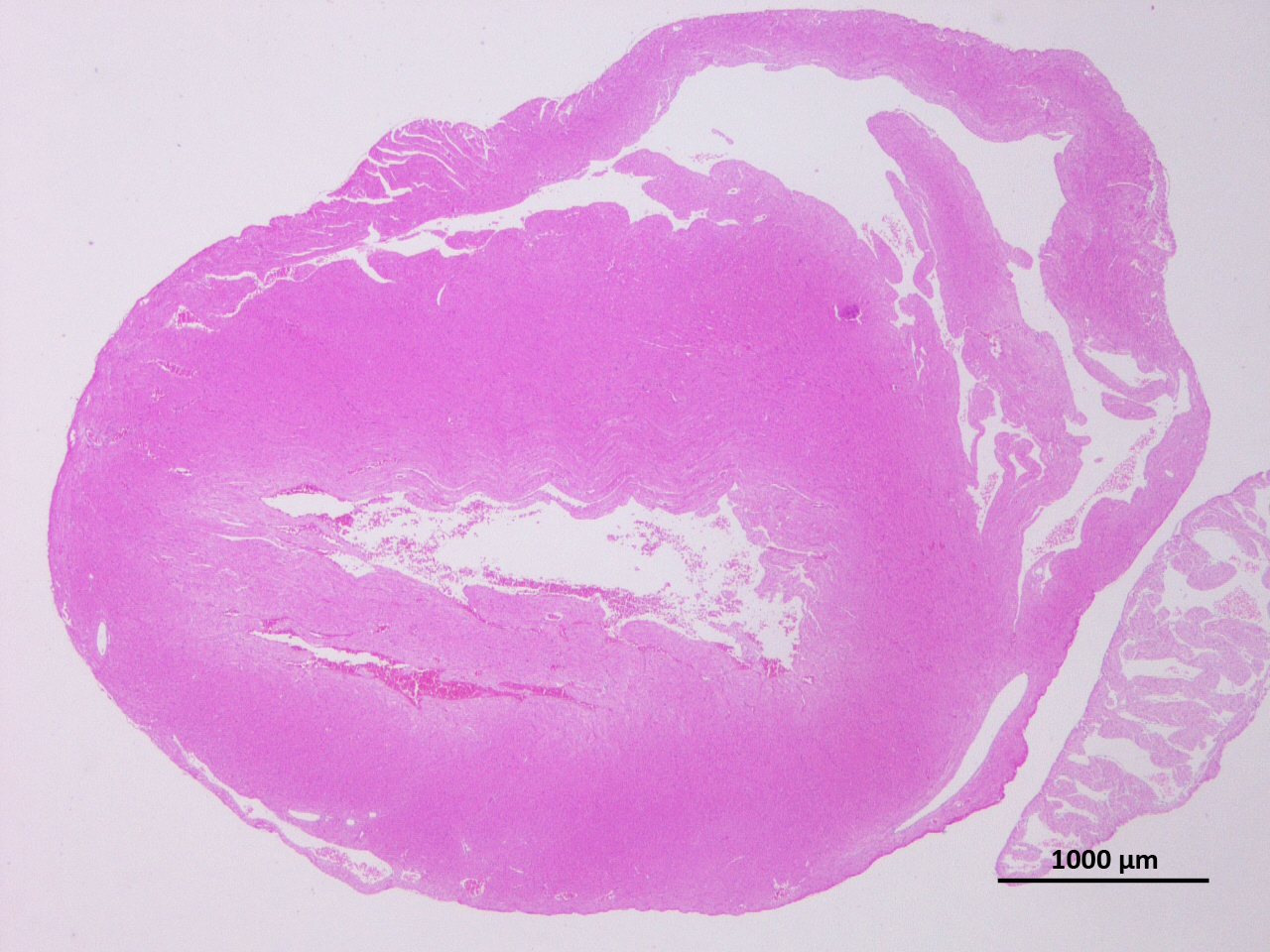

Supplement: Supplementary file 1 [file toxins-18-00278-s001.zip › Figure S8. Uncropped full-size histopathological micrographs of heart tissues corresponding to Figure 13B/Figure-13B-Heart/Control—1.jpg]

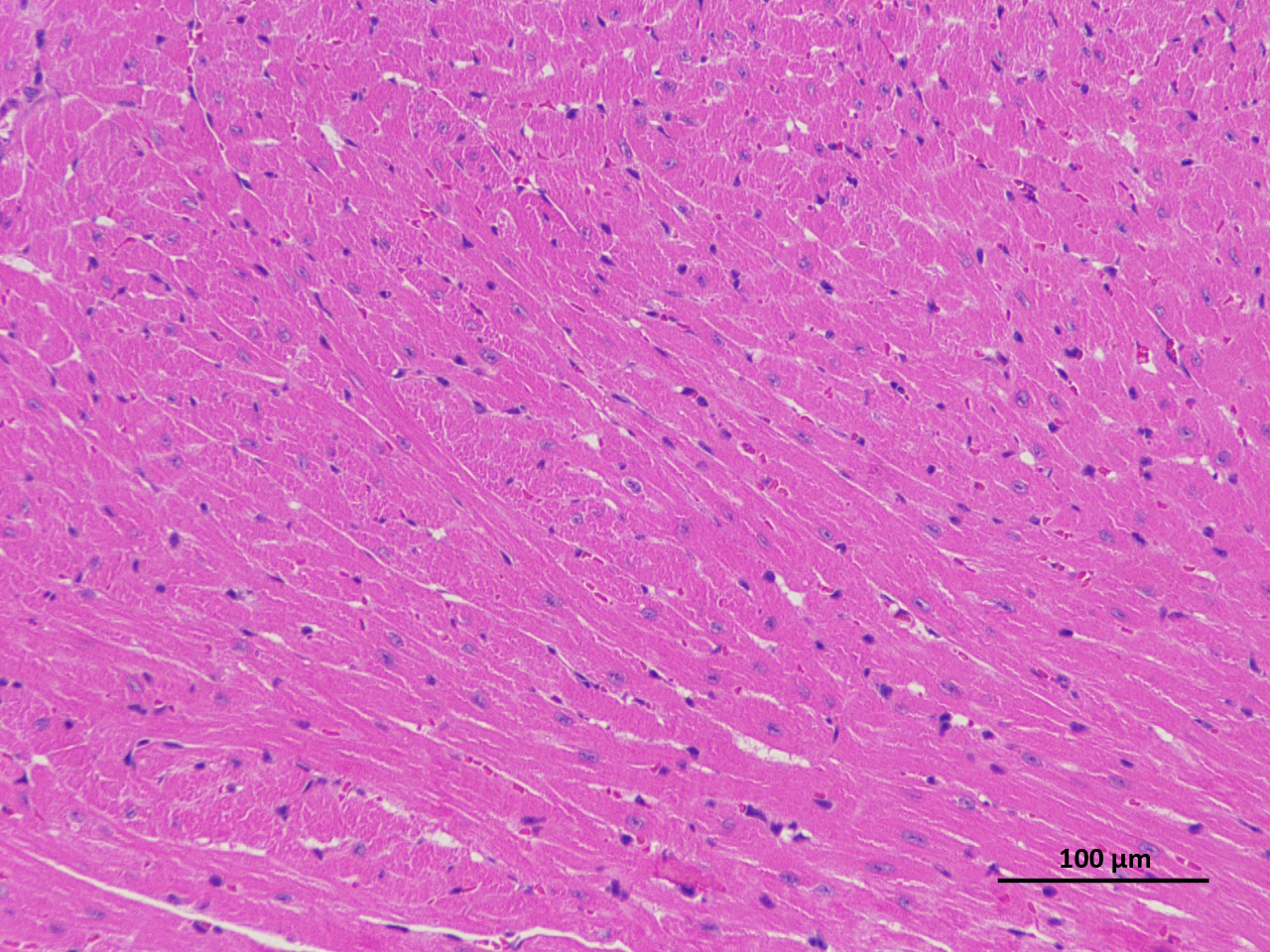

Supplement: Supplementary file 1 [file toxins-18-00278-s001.zip › Figure S8. Uncropped full-size histopathological micrographs of heart tissues corresponding to Figure 13B/Figure-13B-Heart/Control—2.jpg]

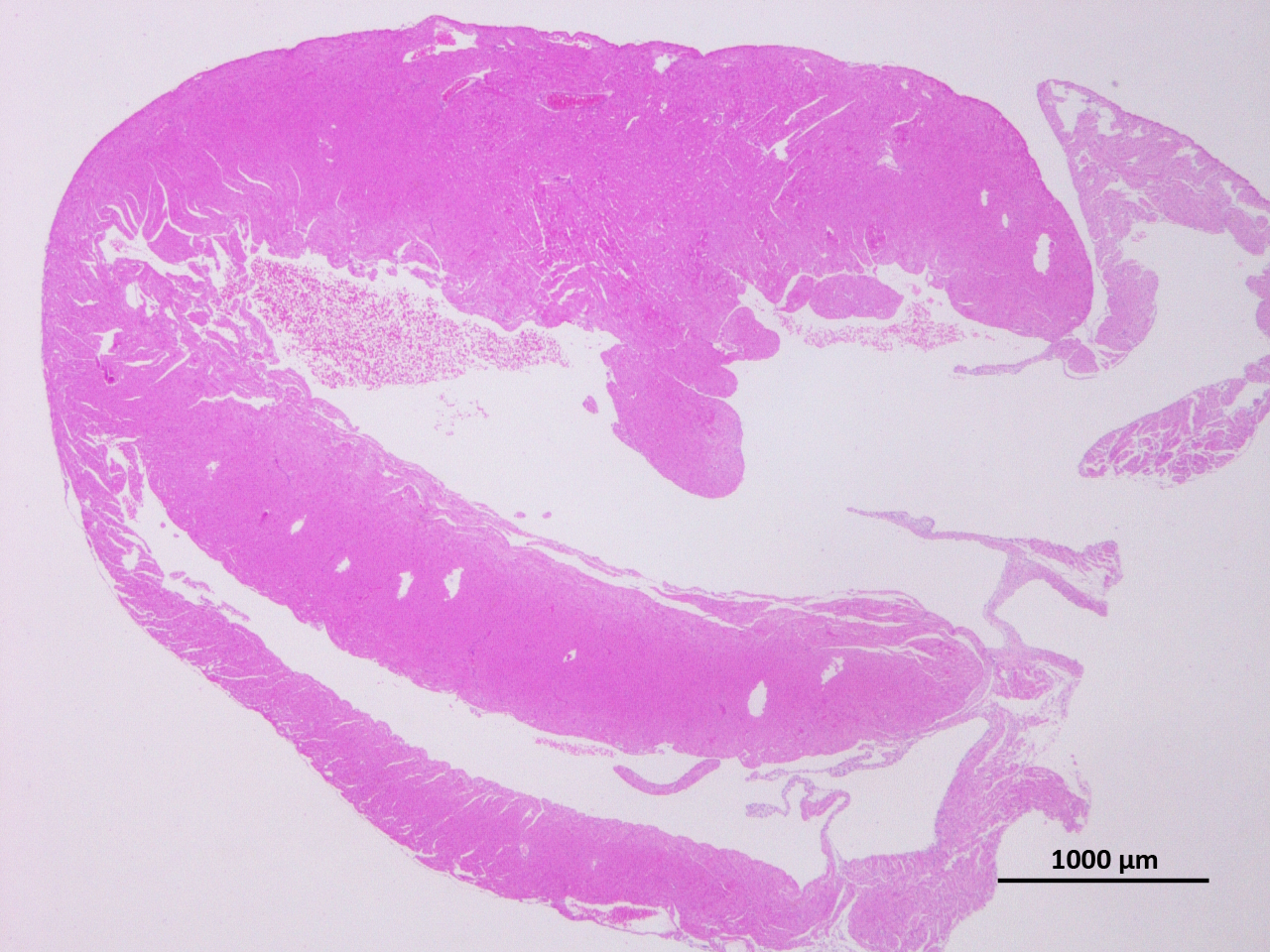

Supplement: Supplementary file 1 [file toxins-18-00278-s001.zip › Figure S8. Uncropped full-size histopathological micrographs of heart tissues corresponding to Figure 13B/Figure-13B-Heart/EGCG—1.jpg]

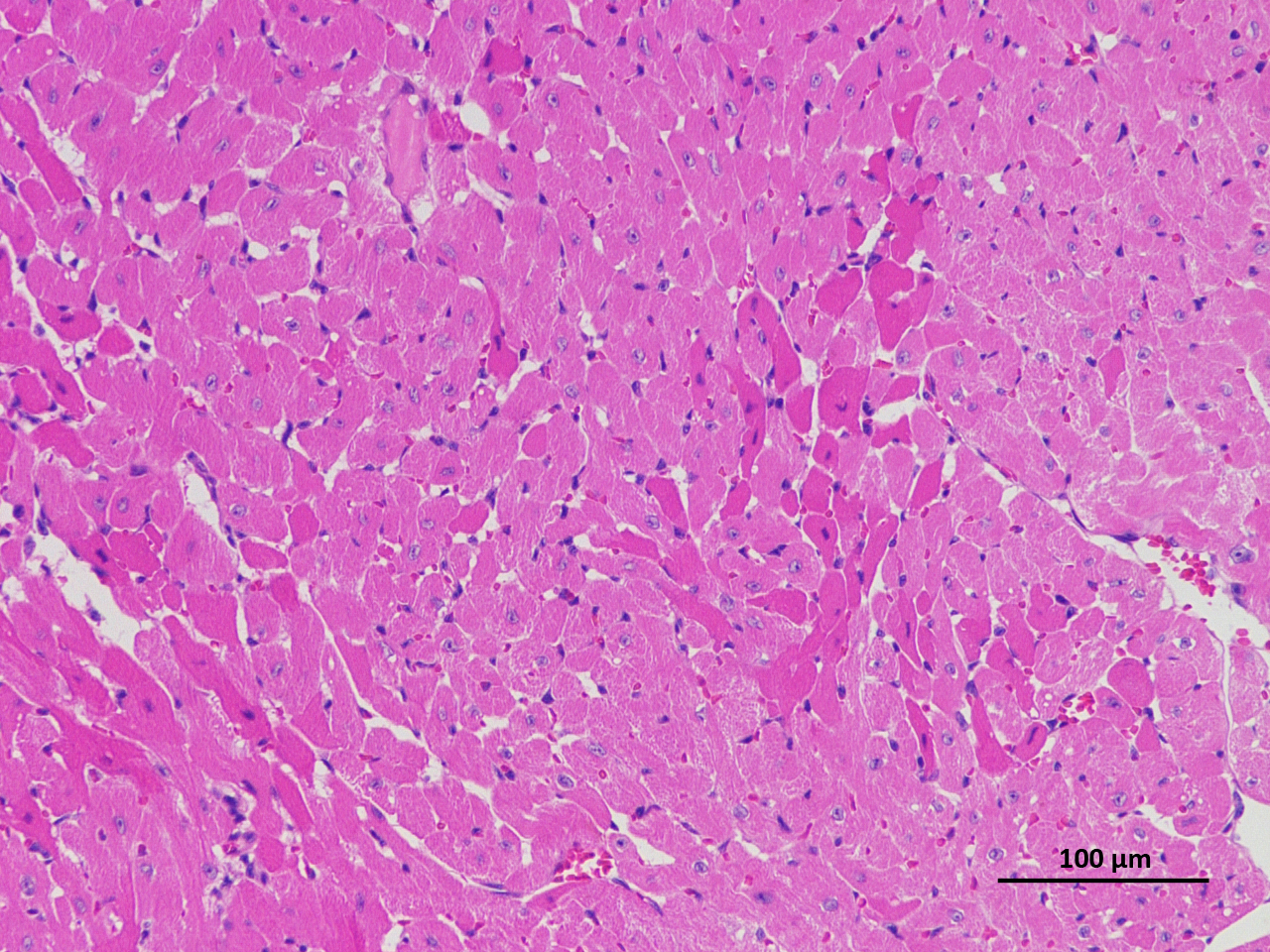

Supplement: Supplementary file 1 [file toxins-18-00278-s001.zip › Figure S8. Uncropped full-size histopathological micrographs of heart tissues corresponding to Figure 13B/Figure-13B-Heart/EGCG—2.jpg]

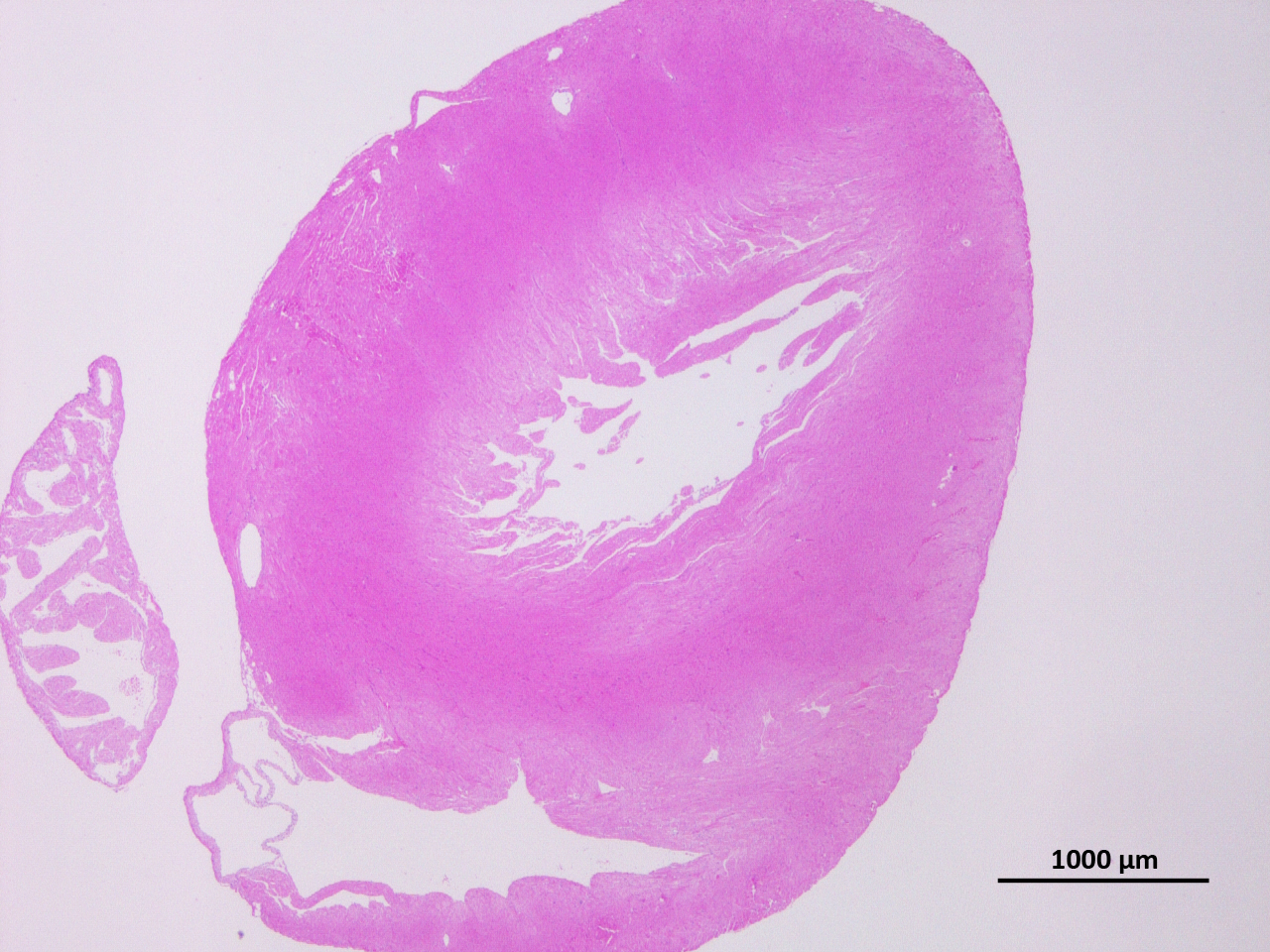

Supplement: Supplementary file 1 [file toxins-18-00278-s001.zip › Figure S8. Uncropped full-size histopathological micrographs of heart tissues corresponding to Figure 13B/Figure-13B-Heart/HTC—1.jpg]

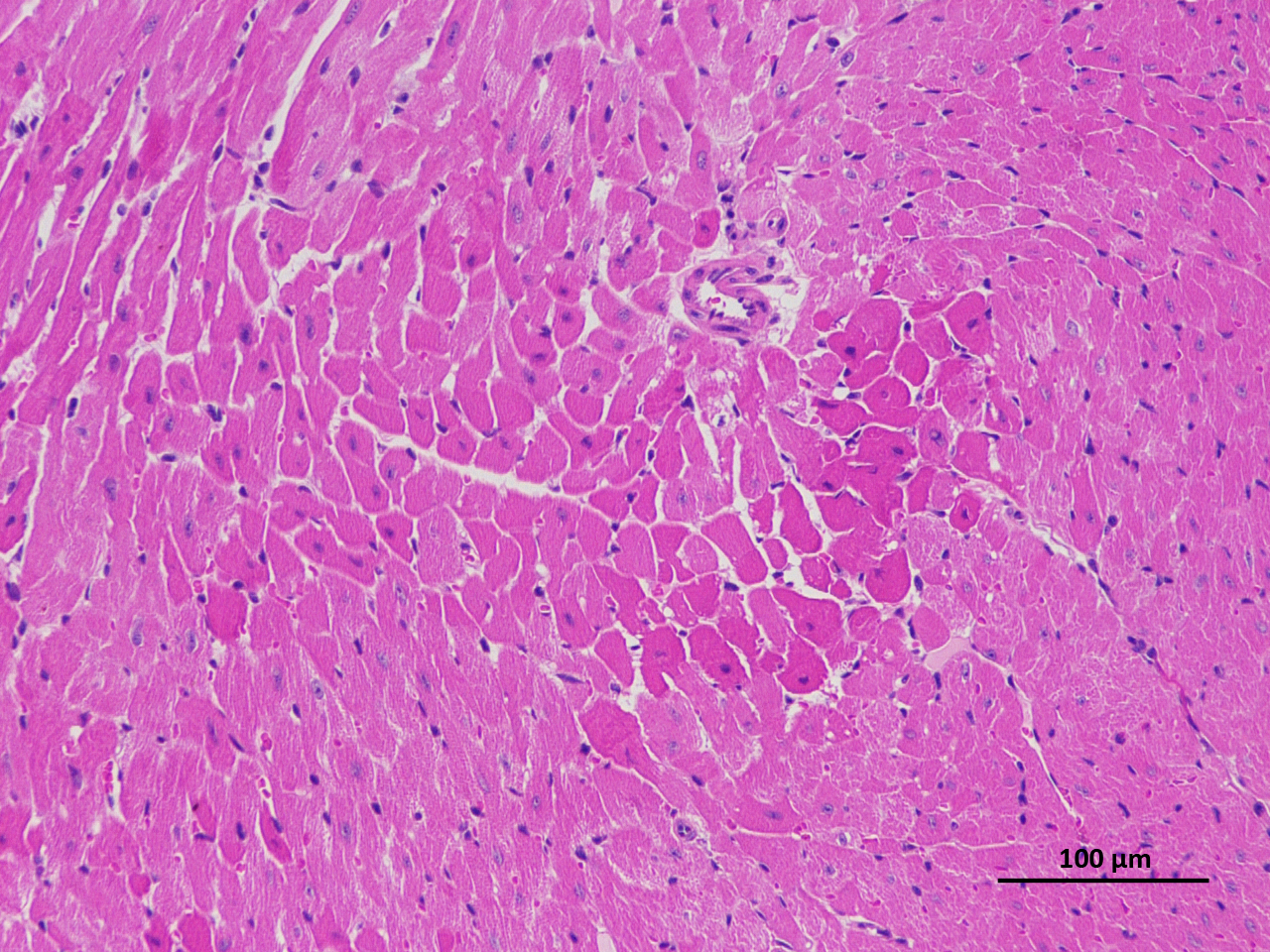

Supplement: Supplementary file 1 [file toxins-18-00278-s001.zip › Figure S8. Uncropped full-size histopathological micrographs of heart tissues corresponding to Figure 13B/Figure-13B-Heart/HTC—2.jpg]

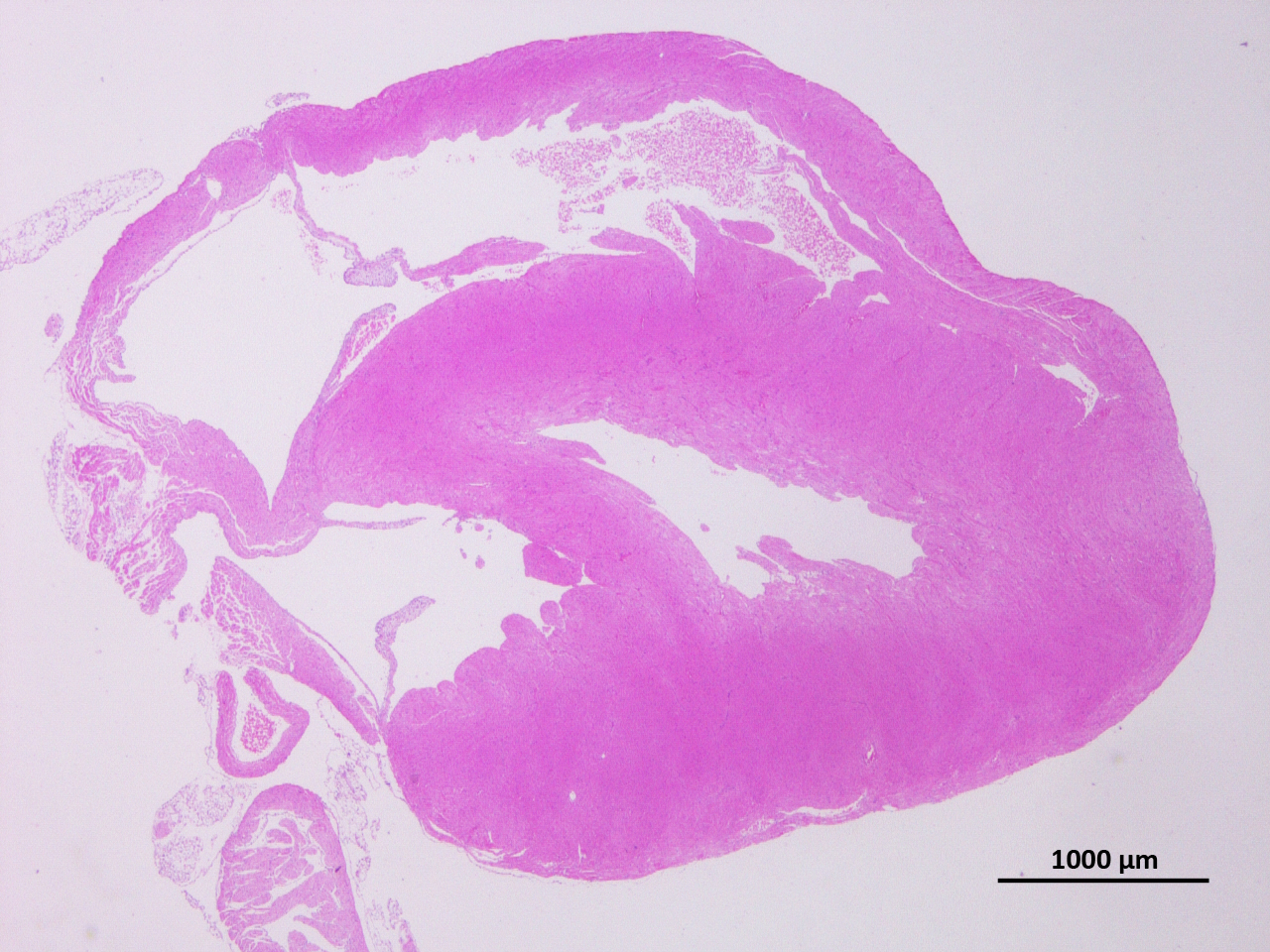

Supplement: Supplementary file 1 [file toxins-18-00278-s001.zip › Figure S8. Uncropped full-size histopathological micrographs of heart tissues corresponding to Figure 13B/Figure-13B-Heart/PEG-EGCG-HTC—1.jpg]

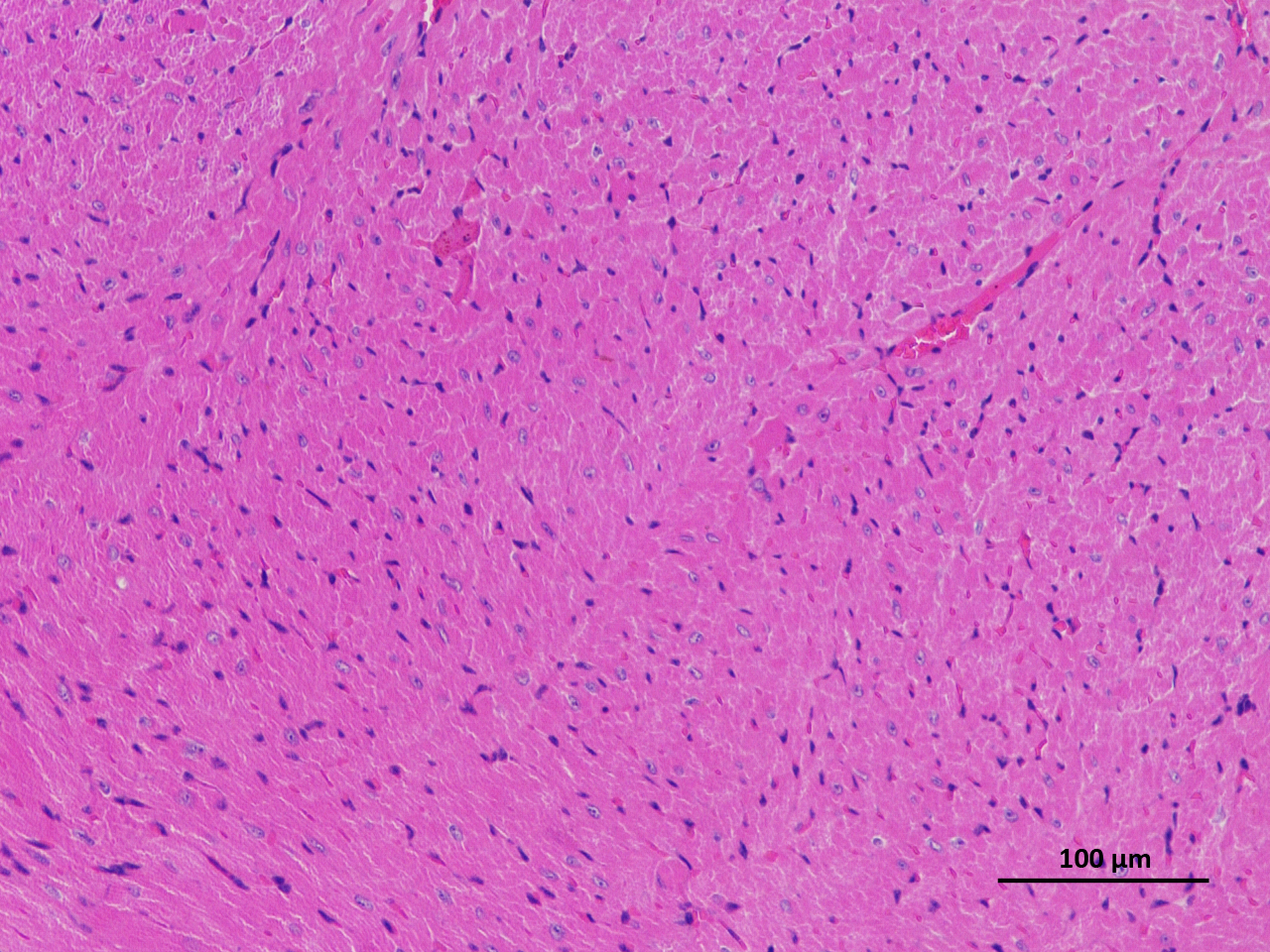

Supplement: Supplementary file 1 [file toxins-18-00278-s001.zip › Figure S8. Uncropped full-size histopathological micrographs of heart tissues corresponding to Figure 13B/Figure-13B-Heart/PEG-EGCG-HTC—2.jpg]

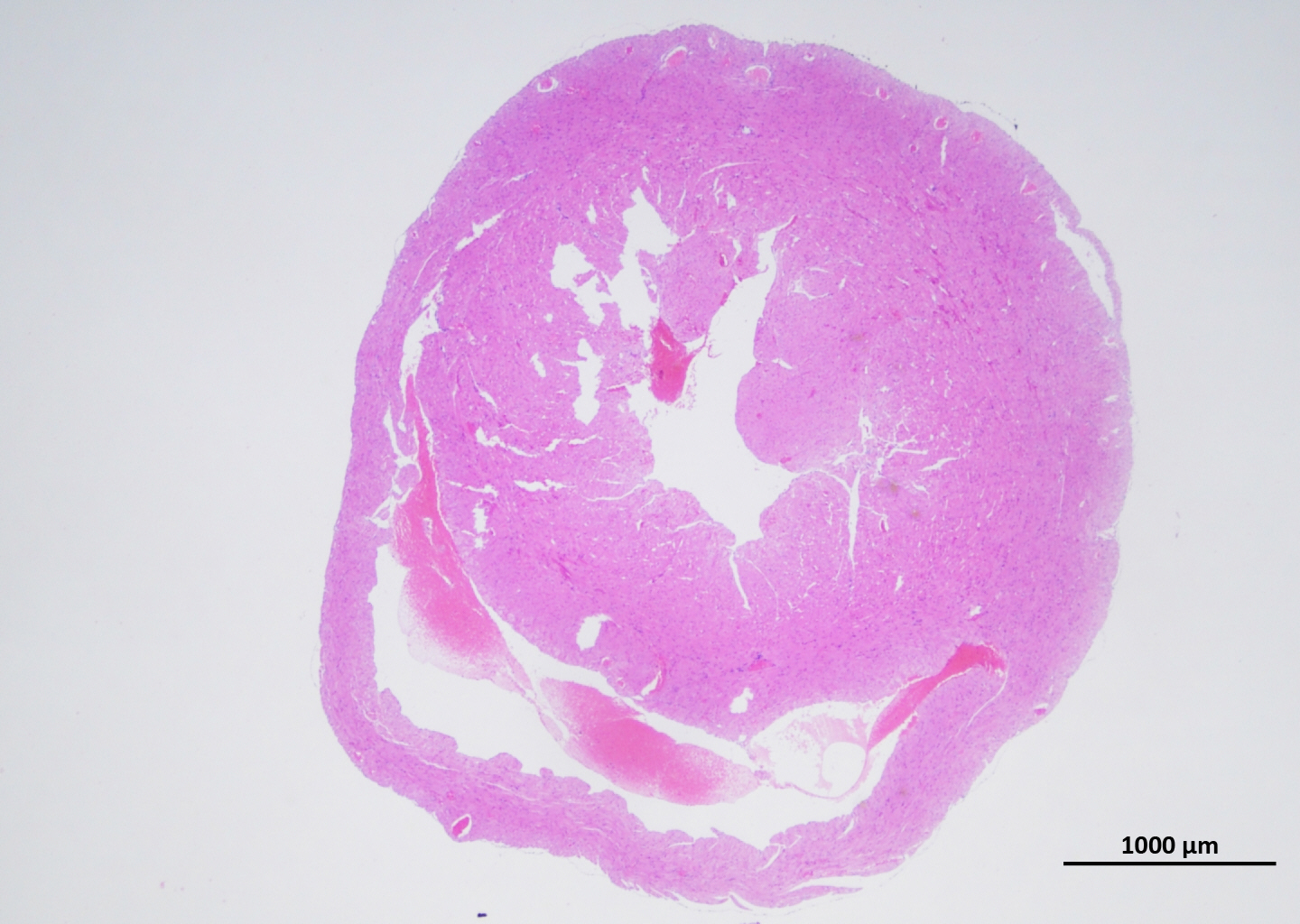

Supplement: Supplementary file 1 [file toxins-18-00278-s001.zip › Figure S8. Uncropped full-size histopathological micrographs of heart tissues corresponding to Figure 13B/Figure-13B-Heart/PEG-EGCG—1.jpg]

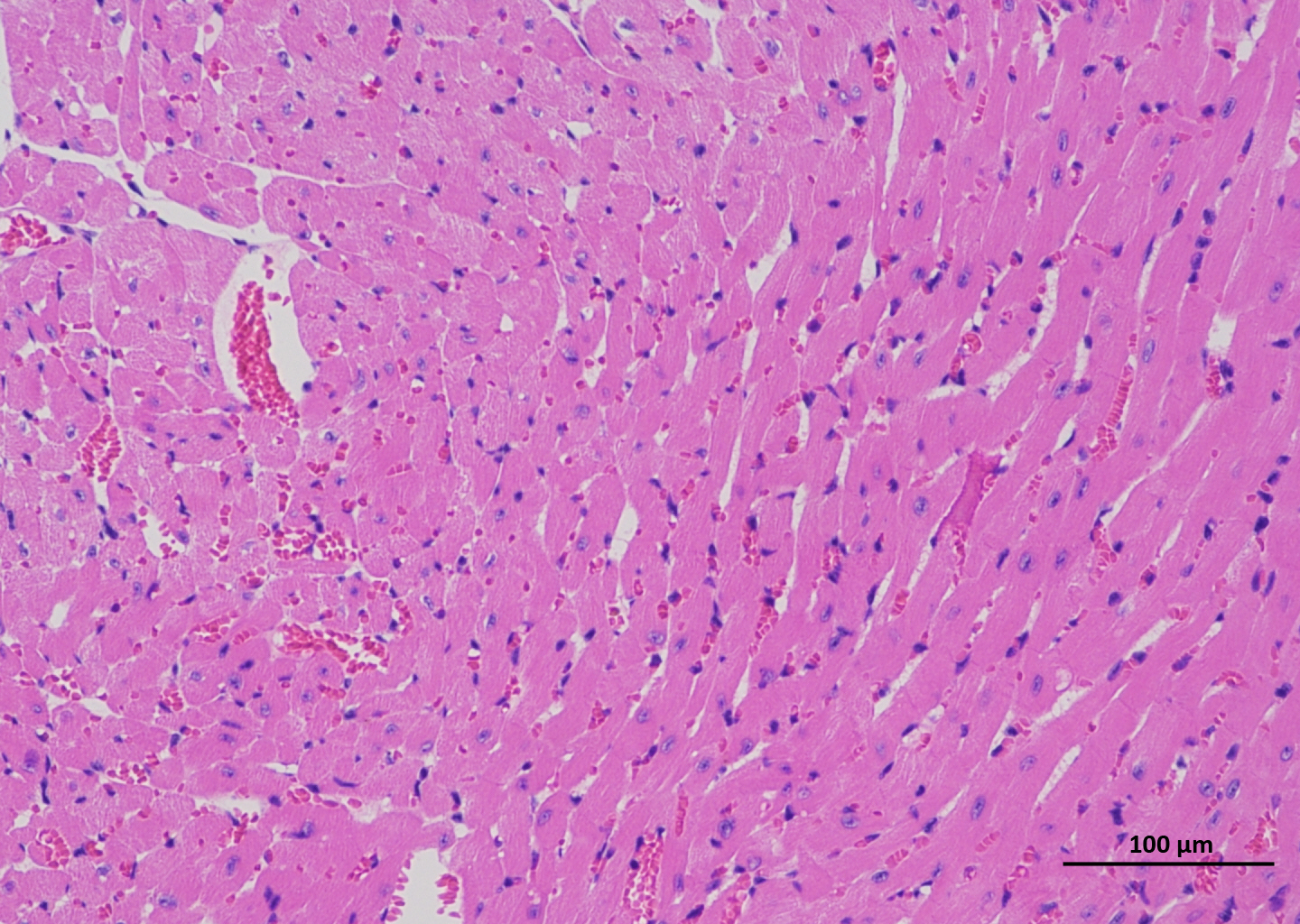

Supplement: Supplementary file 1 [file toxins-18-00278-s001.zip › Figure S8. Uncropped full-size histopathological micrographs of heart tissues corresponding to Figure 13B/Figure-13B-Heart/PEG-EGCG—2.jpg]

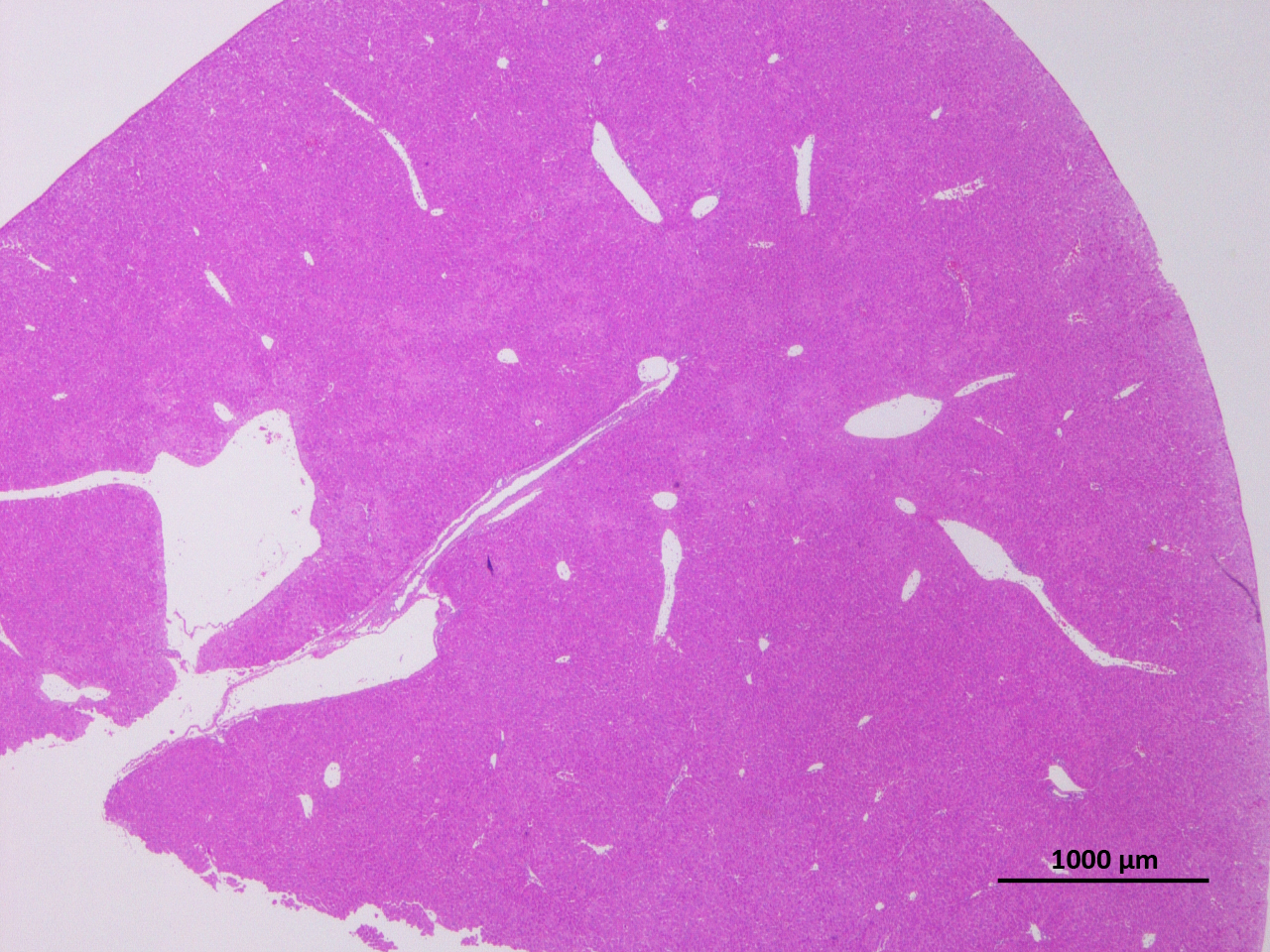

Supplement: Supplementary file 1 [file toxins-18-00278-s001.zip › Figure S9. Uncropped full-size histopathological micrographs of liver tissues corresponding to Figure 13B/Figure-13B-Liver/EGCG—1.jpg]

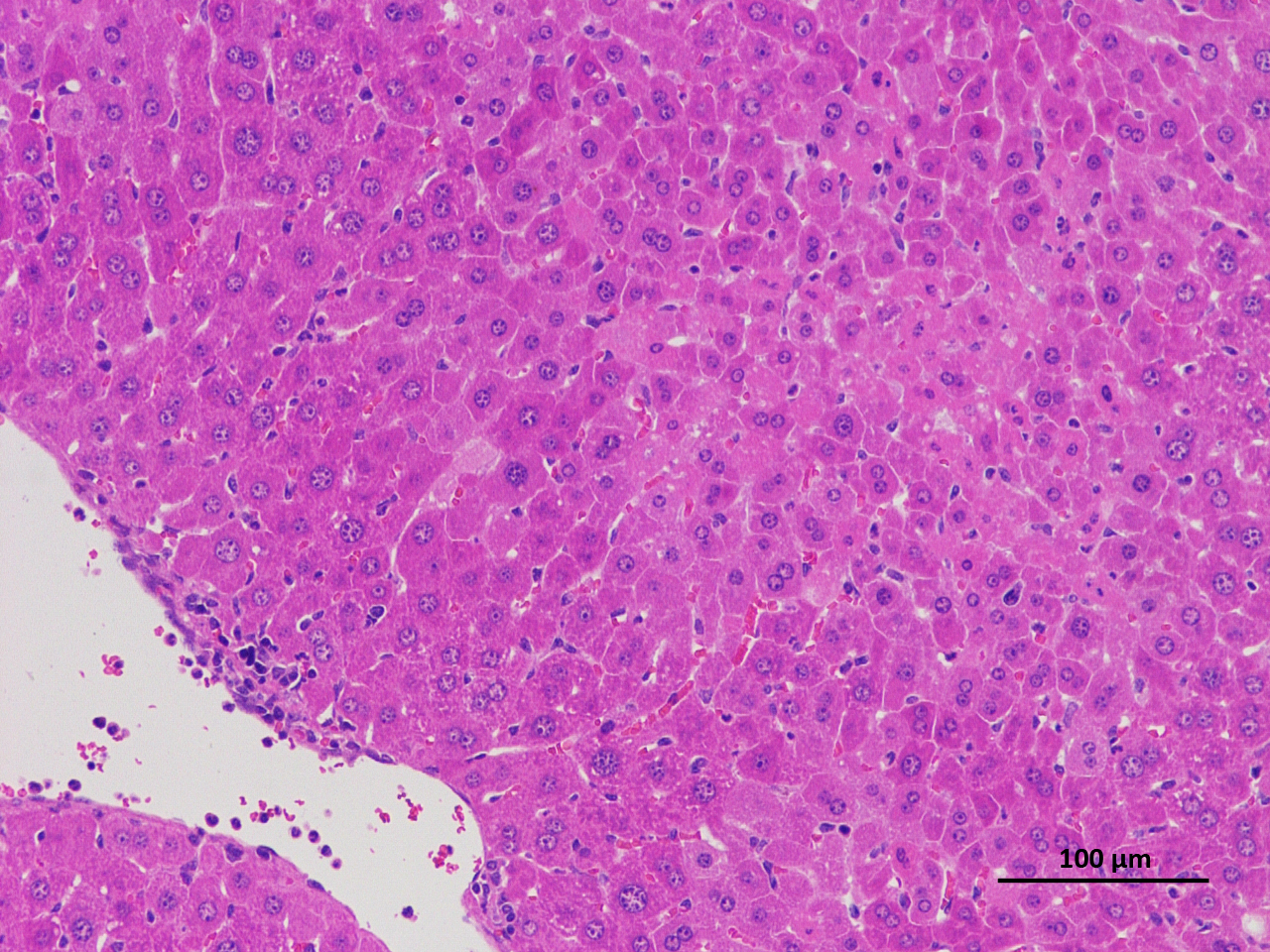

Supplement: Supplementary file 1 [file toxins-18-00278-s001.zip › Figure S9. Uncropped full-size histopathological micrographs of liver tissues corresponding to Figure 13B/Figure-13B-Liver/EGCG—2.jpg]

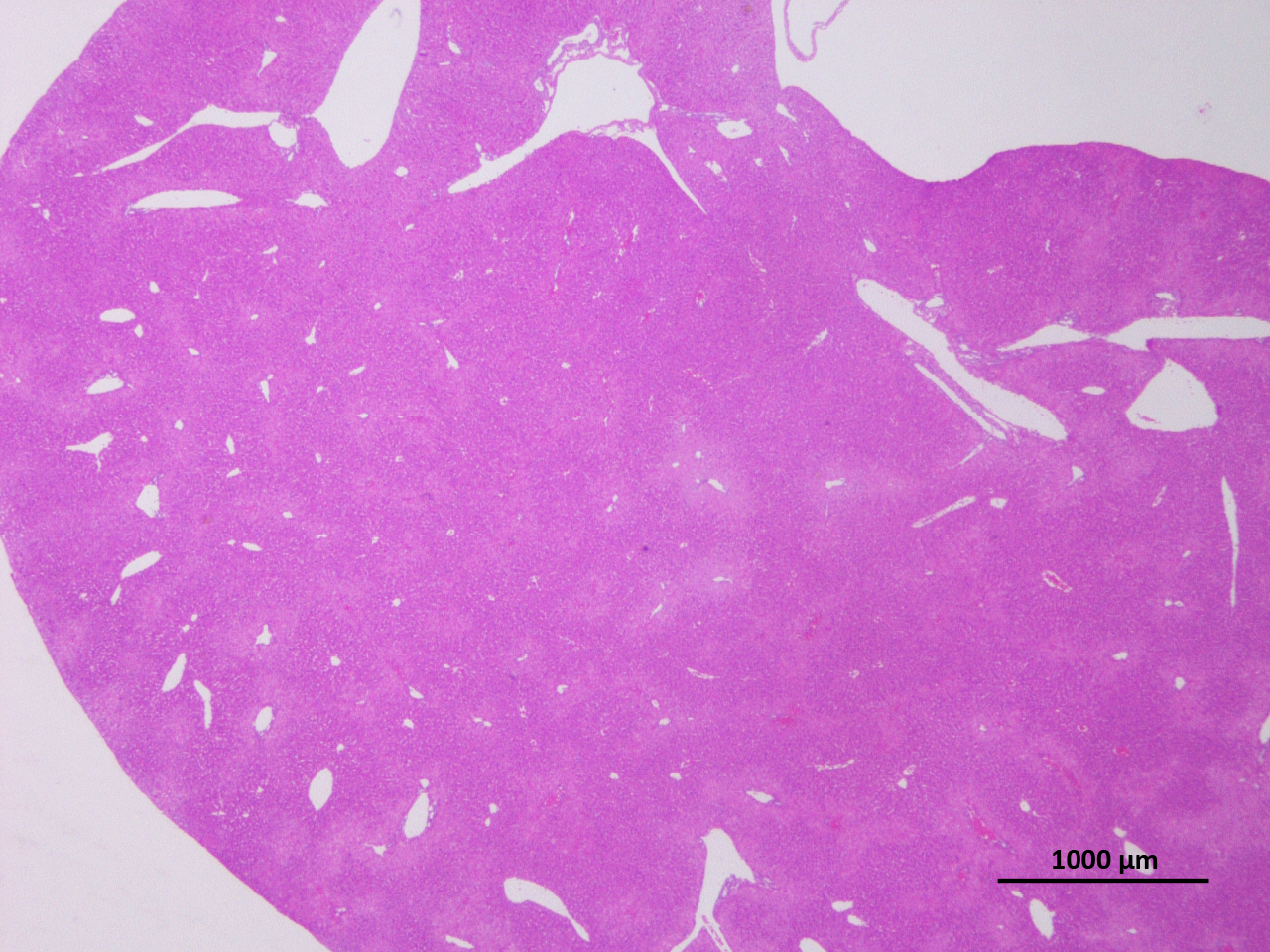

Supplement: Supplementary file 1 [file toxins-18-00278-s001.zip › Figure S9. Uncropped full-size histopathological micrographs of liver tissues corresponding to Figure 13B/Figure-13B-Liver/HTC—1.jpg]

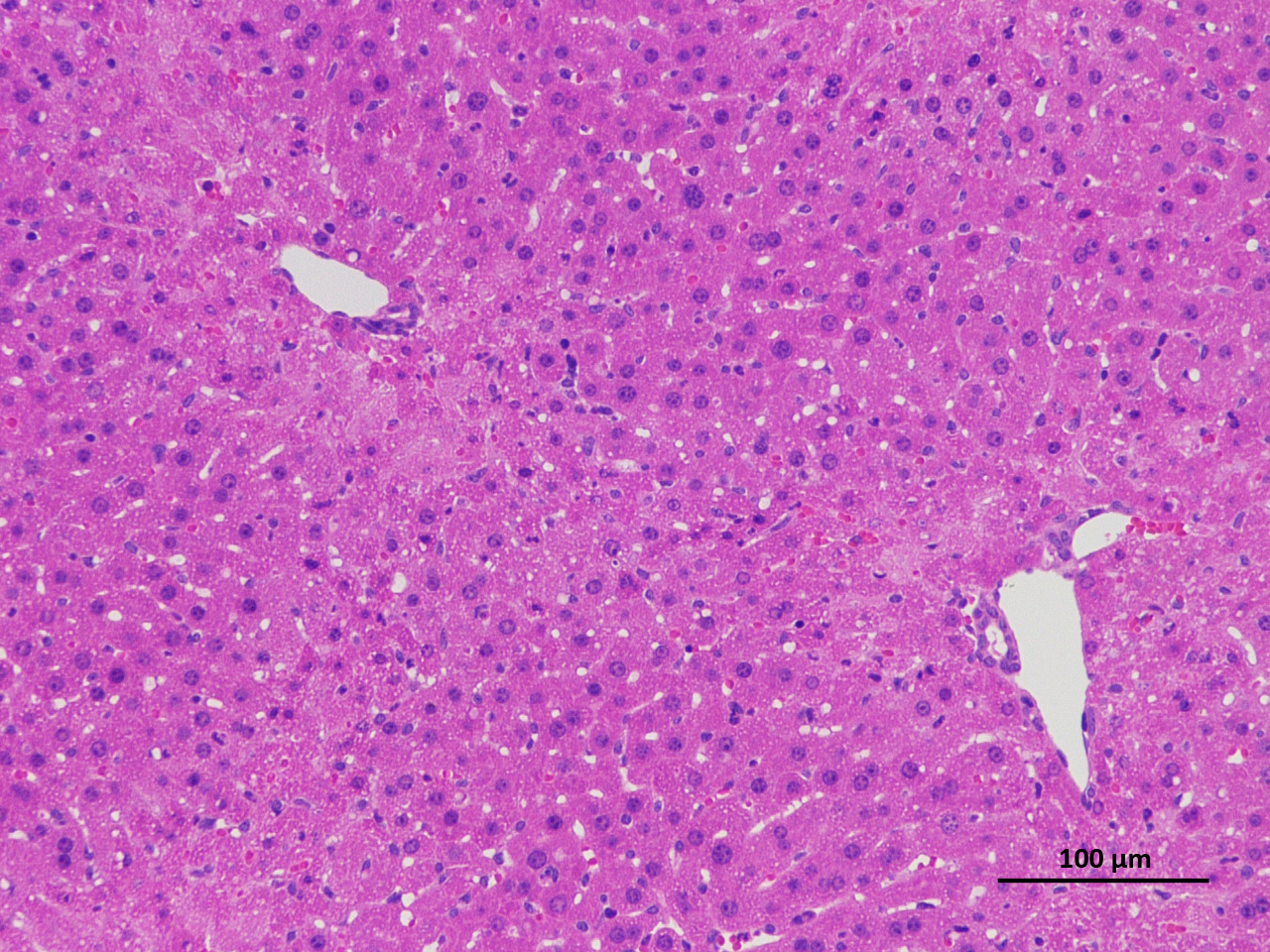

Supplement: Supplementary file 1 [file toxins-18-00278-s001.zip › Figure S9. Uncropped full-size histopathological micrographs of liver tissues corresponding to Figure 13B/Figure-13B-Liver/HTC—2.jpg]

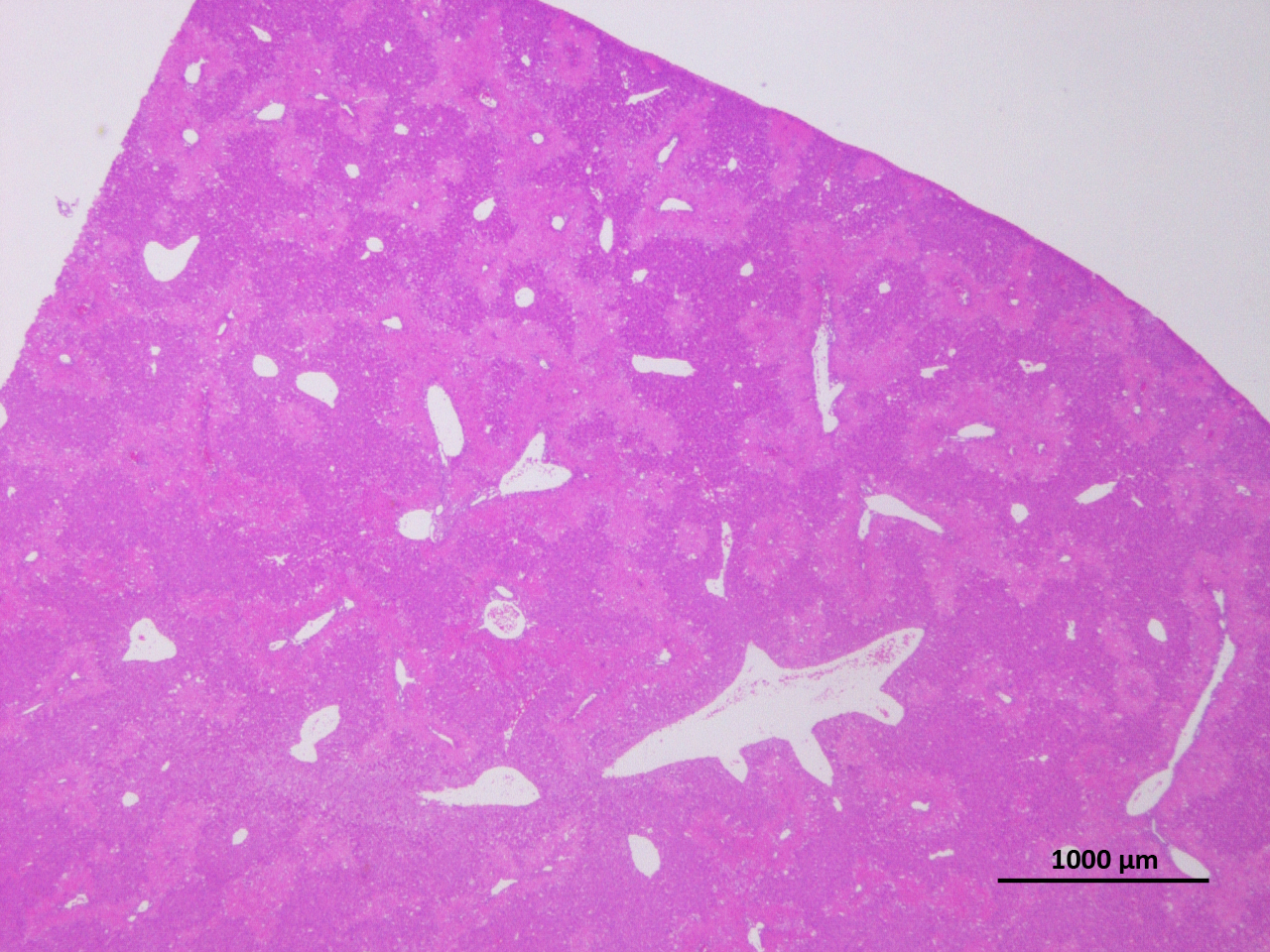

Supplement: Supplementary file 1 [file toxins-18-00278-s001.zip › Figure S9. Uncropped full-size histopathological micrographs of liver tissues corresponding to Figure 13B/Figure-13B-Liver/PBS—1.jpg]

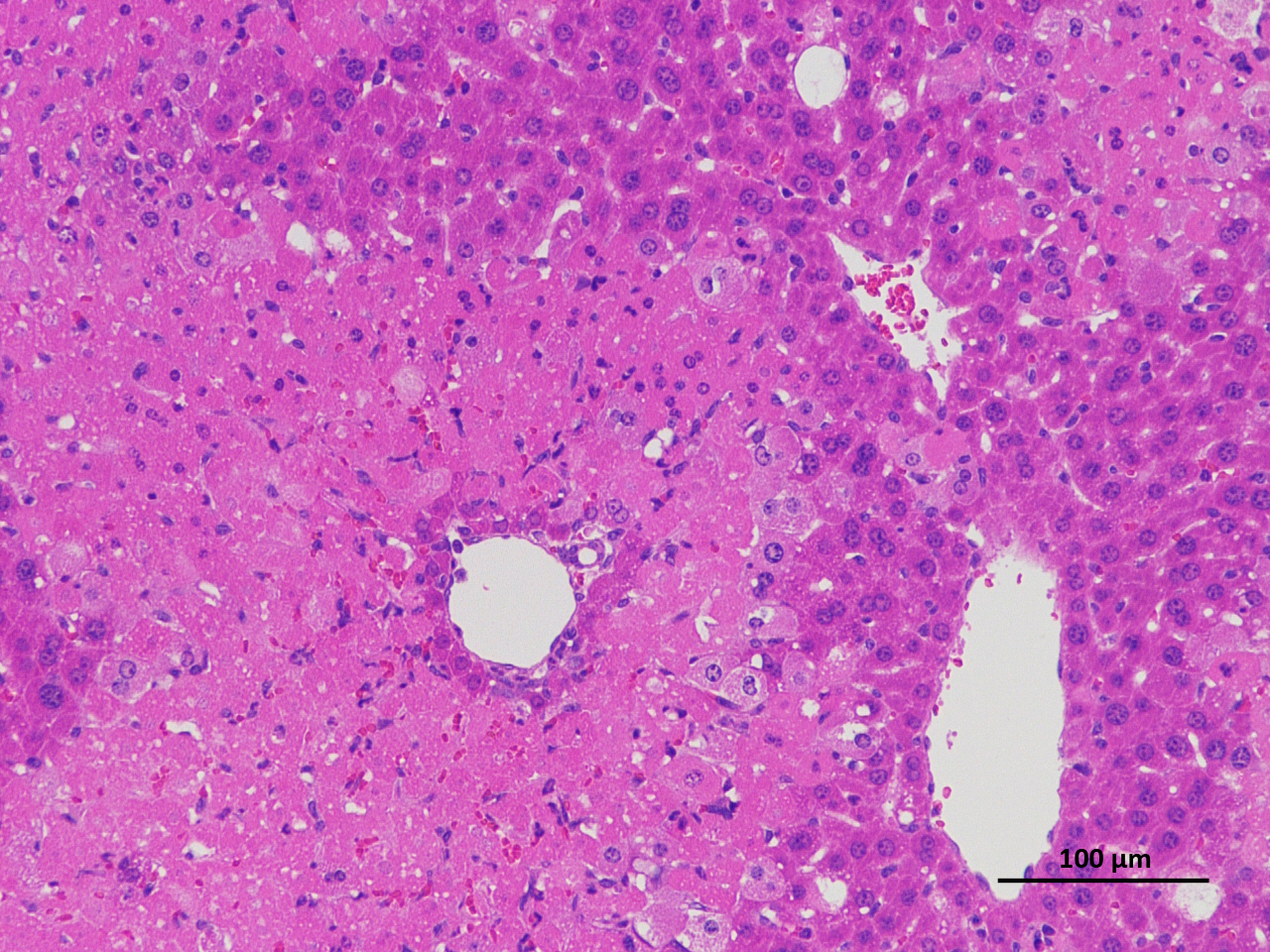

Supplement: Supplementary file 1 [file toxins-18-00278-s001.zip › Figure S9. Uncropped full-size histopathological micrographs of liver tissues corresponding to Figure 13B/Figure-13B-Liver/PBS—2.jpg]

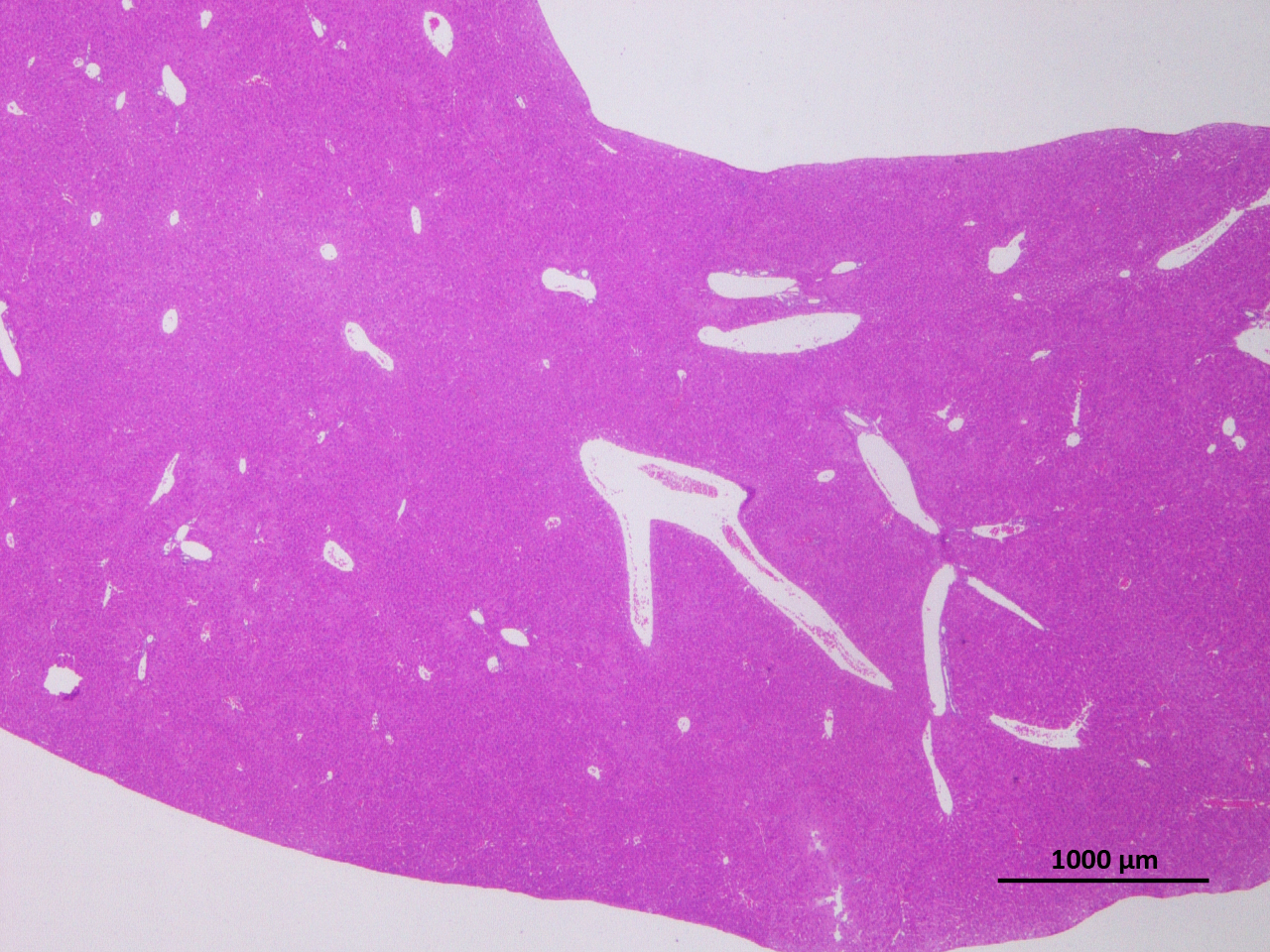

Supplement: Supplementary file 1 [file toxins-18-00278-s001.zip › Figure S9. Uncropped full-size histopathological micrographs of liver tissues corresponding to Figure 13B/Figure-13B-Liver/PEG-EGCG—1.jpg]

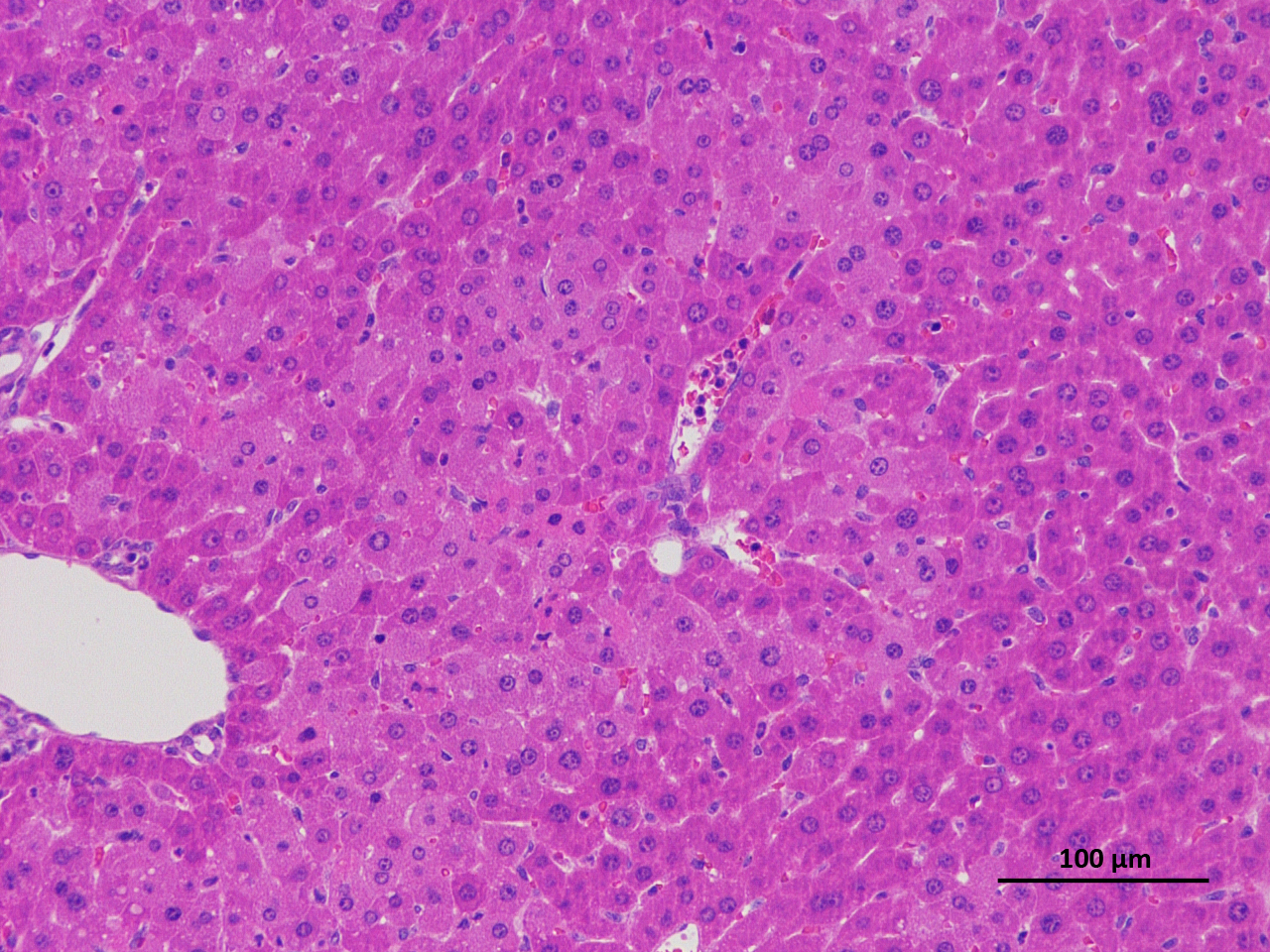

Supplement: Supplementary file 1 [file toxins-18-00278-s001.zip › Figure S9. Uncropped full-size histopathological micrographs of liver tissues corresponding to Figure 13B/Figure-13B-Liver/PEG-EGCG—2.jpg]

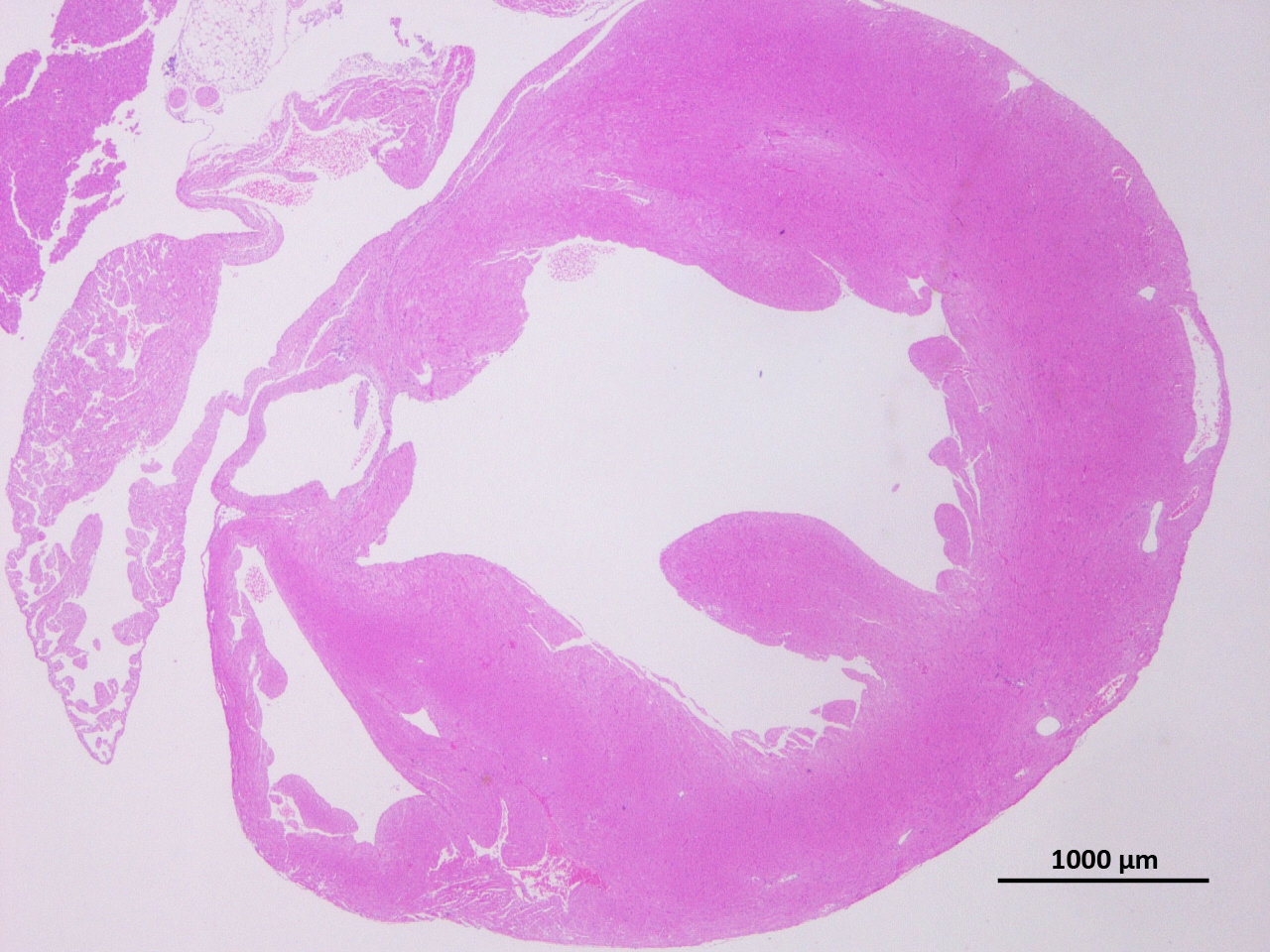

Supplement: Supplementary file 1 [file toxins-18-00278-s001.zip › Figure S10. Uncropped full-size histopathological micrographs of heart tissues corresponding to Figure 14B/Figure-14B-Heart/Control—1.jpg]

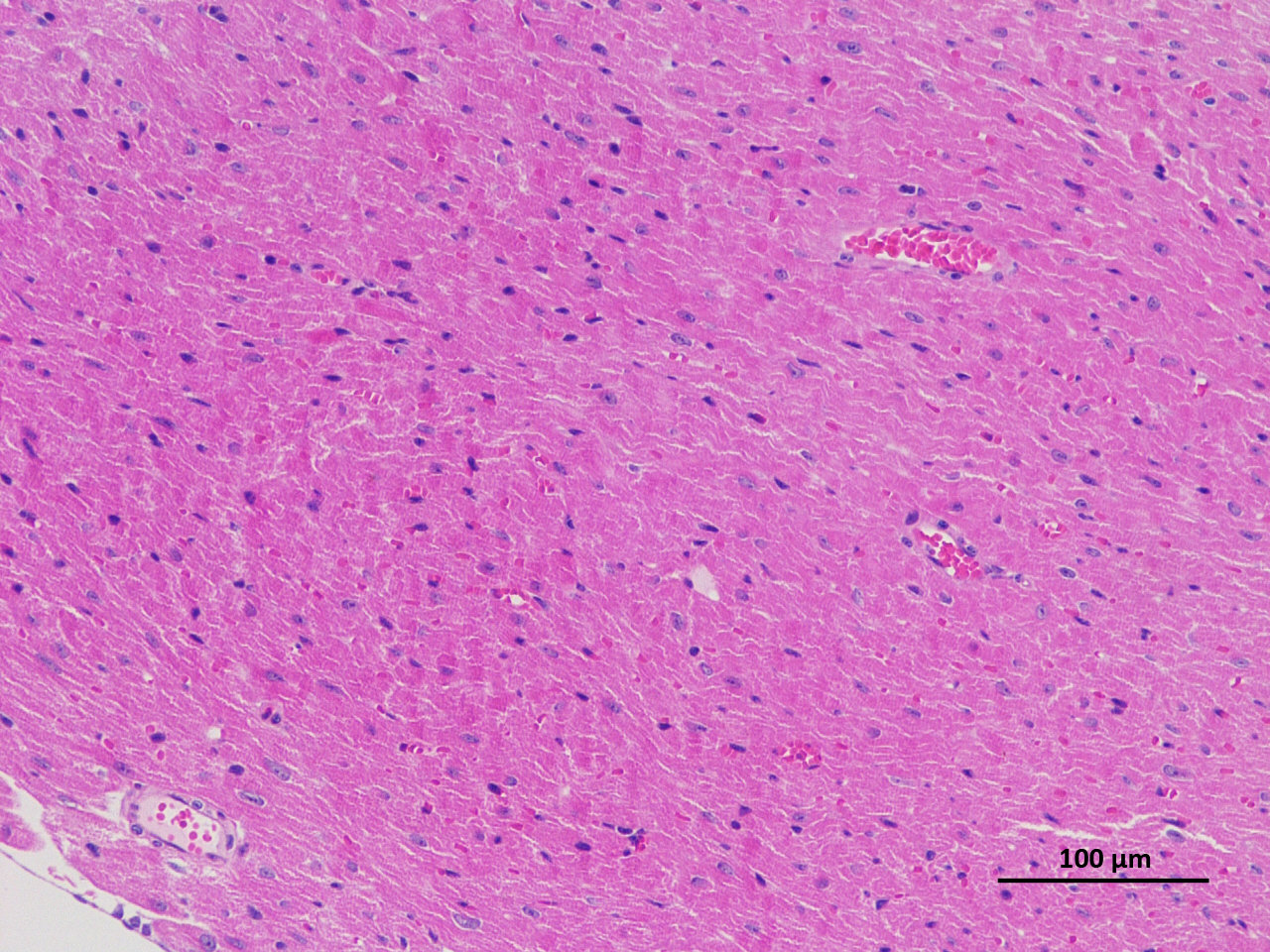

Supplement: Supplementary file 1 [file toxins-18-00278-s001.zip › Figure S10. Uncropped full-size histopathological micrographs of heart tissues corresponding to Figure 14B/Figure-14B-Heart/Control—2.jpg]

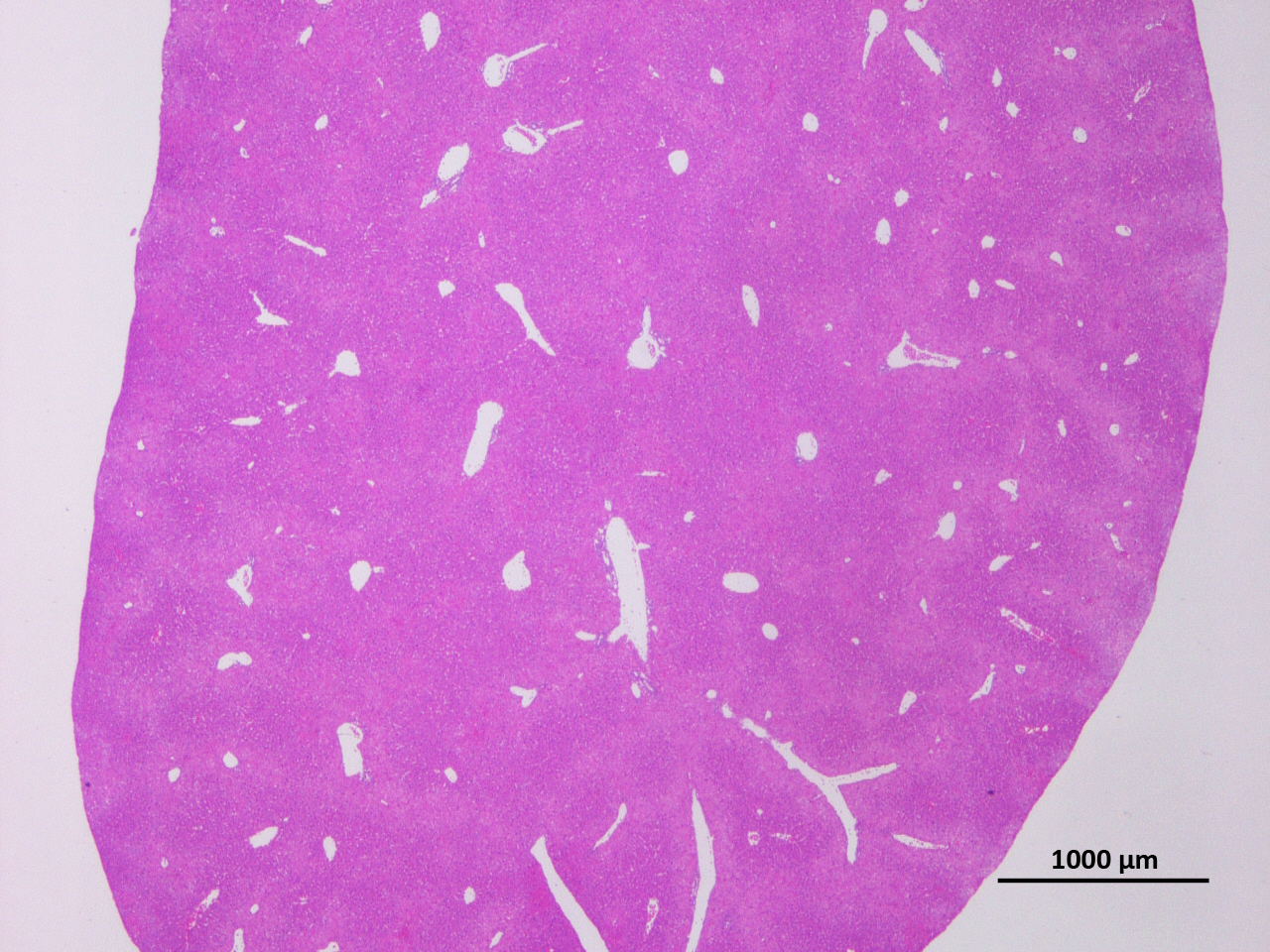

Supplement: Supplementary file 1 [file toxins-18-00278-s001.zip › Figure S11. Uncropped full-size histopathological micrographs of liver tissues corresponding to Figure 14B/Figure-14B-Liver/EGCG—1.jpg]

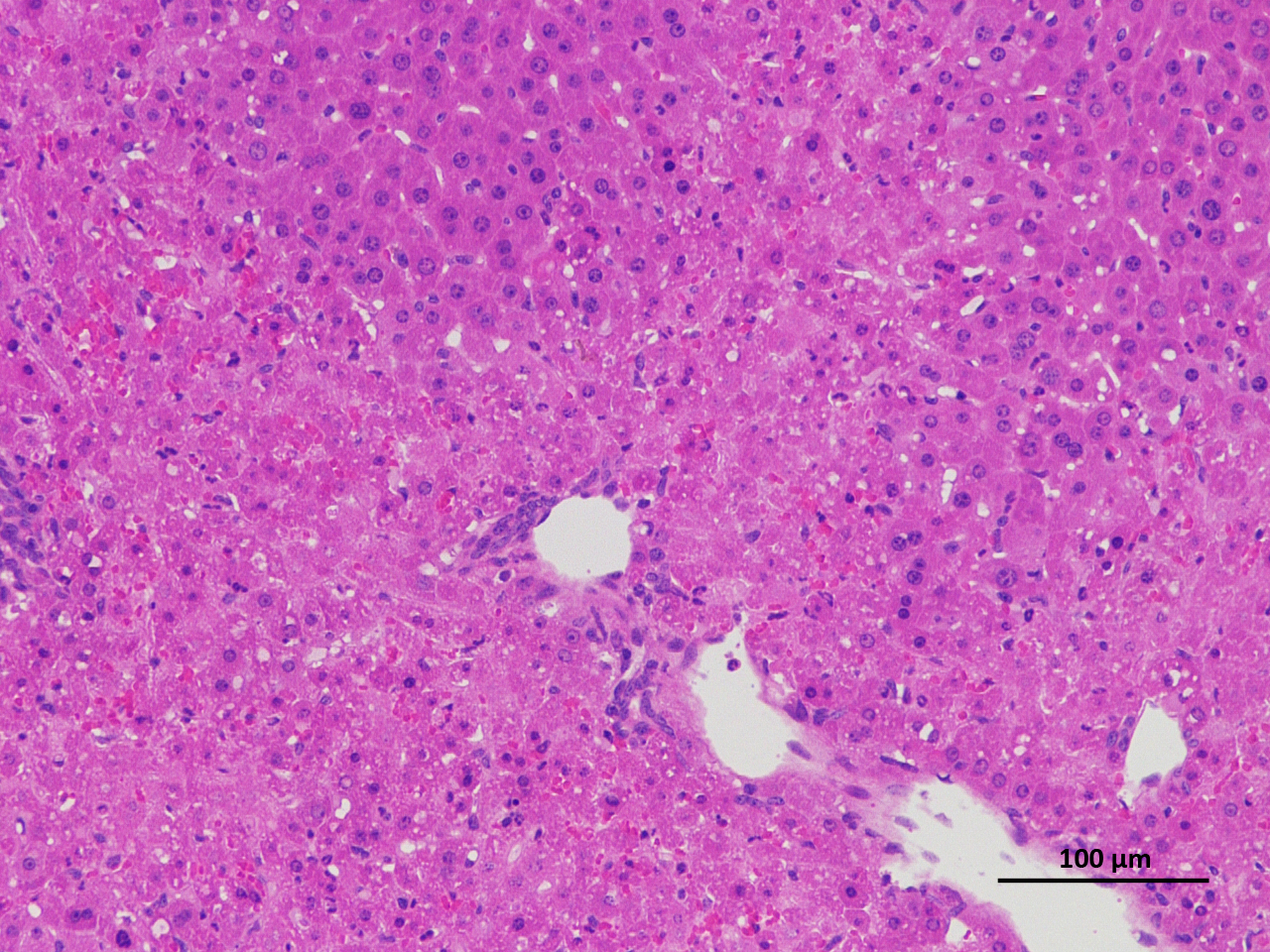

Supplement: Supplementary file 1 [file toxins-18-00278-s001.zip › Figure S11. Uncropped full-size histopathological micrographs of liver tissues corresponding to Figure 14B/Figure-14B-Liver/EGCG—2.jpg]

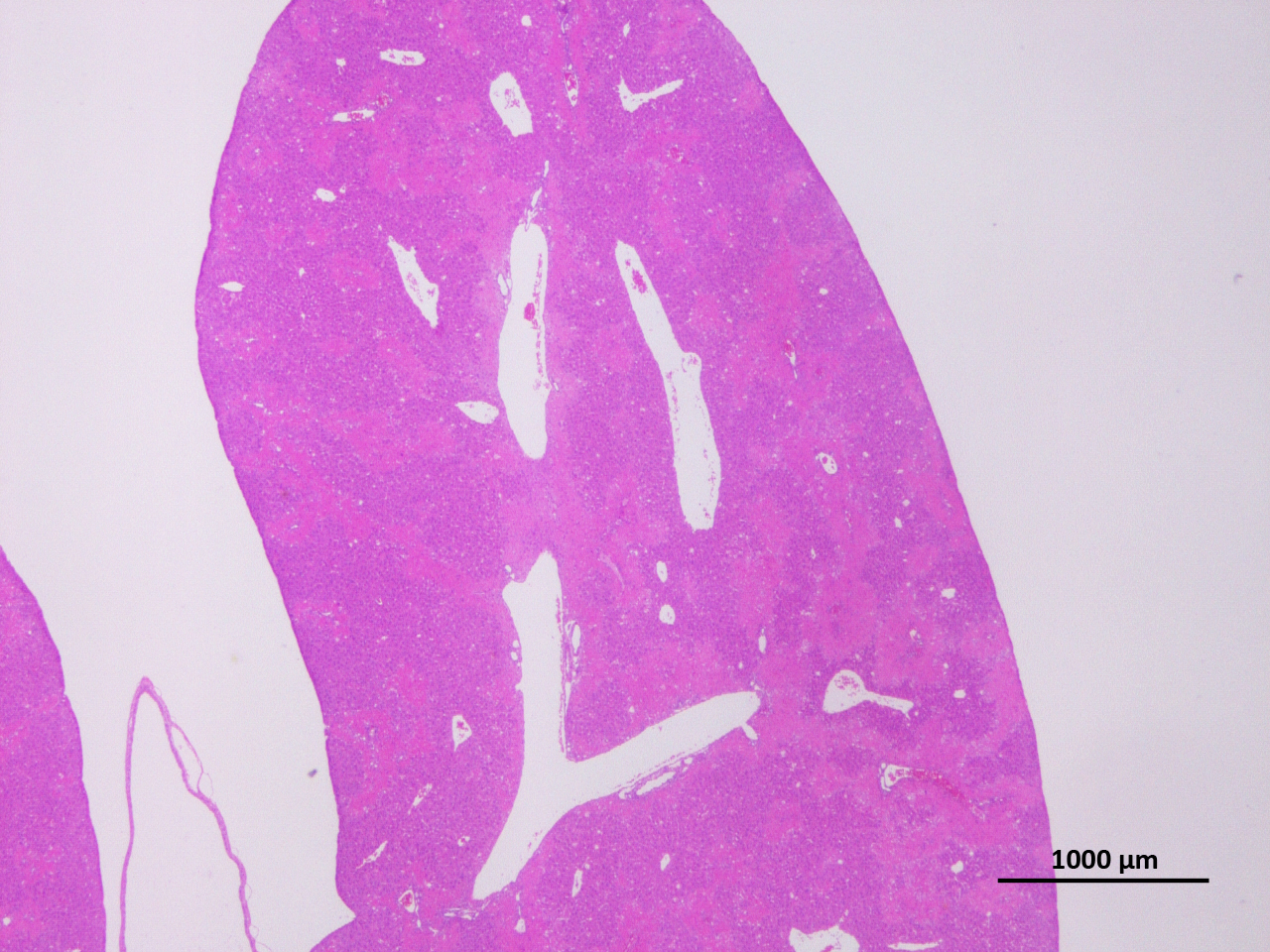

Supplement: Supplementary file 1 [file toxins-18-00278-s001.zip › Figure S11. Uncropped full-size histopathological micrographs of liver tissues corresponding to Figure 14B/Figure-14B-Liver/HTC—1.jpg]

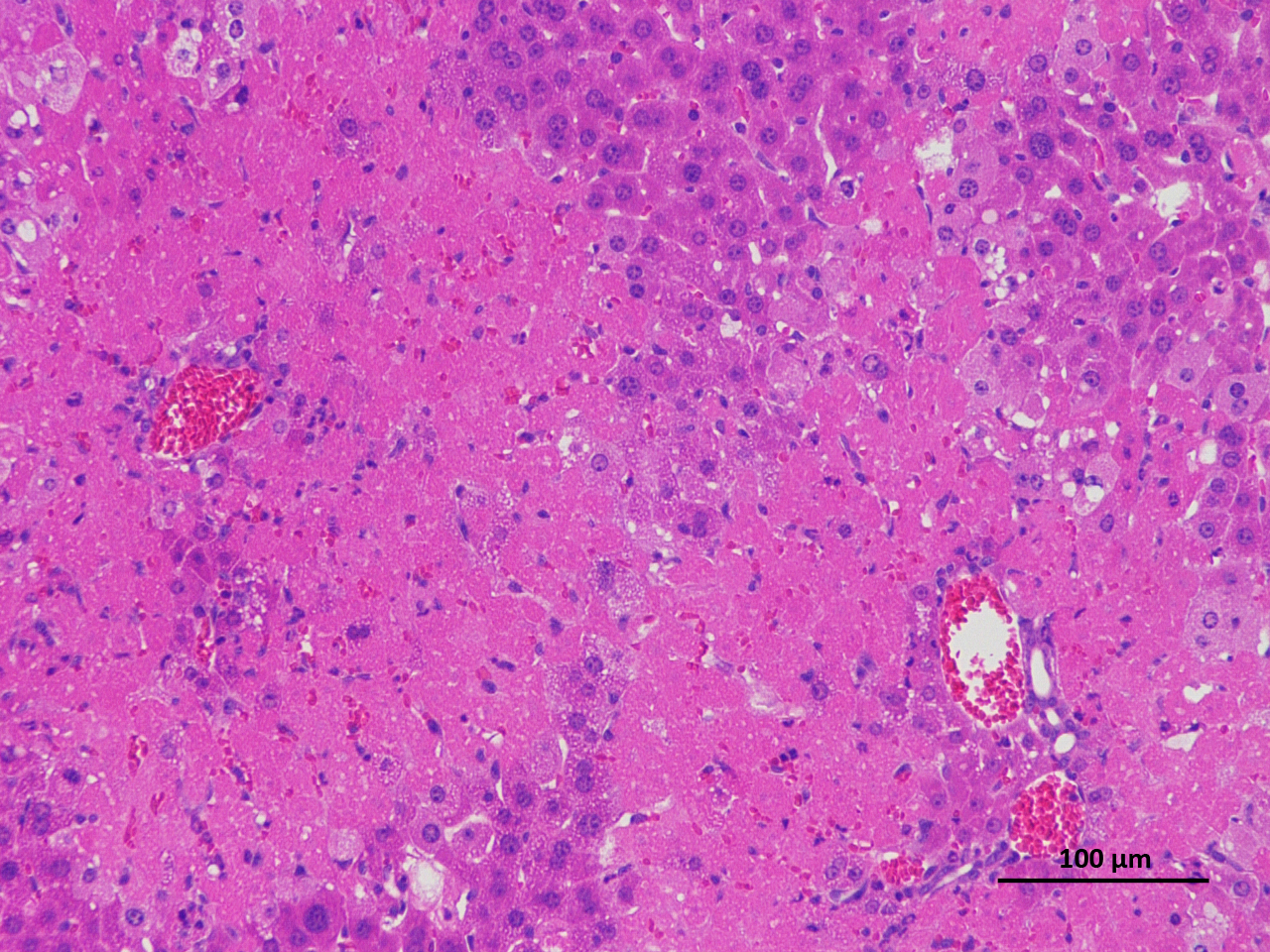

Supplement: Supplementary file 1 [file toxins-18-00278-s001.zip › Figure S11. Uncropped full-size histopathological micrographs of liver tissues corresponding to Figure 14B/Figure-14B-Liver/HTC—2.jpg]

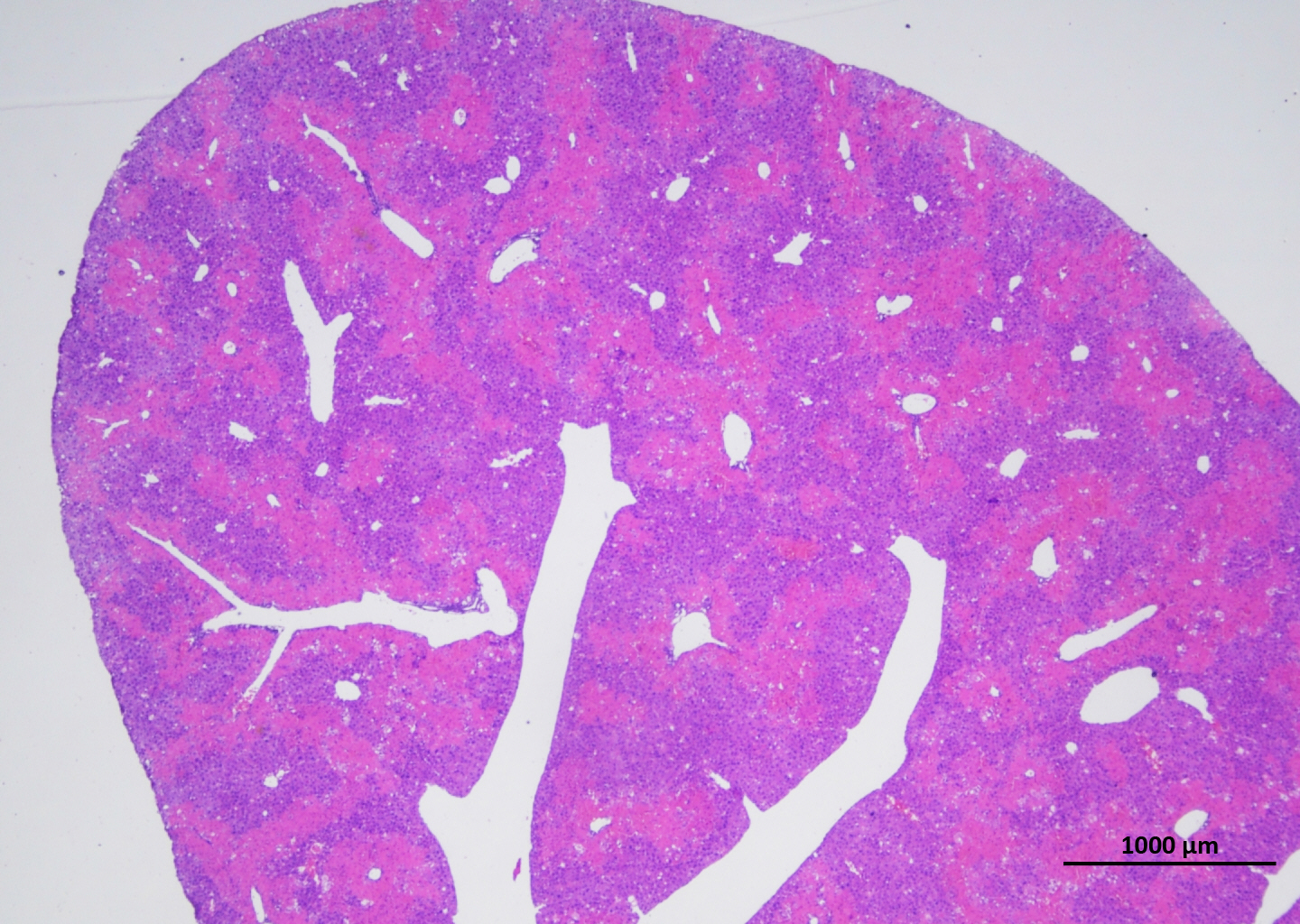

Supplement: Supplementary file 1 [file toxins-18-00278-s001.zip › Figure S11. Uncropped full-size histopathological micrographs of liver tissues corresponding to Figure 14B/Figure-14B-Liver/PBS—1.jpg]

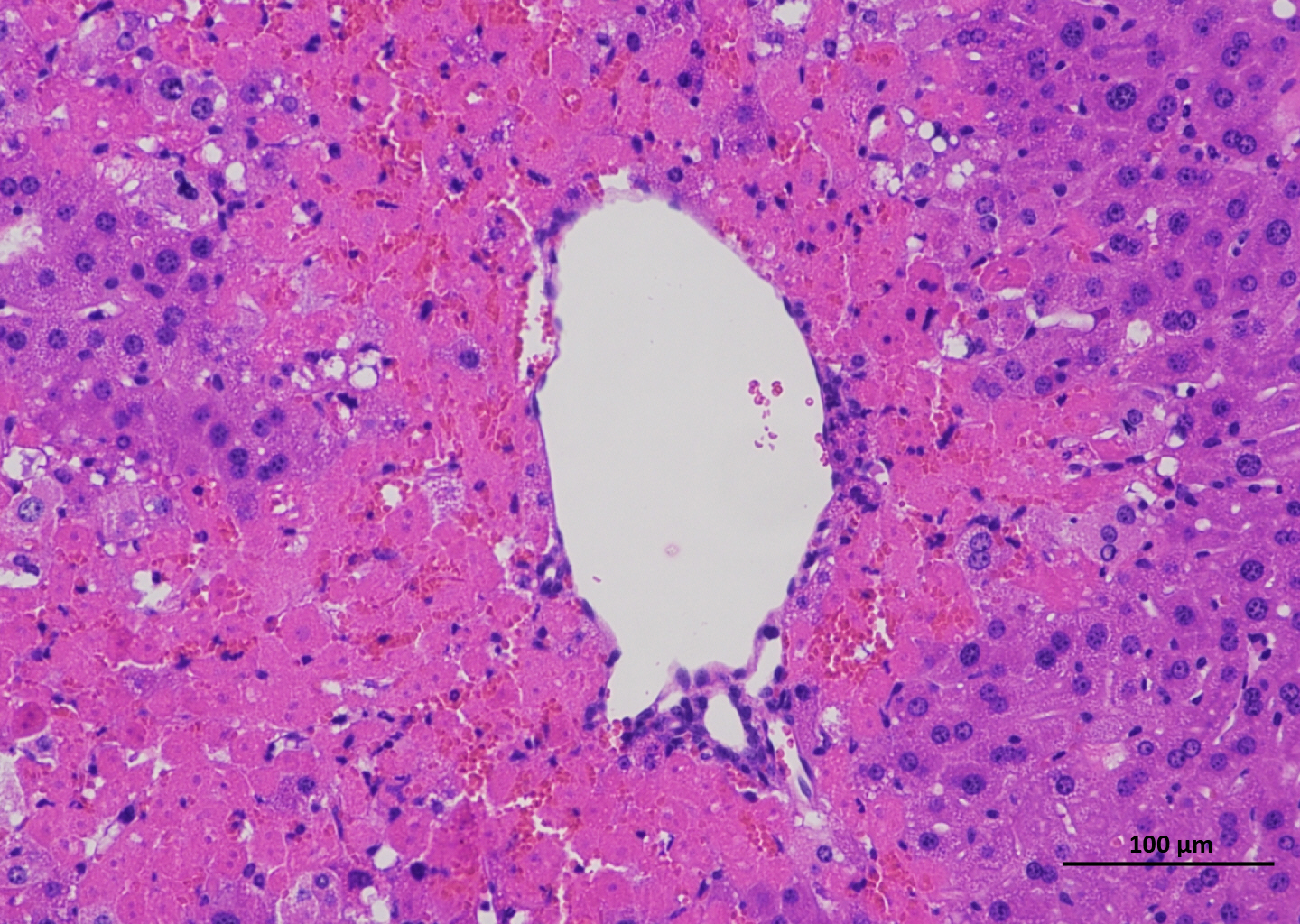

Supplement: Supplementary file 1 [file toxins-18-00278-s001.zip › Figure S11. Uncropped full-size histopathological micrographs of liver tissues corresponding to Figure 14B/Figure-14B-Liver/PBS—2.jpg]

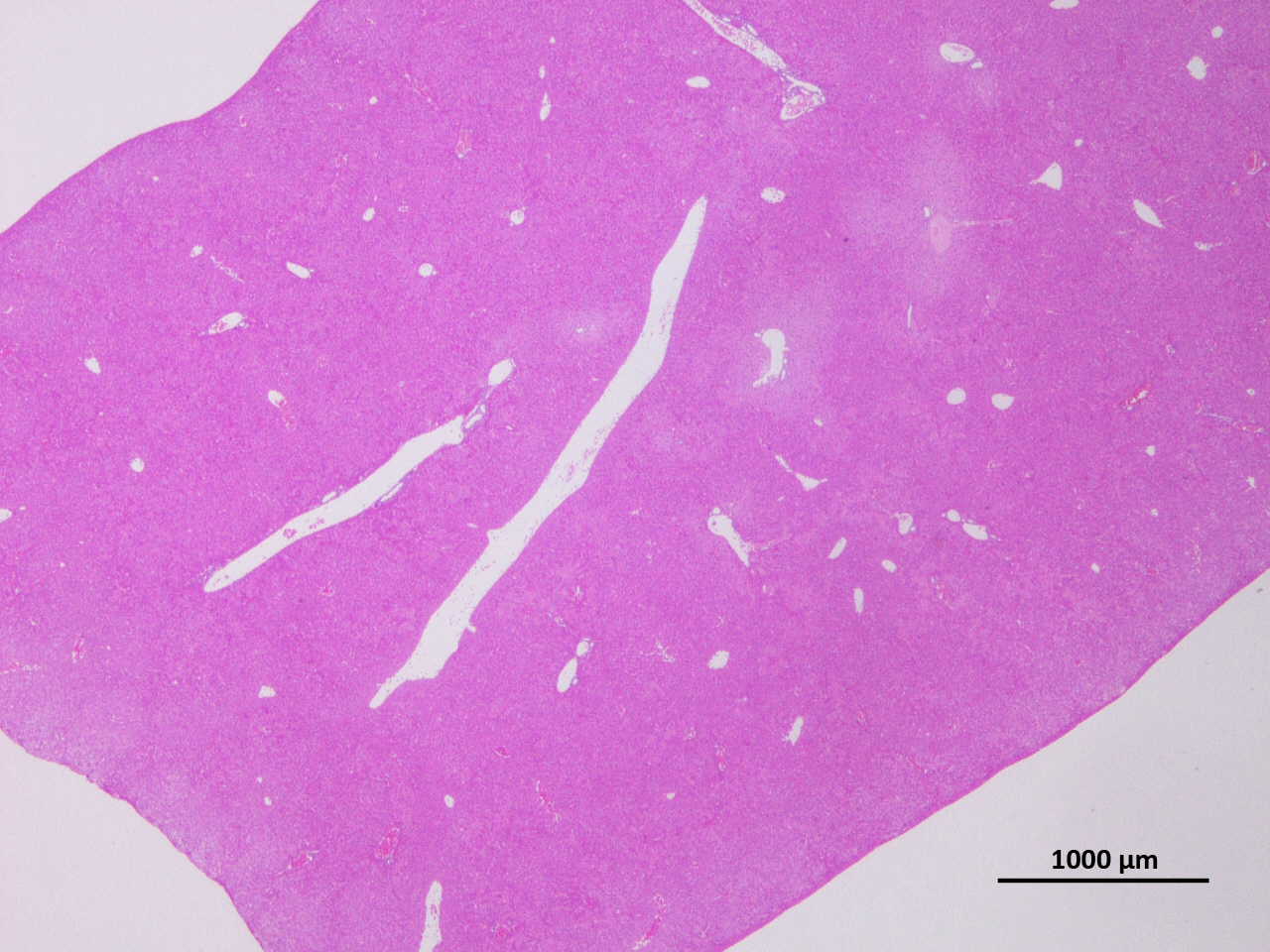

Supplement: Supplementary file 1 [file toxins-18-00278-s001.zip › Figure S11. Uncropped full-size histopathological micrographs of liver tissues corresponding to Figure 14B/Figure-14B-Liver/PEG-EGCG—1.jpg]

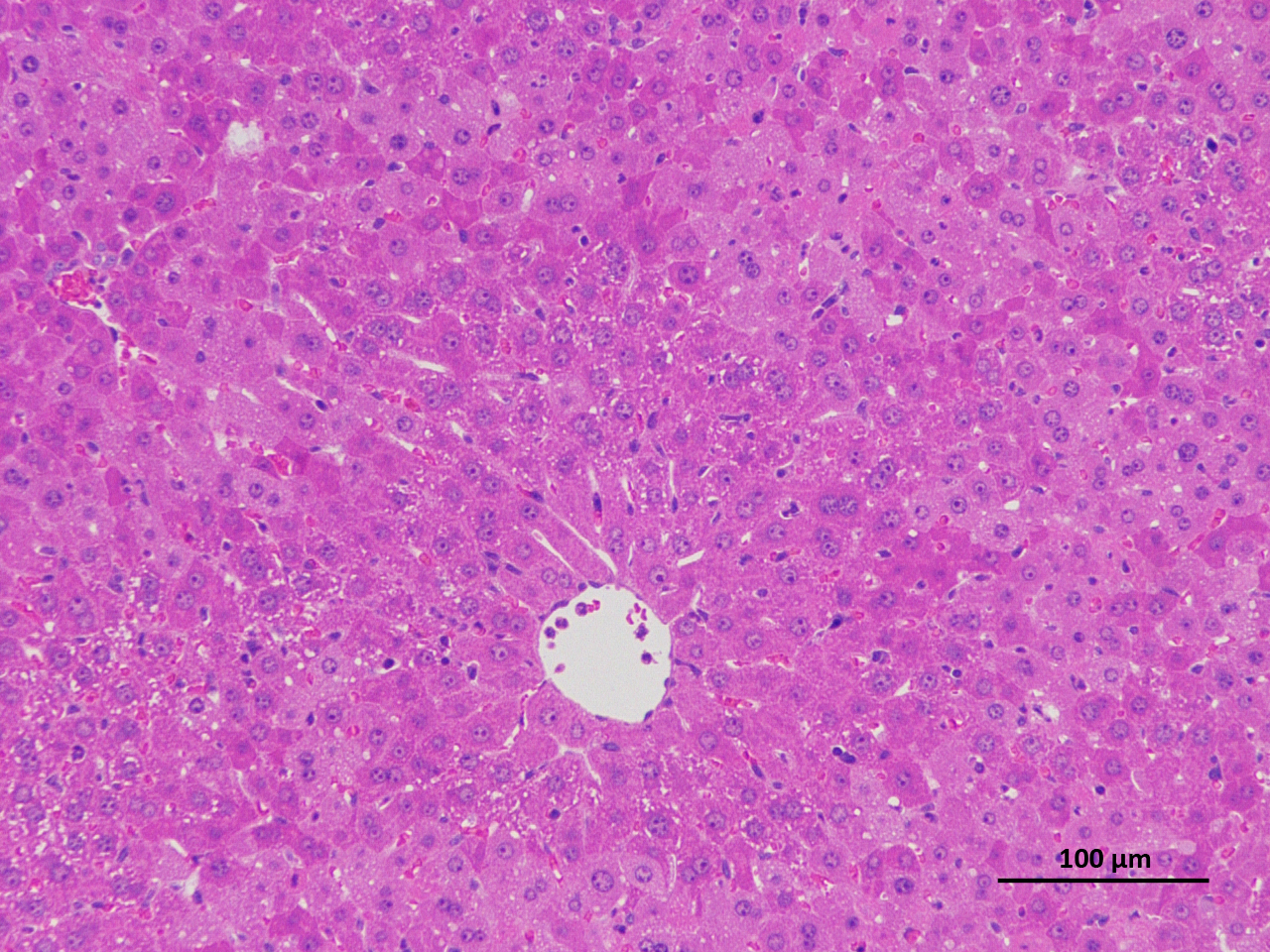

Supplement: Supplementary file 1 [file toxins-18-00278-s001.zip › Figure S11. Uncropped full-size histopathological micrographs of liver tissues corresponding to Figure 14B/Figure-14B-Liver/PEG-EGCG—2.jpg]

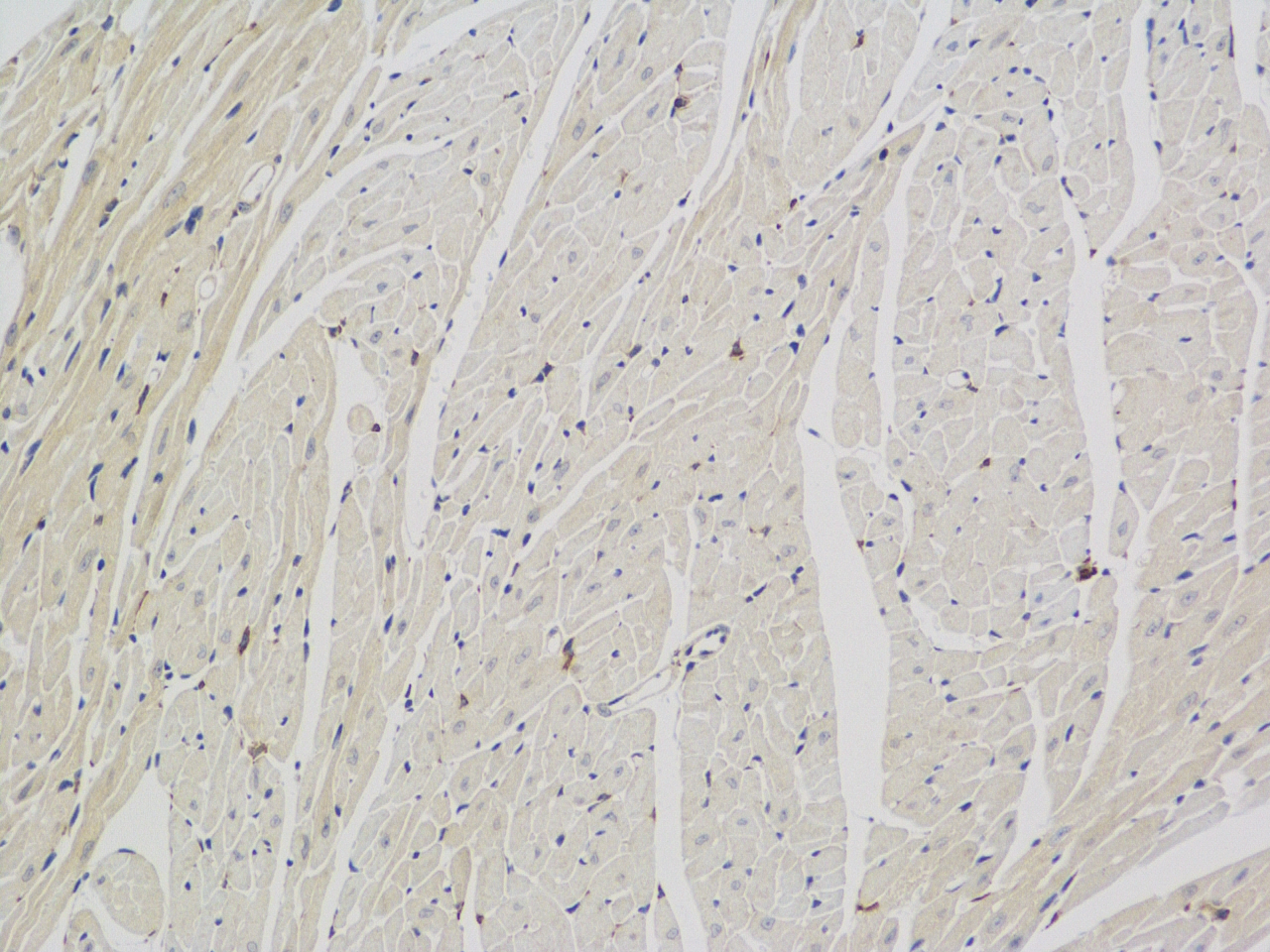

Supplement: Supplementary file 1 [file toxins-18-00278-s001.zip › Figure S5. Uncropped original full-size cardiac tissue immunostaining images corresponding to Figure 7C/EGCG-Bax-xl.jpg]

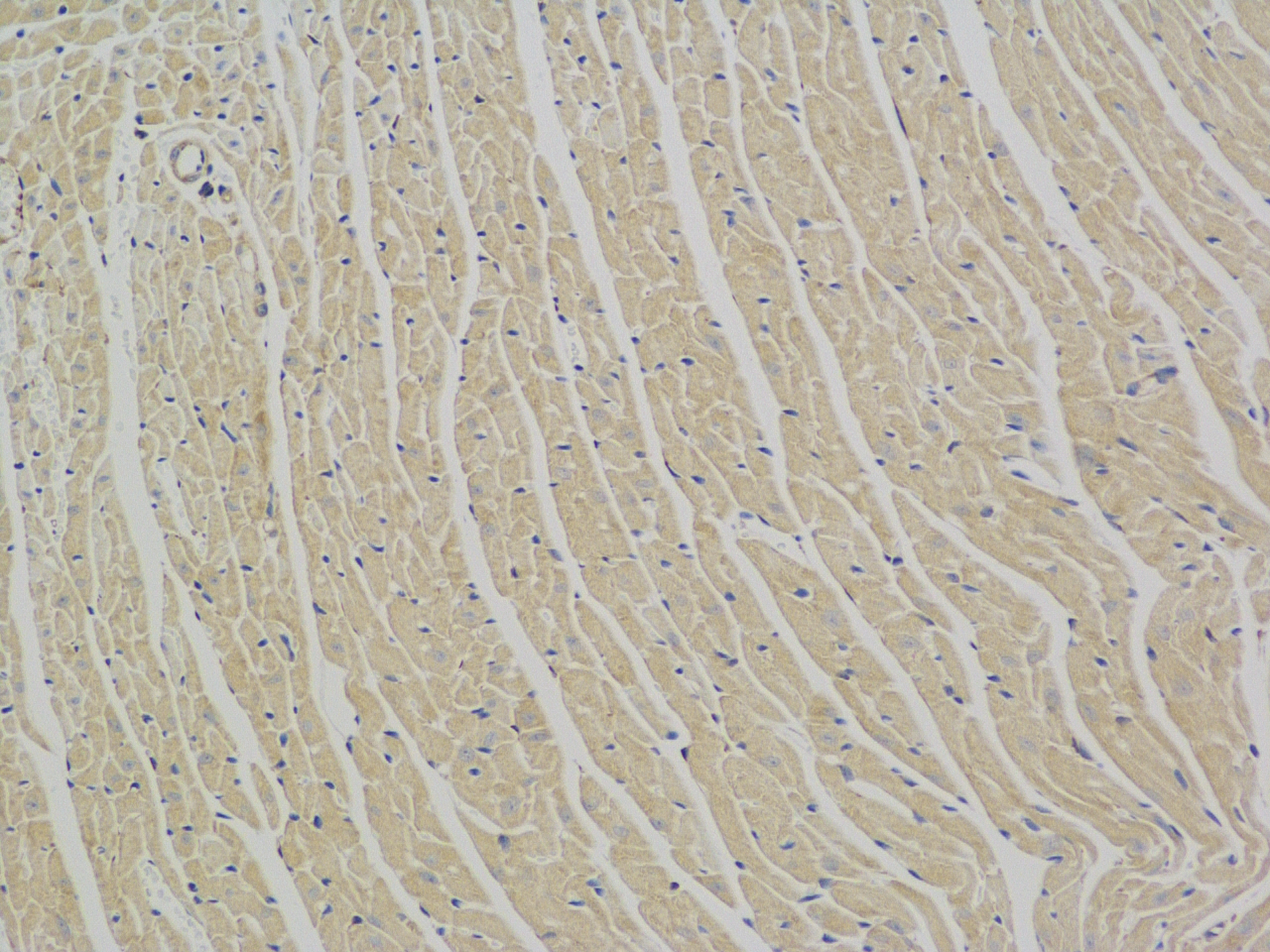

Supplement: Supplementary file 1 [file toxins-18-00278-s001.zip › Figure S5. Uncropped original full-size cardiac tissue immunostaining images corresponding to Figure 7C/EGCG-Bcl.jpg]

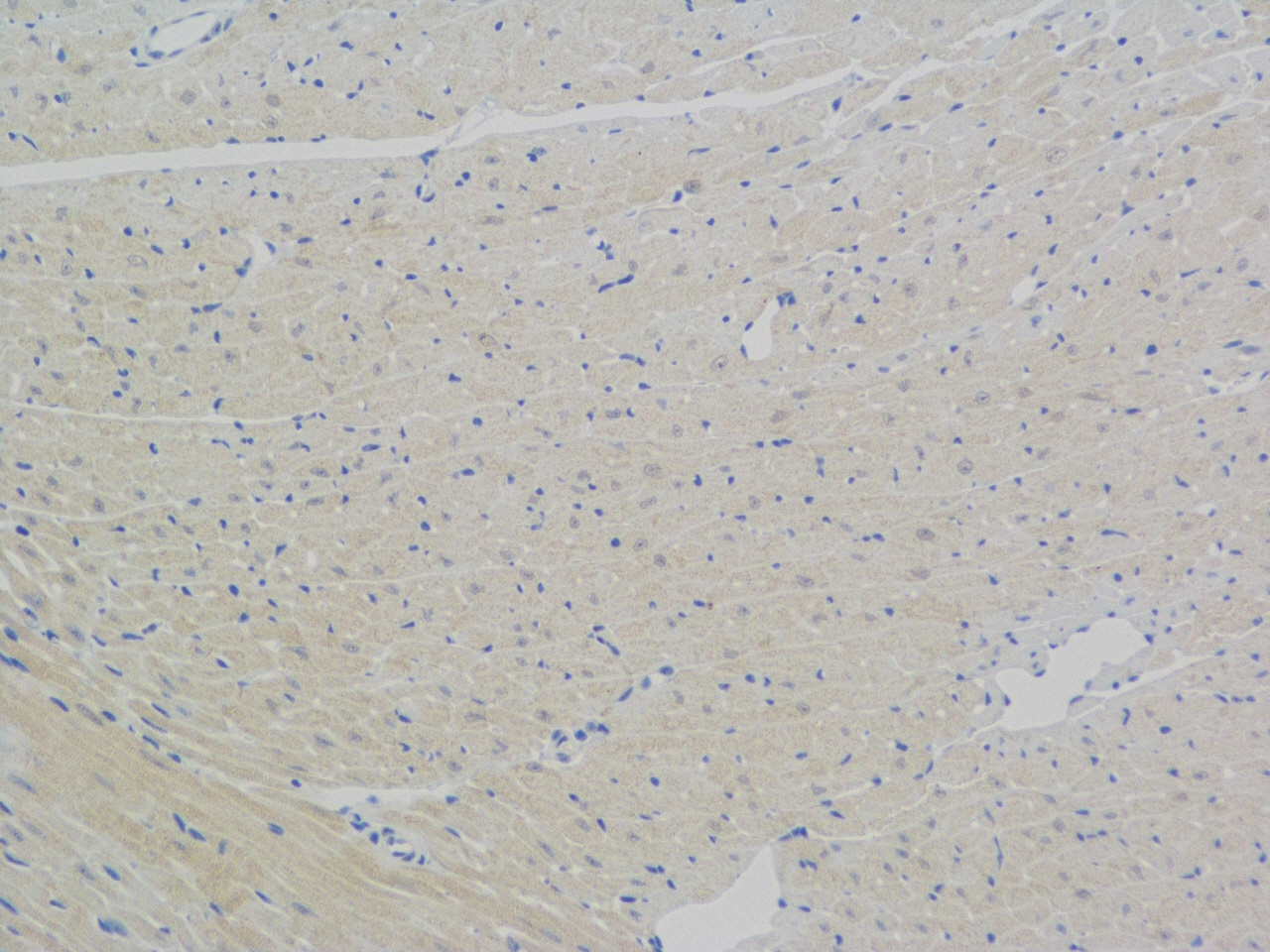

Supplement: Supplementary file 1 [file toxins-18-00278-s001.zip › Figure S5. Uncropped original full-size cardiac tissue immunostaining images corresponding to Figure 7C/EGCG-Cleaved caspase-3.jpg]

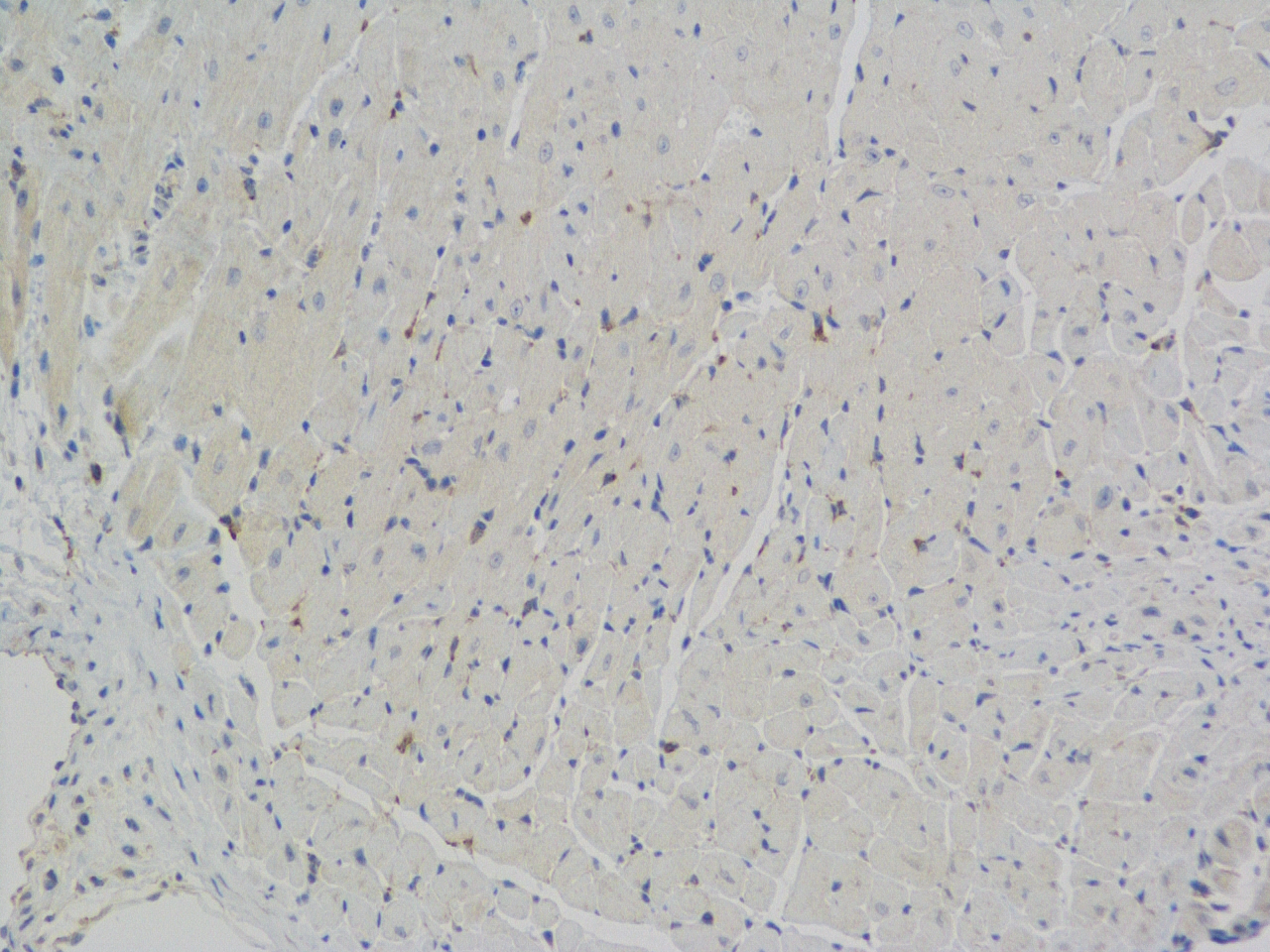

Supplement: Supplementary file 1 [file toxins-18-00278-s001.zip › Figure S5. Uncropped original full-size cardiac tissue immunostaining images corresponding to Figure 7C/EGCG-Cleaved PARP.jpg]

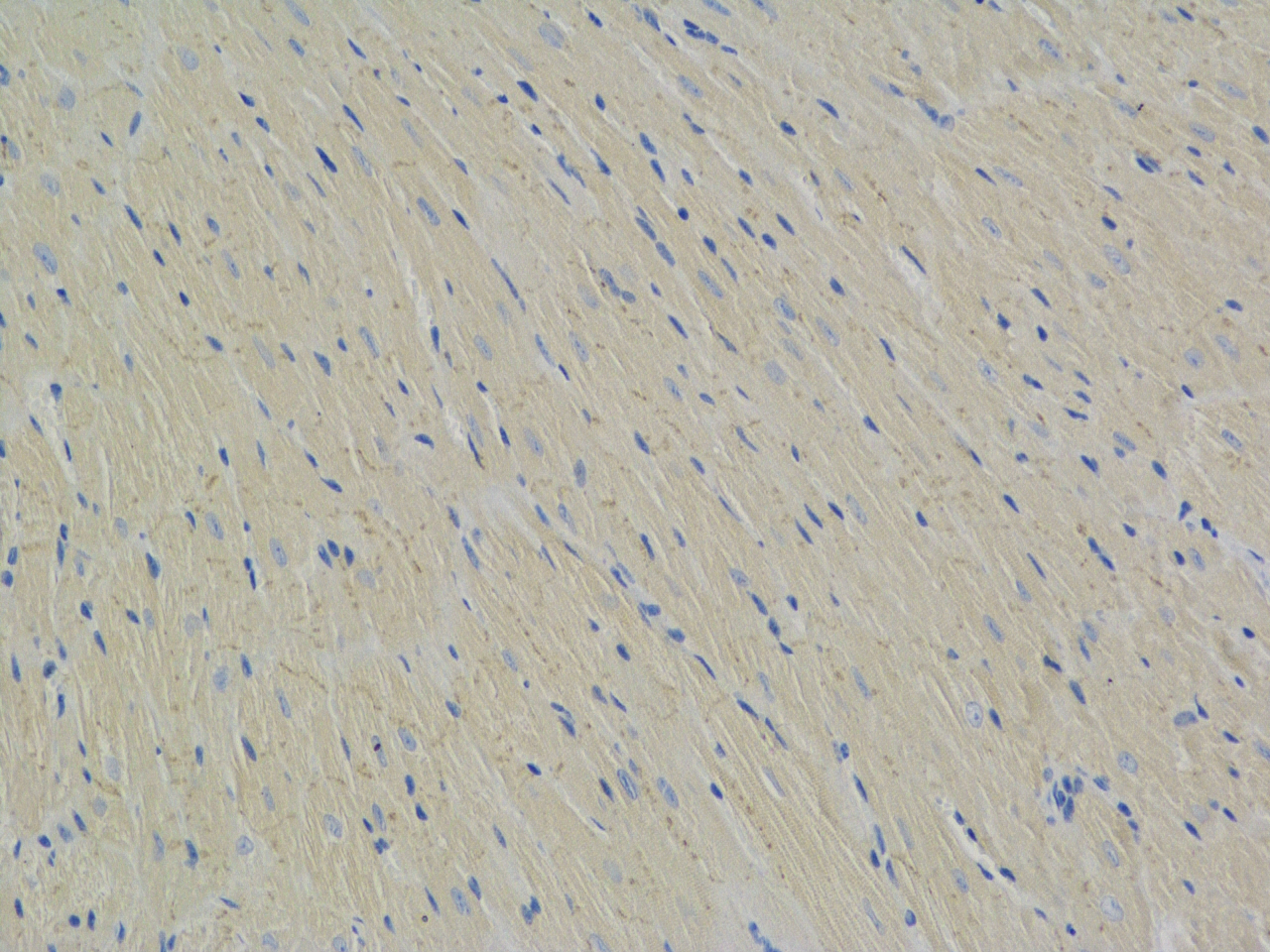

Supplement: Supplementary file 1 [file toxins-18-00278-s001.zip › Figure S5. Uncropped original full-size cardiac tissue immunostaining images corresponding to Figure 7C/NnV+EGCG-Bax.jpg]

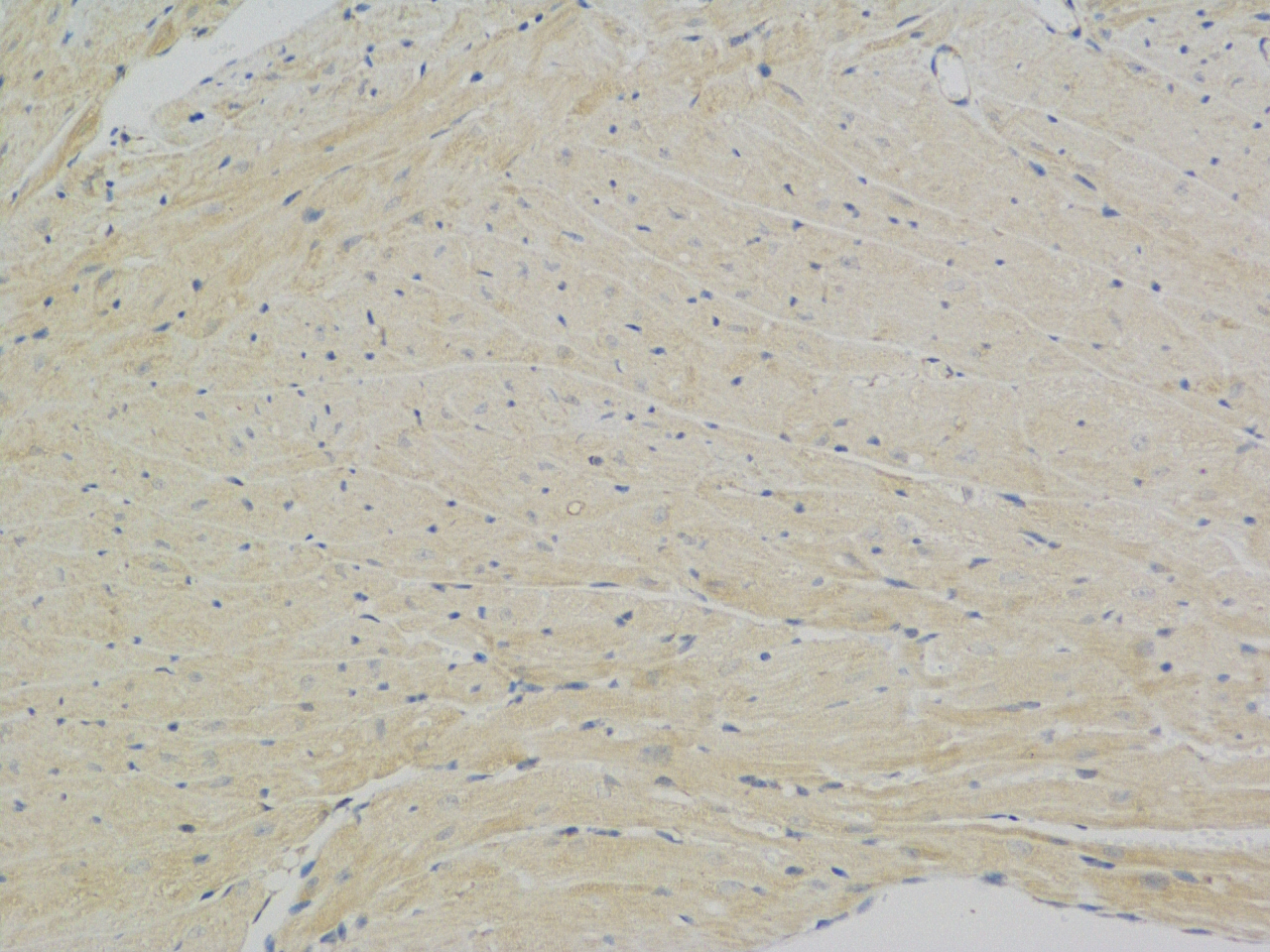

Supplement: Supplementary file 1 [file toxins-18-00278-s001.zip › Figure S5. Uncropped original full-size cardiac tissue immunostaining images corresponding to Figure 7C/NnV+EGCG-Bcl-xl.jpg]

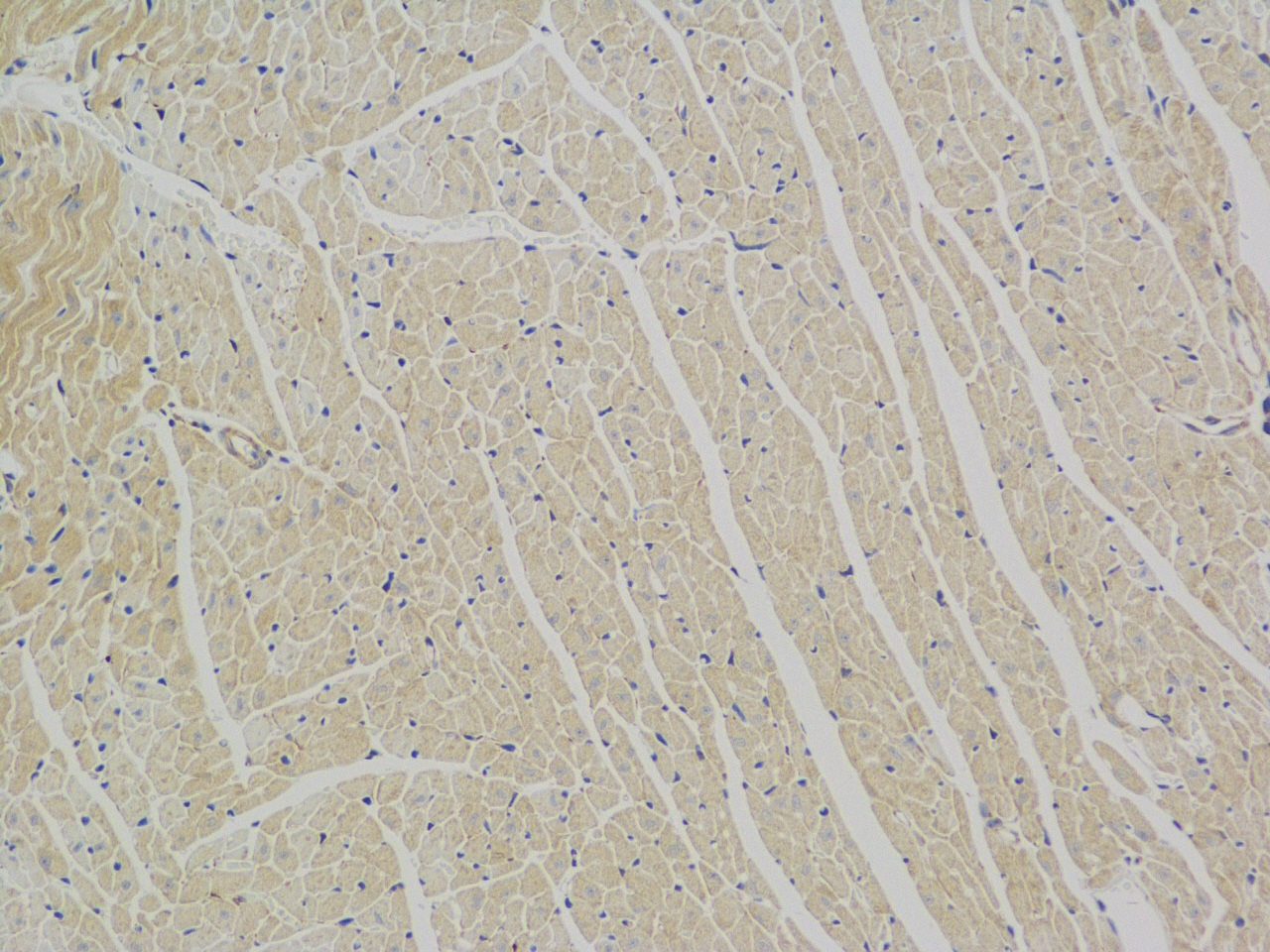

Supplement: Supplementary file 1 [file toxins-18-00278-s001.zip › Figure S5. Uncropped original full-size cardiac tissue immunostaining images corresponding to Figure 7C/NnV+EGCG-Cleaved Caspase-3.jpg]

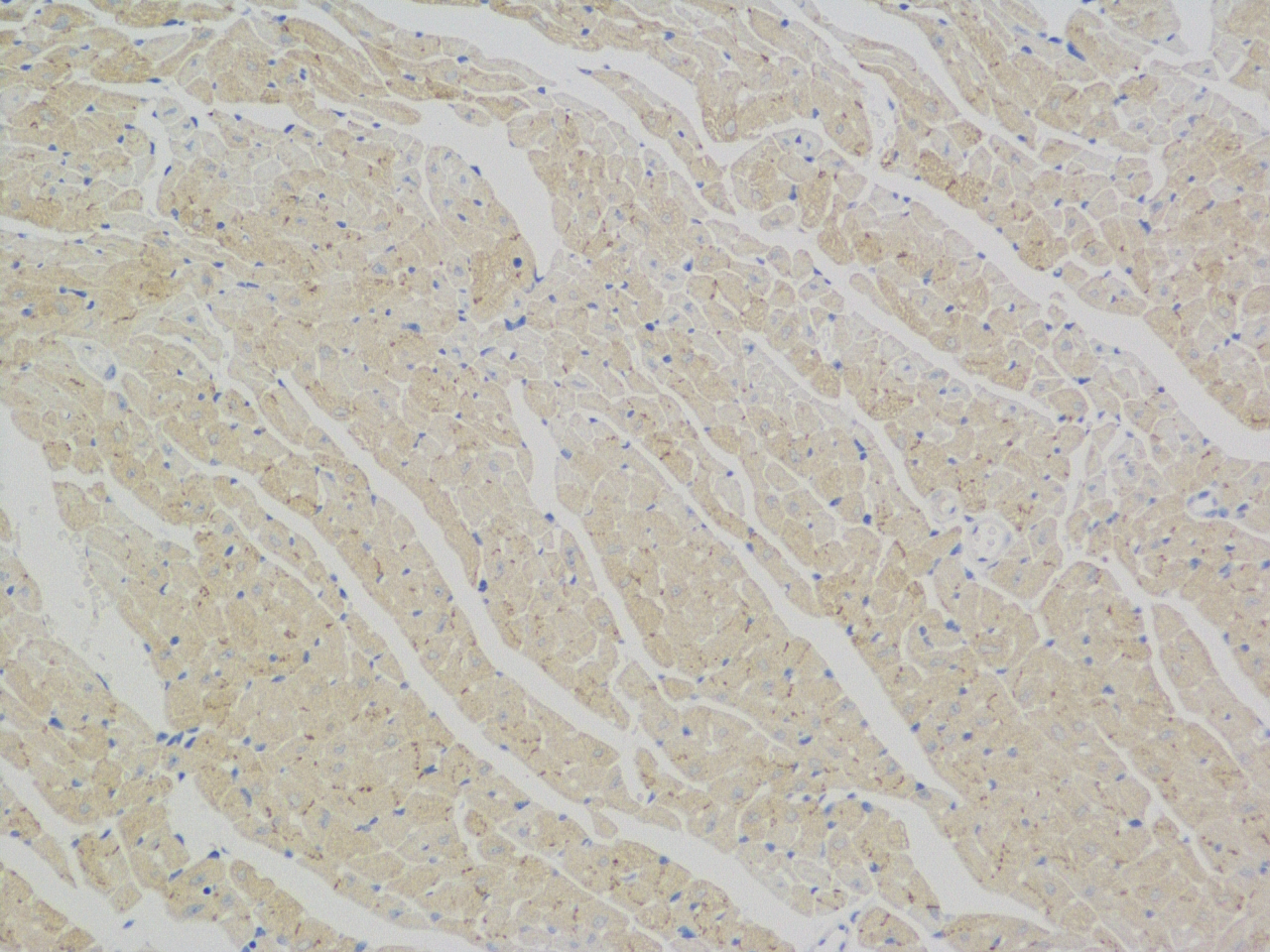

Supplement: Supplementary file 1 [file toxins-18-00278-s001.zip › Figure S5. Uncropped original full-size cardiac tissue immunostaining images corresponding to Figure 7C/NnV+EGCG-Cleaved PARP.jpg]

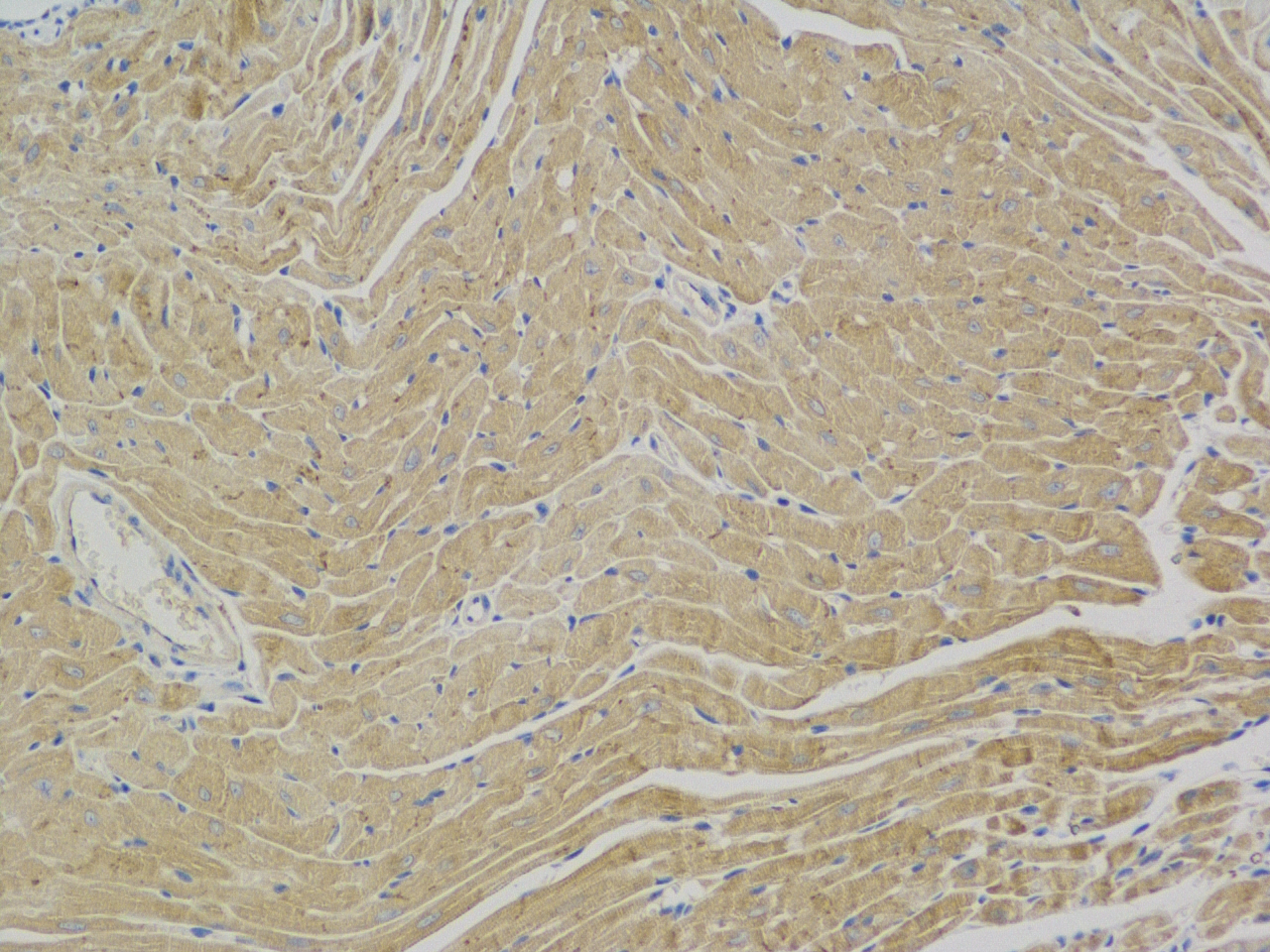

Supplement: Supplementary file 1 [file toxins-18-00278-s001.zip › Figure S5. Uncropped original full-size cardiac tissue immunostaining images corresponding to Figure 7C/NnV-Bax.jpg]

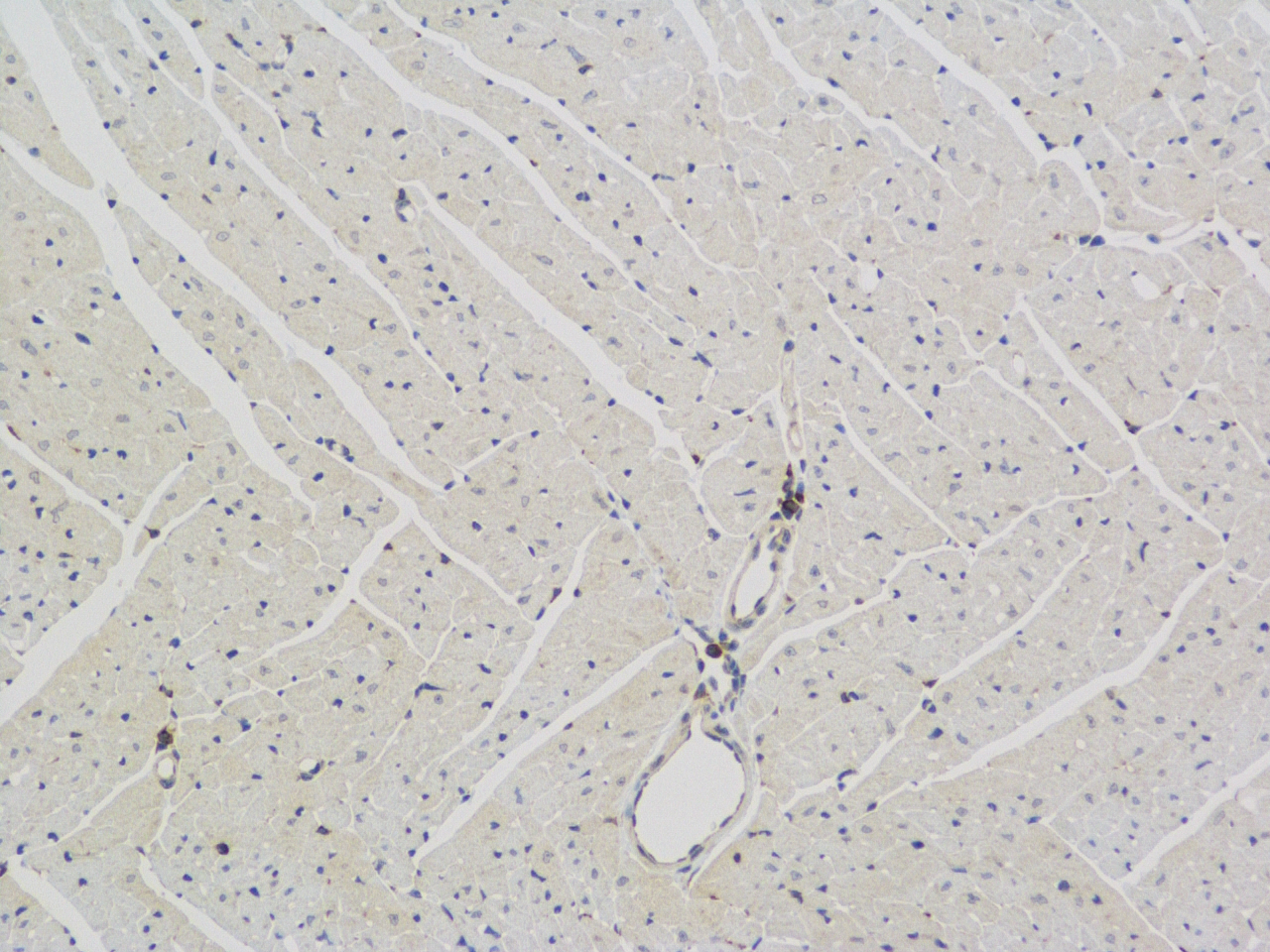

Supplement: Supplementary file 1 [file toxins-18-00278-s001.zip › Figure S5. Uncropped original full-size cardiac tissue immunostaining images corresponding to Figure 7C/NnV-Bcl-xl.jpg]

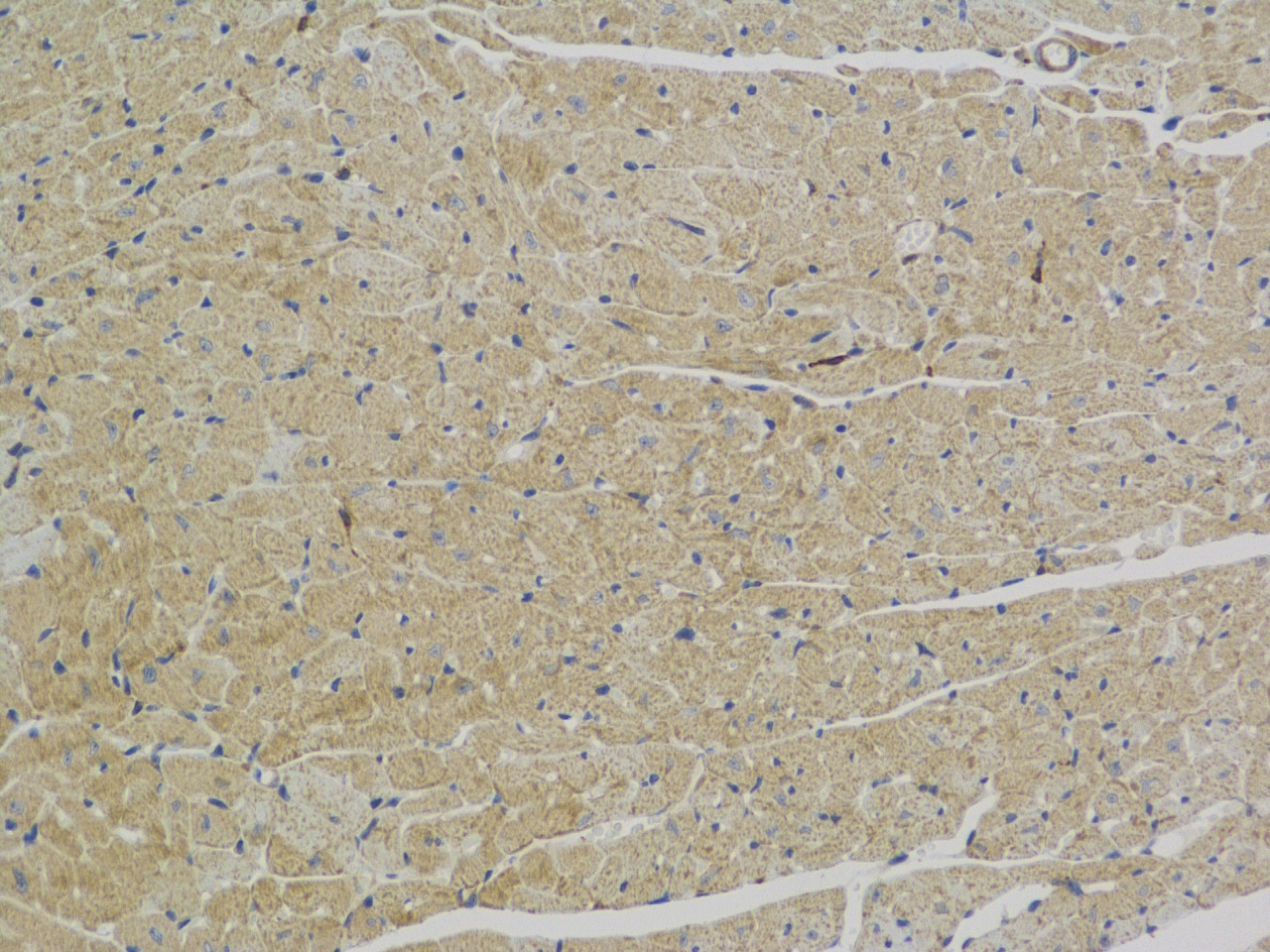

Supplement: Supplementary file 1 [file toxins-18-00278-s001.zip › Figure S5. Uncropped original full-size cardiac tissue immunostaining images corresponding to Figure 7C/NnV-cleaved caspase-3.jpg]

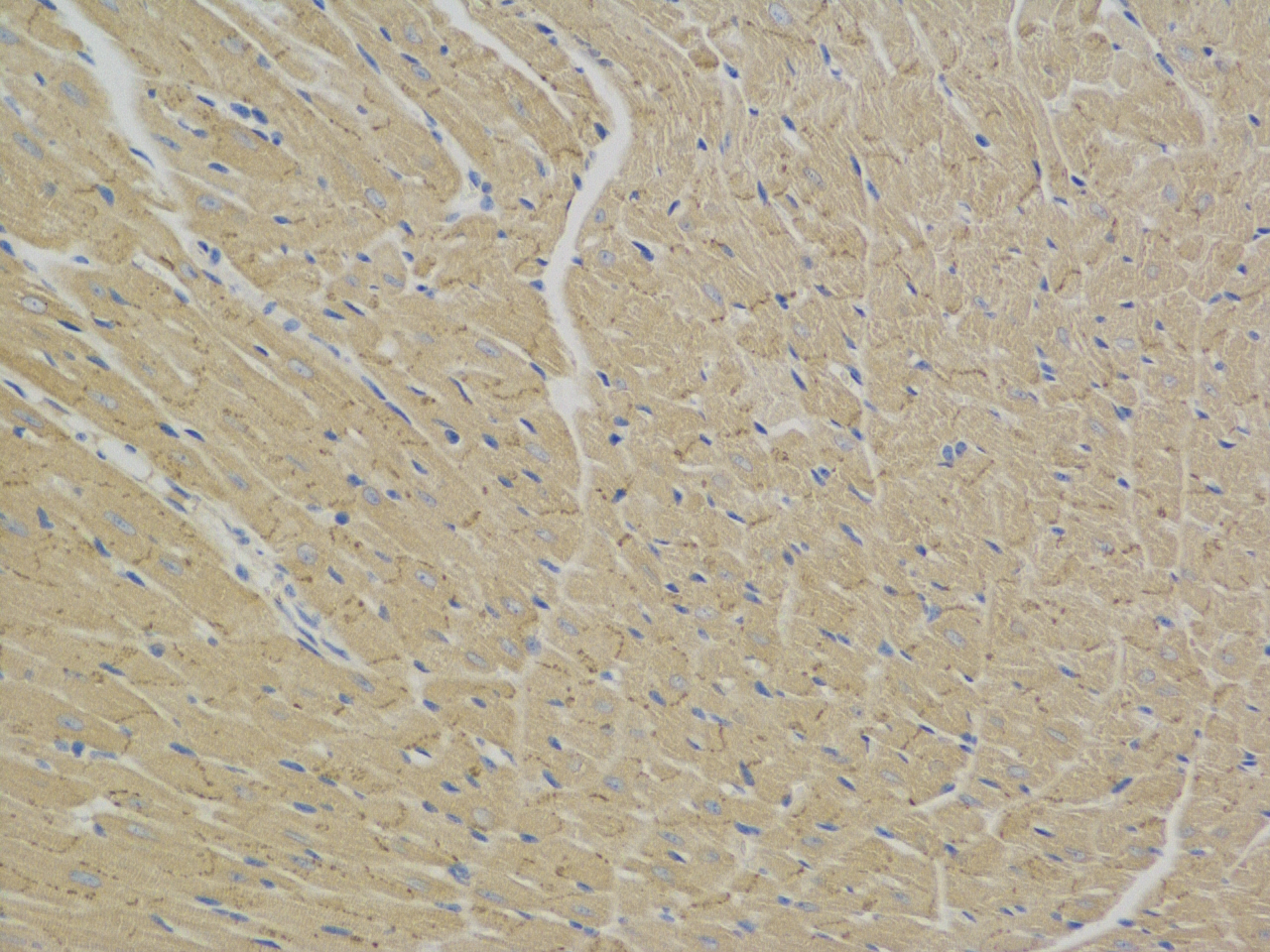

Supplement: Supplementary file 1 [file toxins-18-00278-s001.zip › Figure S5. Uncropped original full-size cardiac tissue immunostaining images corresponding to Figure 7C/NnV-cleaved PARP.jpg]

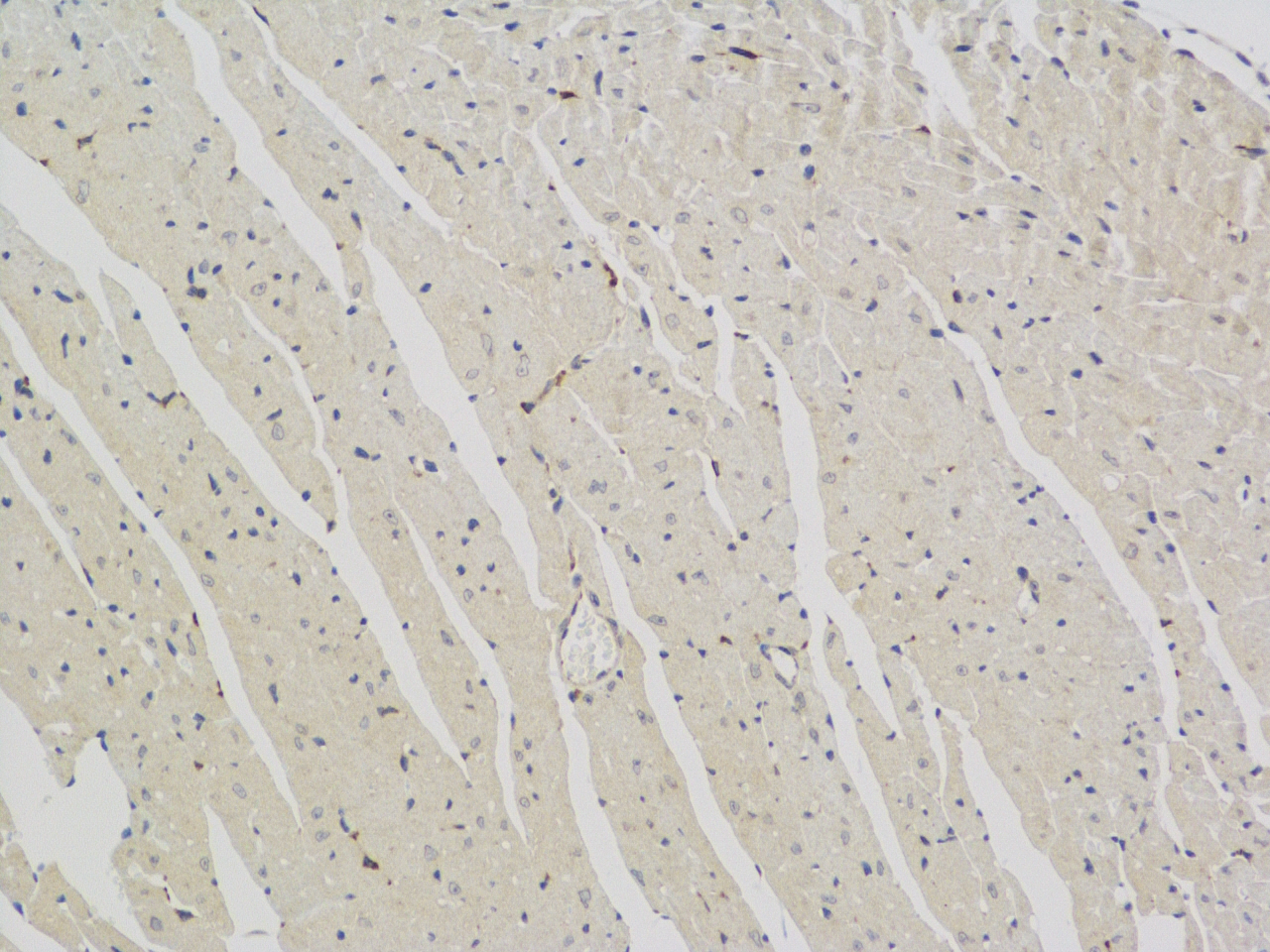

Supplement: Supplementary file 1 [file toxins-18-00278-s001.zip › Figure S5. Uncropped original full-size cardiac tissue immunostaining images corresponding to Figure 7C/PBS-Bax.jpg]

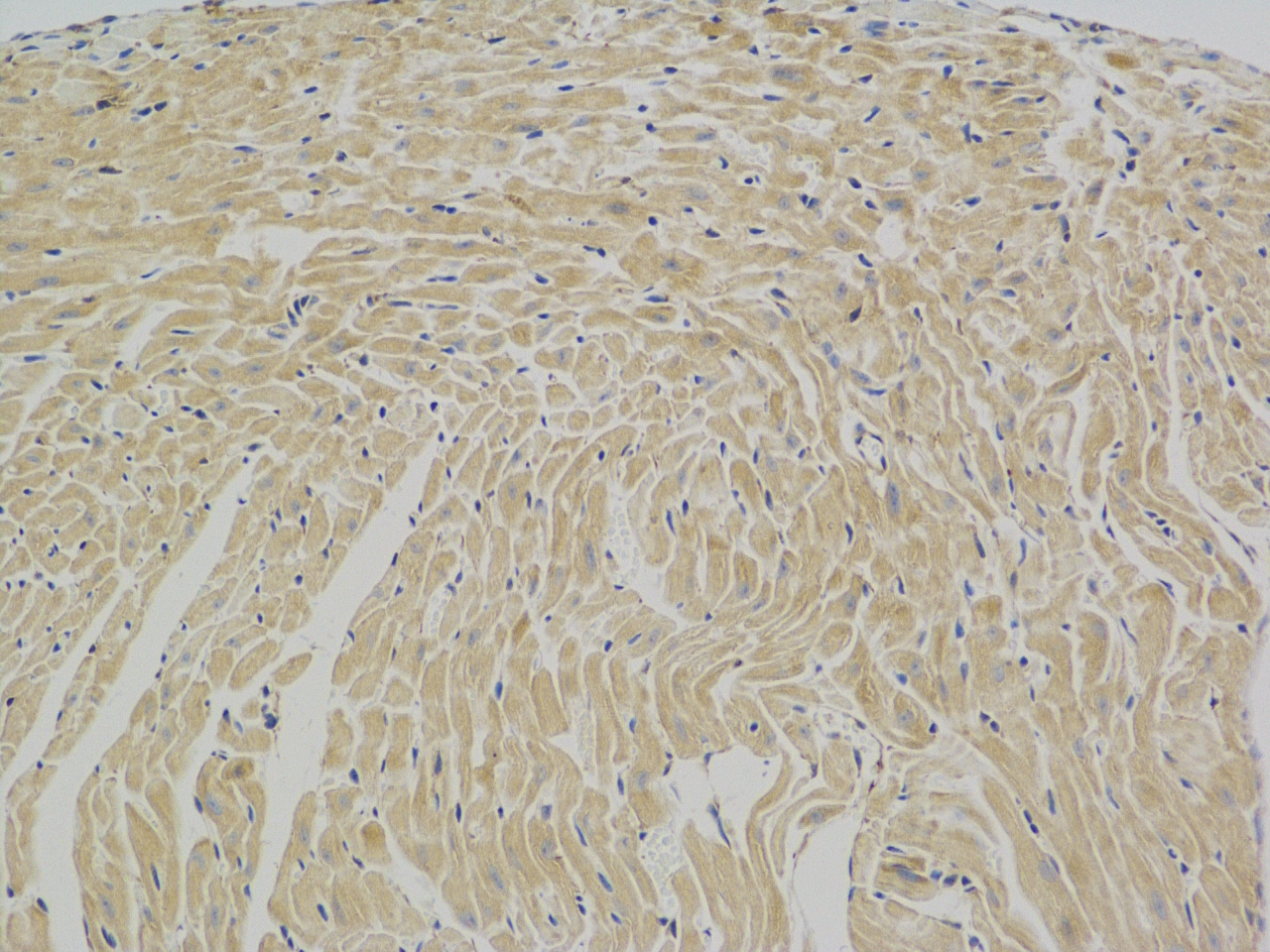

Supplement: Supplementary file 1 [file toxins-18-00278-s001.zip › Figure S5. Uncropped original full-size cardiac tissue immunostaining images corresponding to Figure 7C/PBS-Bcl-xl.jpg]

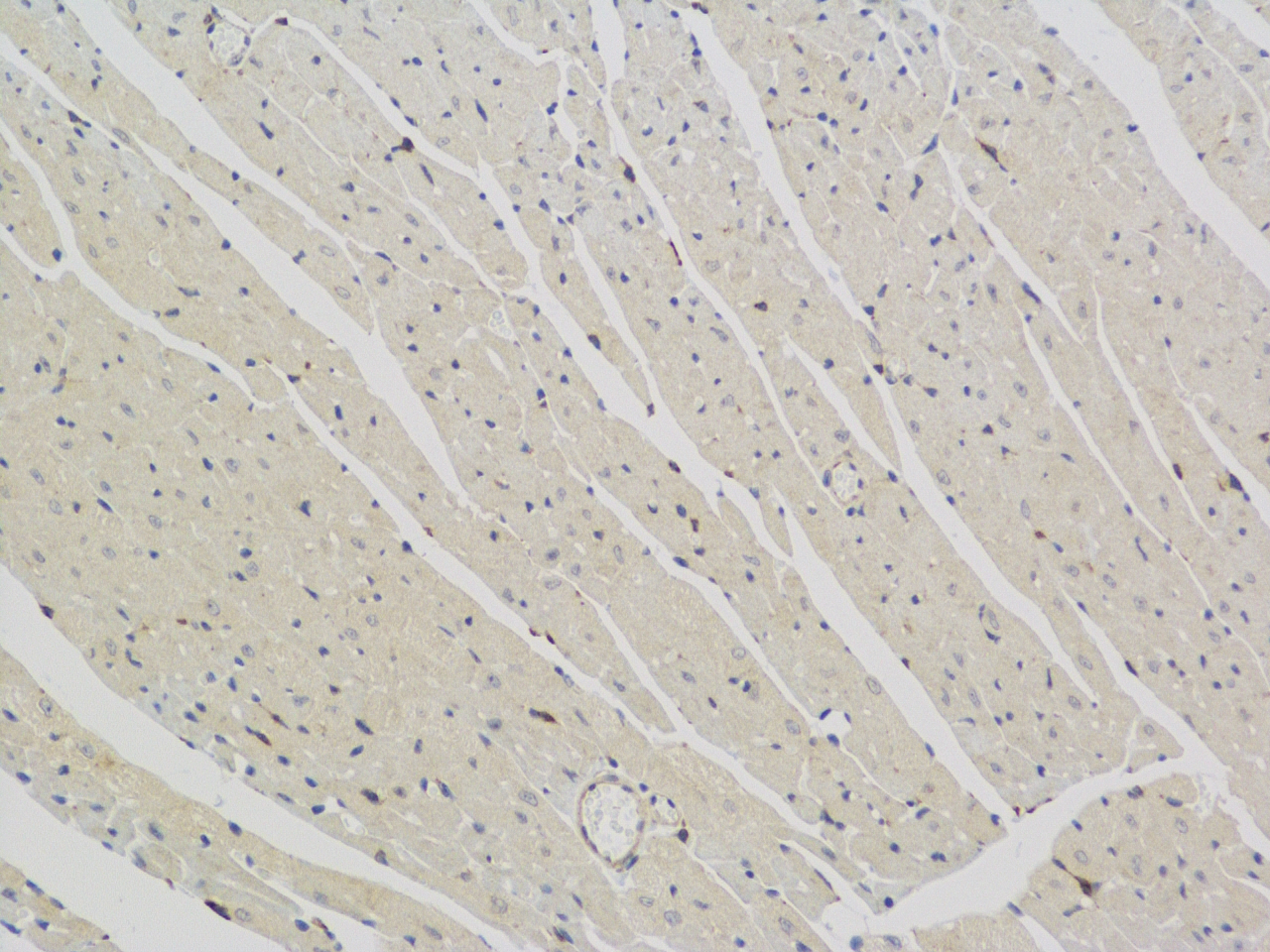

Supplement: Supplementary file 1 [file toxins-18-00278-s001.zip › Figure S5. Uncropped original full-size cardiac tissue immunostaining images corresponding to Figure 7C/PBS-Cleaved caspase-3.jpg]

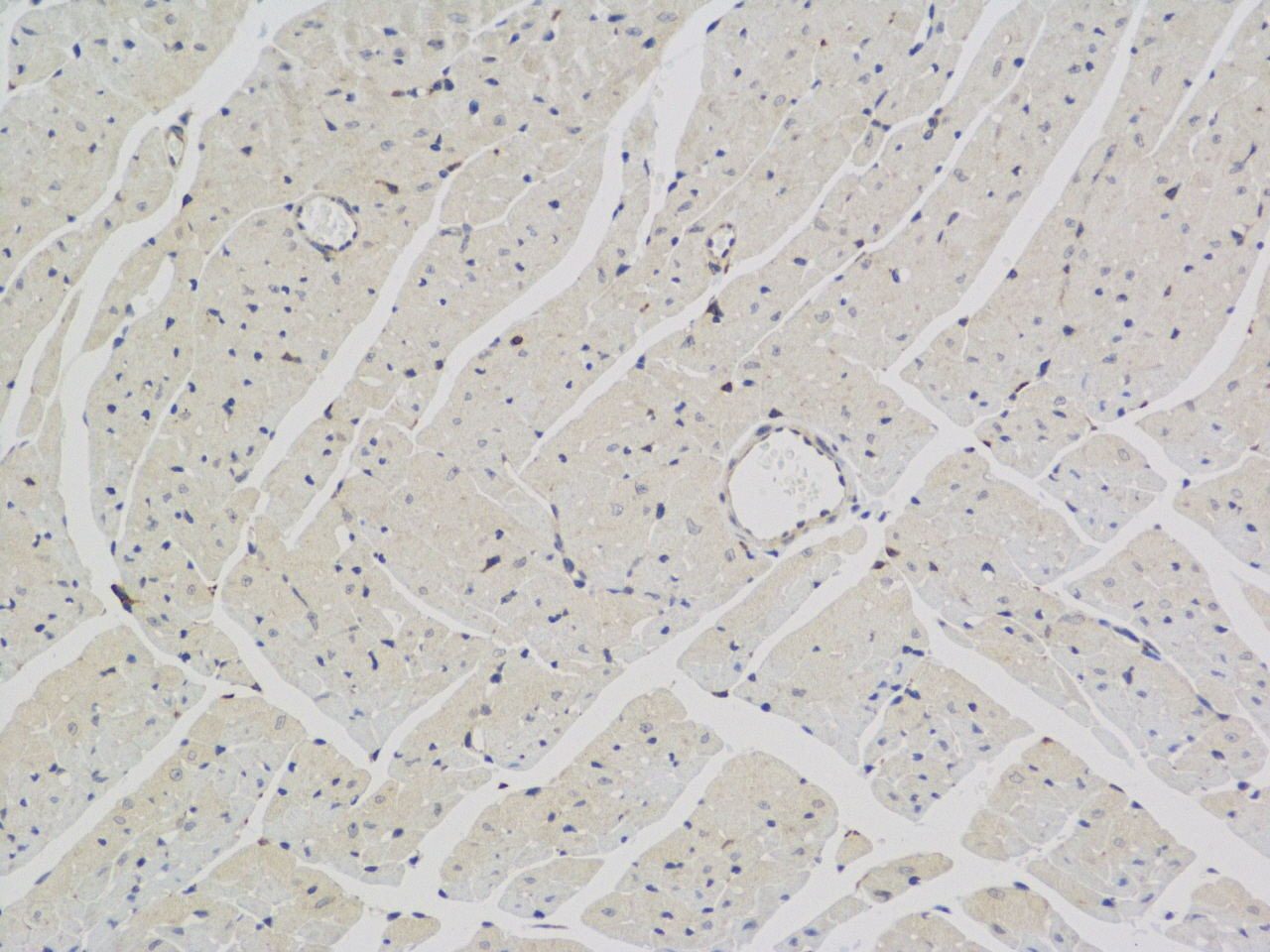

Supplement: Supplementary file 1 [file toxins-18-00278-s001.zip › Figure S5. Uncropped original full-size cardiac tissue immunostaining images corresponding to Figure 7C/PBS-Cleaved PARP.jpg]

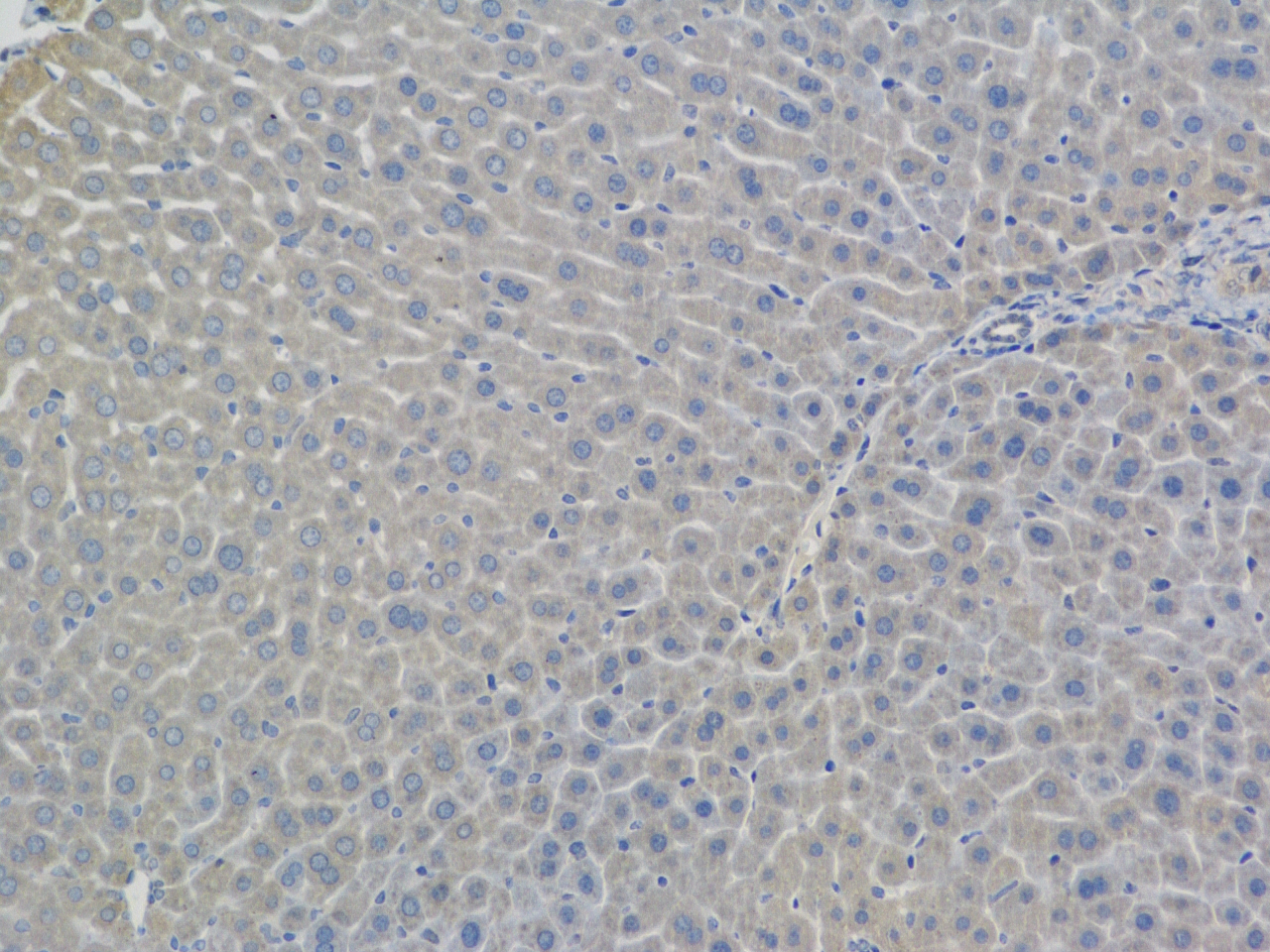

Supplement: Supplementary file 1 [file toxins-18-00278-s001.zip › Figure S6. Uncropped full-size hepatic tissue immunostaining images corresponding to Figure 8C/EGCG-Bax.jpg]

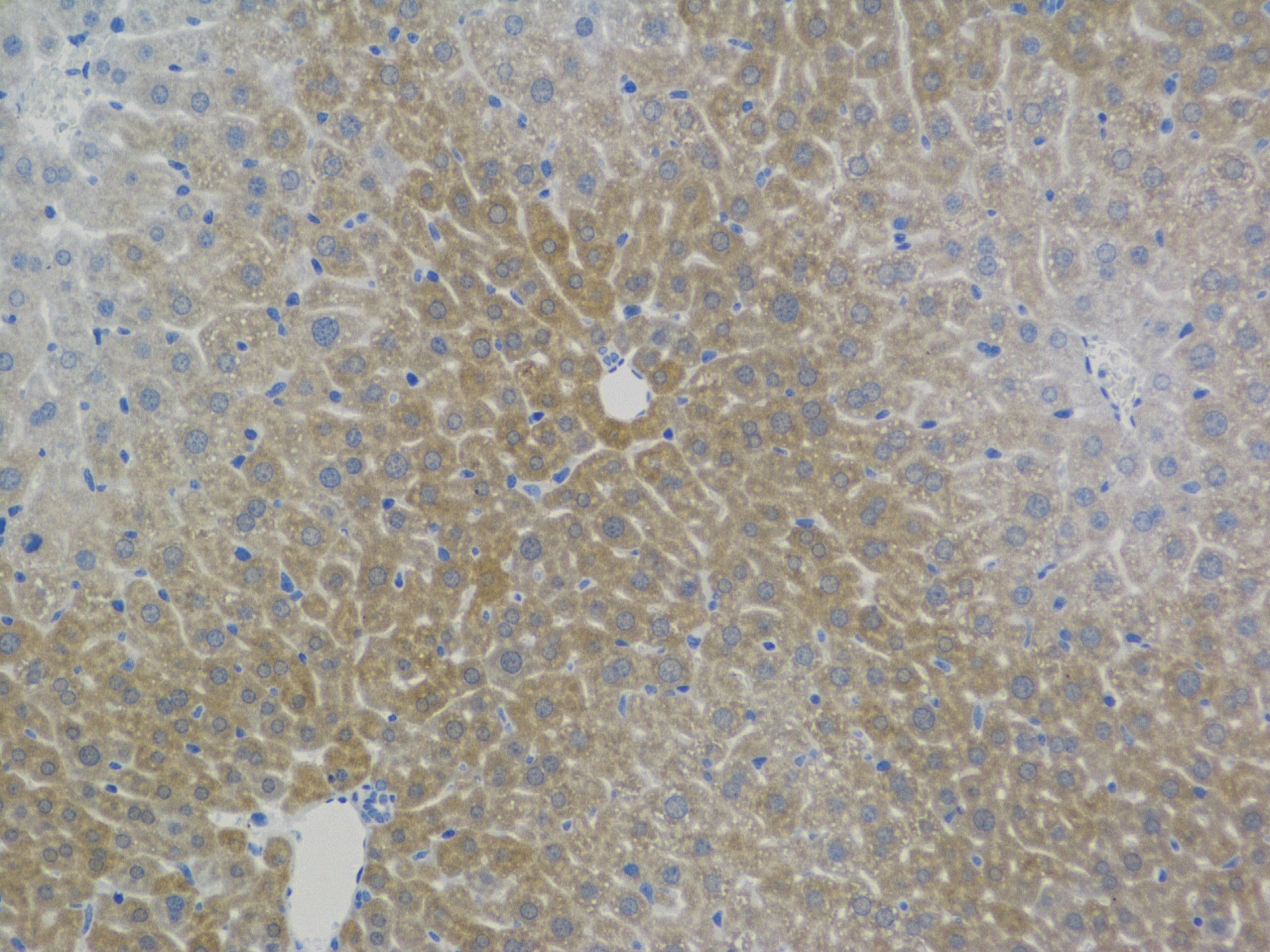

Supplement: Supplementary file 1 [file toxins-18-00278-s001.zip › Figure S6. Uncropped full-size hepatic tissue immunostaining images corresponding to Figure 8C/EGCG-Bcl-xl.jpg]

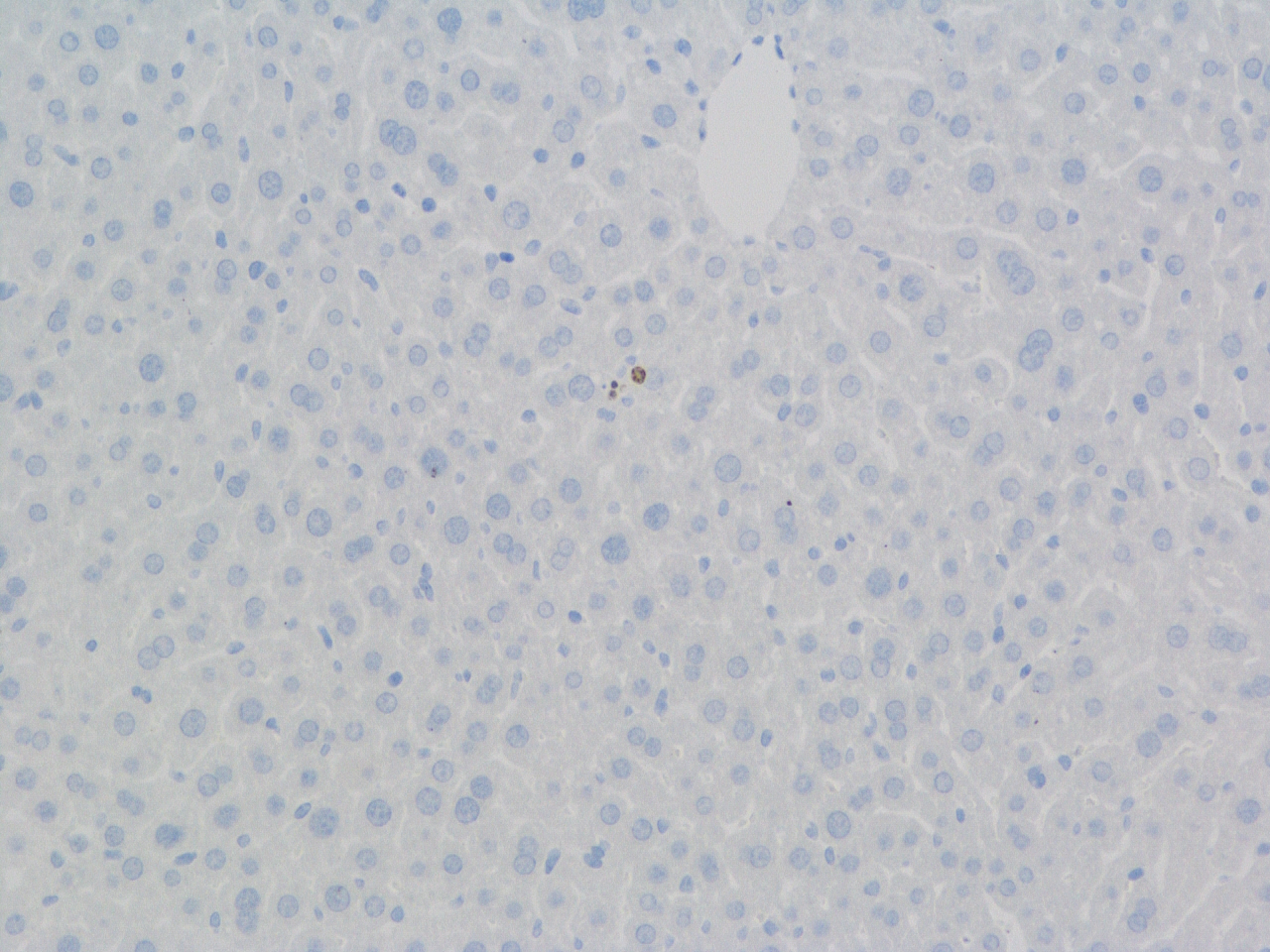

Supplement: Supplementary file 1 [file toxins-18-00278-s001.zip › Figure S6. Uncropped full-size hepatic tissue immunostaining images corresponding to Figure 8C/EGCG-Cleaved Caspase-3.jpg]

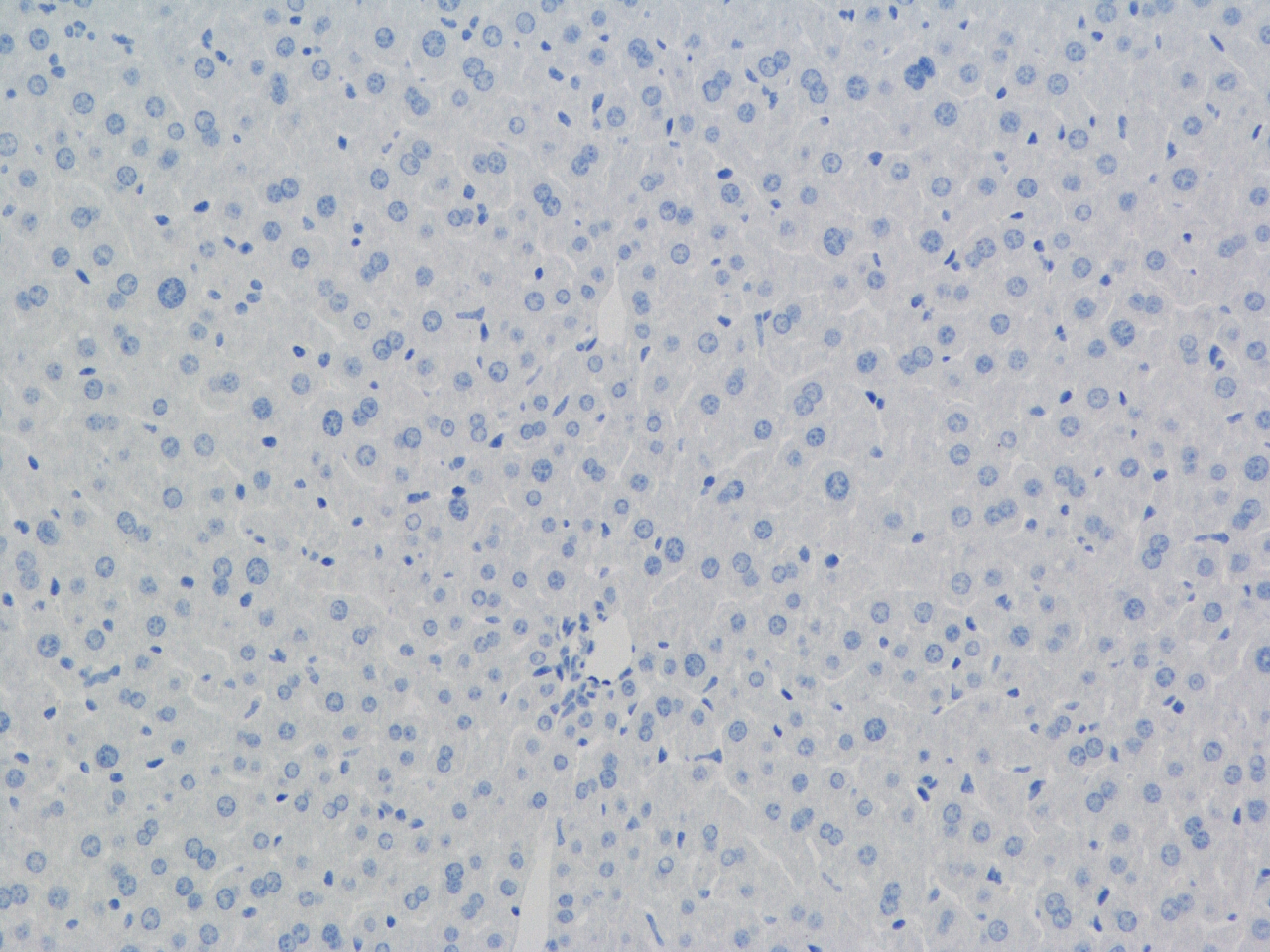

Supplement: Supplementary file 1 [file toxins-18-00278-s001.zip › Figure S6. Uncropped full-size hepatic tissue immunostaining images corresponding to Figure 8C/EGCG-Cleaved PARP.jpg]

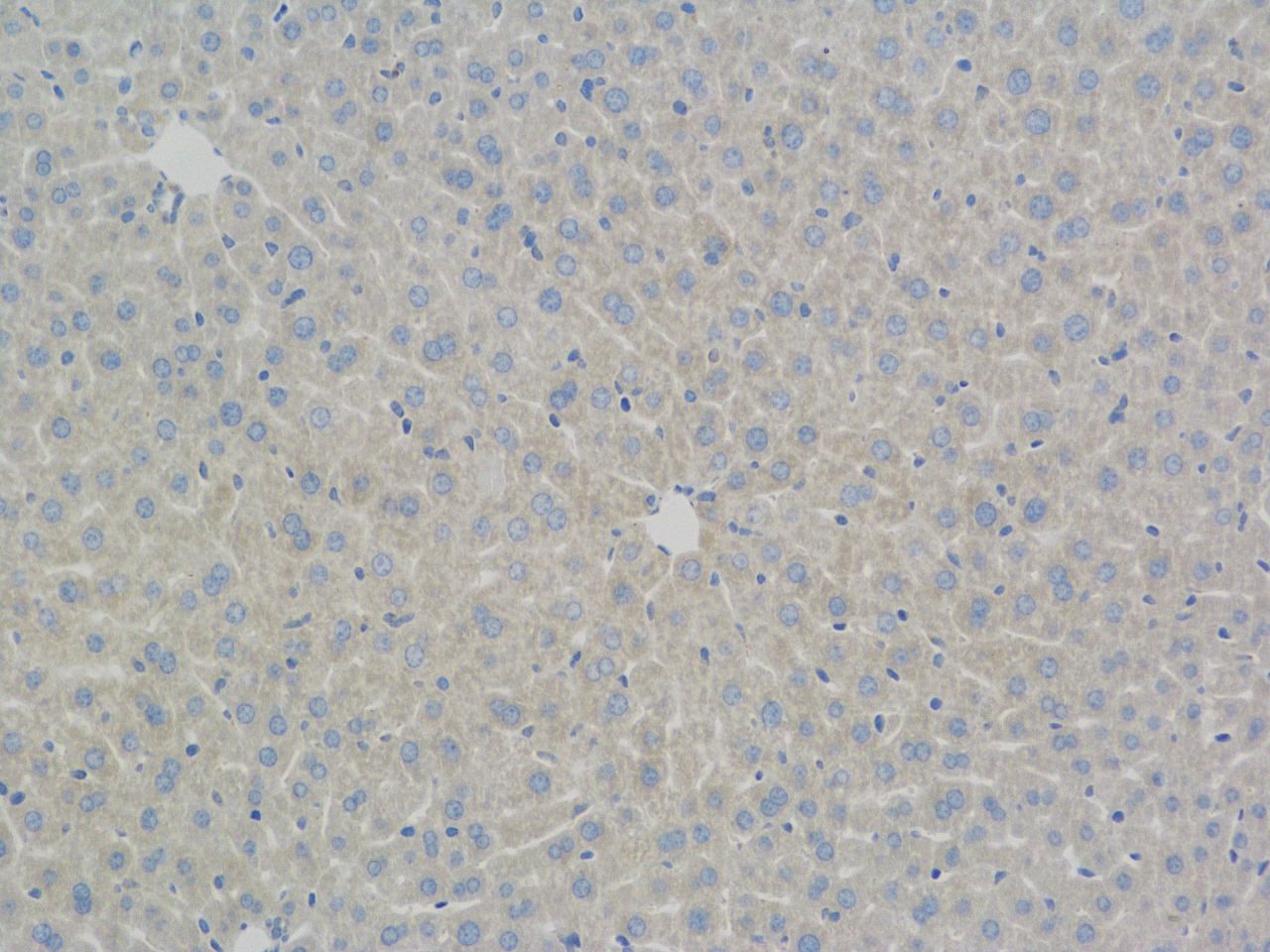

Supplement: Supplementary file 1 [file toxins-18-00278-s001.zip › Figure S6. Uncropped full-size hepatic tissue immunostaining images corresponding to Figure 8C/NnV+EGCG-Bax.jpg]

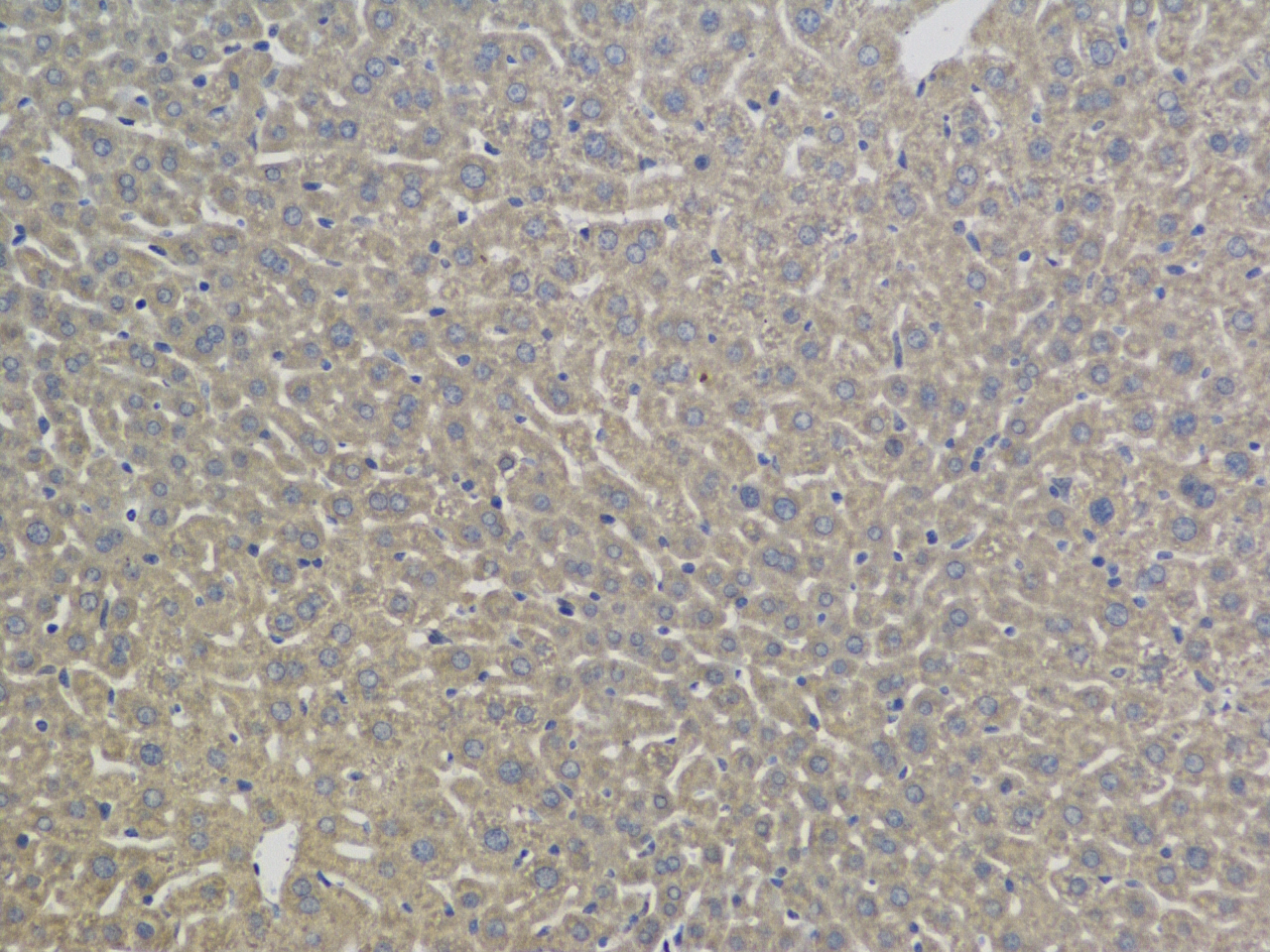

Supplement: Supplementary file 1 [file toxins-18-00278-s001.zip › Figure S6. Uncropped full-size hepatic tissue immunostaining images corresponding to Figure 8C/NnV+EGCG-Bcl-xl.jpg]

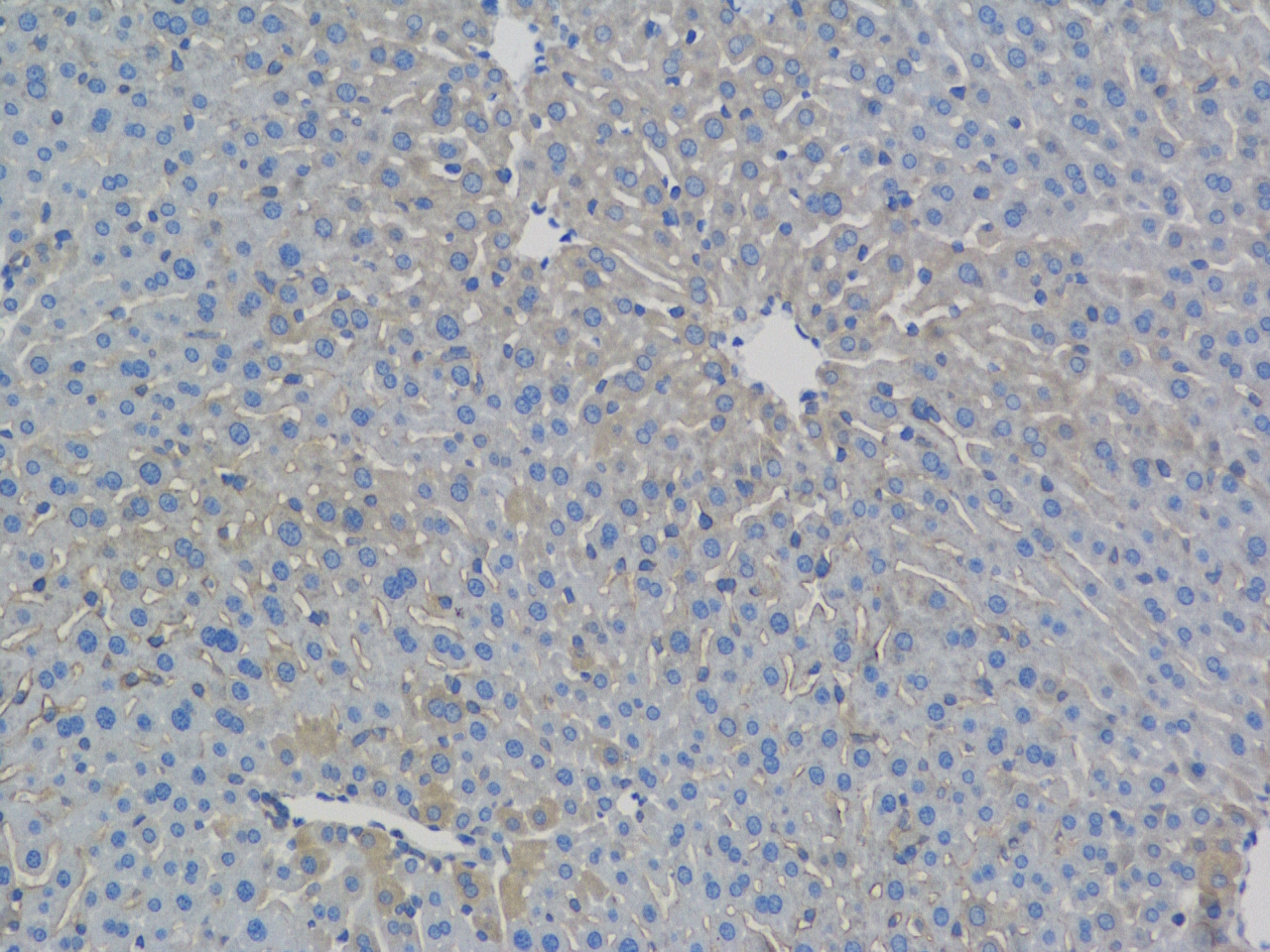

Supplement: Supplementary file 1 [file toxins-18-00278-s001.zip › Figure S6. Uncropped full-size hepatic tissue immunostaining images corresponding to Figure 8C/NnV+EGCG-Cleaved Caspase.jpg]

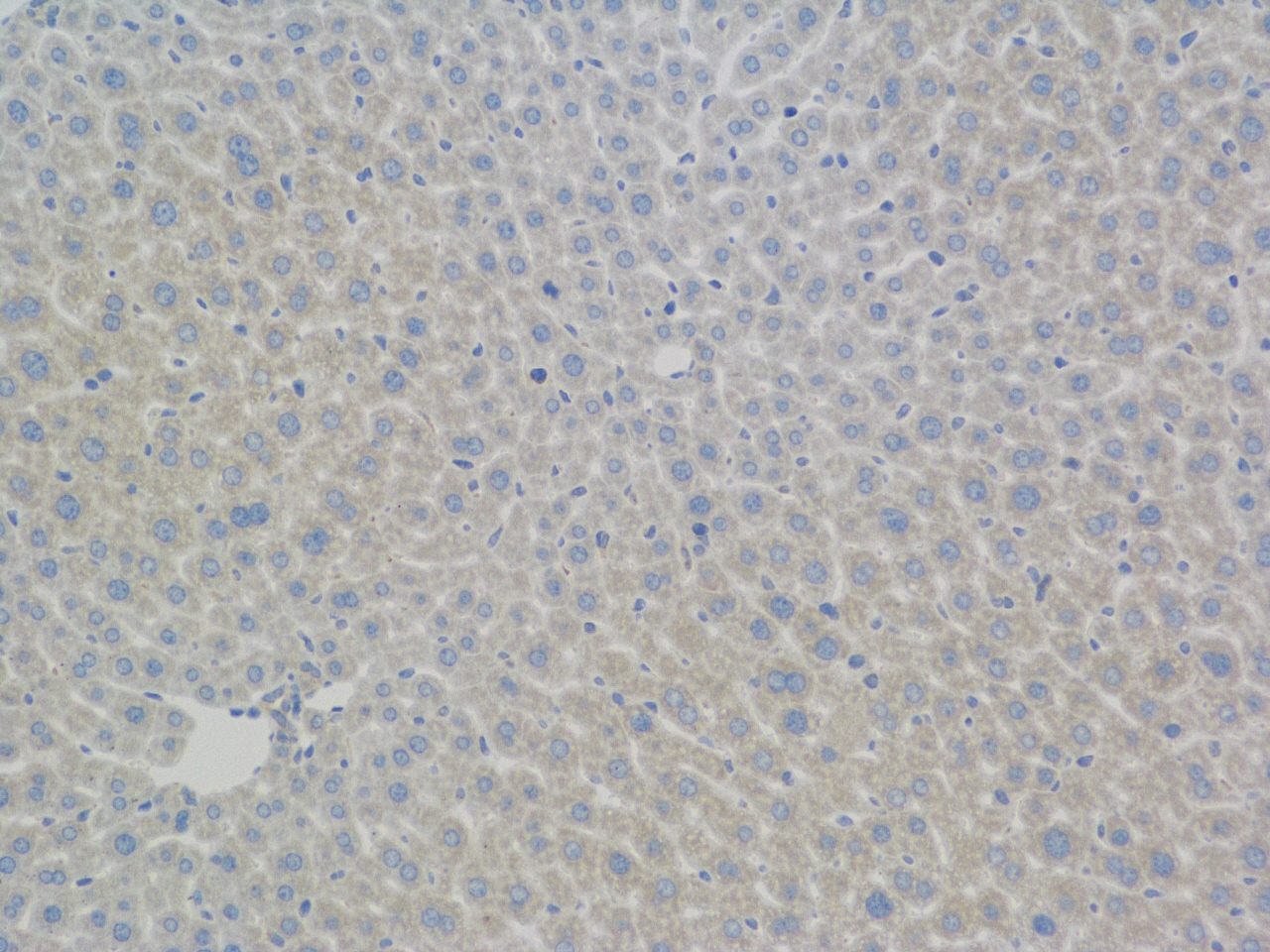

Supplement: Supplementary file 1 [file toxins-18-00278-s001.zip › Figure S6. Uncropped full-size hepatic tissue immunostaining images corresponding to Figure 8C/NnV+EGCG-Cleaved PARP.jpg]

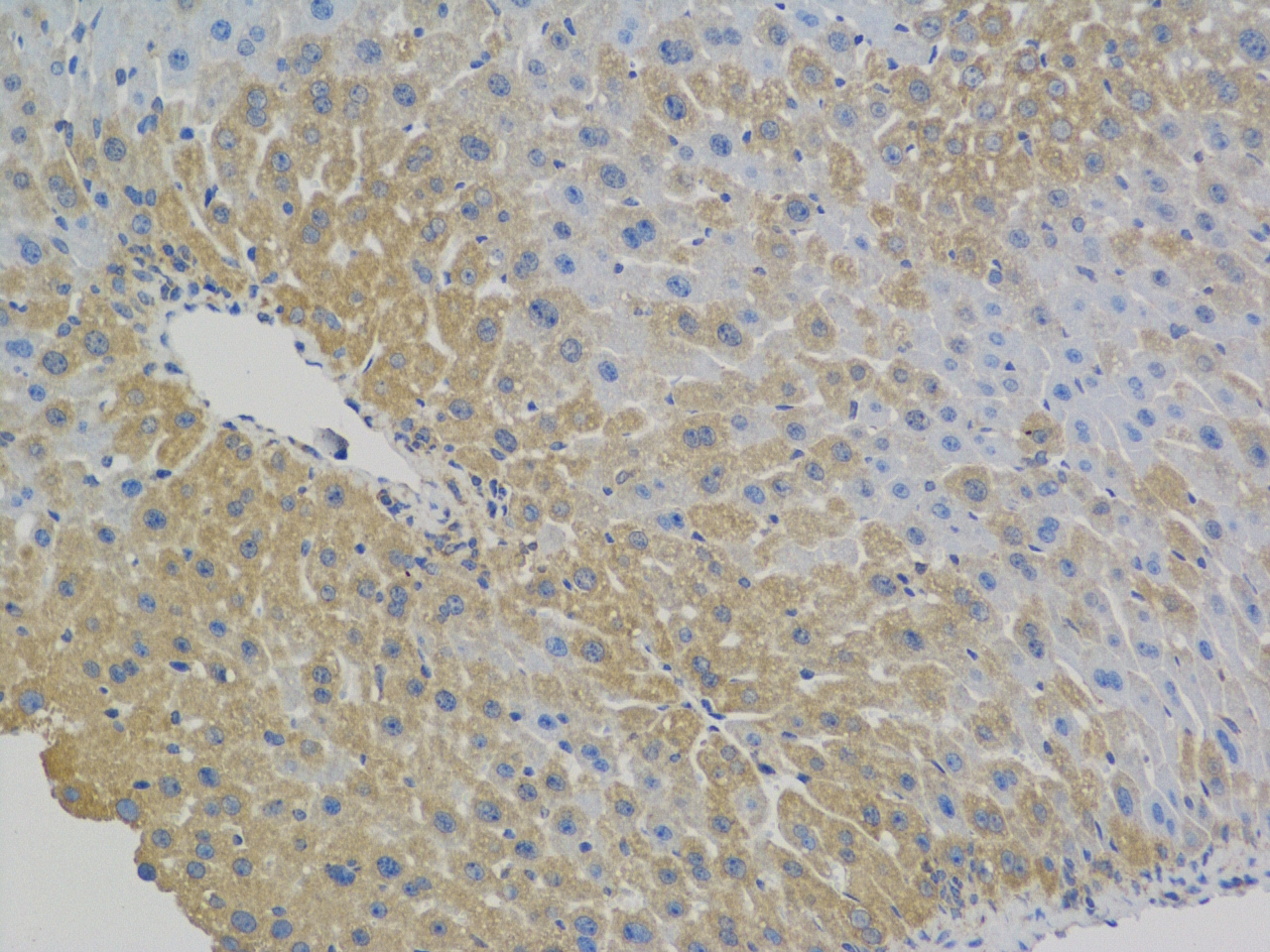

Supplement: Supplementary file 1 [file toxins-18-00278-s001.zip › Figure S6. Uncropped full-size hepatic tissue immunostaining images corresponding to Figure 8C/NnV-Bax.jpg]

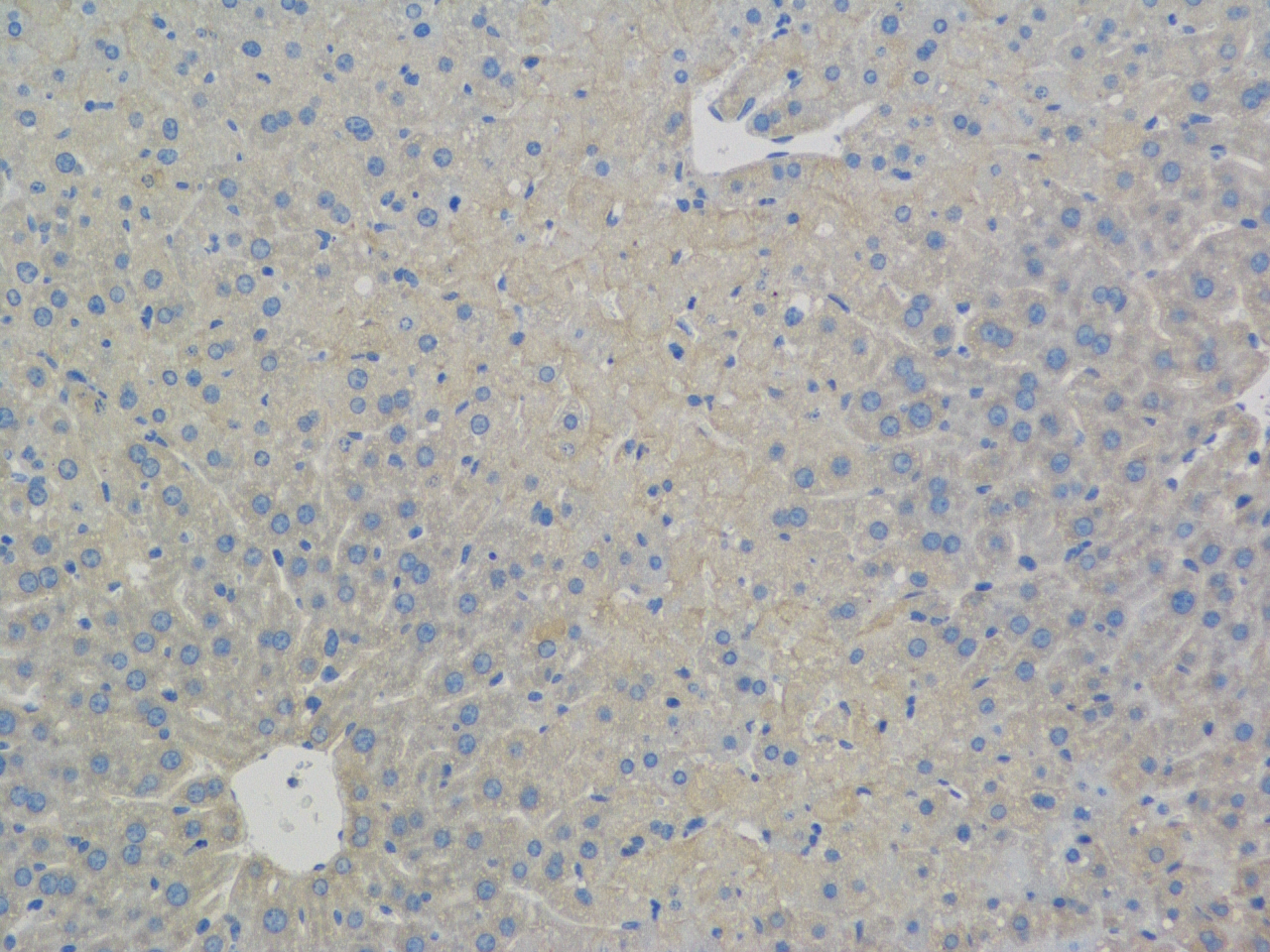

Supplement: Supplementary file 1 [file toxins-18-00278-s001.zip › Figure S6. Uncropped full-size hepatic tissue immunostaining images corresponding to Figure 8C/NnV-Bcl-xl.jpg]

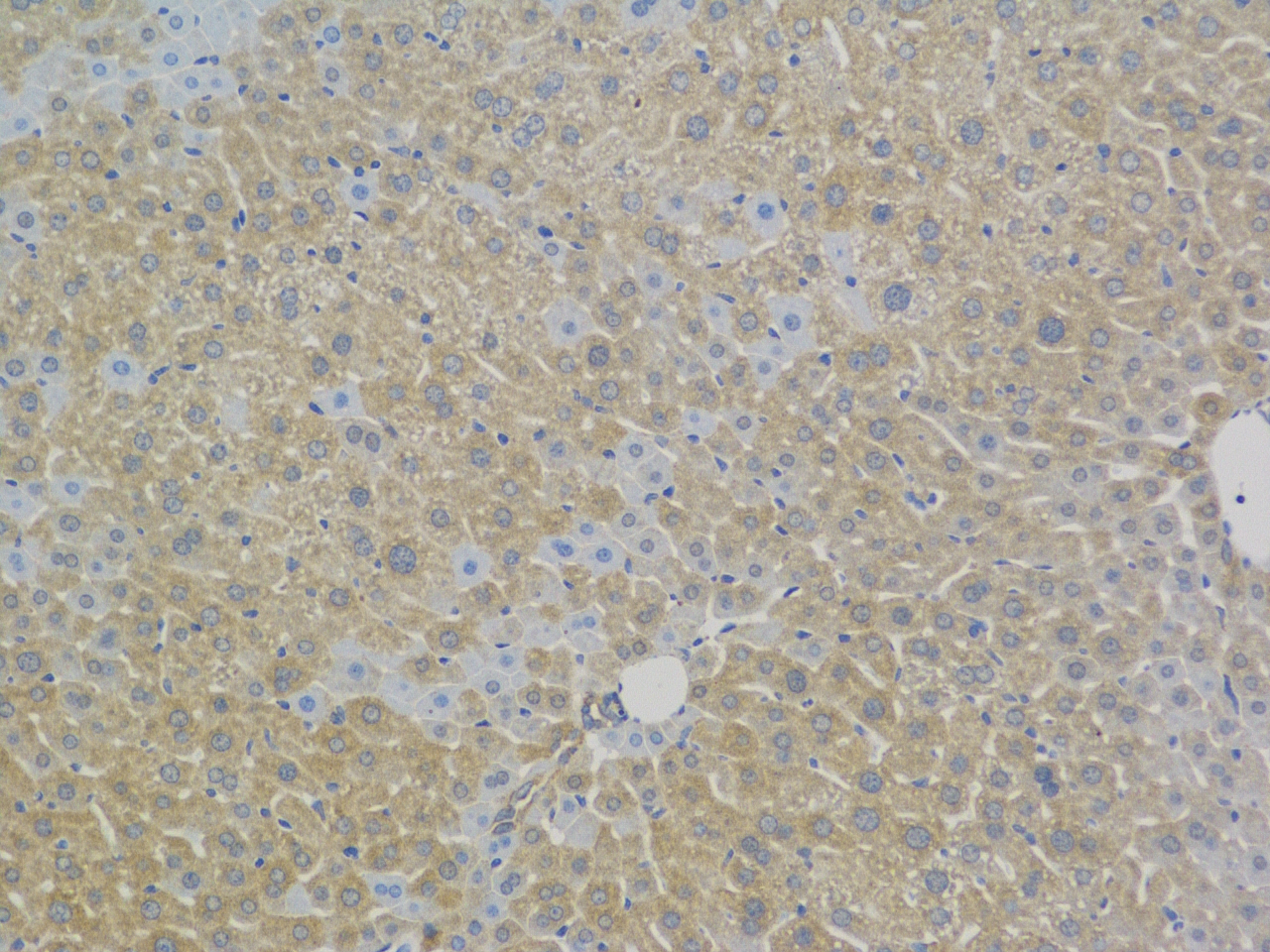

Supplement: Supplementary file 1 [file toxins-18-00278-s001.zip › Figure S6. Uncropped full-size hepatic tissue immunostaining images corresponding to Figure 8C/NnV-Cleaved Caspase-3.jpg]

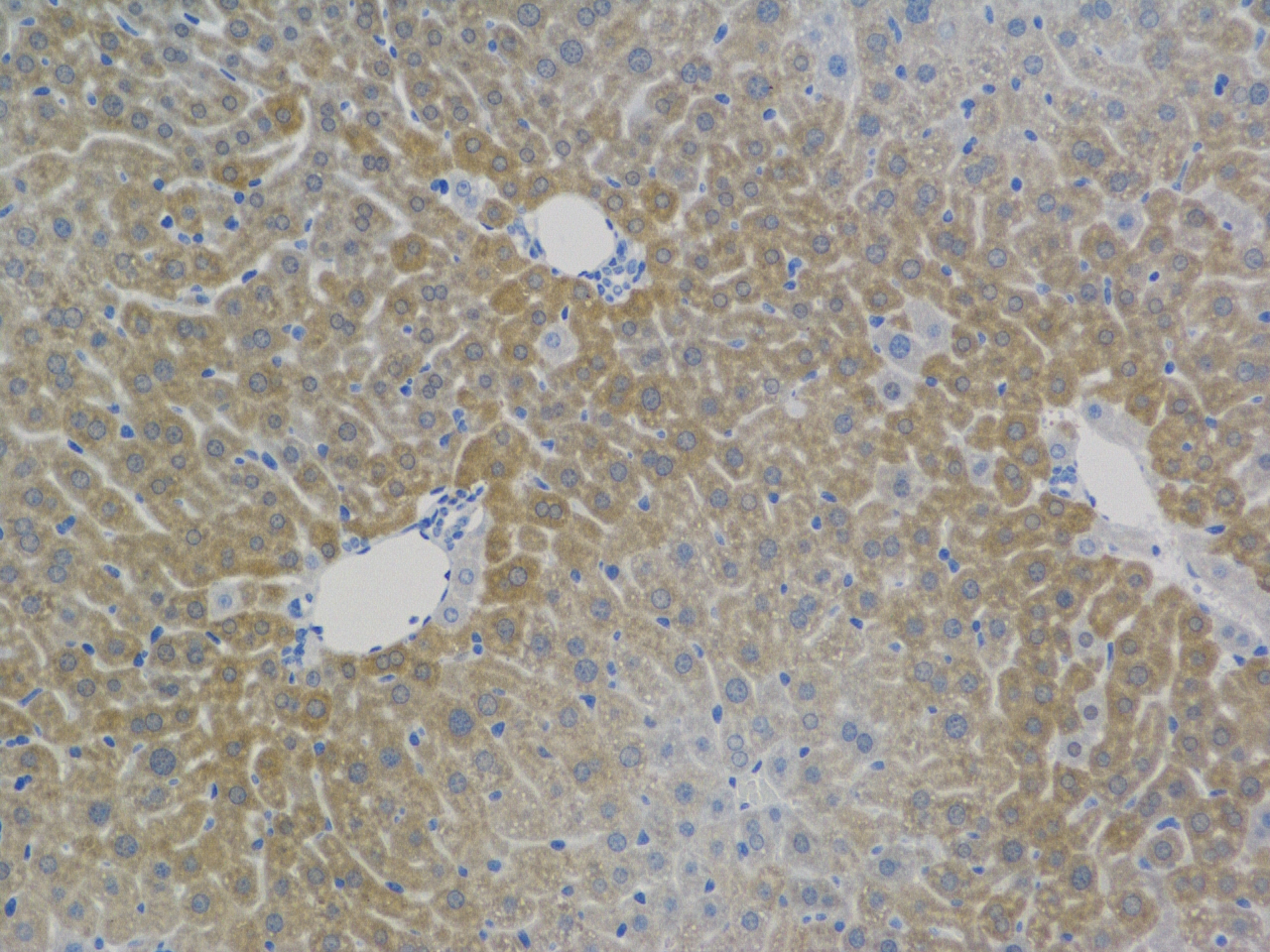

Supplement: Supplementary file 1 [file toxins-18-00278-s001.zip › Figure S6. Uncropped full-size hepatic tissue immunostaining images corresponding to Figure 8C/NnV-Cleaved PARP.jpg]

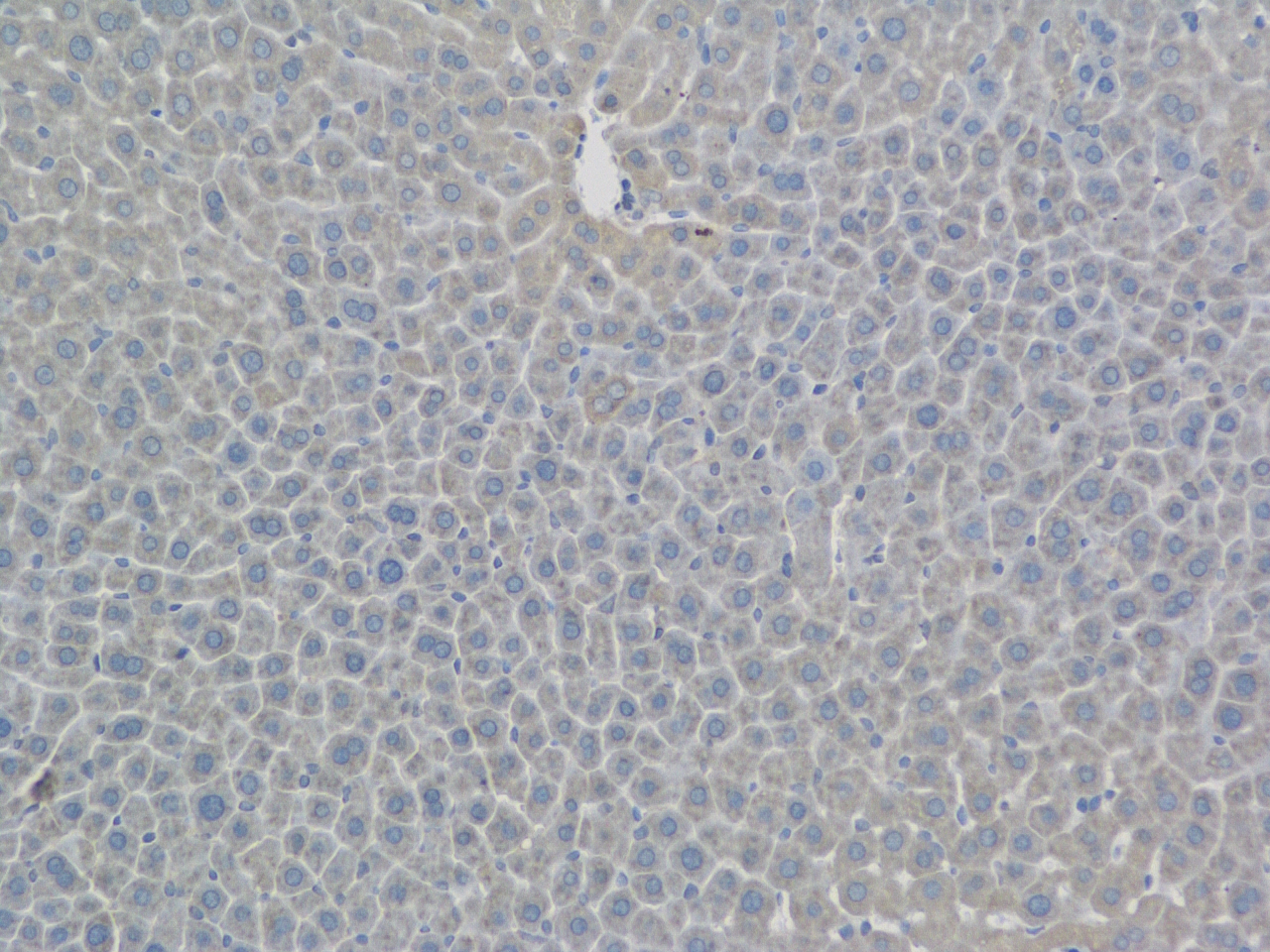

Supplement: Supplementary file 1 [file toxins-18-00278-s001.zip › Figure S6. Uncropped full-size hepatic tissue immunostaining images corresponding to Figure 8C/PBS-Bax.jpg]

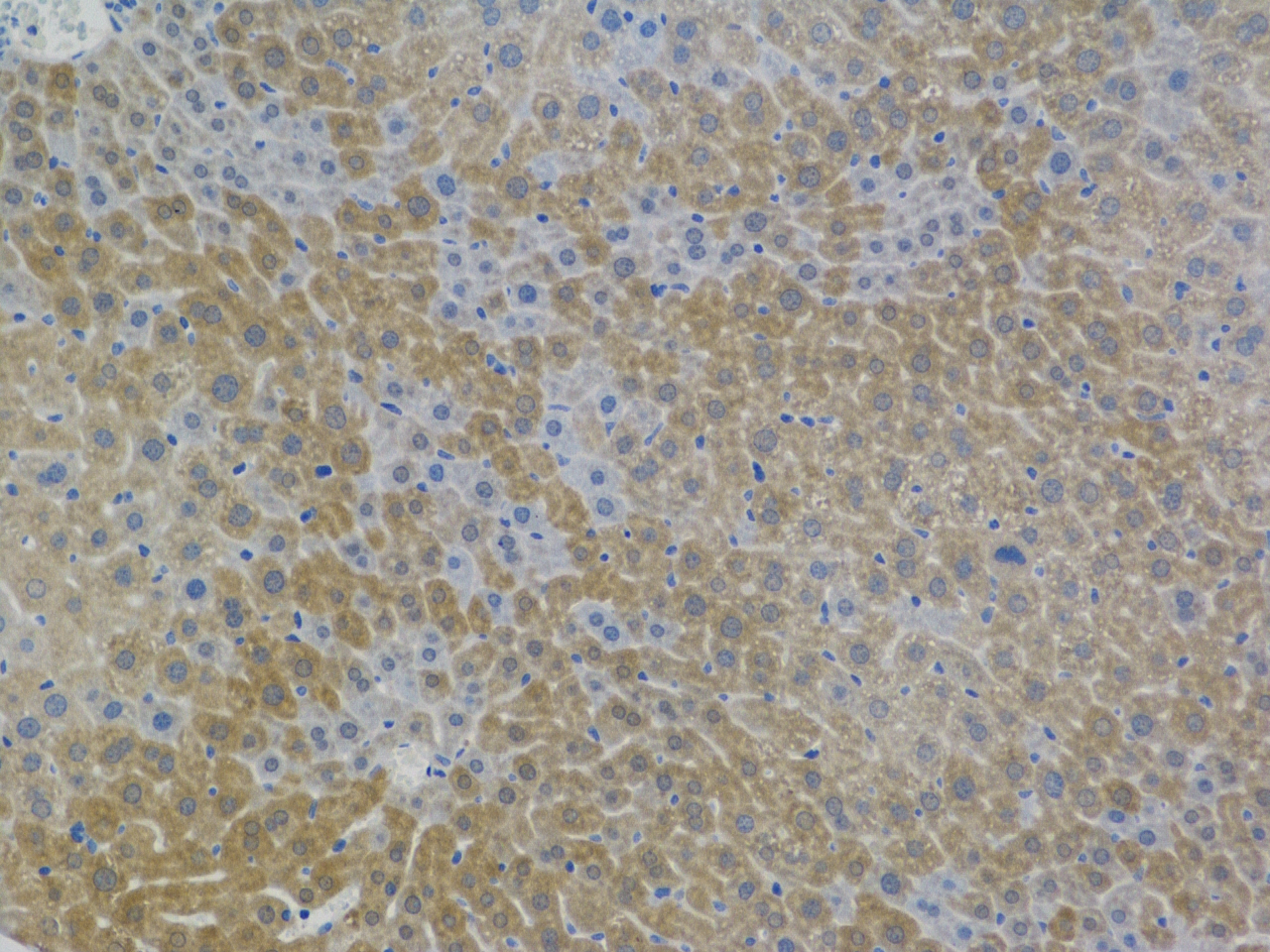

Supplement: Supplementary file 1 [file toxins-18-00278-s001.zip › Figure S6. Uncropped full-size hepatic tissue immunostaining images corresponding to Figure 8C/PBS-Bcl-xl.jpg]

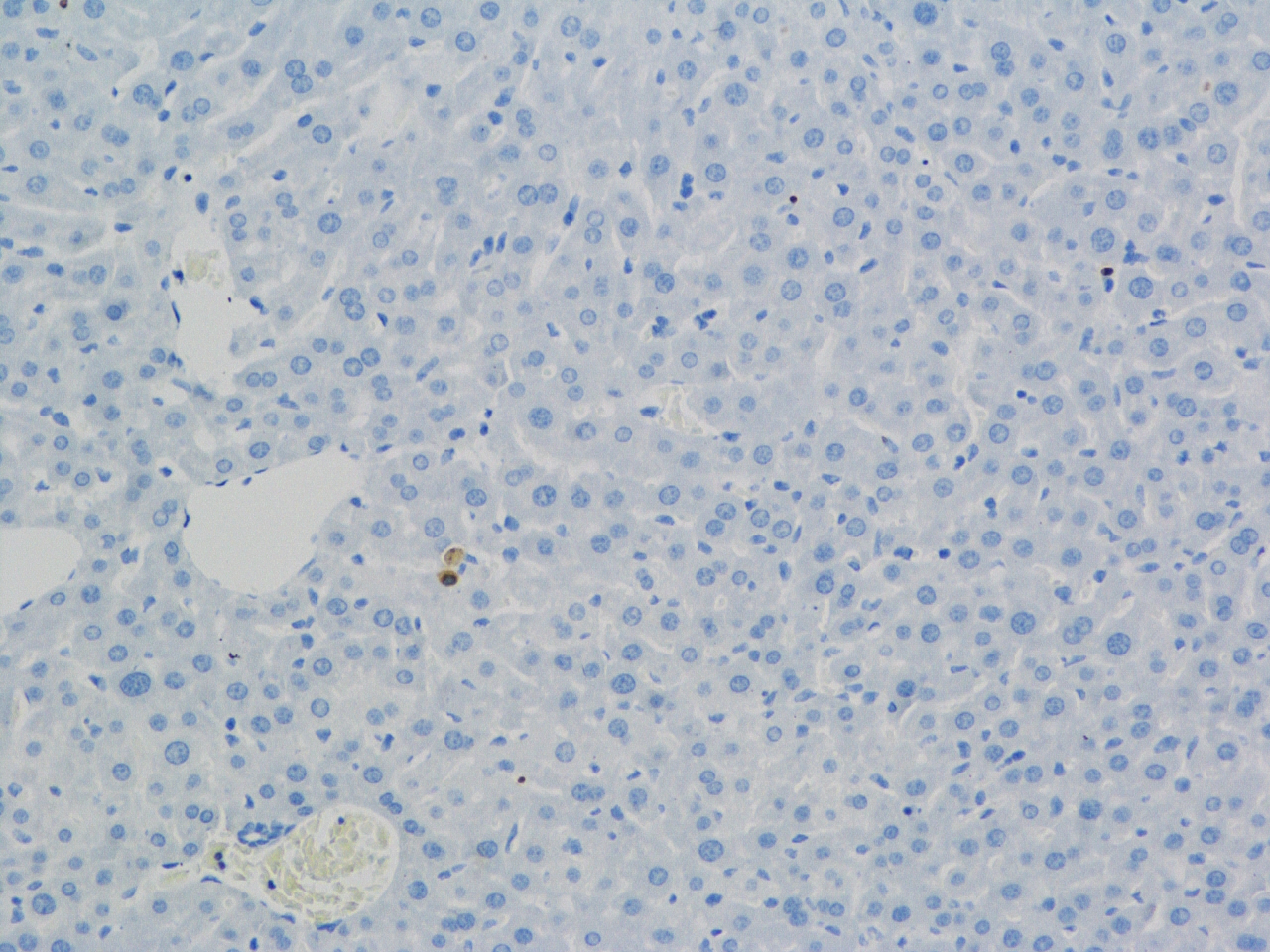

Supplement: Supplementary file 1 [file toxins-18-00278-s001.zip › Figure S6. Uncropped full-size hepatic tissue immunostaining images corresponding to Figure 8C/PBS-Cleaved Caspase-3.jpg]

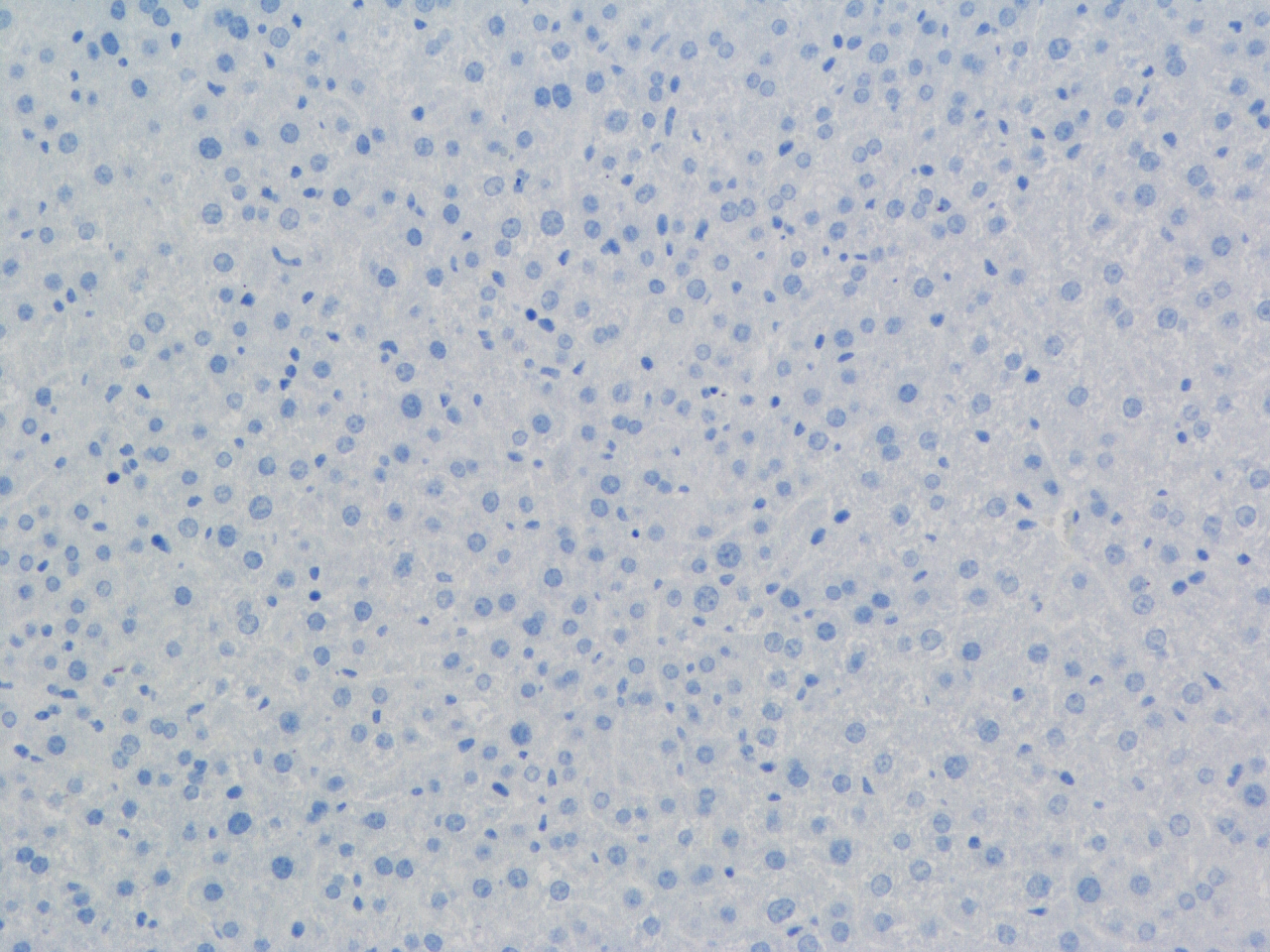

Supplement: Supplementary file 1 [file toxins-18-00278-s001.zip › Figure S6. Uncropped full-size hepatic tissue immunostaining images corresponding to Figure 8C/PBS-Cleaved PARP.jpg]

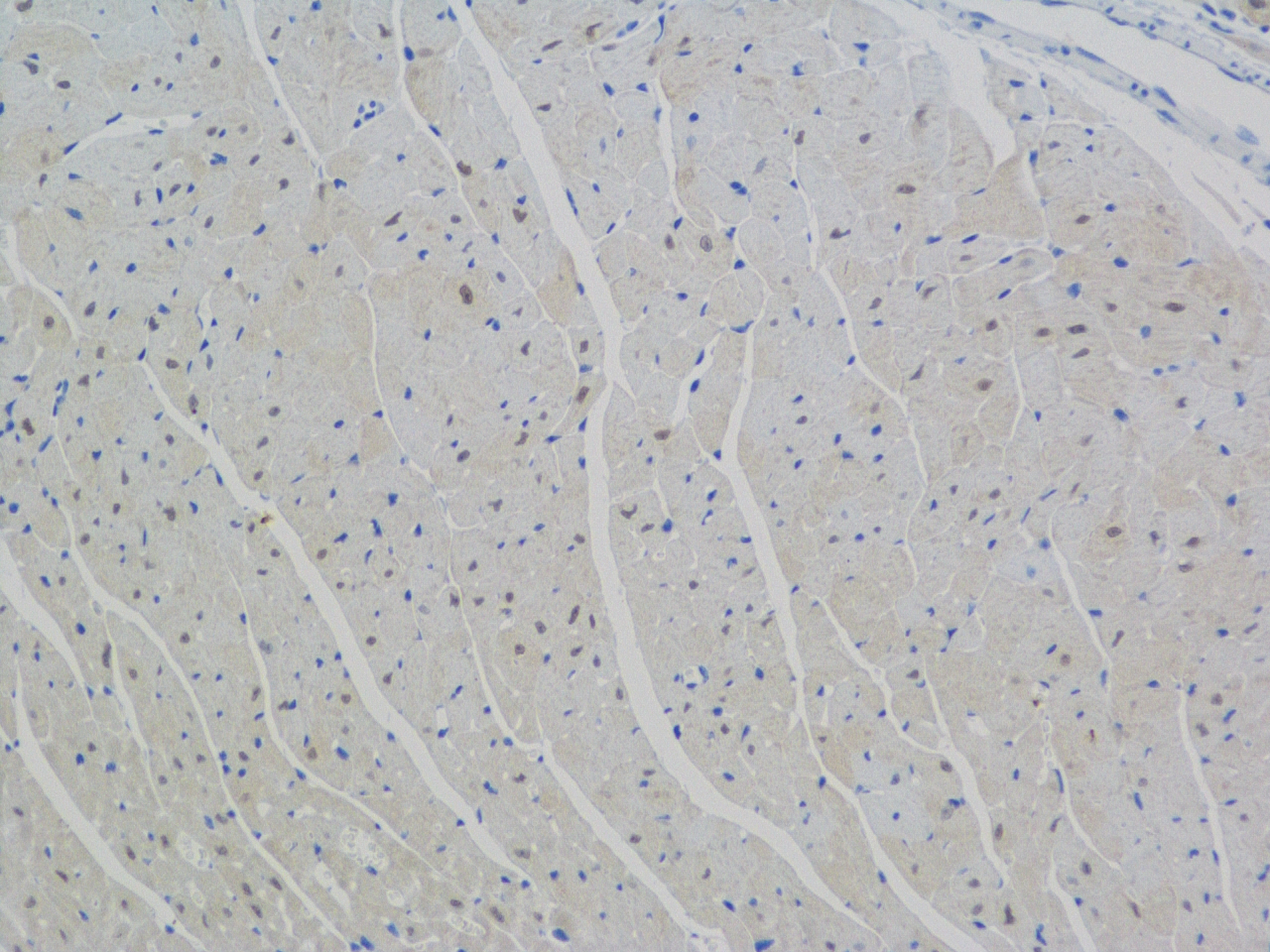

Supplement: Supplementary file 1 [file toxins-18-00278-s001.zip › Figure S1. Uncropped original full-size cardiac tissue immunostaining images corresponding to Figure 3C/EGCG-CAT.jpg]

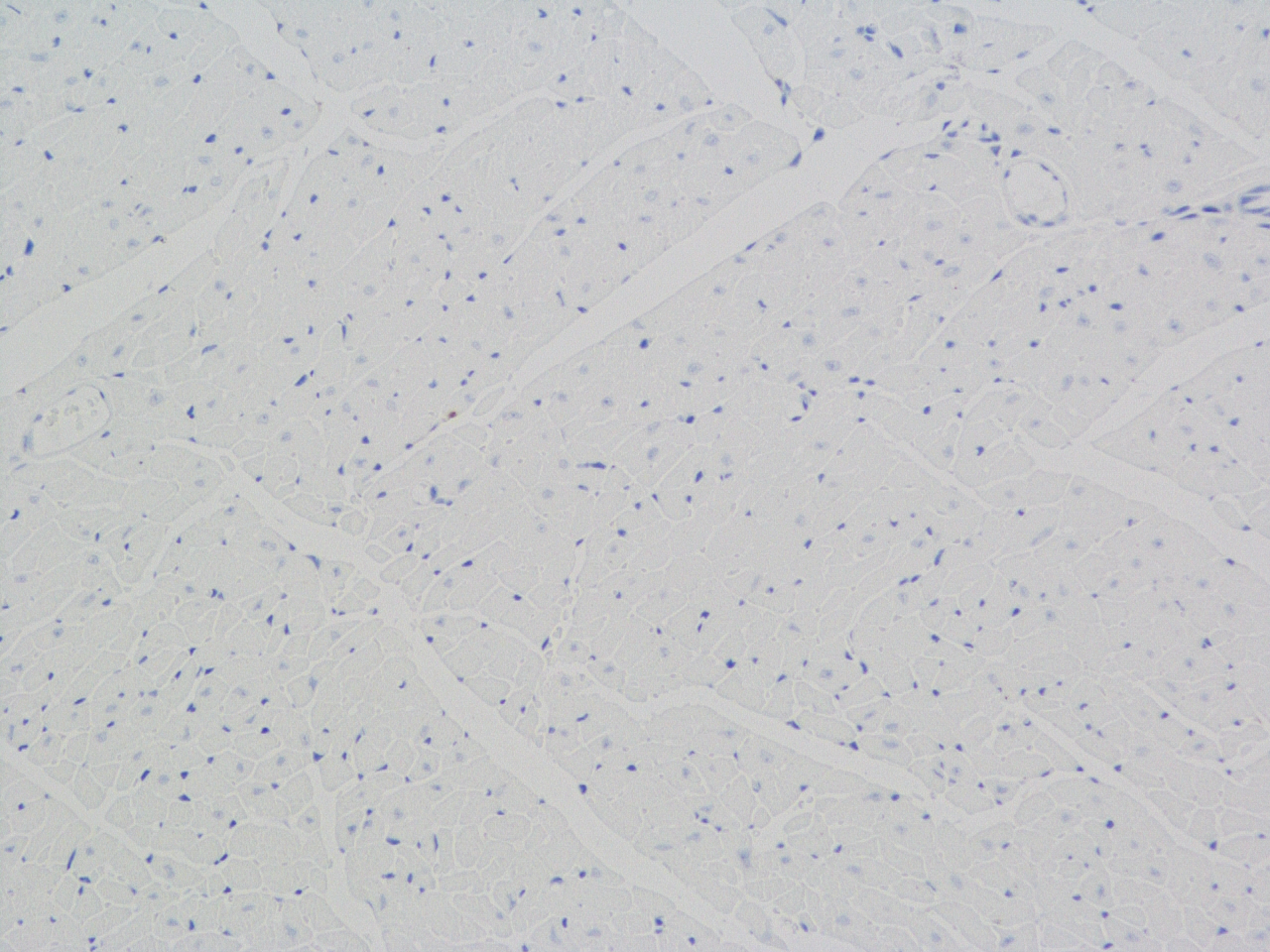

Supplement: Supplementary file 1 [file toxins-18-00278-s001.zip › Figure S1. Uncropped original full-size cardiac tissue immunostaining images corresponding to Figure 3C/EGCG-GPX4.jpg]

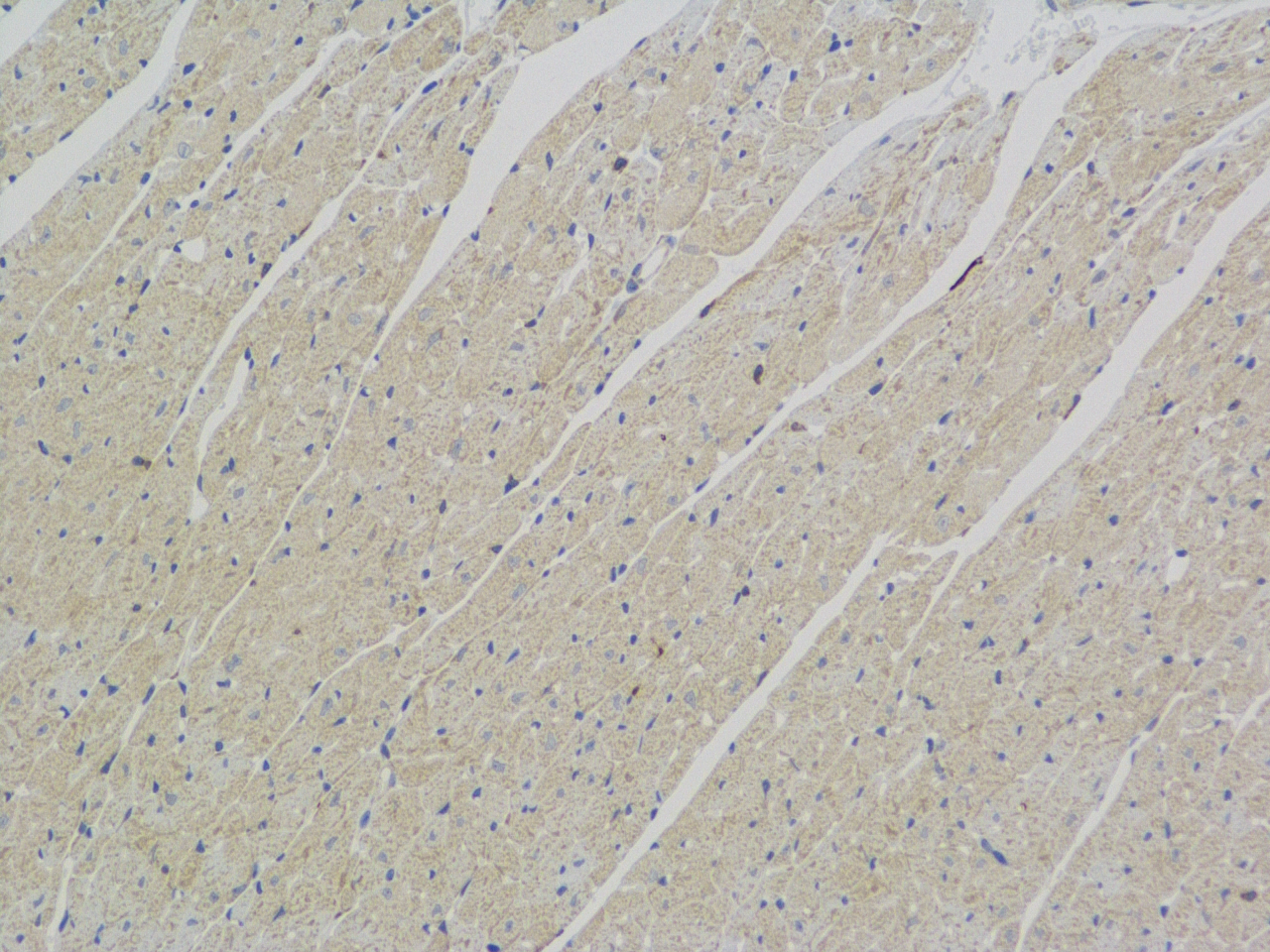

Supplement: Supplementary file 1 [file toxins-18-00278-s001.zip › Figure S1. Uncropped original full-size cardiac tissue immunostaining images corresponding to Figure 3C/EGCG-HO-1.jpg]

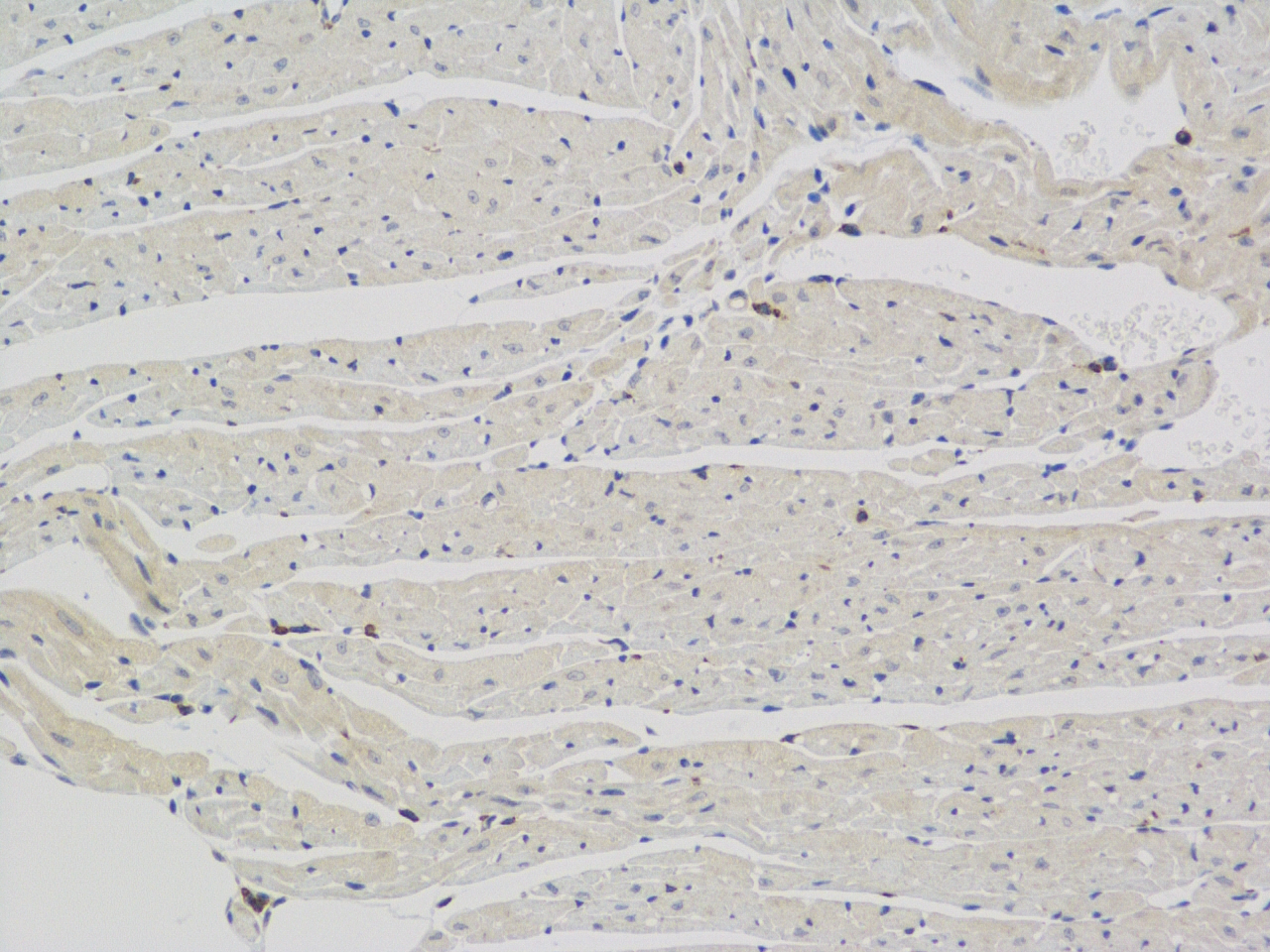

Supplement: Supplementary file 1 [file toxins-18-00278-s001.zip › Figure S1. Uncropped original full-size cardiac tissue immunostaining images corresponding to Figure 3C/EGCG-Nrf2.jpg]

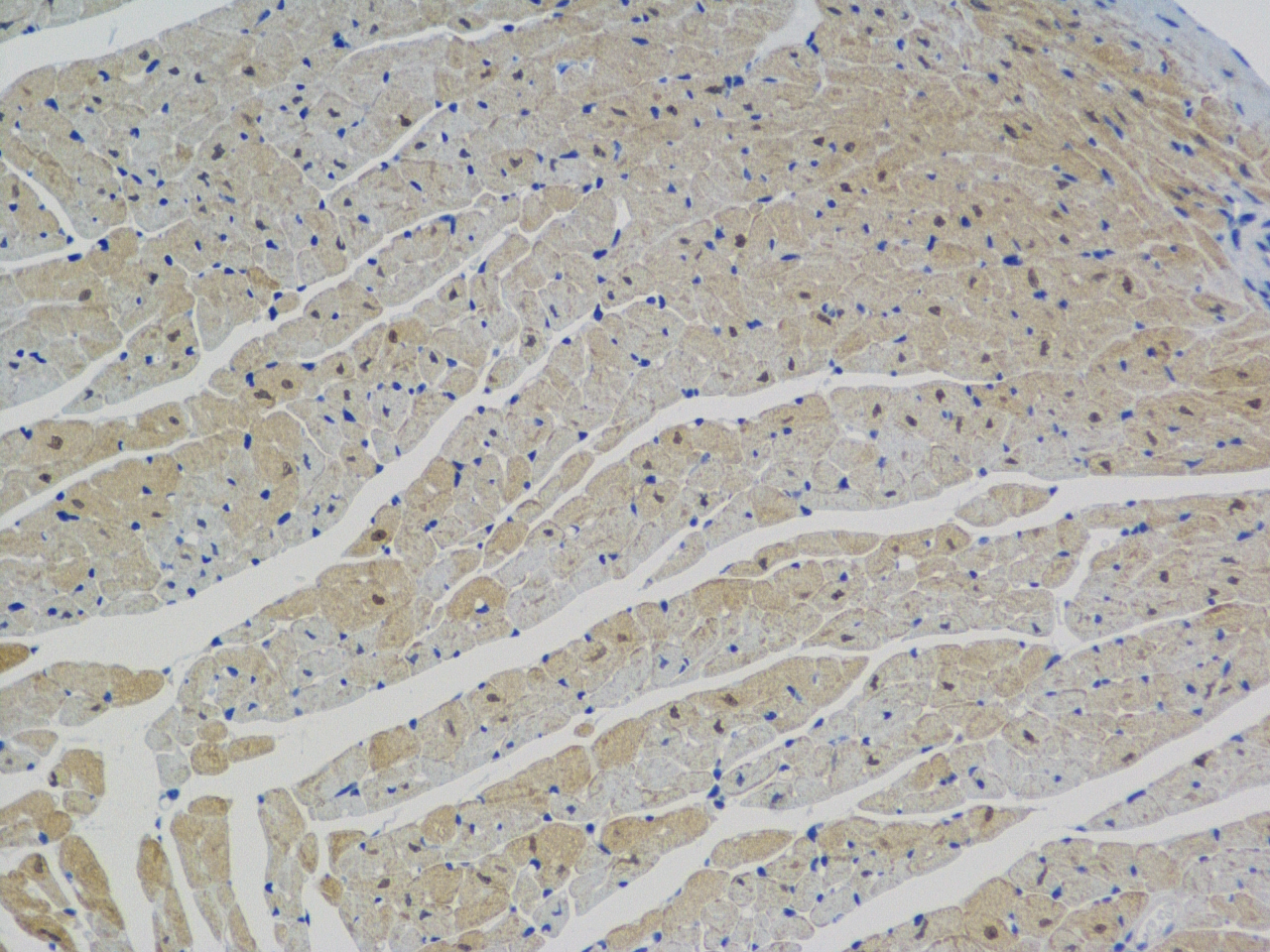

Supplement: Supplementary file 1 [file toxins-18-00278-s001.zip › Figure S1. Uncropped original full-size cardiac tissue immunostaining images corresponding to Figure 3C/NnV+EGCG-CAT.jpg]

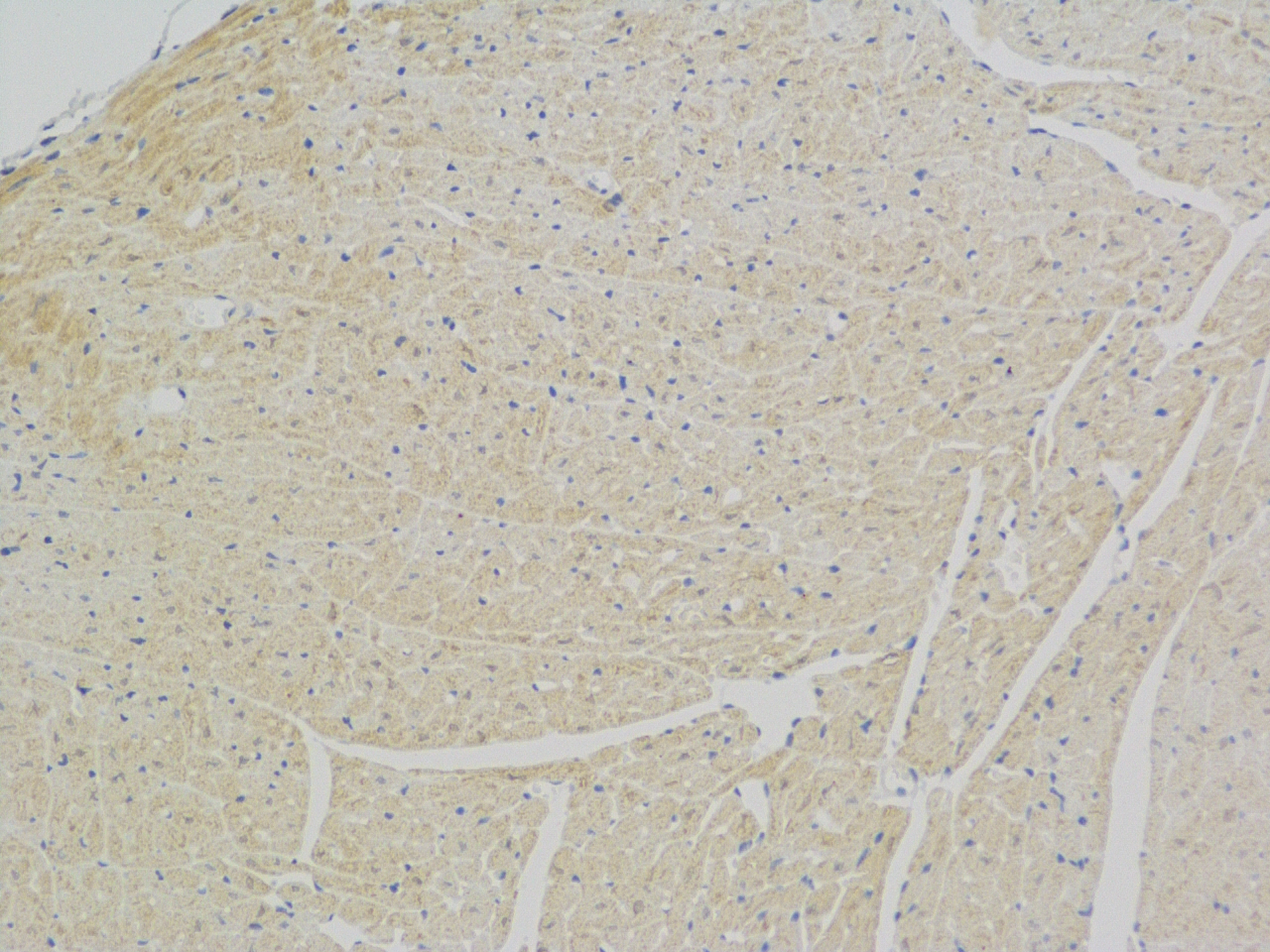

Supplement: Supplementary file 1 [file toxins-18-00278-s001.zip › Figure S1. Uncropped original full-size cardiac tissue immunostaining images corresponding to Figure 3C/NnV+EGCG-GPX4.jpg]

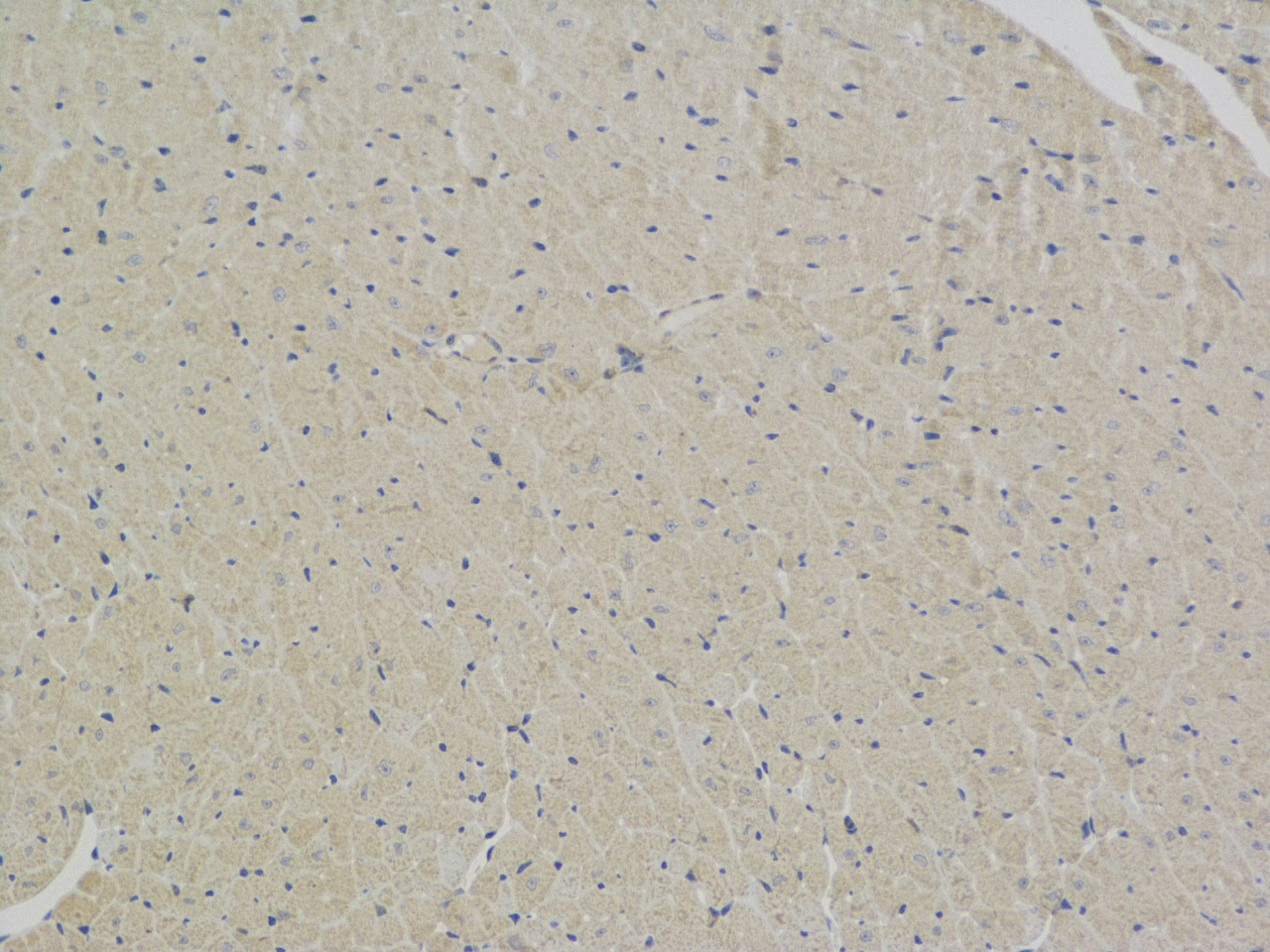

Supplement: Supplementary file 1 [file toxins-18-00278-s001.zip › Figure S1. Uncropped original full-size cardiac tissue immunostaining images corresponding to Figure 3C/NnV+EGCG-HO-1.jpg]

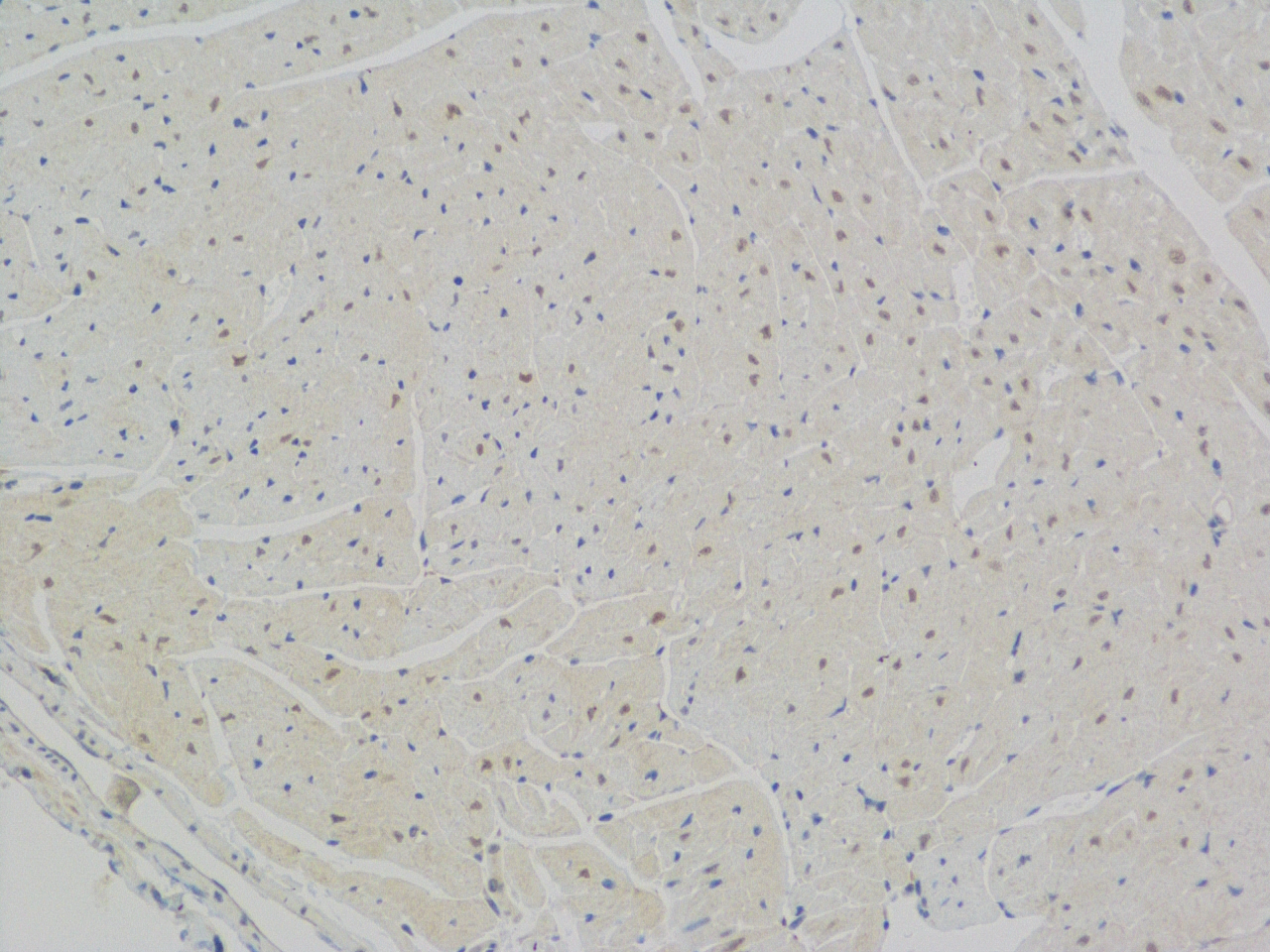

Supplement: Supplementary file 1 [file toxins-18-00278-s001.zip › Figure S1. Uncropped original full-size cardiac tissue immunostaining images corresponding to Figure 3C/NnV+EGCG-Nrf2.jpg]

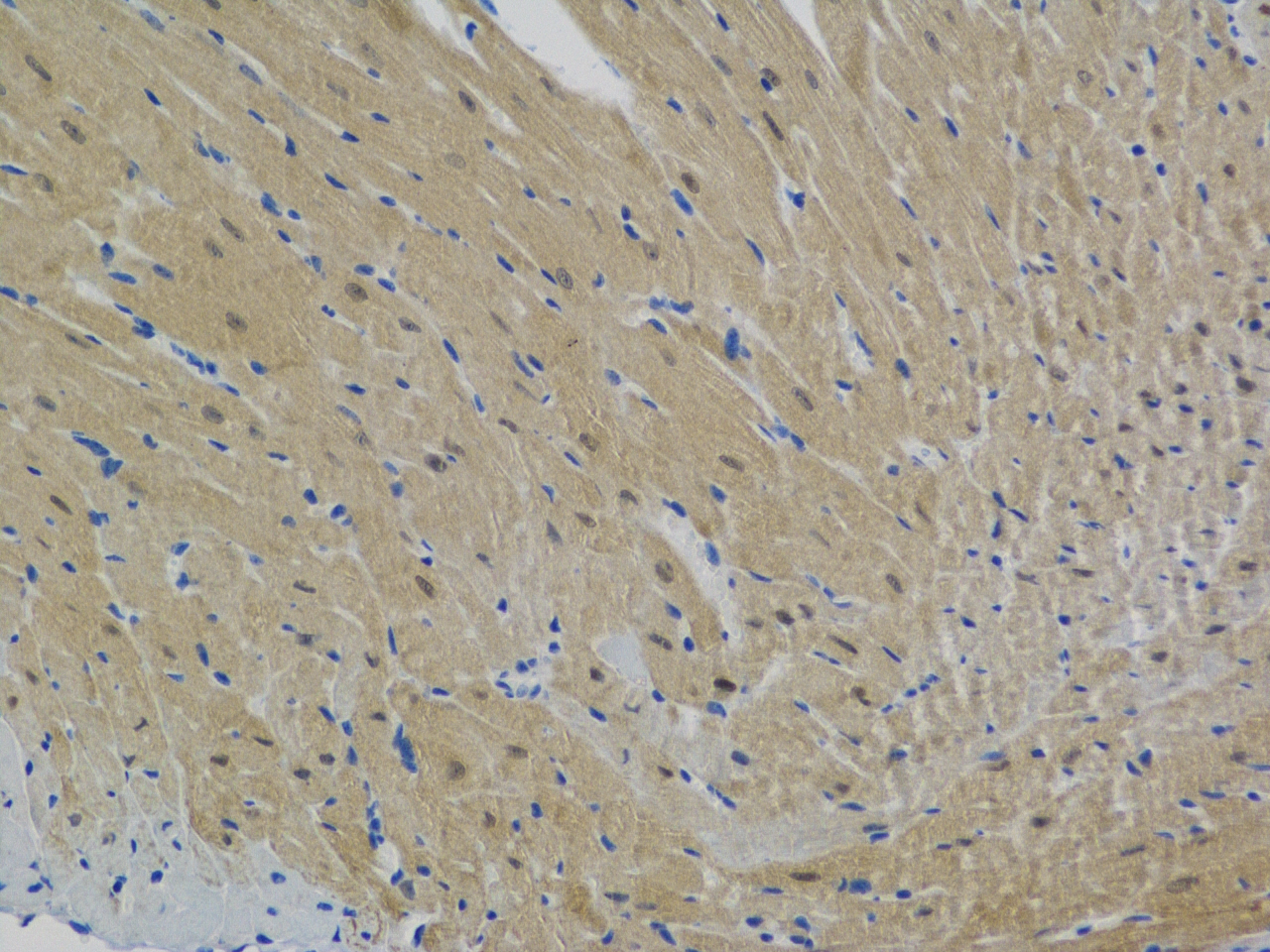

Supplement: Supplementary file 1 [file toxins-18-00278-s001.zip › Figure S1. Uncropped original full-size cardiac tissue immunostaining images corresponding to Figure 3C/NnV-CAT.jpg]

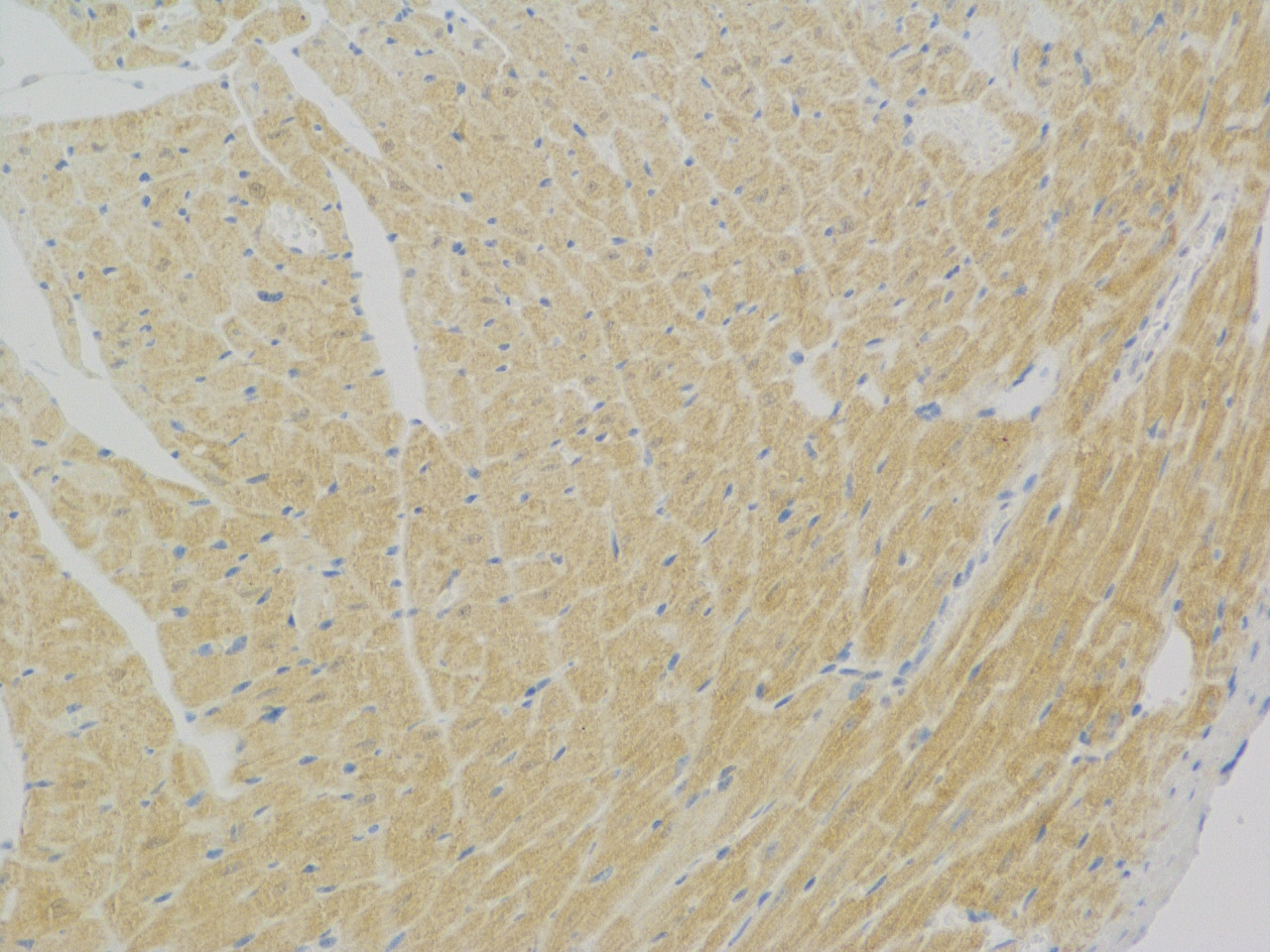

Supplement: Supplementary file 1 [file toxins-18-00278-s001.zip › Figure S1. Uncropped original full-size cardiac tissue immunostaining images corresponding to Figure 3C/NnV-GPX4.jpg]

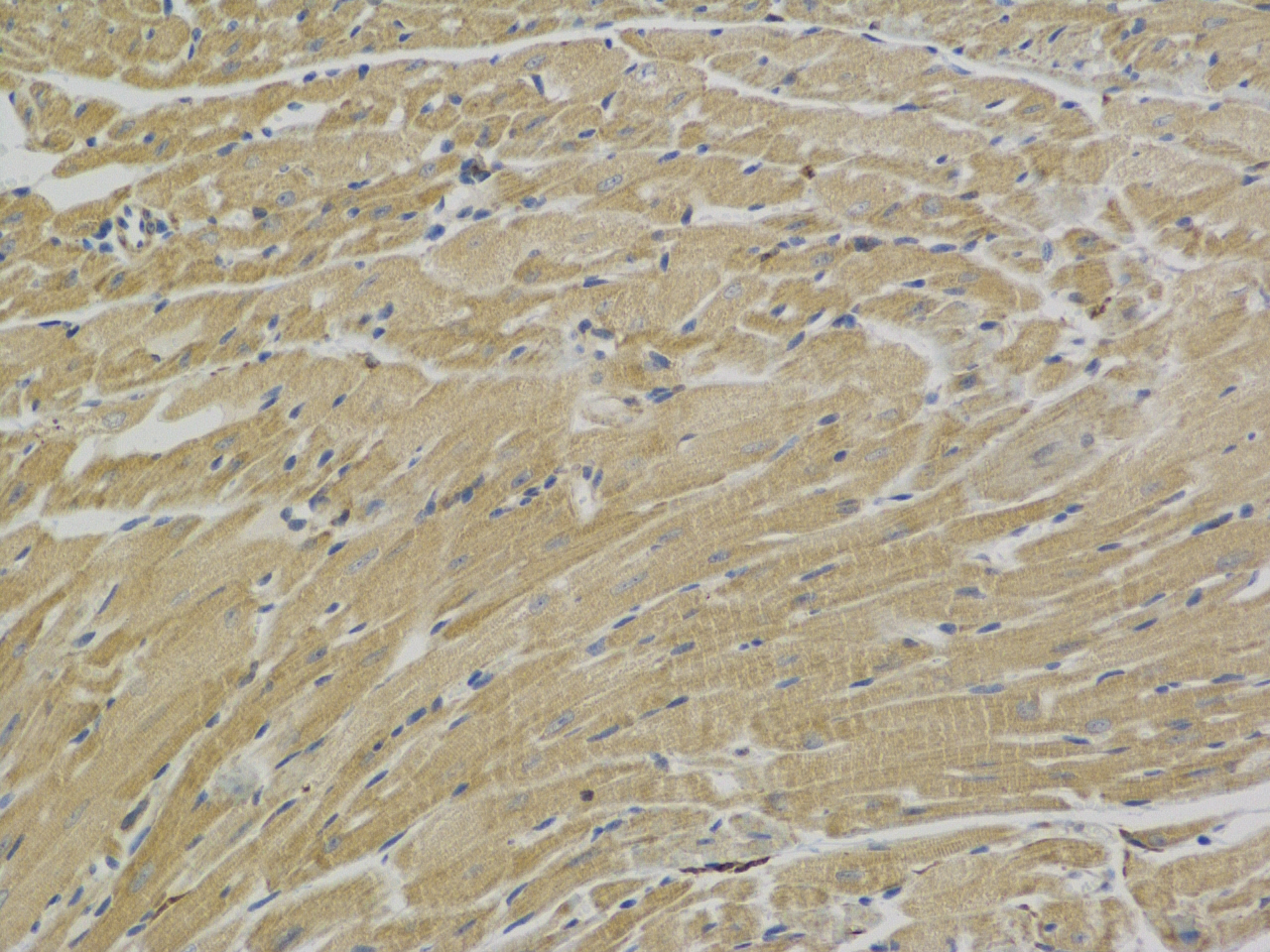

Supplement: Supplementary file 1 [file toxins-18-00278-s001.zip › Figure S1. Uncropped original full-size cardiac tissue immunostaining images corresponding to Figure 3C/NnV-HO-1.jpg]

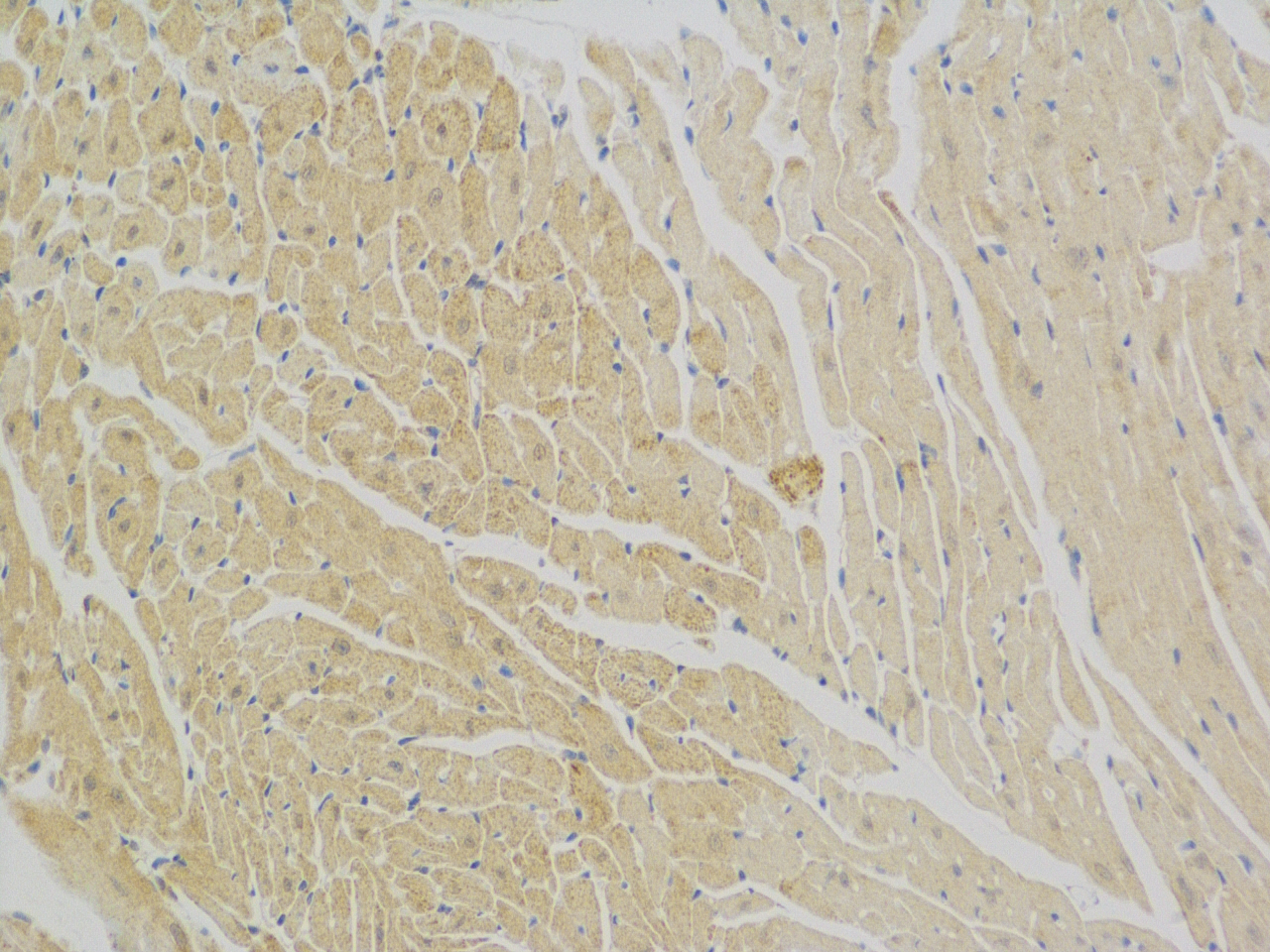

Supplement: Supplementary file 1 [file toxins-18-00278-s001.zip › Figure S1. Uncropped original full-size cardiac tissue immunostaining images corresponding to Figure 3C/NnV-Nrf2.jpg]

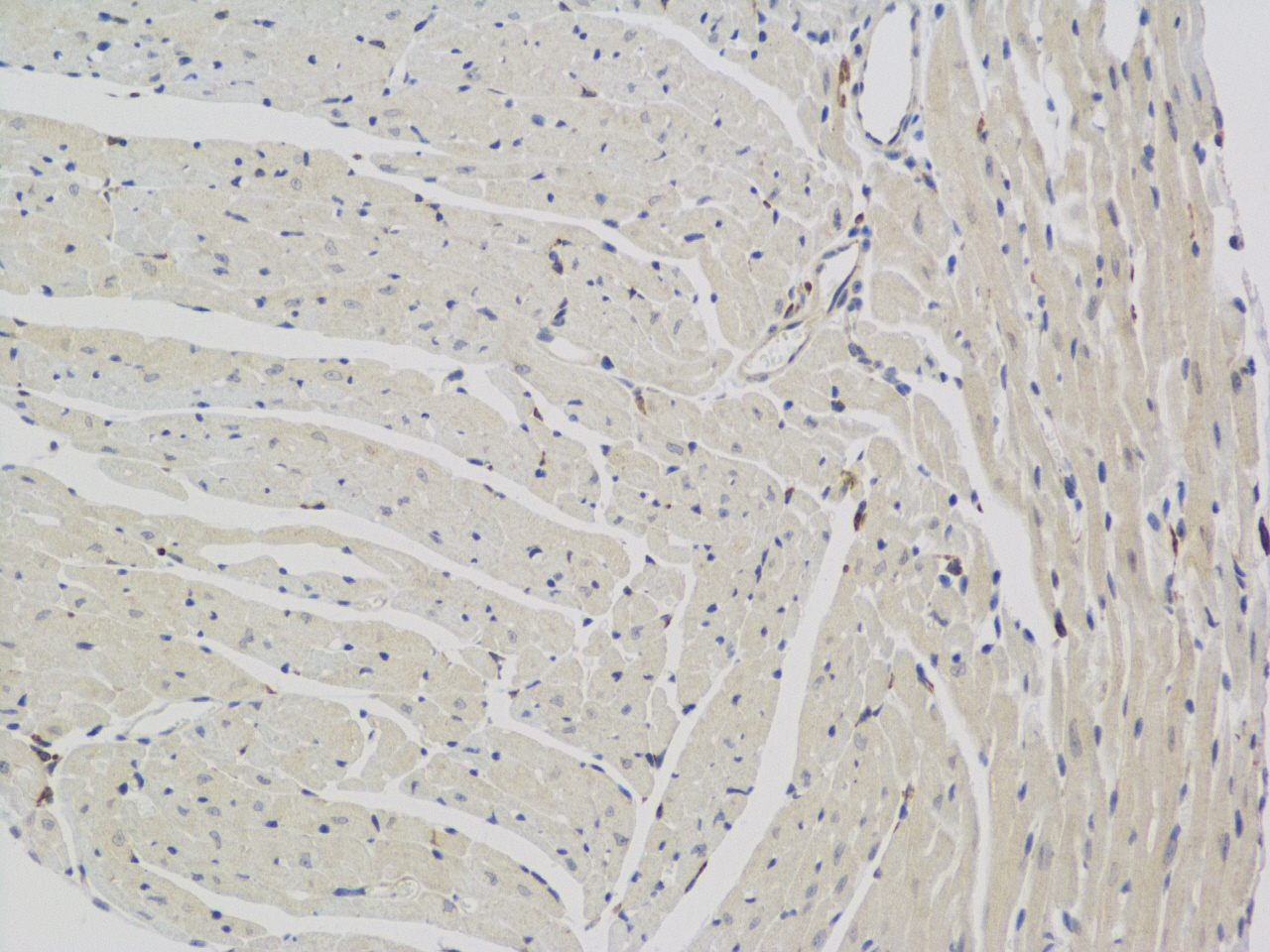

Supplement: Supplementary file 1 [file toxins-18-00278-s001.zip › Figure S1. Uncropped original full-size cardiac tissue immunostaining images corresponding to Figure 3C/PBS-CAT.jpg]

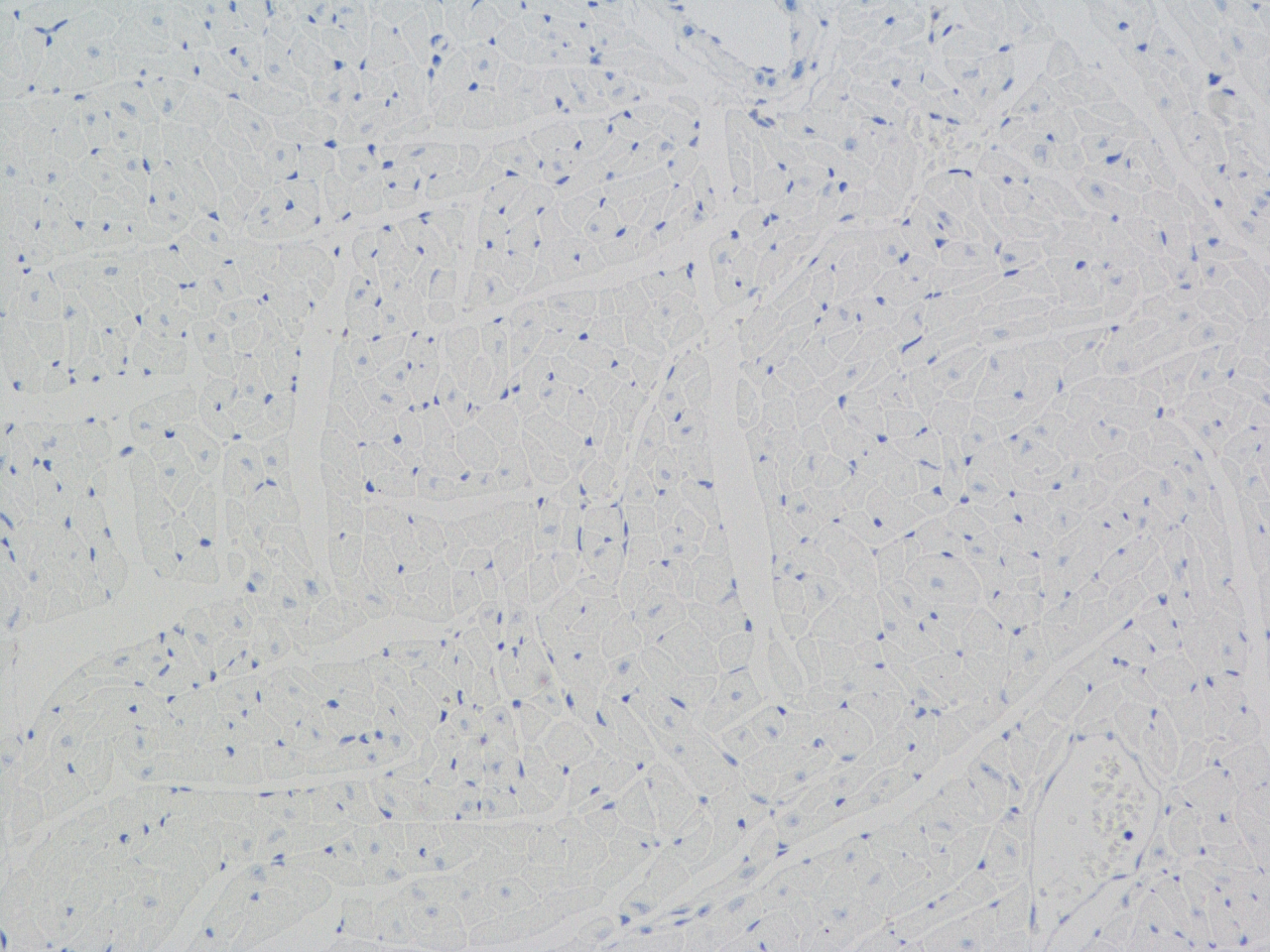

Supplement: Supplementary file 1 [file toxins-18-00278-s001.zip › Figure S1. Uncropped original full-size cardiac tissue immunostaining images corresponding to Figure 3C/PBS-GPX4.jpg]

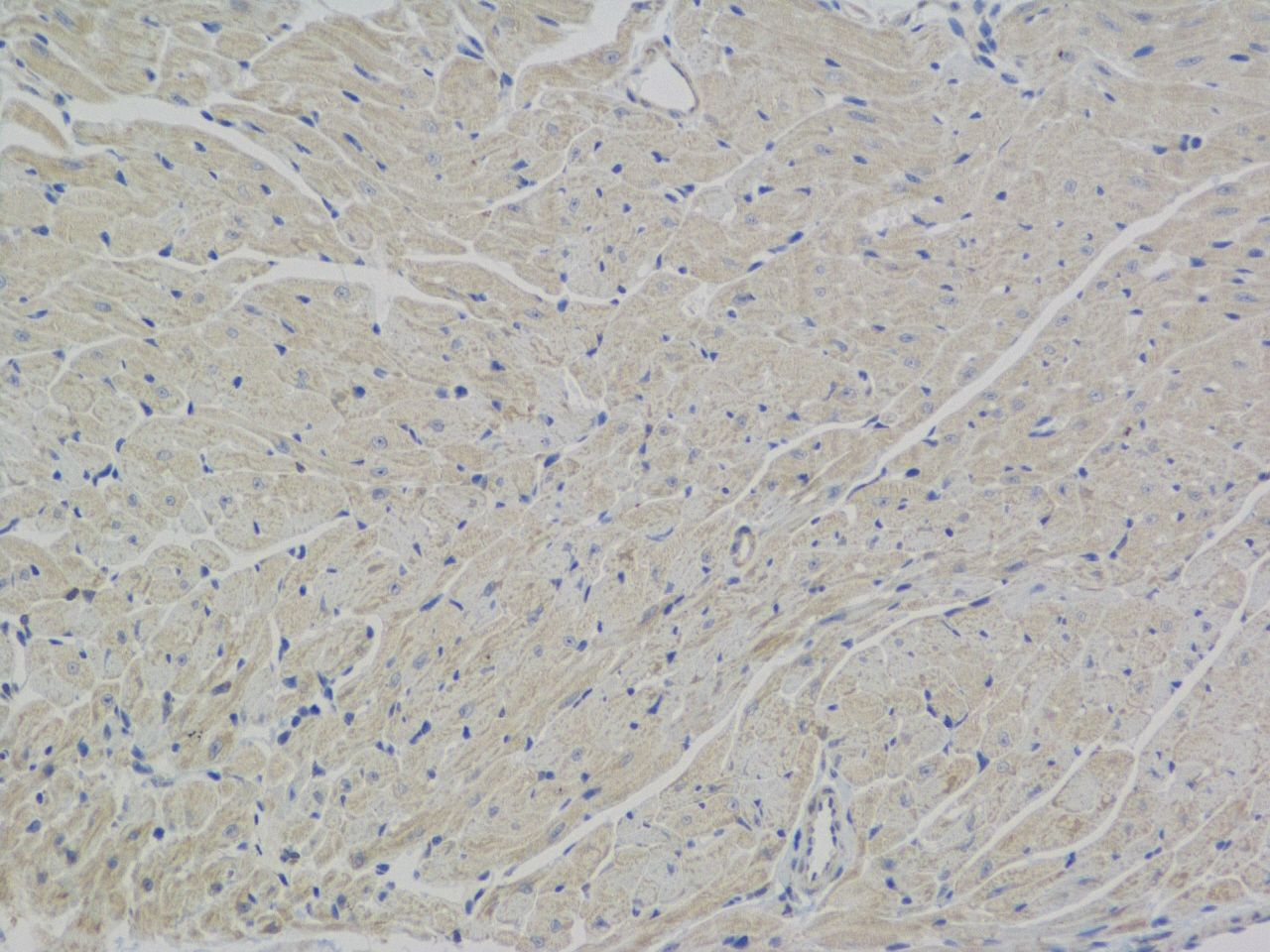

Supplement: Supplementary file 1 [file toxins-18-00278-s001.zip › Figure S1. Uncropped original full-size cardiac tissue immunostaining images corresponding to Figure 3C/PBS-HO-1.jpg]
